# Highly selective hydrogenation of amides catalysed by a molybdenum pincer complex: scope and mechanism†
†Electronic supplementary information (ESI) available. See DOI: 10.1039/c9sc03453f


**DOI:** 10.1039/c9sc03453f

**Published:** 2019-10-08

**Authors:** Thomas Leischner, Lluis Artús Suarez, Anke Spannenberg, Kathrin Junge, Ainara Nova, Matthias Beller

**Affiliations:** a Leibniz Institut für Katalyse e. V. , Albert-Einstein-Straße 29a , Rostock , 18059 , Germany . Email: Matthias.Beller@catalysis.de; b Hylleraas Centre for Quantum Molecular Sciences , Department of Chemistry , University of Oslo , P.O. Box 1033, Blindern , N-0315 , Oslo , Norway . Email: Ainara.nova@kjemi.uio.no

## Abstract

A series of molybdenum pincer complexes has been shown for the first time to be active in the catalytic hydrogenation of amides. Among the tested catalysts, **Mo-1a** proved to be particularly well suited for the selective C–N hydrogenolysis of *N*-methylated formanilides. Notably, high chemoselectivity was observed in the presence of certain reducible groups including even other amides. The general catalytic performance as well as selectivity issues could be rationalized taking an anionic Mo(0) as the active species. The interplay between the amide C=O reduction and the catalyst poisoning by primary amides accounts for the selective hydrogenation of *N*-methylated formanilides. The catalyst resting state was found to be a Mo–alkoxo complex formed by reaction with the alcohol product. This species plays two opposed roles – it facilitates the protolytic cleavage of the C–N bond but it encumbers the activation of hydrogen.

## Abstract 2

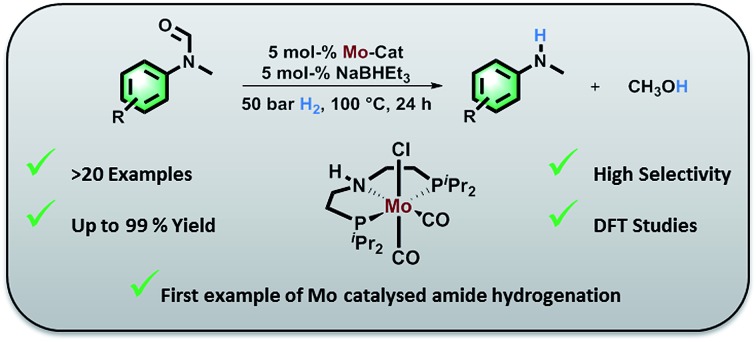
A series of molybdenum pincer complexes has been shown for the first time to be active in the catalytic hydrogenation of amides.

## Introduction

The reduction of carboxylic acid derivatives *via* catalytic homogeneous hydrogenation represents an attractive atom-economic and environmentally benign methodology.^1^,^2^ To date, the vast majority of homogeneous catalysts for these transformations rely on noble metals.^3^ The limited availability of these elements along with their toxicity and pollutive nature initiated efforts for their replacement. Significant progress in this direction has been achieved in the past decade, in particular with respect to iron,^4^ manganese^5^ and cobalt^6^ based systems. Thus, several examples of base metal catalysed hydrogenations of aldehydes, ketones, carboxylic acids, esters and nitriles have been reported in recent years, some of them with remarkable activities and selectivities.2a,^7^ On the contrary, hydrogenation of amides is known to a much less extent.^8^ The latter can be attributed to the extremely low electrophilicity of the carbonyl group, which renders their hydrogenation particularly challenging.

**Scheme 1:**
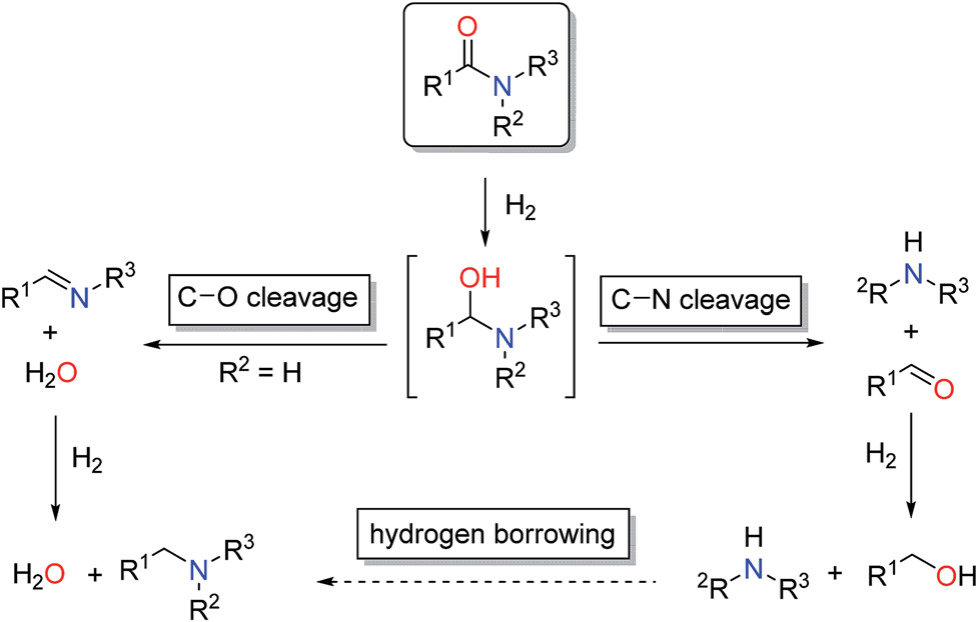
Pathways for amide reduction.

In general, catalytic hydrogenation of amides can proceed *via* either C–N (hydrogenolysis) or C–O (hydrogenation) bond cleavage of the intermediate hemiaminal (Scheme 1). While the C–O bond scission results in the formation of the alkylated/benzylated amine with H_2_O as the only by-product, the C–N bond cleavage leads to the free amine and the corresponding alcohol. Recently, an additional amide hydrogenation pathway was demonstrated, where the alkylated/benzylated amine is produced by a hydrogen borrowing/autotransfer mechanism from the initially formed alcohol and amine under specific acidic reaction conditions.^9^ Until today, the development of catalytic systems that enable these chemoselective transformations continues to be challenging and therefore are subject of ongoing research.

Initial efforts in this direction mainly focused on homogeneous ruthenium catalysts.^10^ Since the inspiring report by Cole-Hamilton and co-workers in 2012, various Ru-based systems for the highly selective scission of either the C–N or the C–O bond have been described.^10^

**Scheme 2:**
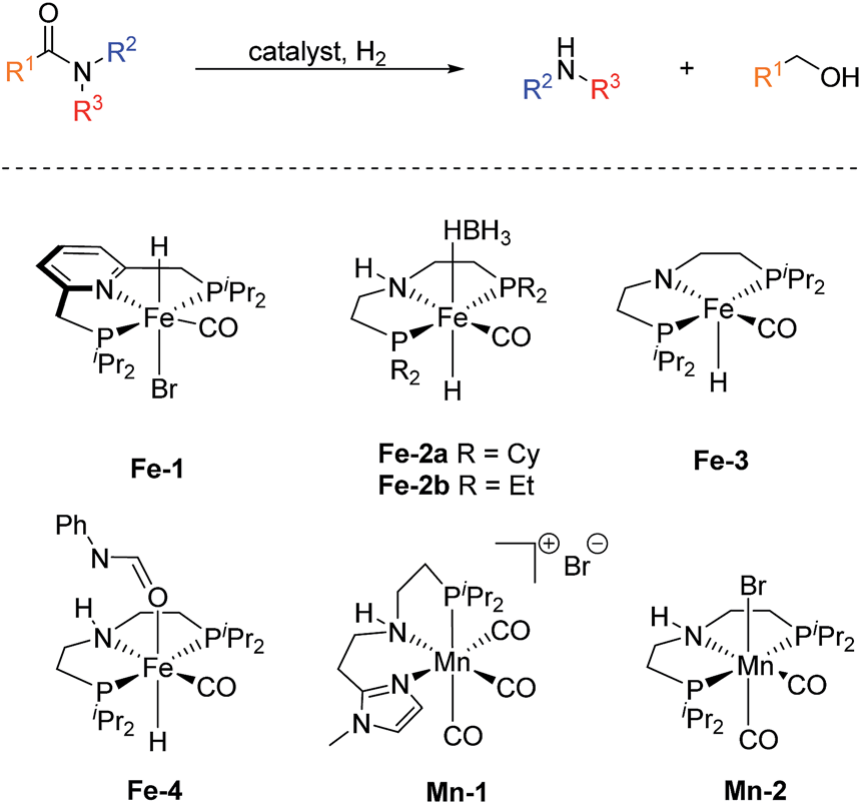
Base metal catalysts reported for the hydrogenolysis (C–N bond cleavage) of amides.

In sharp contrast, reports on homogeneous base metal catalysts for this important reaction are particularly scarce. Pioneering work in this area was published by the groups of Milstein, Langer and Sanford only as late as 2016.^11^–^13^ For the first time, they could demonstrate the ability of certain iron PNP pincer complexes (**Fe-1** as well as **Fe-2a**/**b**, Scheme 2) to promote the C–N bond cleavage in a number of different amides.

More specifically, Milstein and co-workers reported, that **Fe-1**, after activation with KHMDS, induced the hydrogenolysis of activated aliphatic and aromatic 2,2,2-trifluoroacetamides. However, no reaction was observed, with more common substrates such as *N*-phenylacetamide and *N*-phenylbenzamide.^11^ The protocols described by Sanford (**Fe-2a**) and Langer (**Fe-2b**) showed more general substrate scopes and obtained notable conversions and yields also for unactivated amides.^12^,^13^

Additionally, Bernskoetter and co-workers showed that the pentavalent iron PNP-pincer complex **Fe-3** is particularly active for the hydrogenolysis of a number of secondary formanilides and *N*-formylmorpholine (Scheme 2). The system stands out due to its extremely low catalyst loading (0.018–0.07 mol%) and notably operates under base-free conditions. Interestingly, the group of Bernskoetter demonstrated that an addition of 20 equivalents of formanilide resulted in a significantly improved activity of the system towards otherwise almost unreactive *N*-methylformanilide. Based on NMR experiments, the authors concluded that the catalyst adopts a different resting state in the presence of the additive (**Fe-4**, Scheme 2) and thus is less prone towards deactivating side reactions.^14^ The computational study of this reaction also suggested that the formanilide additive is involved in the C–N bond cleavage of the hemiaminal intermediate, which is the rate limiting step.^15^

Recently, our group reported the very first example of a manganese catalysed deaminative hydrogenation of amides under relatively mild conditions.^16^ After activation with exogenous base, the PNN pincer complex **Mn-1** (Scheme 2) exhibits remarkable activity for the hydrogenation of a broad scope of secondary and tertiary amides to the corresponding alcohols and amines. Notably, also more challenging primary amides were successfully cleaved in modest yields, tough more forcing conditions were shown to be necessary. The generality of the system was finally highlighted by the cleavage of the amide bond in the herbicide diflufenican. To date, **Mn-1** represents one of the most active and broadly applicable non-noble metal catalysts for amide hydrogenation. In a related study, Prakash and co-workers demonstrated that the manganese PNP pincer complex **Mn-2** is a suitable catalyst for the hydrogenation of formamides. The reaction proceeds *via* cleavage of the C–N bond to produce methanol and the corresponding amine.^17^

In 2018, we published the synthesis of a number of structurally related molybdenum PNP pincer complexes. Among the described complexes, **Mo-1a** (Table 1) was shown to be active in the catalytic hydrogenation of different acetophenones and styrenes.^18^ Similar Mo-systems have also been used for the hydrogenation of CO_2_, imines and nitriles.^19^ Based on these reports and our previous work, we became interested in the behaviour of such base-metal catalysts for the reductive cleavage of amides. Herein, we demonstrate its suitability for the hydrogenolysis of *N*-methylated formanilides under relatively mild conditions. To the best of our knowledge, PNP pincer supported molybdenum complexes have not been described for such transformations. Interestingly, the optimal catalyst exhibits a high selectivity for formamides. This preference has been rationalized by means of DFT calculations, which suggest that the produced MeOH reacts with the catalyst and changes the mechanism and rate limiting step of the reaction. This result, which is not observed in related Fe-catalysts, indicates that the catalyst design strategy should be adapted to the nature of the metal centre.

**Table 1:** Hydrogenation of *N*-methylformanilide **1a** to *N*-methylaniline **2a** and methanol **3** using Mo catalysts **Mo-1a–c** and **Mo-2**

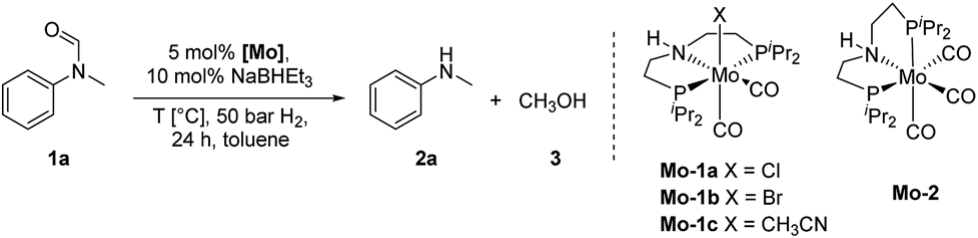				
Entry^*a*^ ^,^^*b*^	[Mo]	*T* [°C]	Conv^*c*^. [%]	**2a** ^*c*^ [%]
1	**Mo-1a**	130	>99	99
2	**Mo-1b**	130	>99	99
3	**Mo-1c**	130	>99	99
4	**Mo-2**	130	10	9
5^*d*^	—	130	10	8
6	**Mo-1a**	100	>99	98
7	**Mo-1b**	100	>99	99
8	**Mo-1c**	100	76	73
9	**Mo-1a**	80	89%	86%
10	**Mo-1b**	80	87%	84%
11^*e*^	**Mo-1a**	80	49	47
12^*e*^	**Mo-1b**	80	46	46

^*a*^Standard reaction conditions: *N*-methylformanilide **1a** (67.6 mg, 0.5 mmol), NaBHEt_3_ (50 μL, 0.05 mmol, 10 mol%), 2 mL toluene, 50 bar H_2_, 24 h.

^*b*^Yield of **3** was not determined.

^*c*^Conversion of **1a** and yield of **2a** were determined by GC using hexadecane as internal standard.

^*d*^No catalyst was used.

^*e*^Reaction was performed with 2.5 mol% of Mo catalyst.

## Results and discussion

### Catalytic hydrogenation of amides using molybdenum pincer complexes

At the outset of our study, we explored molybdenum-based PNP pincer complexes **Mo-1a–c** and **Mo-2** (Table 1), recently synthesised by our group, as potential catalysts for the hydrogenation of amides. Using *N*-methylformanilide **1a** as benchmark substrate, preliminary experiments were conducted using 5 mol% of Mo catalyst in toluene at 50 bar H_2_ and 130 °C, in the presence of 10 mol% of NaBHEt_3_. The reaction proceeded smoothly for complexes **Mo-1a–c** to afford *N*-methylaniline **2a** in quantitative yield along with methanol as the only by-product (Table 1, entries 1–3). However, complex **Mo-2** failed to display any catalytic activity (Table 1, entry 4). Next, the activity of the complexes was tested at reduced temperatures (Table 1, entries 6–10). It was found, that complexes **Mo-1a** as well as **Mo-1b** were equally efficient, when the reaction was conducted at 100 °C. Catalyst **Mo-1c**, however, gave a somewhat lower conversion and yield. Further reduction of the reaction temperature to 80 °C resulted once again in similar conversions and yields for **Mo-1a** and **Mo-1b**, respectively. Based on these observations, the catalyst loading was reduced to 2.5 mol% under otherwise identical reaction conditions (Table 1, entries 11 and 12). It turned out, that changing this parameter also led to almost identical outcomes for both catalytic systems. Therefore we concluded that, under reaction conditions, **Mo-1a** and **Mo-1b** very likely form the same active species. On the basis of the obtained results and due to the more challenging synthesis of **Mo-1b**, we decided to focus on catalyst **Mo-1a** in the due course of the study.

**Fig. 1:**
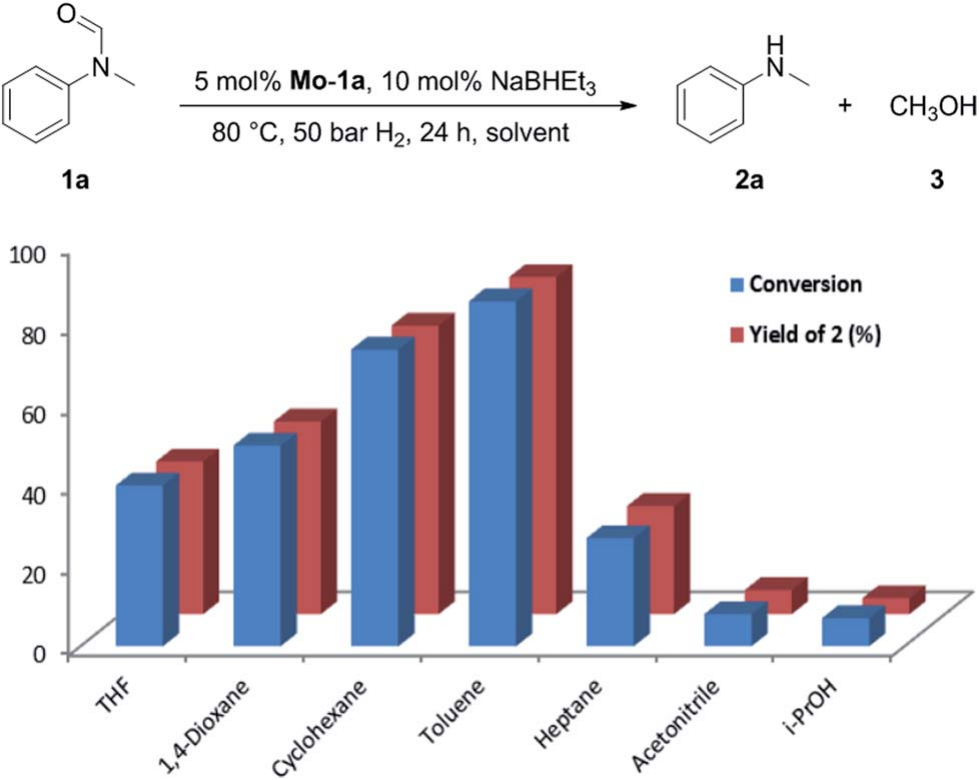
Study of the solvent effect in the hydrogenation of *N*-methylformanilide **1a** to *N*-methylaniline **2a** and methanol **3** catalysed by **Mo-1a**.

Selecting 80 °C reaction temperature and 5 mol% of **Mo-1a** (Table 1, entry 8) as the optimal setting for further optimization, we tested several different solvents. In contrast to previous work on manganese catalysed hydrogenolysis of amides, toluene was found to give the best results. Cyclohexane yielded slightly lower activities, while *n*-heptane as well as polar solvents, were shown to be significantly less suitable for the attempted transformation (Fig. 1).

Subsequently, we studied the influence of dihydrogen pressure, catalyst loading as well as the amount of additive used on the reaction outcome (Table 1, see ESI†). Lowering the pressure to 30 bar H_2_ resulted in a sharp drop in activity. However, no loss of reactivity was observed when the amount of NaBHEt_3_ was decreased to 5 mol%. A rise of the reaction temperature to 100 °C resulted in full conversion of the benchmark substrate to *N*-methylaniline in the presence of 5 mol% NaBHEt_3_ and **Mo-1a**, respectively. Further mitigation of the catalyst loading as well as the amount of NaBHEt_3_, however, had negative effects on the catalytic performance of the system.

Having optimised conditions in hand, we proceeded to the application of **Mo-1a** in the hydrogenation of a variety of different *N*-methylformanilides to the corresponding anilines and methanol (Table 2).

**Table 2:** Substrate scope in the hydrogenation of *N*-methylformanilides to *N*-methylanilines **2** and methanol **3** catalysed by **Mo-1a**

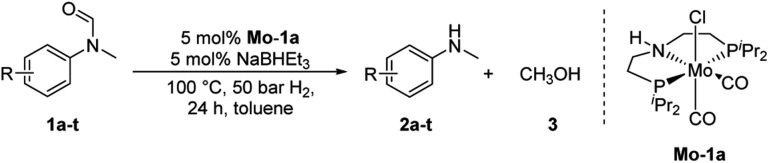			
Entry^*a*^ ^,^^*b*^	Formamide	Conv^*c*^. (%)	Yield^*d*^ of **2** (%)
1	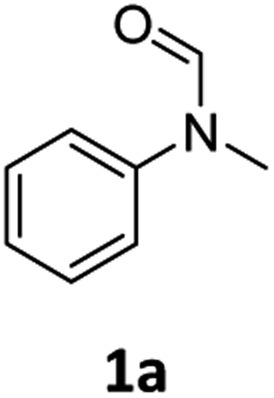	>99	94
2^*e*^	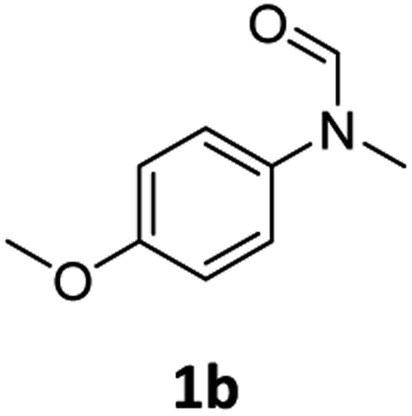	>99	96
3	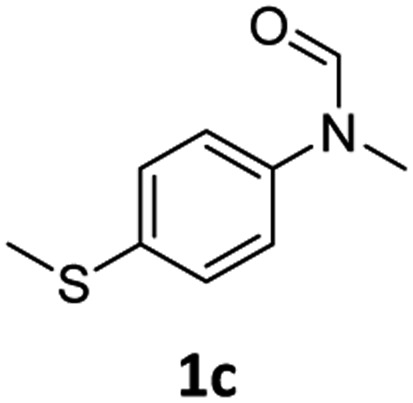	>99	95
4	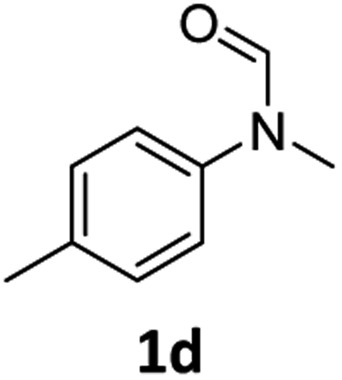	83	80
5	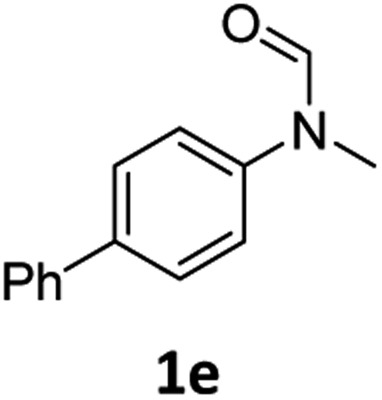	87	84
6^*e*^	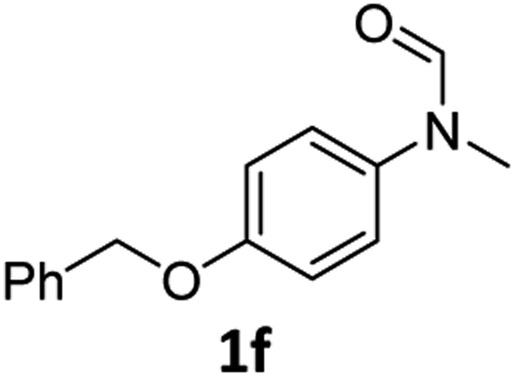	56	52
7^*e*^ ^*g*^	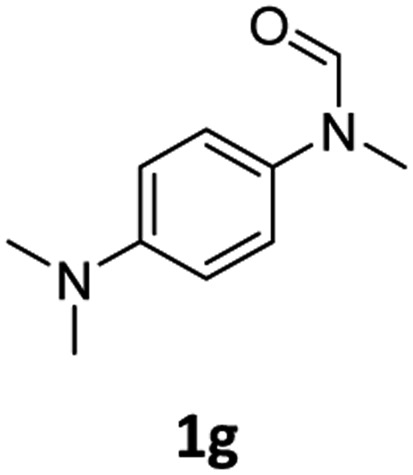	46	43
8	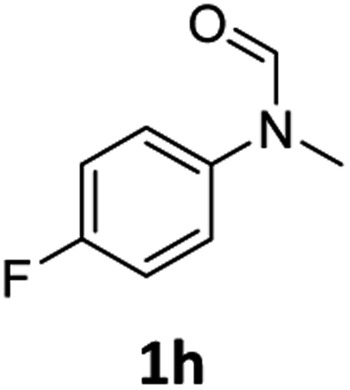	98	93
9	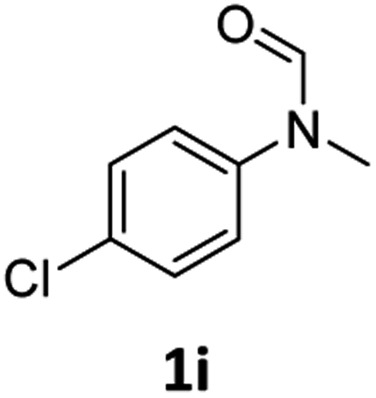	40	34^*f*^
10	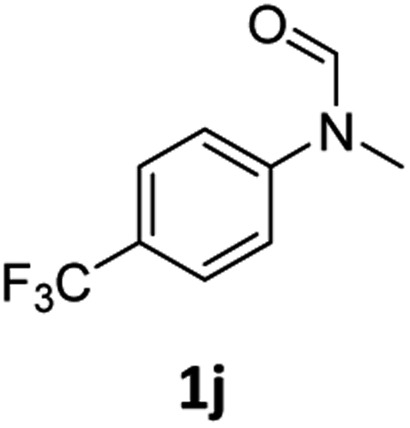	>99	>99
11^*e*^	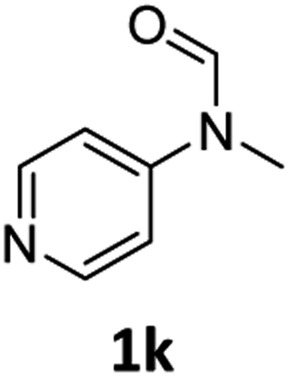	52	50
12	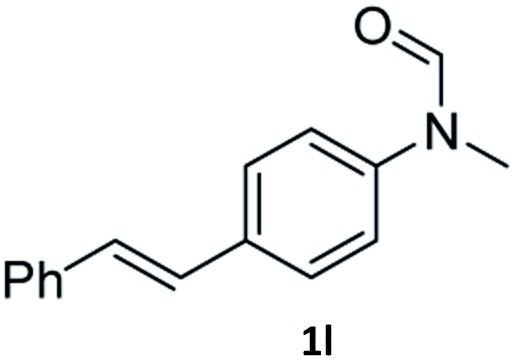	95	92
13	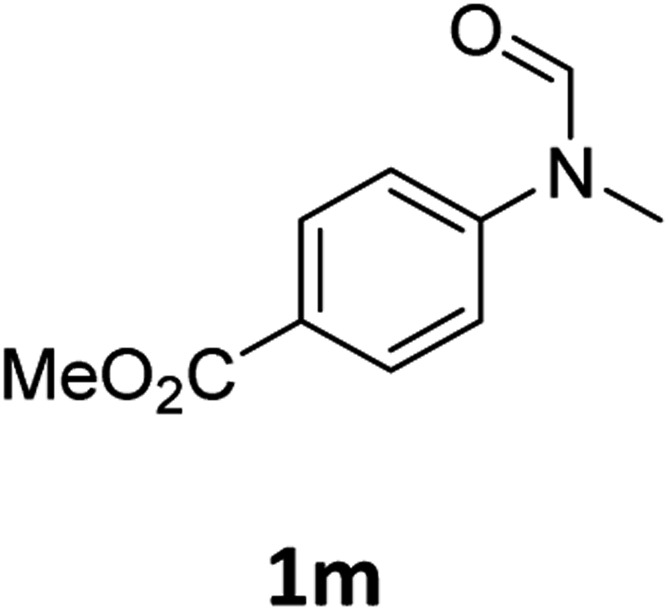	>99	97
14^*e*^	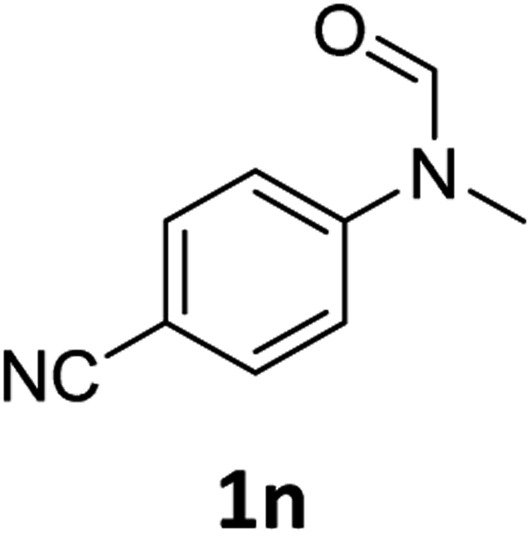	14	12^*f*^
15^*e*^	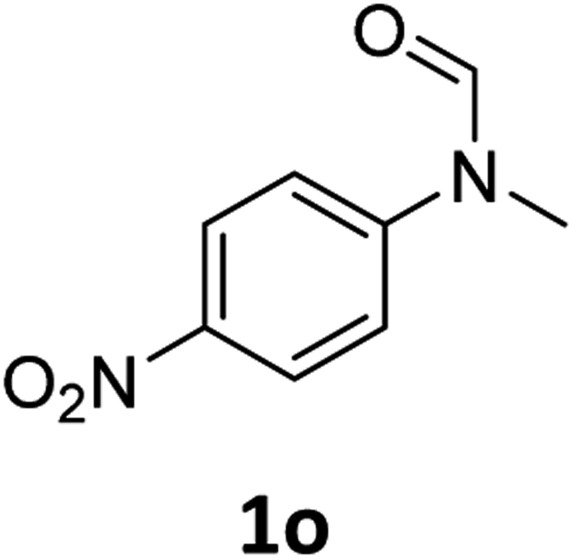	8	6^*f*^
16	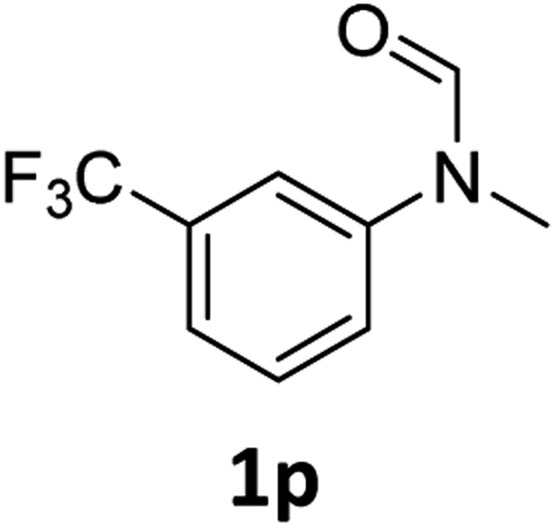	>99	97
17	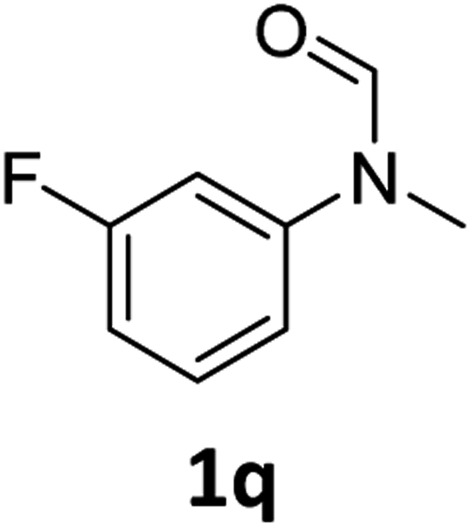	>99	98
18	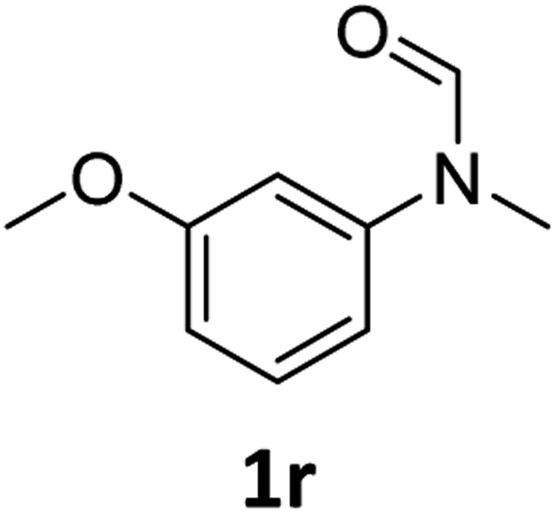	>99	93
19^*e*^	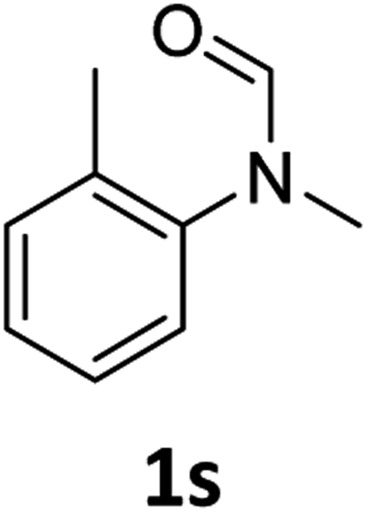	12	9^*f*^
20^*e*^	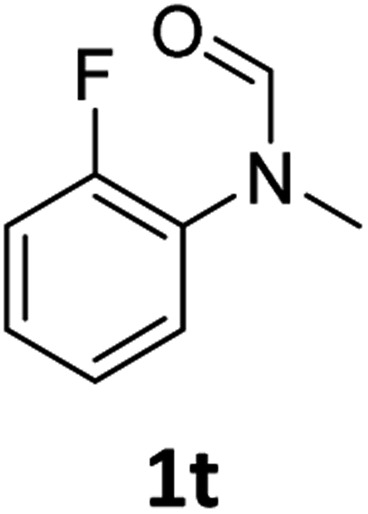	18	15^*f*^

^*a*^Standard reaction conditions: *N*-methylformanilide (0.5 mmol), **Mo-1a** (12.5 mg, 5 mol%), NaBHEt_3_ (50 μL, stock solution 0.5 M in THF, 5 mol%), 2 mL toluene, 50 bar H_2_, 24 h.

^*b*^Yield of **3** was not determined.

^*c*^Conversions of *N*-methylformanilides were determined by GC using hexadecane as internal standard.

^*d*^Isolated yields.

^*e*^Reaction was carried out at 130 °C.

^*f*^Yields were determined by GC using hexadecane as internal standard.

^*g*^Yield was determined based on the hydrochloride salt.

Most substrates were hydrogenated in good to excellent yields under optimised conditions at 100 °C and 50 bar H_2_ over 24 h, using toluene as solvent. In general, *meta*- and *para*-substitution were well tolerated, while substituents in *ortho*-position (Table 2, entries 19 and 20) appeared to be troublesome, probably due to steric hindrance. Amides containing electron donating groups were less reactive under standard conditions as compared to the benchmark substrate. In some cases higher reaction temperatures were required, in order to achieve good conversions (Table 2, entries 2, 6, 7). Notably, the thiomethyl substituted derivative (Table 2, entry 3) was fully hydrogenated and no catalyst poisoning effect was observed. Moreover, the system tolerated fluoro-substituents (Table 2, entries 8, 17, 20) and no dehalogenation products were detected. Interestingly, the system showed a good functional group tolerance towards substrates containing other reducible moieties such as benzyl ethers, C=C double bonds and esters (Table 2, entries 6, 12, 13). Noteworthy, no double bond isomerisation occurred during the reduction of a stilbene derivative (Table 2, entry 12). Additionally, pyridines, nitriles and nitro arenes remained unaffected under our reaction conditions; however, only poor to modest conversions were observed when the reaction was carried out at 130 °C (Table 2, entries 11, 14, 15). Presumably, this effect originates from substrate coordination to the metal centre and subsequent catalyst deactivation. The system turned out to be sensitive towards halides other than fluorine. Hence, during one of the hydrogenations, small amounts of the dehalogenation product were detected (Table 2, entry 9).

**Scheme 3:**
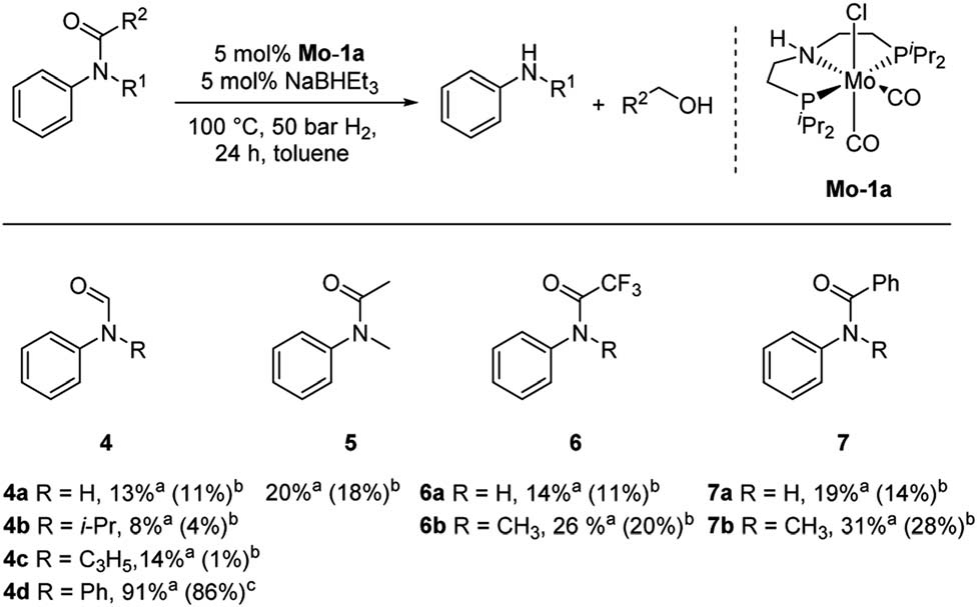
Hydrogenation of different amides (**4–7**) to the corresponding amines and alcohols catalysed by **Mo-1a**. ^*a*^Conversions of amides were determined by GC using hexadecane as internal standard. ^*b*^Yields were determined by GC using hexadecane as internal standard and refer to anilines, yields of alcohols were not determined. ^*c*^Isolated yields of anilines.

Subsequently, we investigated the more general applicability of our PNP pincer complex **Mo-1a** in the hydrogenation of other amides. Initial experiments focussed on the role of the nitrogen substitution on the reaction outcome. For this purpose, a series of different secondary and tertiary formanilides were subjected to our protocol (Scheme 3). The presence of an NH moiety turned out to be detrimental, as was observed for the parental formanilide (**4a**). This is in sharp contrast with the results obtained with Fe pincer complexes, in which formanilide derivatives give the highest conversion.^14^ In order to further validate this, 2,2,2-trifluoroacetanilide (**6a**) and simple benzanilide (**7a**) were employed and results comparable to formanilide (**4a**) were obtained. Likewise, only low conversions and yields were obtained in the case of *N*-^i^Pr- (**4b**) and *N*-allylformanilide (**4c**), respectively. Surprisingly, when *N*-allylformanilide was tested as substrate, the formation of *N*-allylaniline was only observed in traces. The main product was identified to be aniline, thus hinting at a deallylation pathway that additionally takes place to the envisaged hydrogenolysis. In contrast, *N*,*N*-diphenylformanilide (**4d**) was reduced smoothly and *N*,*N*-diphenylamine was isolated in excellent yield. Next, the hydrogenation of *N*-methylacetanilide (**5**) and the more activated 2,2,2-*N*-methyl-trifluoroacetanilide (**6b**), respectively, were attempted. In either case, only poor conversions were determined demonstrating the high preference of this complex for specific formanilides. This was further supported by the low reactivity of *N*-methylbenzanilide (**7b**) and some aliphatic formamides (see Table 2, ESI†).

**Scheme 4:**
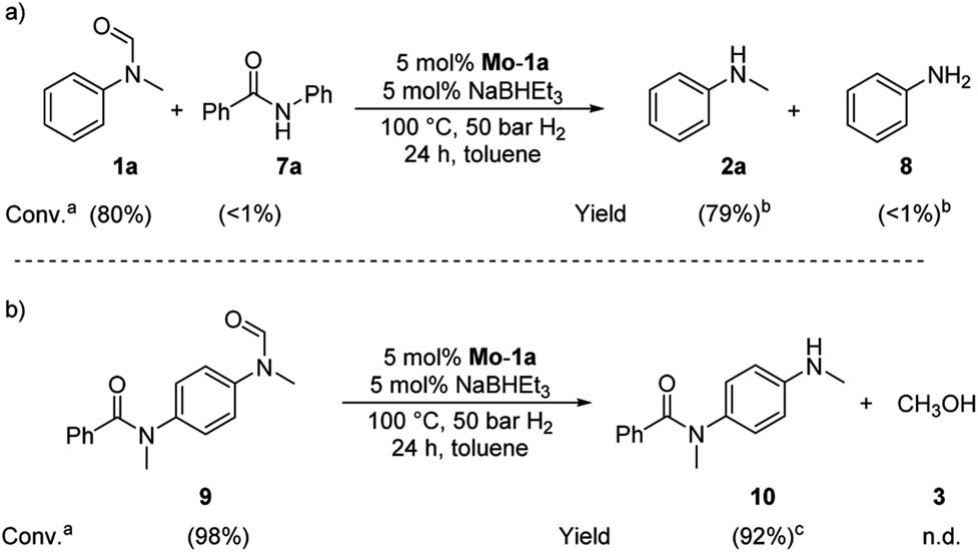
Selective hydrogenations of (a) *N*-methylformanilide **1a** in the presence of benzamide **7a** and (b) *N*-methyl-*N*-(4-(*N*-methylformamide)phenyl)benzamide **9**. Standard conditions: substrate(s) 0.5 mmol (each), **Mo-1a** (12.5 mg, 0.025 mmol, 5 mol%), NaBHEt_3_ (50 μL, 0.5 M stock solution in THF, 0.025 mmol, 5 mol%), toluene (2 mL), 50 bar H_2_, 100 °C, 24 h. ^*a*^Conversions determined by GC using hexadecane as internal standard. ^*b*^Yields determined by GC using hexadecane as internal standard. ^*c*^Isolated yield.

Based on these observations, we were curious to demonstrate selective formamide reduction in the presence of other amide moieties. In a proof of concept experiment, the hydrogenation of the benchmark amide in the presence of benzamide **7a** was conducted (Scheme 4, eqn (a)). It could be shown that **Mo-1a** was capable to cleave *N*-methylformanilide (**1a**) with extremely high preference. Notably, the reaction still proceeded with 80% conversion with respect to *N*-methylformanilide (**1a**). To further highlight the scope of our system, we designed model substrate **9** combining two amide functionalities in one structure. After 24 h reaction, the intended hydrogenolysis of the formamide moiety in **9** had occurred smoothly and the target molecule **10** was isolated in a very high yield (92%). Notably, no cleavage of the benzamide was observed.

We believe these results could pave the way towards new and selective deprotection strategies in organic synthesis mediated by this base metal PNP pincer complex.

### Reaction mechanism

**Scheme 5:**
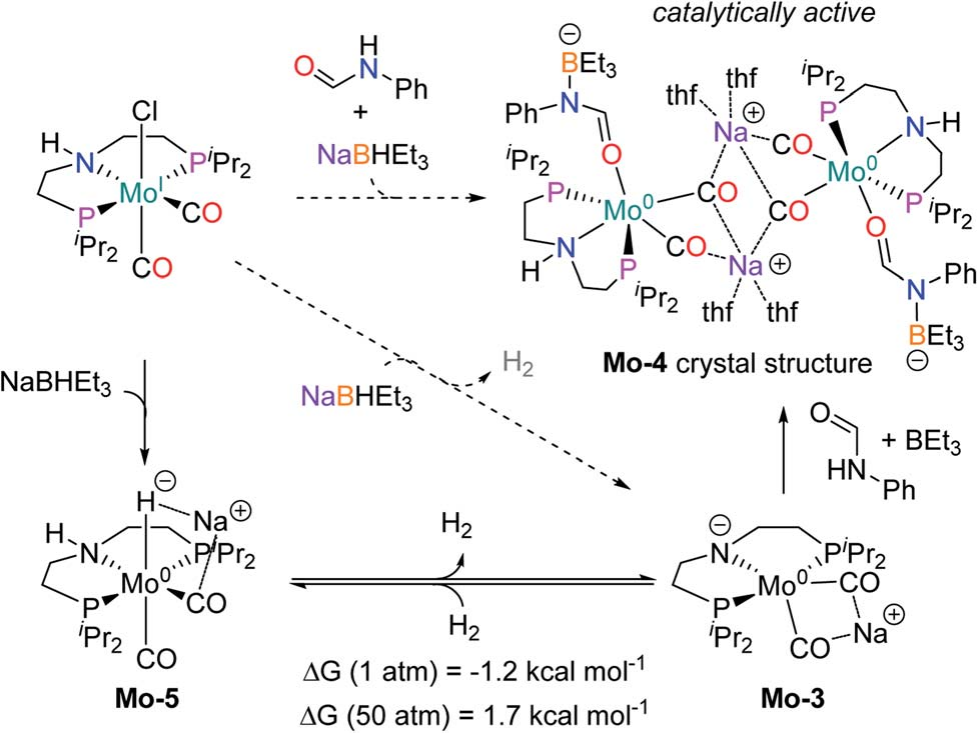
Reactions performed to get insight on the active catalytic species (in dashed arrows) with the experimental observed products (H_2_ and the crystal structure of **Mo-4**, in color) and the intermediates proposed (**Mo-3** and **Mo-5**). Gibbs energies calculated for the de-hydrogenation of **Mo-5** at different pressure.

**Fig. 2:**
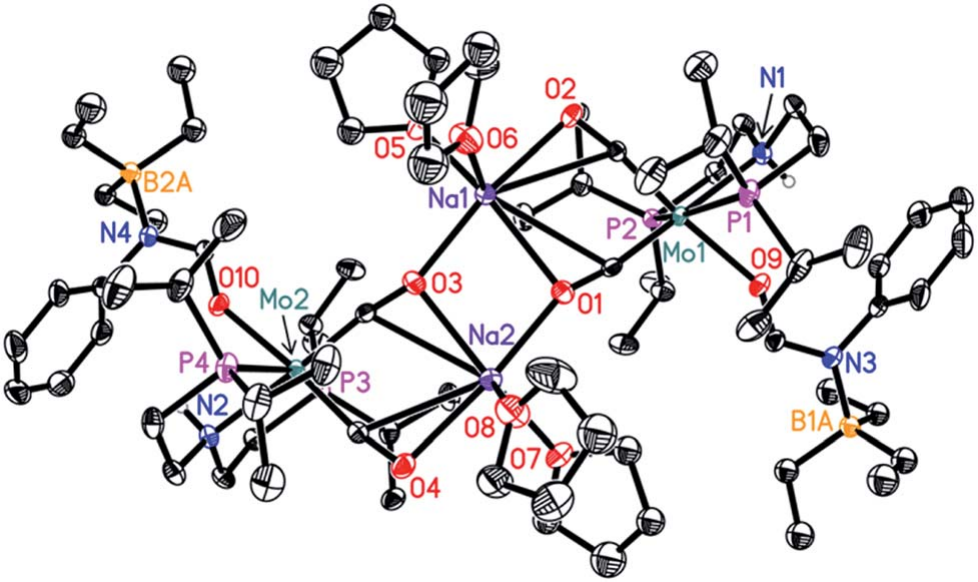
Molecular structure of **Mo-4** in the crystal (see Scheme 5 for a graphical representation). Displacement ellipsoids correspond to 30% probability. Hydrogen atoms except the N-bound are omitted for clarity.

In order to understand the general reactivity of **Mo-1a** and its performance with different amides, DFT calculations and supporting experiments were conducted. Scheme 5 shows the experiments performed to determine the active catalyst species. Treatment of **Mo-1a** with NaBHEt_3_ resulted in rapid hydrogen evolution. The nature of the gas was determined in a scale up experiment (100 μmol of **Mo-1a**) using GC-analysis. This observation prompted us to assume that the obtained reaction product was likely to be a pincer amido species such as **Mo-3**, in which Mo(i) has been reduced to Mo(0). This conclusion was further supported by HR-ESI mass spectrometry of the corresponding reaction mixture. When the distinct reactivity of the catalyst towards formanilide was studied, we isolated **Mo-4** in form of colorless needles from the reaction mixture (Fig. 2; for detailed experimental procedure see ESI†).

Notably, the crystal structure of **Mo-4** (Fig. 2 and Scheme 5) features two anionic Mo(0) complexes neutralized by two Na^+^ cations interacting with the CO ligands. In order to investigate, whether **Mo-4** is involved in the catalytic cycle, the reduction of *N*-methylformanilide was carried out using 2.5 mol% of **Mo-4** under conditions optimized for **Mo-1a**. In fact, we observed full conversion of the substrate and isolated *N*-methylaniline in 92% yield. Thus, we conclude, that the catalytically active species contains a Mo(0) center. This is also consistent with the EPR-silent nature of the product formed in the activation of **Mo-1a** by NaBHEt_3_.

The observed activity of **Mo-4** suggests that the Mo(0)-complexes **Mo-3** and **Mo-5**, shown in Scheme 5, are presumably the main catalytic intermediates. Similar species have been proposed for the isoelectronic Fe(ii)-complexes **Fe-2**, **Fe-3** and the Mn(i)-complex **Mn-2** (Scheme 2).^20^

Based on these results, DFT calculations, with the M06 functional, including toluene solvation with the SMD model, were used to get further insights into the reaction mechanism (see computational details and ESI for details†). The hydrogenation of **Mo-3** to yield **Mo-5**, was found to be almost isoenergetic, with a small preference for **Mo-3** at 1 bar and **Mo-5** at 50 bar (Scheme 5). These energies agree with the bubbling of H_2_ observed experimentally during the catalyst activation reaction.

**Fig. 3:**
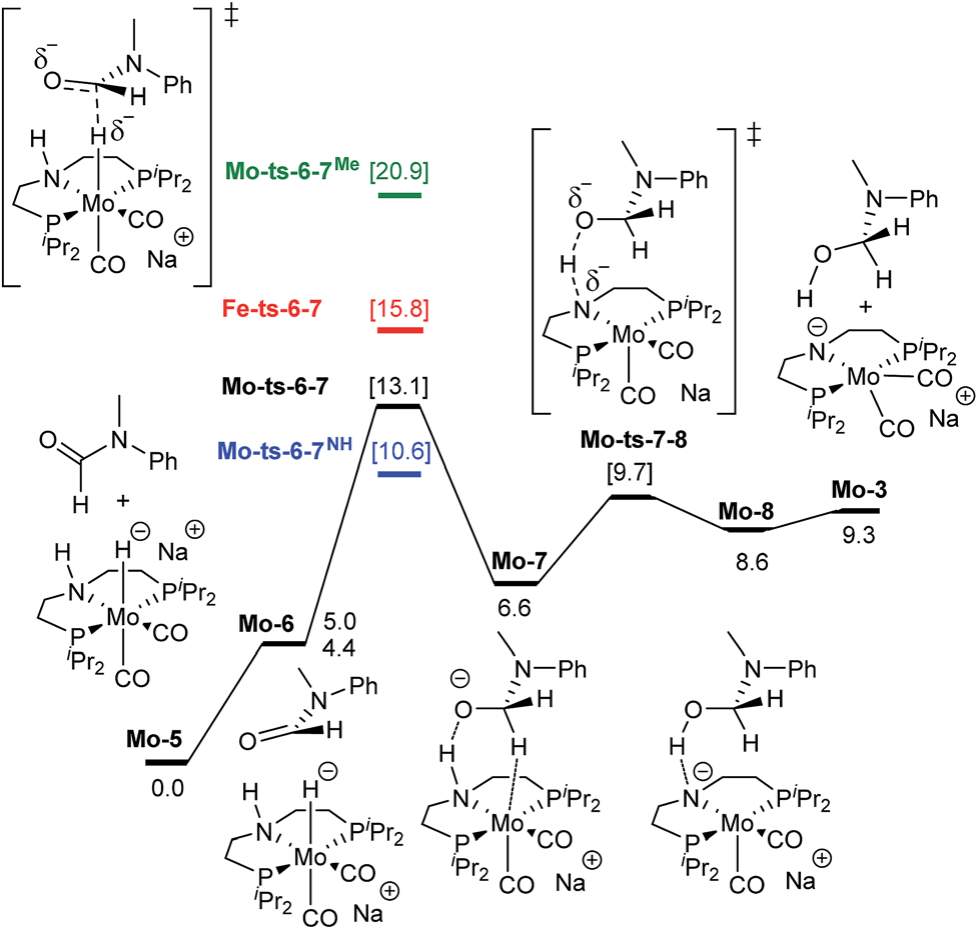
Reaction pathway for the hemiaminal formation from the *N*-methyl formanilide with **Mo-5**. Gibbs energies in toluene (SMD) at 50 atm and 373 K are given in kcal mol^–1^. In blue and green, energies for the hydride transfer using formanilide and *N*-methylacetanilide, respectively. In red, energy for the hydride transfer using the reported **Fe-3** complex at 30 atm (Scheme 2).^15^

As represented in Scheme 1, amide hydrogenolysis is proposed to consist in three steps: amide C=O reduction, C–N bond protonolysis of the formed hemiaminal, and aldehyde C=O reduction. These steps were computed for *N*-methylformanilide and the energy profiles for the preferred pathways are given in Fig. 3 and 5, and the ESI.†

The mechanism for the amide C=O hydrogenation by **Mo-5** consists of the hydride transfer from Mo to the amide carbonyl group (**Mo-ts-6-7**), followed by proton transfer from the ligand nitrogen to the amide oxygen (**Mo-ts-7-8**). This pathway was computed for formanilide (**Mo-ts-6-7^NH^** in Fig. 3) and *N*-methylformanilide. With both substrates, the hydride transfer has the highest energy barrier (10.6 kcal mol^–1^ with formanilide and 13.1 kcal mol^–1^ with *N*-methylformanilide). Interestingly, these energies are lower than those reported by us for the analogous Fe catalyst with formanilide (15.8 kcal mol^–1^, **Fe-ts-6-7** in Fig. 3).^15^

**Fig. 4:**
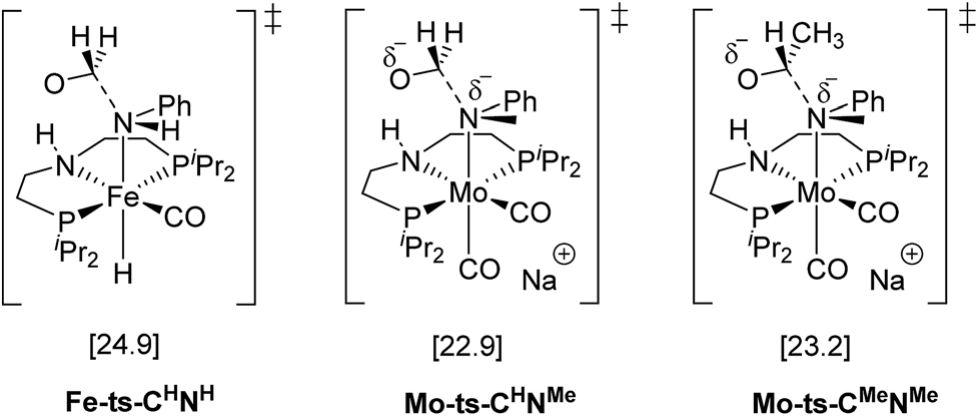
TSs for the C–N bond cleavage step *via* the mechanism previously reported for **Fe-3**.^15^

The mechanism for the C–N bond cleavage from the formed hemiaminal (Scheme 1) was also investigated. In the case of **Fe-3**, this step was reported to proceed *via* the transition state **Fe-ts-C^H^–N^H^** (Fig. 4).^15^ With Mo and *N*-methylformanilide, the same pathway involves a Gibbs energy barrier of 22.9 kcal mol^–1^ (**Mo-ts-C^H^–N^Me^**). An increase of less than 1 kcal mol^–1^ is observed by changing the substrate to *N*-methylacetanilide (**Mo-ts-C^Me^–N^Me^**).

The similar energy barriers obtained with these substrates did not account for the large differences in yield observed experimentally (99% Conv. in *N*-methylformanilide *vs.* 20% Conv. in *N*-methylacetanilide). In addition, the lower energy barriers obtained with Mo compared to Fe are inconsistent with the higher H_2_ pressure and time required to accomplish amide hydrogenation with **Mo-1a** compared to **Fe-3**.^14^

**Fig. 5:**
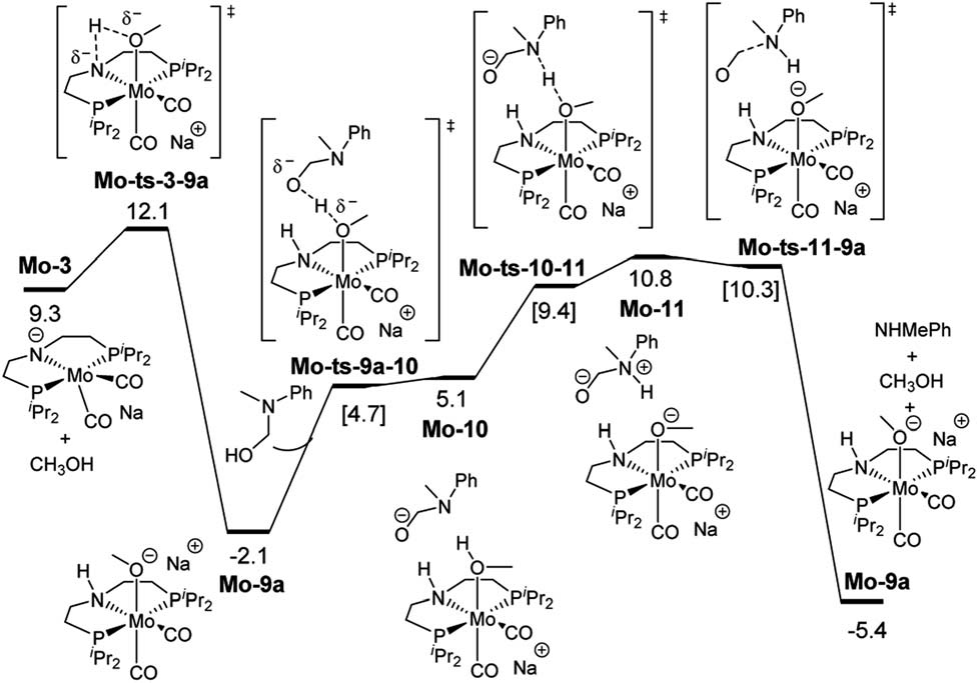
Reaction pathway of the MeOH assisted hemiaminal proton transfer and posterior C–N bond cleavage. Gibbs energies in toluene (SMD) at 50 atm and 373 K are given in kcal mol^–1^.

These discrepancies were explained by considering the reaction of **Mo-3** with methanol leading to the Mo-methoxy intermediate **Mo-9a** (Fig. 5). This reaction, which involves the deprotonation of MeOH by the amido ligand (**Mo-ts-3-9a**), has a low energy barrier (Δ*G*^‡^ = 2.8 kcal mol^–1^) and is highly exergonic (Δ*G* = –11.4 kcal mol^–1^). The formation of related M-methoxy species have been observed for similar Fe, Ru, Os and Mn PNP-pincer complexes.20c,^21^,^22^ This species can promote the protonolysis of the C–N bond by assisting the OH-deprotonation and *N*-protonation of the hemiaminal intermediate (**Mo-ts-11-9a**). The highest energy of this process is 10.8 kcal mol^–1^, which corresponds to the zwitterion hemiaminal intermediate interacting with the methoxide–Mo complex (**Mo-11**). This energy is lower than the energy barrier for the hydride transfer (13.1 kcal mol^–1^), indicating that the C–N bond cleavage is not the rate limiting step once MeOH is formed (note: for a comparison of this mechanism with Mo and Fe-systems see ESI†).

**Scheme 6:**
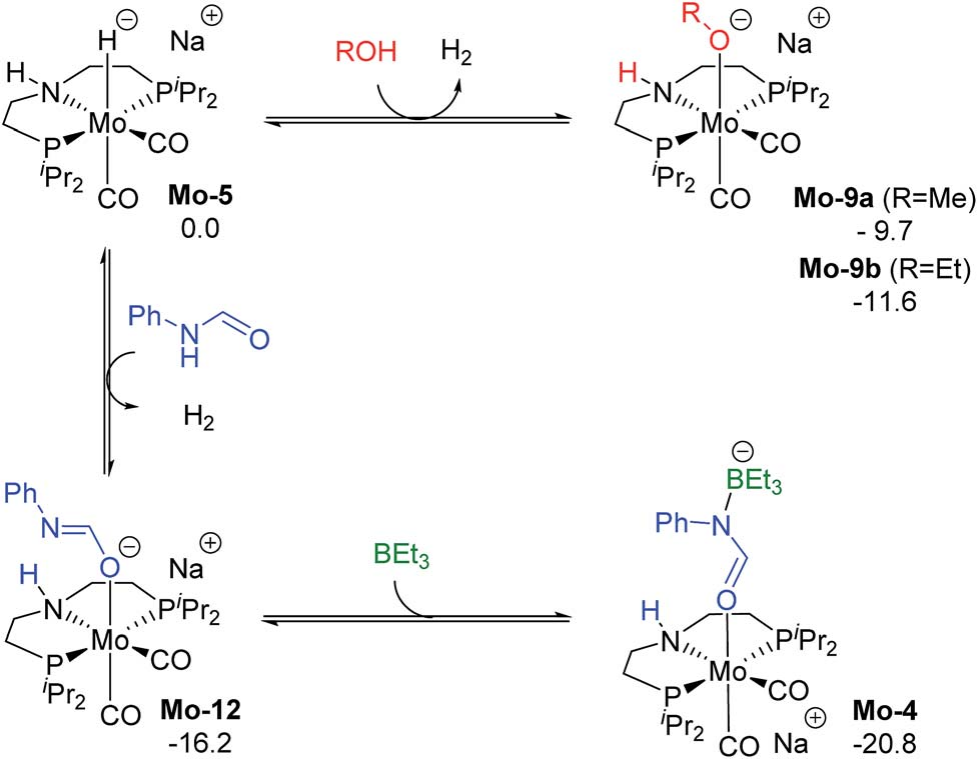
Calculated Gibbs energies (kcal mol^–1^) for the substitution of H_2_ in **Mo-5** by methanol, ethanol, formanilide and BEt_3_ yielding **Mo-9a**, **b**, **Mo-10** and **Mo-4**, respectively.

The reaction of **Mo-5** with MeOH yields hydrogen and is exergonic (Δ*G* = –9.7 kcal mol^–1^, Scheme 6). The methoxy intermediate **Mo-9a** is thus the resting state of the catalyst.

Formanilide, and other secondary amides, can also displace H_2_ from the catalyst (**Mo-12** in Scheme 6). This reaction is even more exergonic (Δ*G* = –16.2 kcal mol^–1^) than with MeOH increasing the global energy barrier for the hydride transfer from 10.6 to 26.8 kcal mol^–1^ with formanilide. This energy may increase to 31.4 kcal mol^–1^ by reaction with BEt_3_ (**Mo-4**). In contrast, with *N*-methylformanilide, the only penalty to pay is the addition of MeOH. Therefore, the energy barrier for the hydride transfer increases from 13.1 to 22.9 kcal mol^–1^, which is lower than the barrier for formanilide, consistent with the larger conversion obtained with *N*-methylformanilide. In the case of *N*-methylacetanilide, the addition of ethanol instead of methanol is expected. The higher stability of the ethoxide complex **Mo-9b** compared to **Mo-9a** by *ca.* 2 kcal mol^–1^ (Scheme 6), together with the higher energy barrier for the hydride transfer with this substrate (Δ*G* = 20.9 kcal mol^–1^, Fig. 3), is consistent with the low yields obtained experimentally with *N*-methylacetanilide.

The mechanism of catalyst recovery by addition of H_2_ to the methoxide complex **Mo-9a** is shown in Fig. S3.† In this pathway, methanol assists the activation of the Mo–H_2_ complex (**Mo-14**) by acting as a proton-shuttle with a global energy barrier of 23.0 kcal mol^–1^. Similar mechanisms have been proposed with Ru–N and Fe–N complexes (see ESI†).21b,^23^

**Fig. 6:**
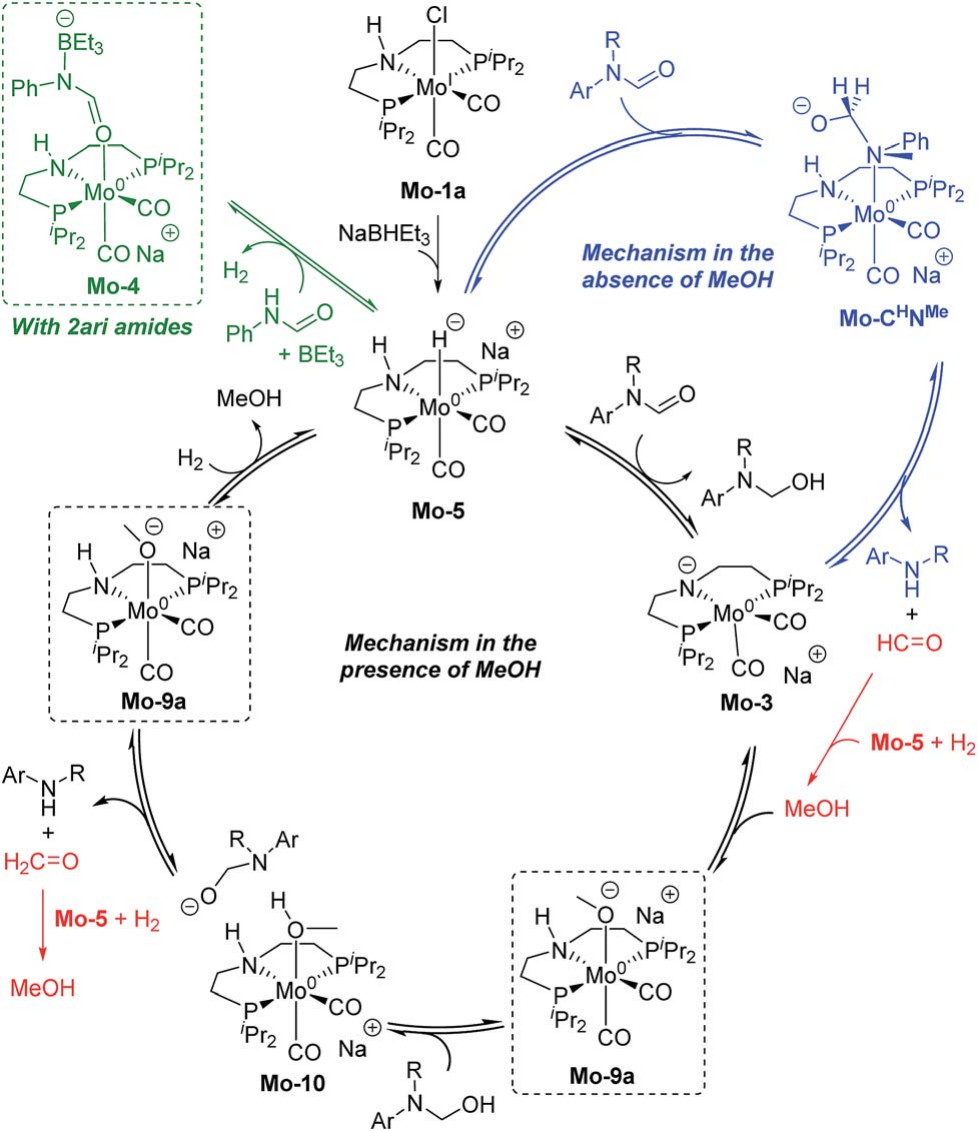
General mechanism for the amide hydrogenation in the absence (in blue) and presence (in black) of methanol with the formaldehyde hydrogenation in red. Dashed squares indicate the catalyst resting state in the presence of MeOH and 2ari amides (in green).

The results from the computational study can be summarized in the catalytic cycle represented in Fig. 6. In the absence of alcohol, the Mo-catalyst is involved in the hemiaminal C–N bond cleavage after the amide C=O reduction (blue cycle). This reaction yields amine and formaldehyde, which is reduced to alcohol by the catalyst **Mo-5** in a subsequent reaction (in red). In the presence of alcohol, a Mo-alkoxo intermediate is formed, **Mo-9a**. This species, which becomes the catalyst resting state, is involved in the hemiaminal C–N bond cleavage. Finally, the catalyst recovery takes place by the displacement of alcohol by H_2_. The nature of the catalyst resting state may change with secondary amides, which reacts with the catalyst forming an adduct (**Mo-4**, in green) that hampers the reaction.

In order to validate this mechanism and the nature of Mo(0) active species, the role of the counter-cation in this reaction was explored computational and experimentally by using LiHBEt_3_, NaHBEt_3_, and KHBEt_3_. Carrying out the benchmark reaction at 80 °C, 5 mol% of the alkali metal hydrides were added to activate **Mo-1a**. It could be shown, that for NaBHEt_3_ and KBHEt_3_ similar conversions of *N*-methylformanilide (**1a**) (76% and 77%, respectively) and yields of **2a** (75% and 73%, respectively) were obtained. However, when LiBHEt_3_ was used, only 10% conversion of **1a** and 9% yield of *N*-methylaniline **2a** was obtained. These results were in agreement with the trends on the energy barriers obtained for the amide C=O reduction step, which are 22.9, 23.0 and 28.8 kcal mol^–1^ with Na^+^, K^+^ and Li^+^, respectively, taking **Mo-9a** as energy reference. The stronger electrostatic interaction of Li^+^ with the methoxide intermediate (**Mo-9a^Li^**), accounts for the highest energy barrier predicted for this system (see ESI†).

**Fig. 7:**
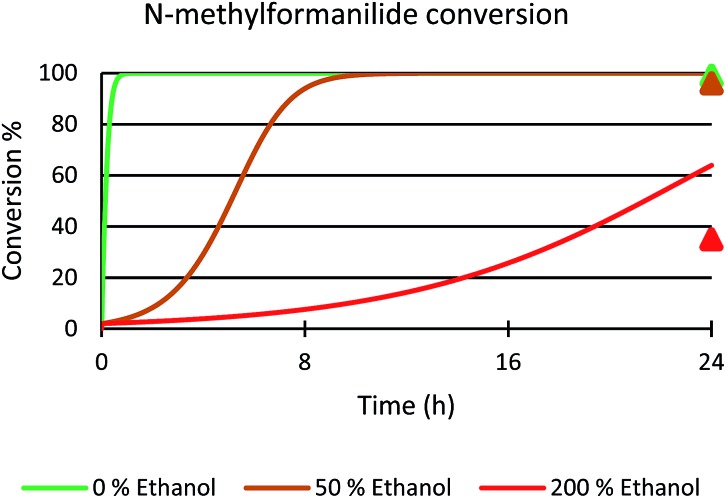
Microkinetic simulation of *N*-methylformanilide **1a** conversion with 0% (green), 50% (brown) and 200% (red) ethanol in solution. The initial concentration of reactants were the same as those used in the experiments; *i.e.* 0.25 M *N*-methylformanilide **1a**, 0.207 M of dihydrogen and 12.5 mM of **Mo-5**. Experimental values at 24 hours represented with triangles.

Next, the role of the alcohol was explored by adding different amounts of ethanol to the benchmark system. In the presence of 50 mol% of EtOH, 96% conversion of *N*-methylformanilide (**1a**) and 93% product yield were obtained. However, the addition of 200 mol% resulted in a sharp decrease in conversion and yield (35% conversion, 32% yield). Thus, it was concluded that ethanol has a detrimental effect on the performance of the catalytic system. Notably, these trends were reproduced with a microkinetic model based on the general mechanism represented in Fig. 6 (in Fig. 7). This model predicted 100% conversion after 24 h of reaction for both 0% and 50% concentrations of ethanol. In contrast, and in line with the experiments, the same model predicted a significant decrease of conversion to 64% with an ethanol concentration of 200% (see ESI for further details†).

## Conclusions

Well-defined molybdenum–PNP pincer complexes have been used for the first time in the hydrogenation of a range of amides to the corresponding alcohols and amines. *N*-Alkylated and *N*-arylated formamides can be hydrogenated to the corresponding products in good to high yields. Applying complex **Mo-1a** high selectivity for the hydrogenation of formamides was observed in the presence of other reducible groups. These results pave the way for potential applications of this type of complexes in synthetic methodologies.

The DFT study shows that the active Mo(0) species (**Mo-5**) reduces the C=O group of the amide through low-energy barriers, compared to Fe-based systems. However, the alcohol product and secondary amides react with the catalyst forming stable adducts encumbering catalyst recovery and increasing the overall barrier for the reduction of the C=O group. These results suggest that further catalyst design should focus on preventing the formation of these adducts, while keeping the high hydricity of the complex.

## Experimental details

### General experimental information

All hydrogenation reactions were set up under Ar in a 300 mL autoclave (PARR Instrument Company). In order to avoid unspecific reductions, all catalytic experiments were carried out in 4 mL glass vials, which were set up in an alloy plate and placed inside the autoclave.

In a glove box, a 4 mL glass vial containing a stirring bar was charged with complex **Mo-1a** (12.5 mg; 5 mol%). Toluene (2 mL) was added and the corresponding brown suspension was treated with NaBHEt_3_ (0.5 M in THF; 50 μL; 10 mol%). The reaction mixture was stirred for 10 minutes and the corresponding substrate was subsequently added. Afterwards, the vial was capped and transferred into an autoclave. Once sealed, the autoclave was purged three times with 10 bar of hydrogen, then pressurized to the desired hydrogen pressure (50 bar), and placed into an aluminum block that was preheated to the desired temperature (100 °C). After 24 h, the autoclave was cooled in an ice bath and the remaining gas was released carefully. The solution was subsequently diluted with ethyl acetate and filtered through a small pad of Celite (1 cm in a Pasteur pipette). The Celite was washed with methanol (2 mL) and the combined filtrates were subsequently evaporated to dryness. The remaining residue was purified by column chromatography (SiO_2_, heptane/EtOAc, gradient 100 : 0 → 0 : 100). In the case of substrate **7**, the purified product was dissolved in 5 mL of Et_2_O and subsequently treated with 1 mL of HCl (2 M in Et_2_O). The reddish precipitate was filtered off, washed three times with 5 mL of Et_2_O and finally dried *in vacuo*. For the characterization of the products of the catalysis, see ESI.†

### Computational details

DFT calculations were carried out with Gaussian 09 24 with the M06 25 functional and the double-z LANL2DZ (on Mo, including relativistic effects)^26^ and 6-31+G** (on all other elements)^27^ basis sets. Calculations were done using the full system. The location of the Na^+^ cation was evaluated in some of the intermediates, and the preferred position is represented in figures and schemes of the manuscript (see ESI†). The geometry optimization and energies of the possible spin states of **Mo-1a** and **Mo-4** were consistent with a doublet and singlet ground state, respectively (see ESI†). Vibrational frequencies were computed at the same level of theory to obtain the thermochemistry corrections (zero-point, thermal and entropy energies) at the experimental *p* = 50 atm and *T* = 373.15 K. The energy of the optimized geometries was refined by single point calculations with triple-z quality basis sets, including the LANL2TZ^26^ on Mo and the 6-311+G** on all other elements.^28^ The energies reported in the text were obtained by adding the thermochemistry corrections to the refined potential energies. The solvation effects of toluene were included in both the geometry optimizations and energy refinements using the continuum SMD model.^29^ The ultrafine (99 590) grid was used in all calculations for higher numerical accuracy. A repository containing all input and output files is available on-line from ioChem BD at ; https://iochem-bd.bsc.es/browse/handle/100/193698.^30^ Microkinetic models were simulated with the COPASI software^31^ using the LSODA algorithm. See ESI for further details.†

## Conflicts of interest

There are no conflicts to declare.

## Supplementary Material

Supplementary information
