# Supplementary material for: Highly selective hydrogenation of amides catalysed by a molybdenum pincer complex: scope and mechanism
Source: Chem Sci. 2019 Oct 8;10(45):10566–76. doi: 10.1039/c9sc03453f (PMC7020655; doi:10.1039/c9sc03453f)
Supplement: Supplementary file 1 [file SC-010-C9SC03453F-s001.pdf]

Electronic Supplementary Material (ESI) for Chemical Science.
This journal is © The Royal Society of Chemistry 2019

Electronic Supplementary Information

Highly Selective Hydrogenation of Amides Catalysed by a Molybdenum Pincer Complex: Scope and Mechanism

Thomas Leischner[a], Lluis Artus Suarez[b], Anke Spannenberg[a], Kathrin Junge[a], Ainara Nova[b], Matthias Beller [a]

Experimental Details

General Experimental information S2

Synthesis of Mo-4 S3

X-ray crystal structure analysis of Mo-4 S4

Table 1. Further Optimisation of the General Catalytic Procedure S5

Table 2. Screening of Aliphatic Formamides S6

General Procedure for the Synthesis of N-Methylated Anilines S7

Synthesis of N-methyl-N-(4-(methylamino)phenyl)benzamide S11

General Procedure for the Synthesis of N-Methylated Formanilides S12

IR spectra of the previously unknown N-methylformanilides and N-methylanilines S22

NMR-spectra of the isolated products of the catalysis S31

Computational Details

DFT functional benchmark S87

Comparison Iron system vs Molybdenum system S89

Catalyst recovery mechanism S91

Hydrogenation of formaldehyde S92

Mo-9a and Mo-ts-6-7 with Li^+, Na^+, K^+ S93

Microkinetic models S95

Evaluation of the position of the Na cation S98

Cartesian coordinates and corresponding corrected Gibbs energies S100

Literature S207

Experimental Details

General Experimental Information.

Dry and oxygen-free solvents (acetonitrile, DCM, toluene, THF, methanol, ethanol and heptane) were collected from an Innovative Technology PS-MD-6 solvent purification system and stored over 3 Å molecular sieves. D_8-THF and CD_2Cl_2 were purchased from euriso-top, degassed by freeze-pump-thaw techniques and subsequently dried over 3 Å molecular sieves. All other chemicals were purchased and used without further purification. D_6-DMSO and $CDCl_3$ were obtained from euriso-top. Aniline, benzamide, trifluoroaceticacid anhydride, $Mo(CO)_6$, benzoylchloride, $LiBHEt_3$ (1M in THF), $NaBHEt_3$ (1M in THF), $KBHEt_3$ (1M in THF), 2,2,2-trifluoroacetanilide, 3-methoxy-*N*-methylaniline, tetraphenylphosphonium chloride, 2-fluoro-*N*-methylaniline, 3-fluoro-*N*-methylaniline, 4-fluoro-*N*-methylaniline, 4-chloro-*N*-methylaniline, 4-methylaminobenzonitrile, *N*-methyl-4-(trifluoromethyl)aniline, 4-(Benzoyloxy)aniline hydrochloride, *N*,*N*-dimtehylamino-*p*-phenylenediamine and *N*-methyl-ε-caprolactam were obtained from Sigma Aldrich. $(^iPr_2PCH_2CH_2)_2NH$ was obtained from Strem (10 wt-% solution in THF). Methyl-4-(methylamino)benzoate was purchased from Alfa Aesar. Formic acid, acetic anhydride, formanilide, *N*-methylformanilide, *N*-methylacetanilide, *N*-methylaniline, *N*-methyl-*p*-anisidine, 4-methylthioaniline, 4-methylaminopyridine, *N*-allylaniline and *N*-isopropylaniline were purchased from TCI. Sodium methoxide, tetraphenylphosphonium tetraphenylborate, *N*-n-octylformamide, *N*-formylmorpholine *N*-cyclohexylformamide *N*-methyl-*p*-toluidine, 1,4-phenylenediamine, 4-chloro-*N*-methylformanilide 4-aminostilbene (*trans*-isomer), 4-aminobiphenyl, *N*-methyl-3-(trifluoromethyl)aniline, *N*,*N*-diphenylformanilide, 2-methyl-*N*-methylformanilide were obtained from ABCR. Complexes Mo-1a-d were synthesized as previously reported.[1] *N*-methyl-*N*-phenylbenzamide[2], 2,2,2-trifluoro-*N*-methyl-*N*-phenylacetamide[3], *N*-isopropyl-*N*-phenylformamide[4] and *N*-allyl-*N*-phenylformamide[4] were synthesized according to literature procedures.

Synthesis of Mo-4:

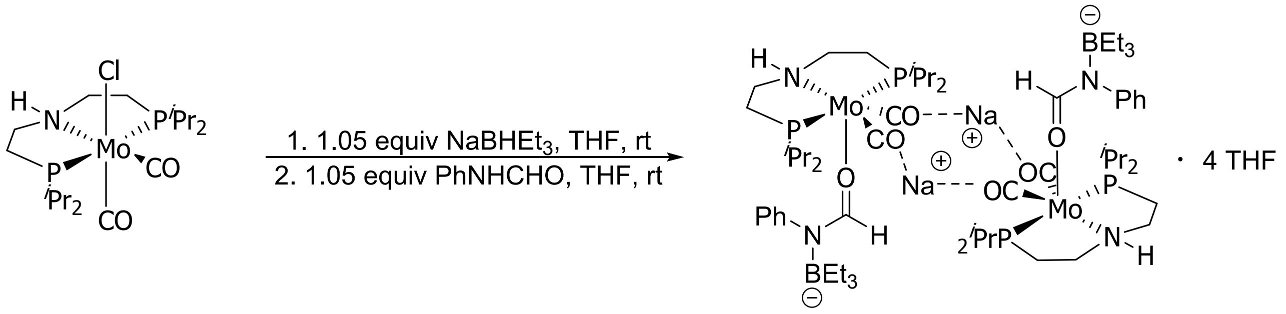

In a predried 25 mL Schlenk tube, 49.2 mg (100 µmol, 1.00 equiv) Mo-1a were suspended in 10 mL of dry THF under an argon atmosphere. The reaction mixture was treated dropwise with 1.05 equiv $NaBHEt_3$ (105 µL, 105 µmol, 1M in THF) and subsequently stirred for 1 h at room temperature. After this time, the occurring gas evolution had completely stopped. (At this stage, a gas tight syringe was used to take a representative sample of the obtained gas phase, which was immediately analysed by gas chromatography.) 12.7 mg (105 µmol, 1.00 equiv) formanilide were added in one portion resulting in a color change from red to brown. The reaction solution was stirred for one more hour and afterwards evaporated to dryness. The crude product was washed two times with 5 mL of pentane, redissolved in 2 mL of THF and subsequently filtered. The obtained clear brownish solution was first layered with 2 mL of *n*-pentane. However no single crystals suitable for X-ray crystallography were obtained. The mixture was layered with additional 2 mL of *n*-heptane, resulting in the growth of sharp brownish needles. The obtained crystals were of X-ray quality and thus used for the determination of the molecular structure of Mo-4.

X-ray crystal structure analysis of Mo-4:

Data were collected on a Bruker Kappa APEX II Duo diffractometer. The structure was solved by direct methods (SHELXS-97: Sheldrick, G. M. *Acta Cryst.* 2008, *A64*, 112.) and refined by full-matrix least-squares procedures on F^2 (SHELXL-2014: Sheldrick, G. M. *Acta Cryst.* 2015, *C71*, 3.). Contributions of co-crystallized disordered solvent molecules were removed from the diffraction data with PLATON/ SQUEEZE (Spek, A. L. *Acta Cryst.* 2015, *C*71, 9). XP (Bruker AXS) was used for graphical representations.

Crystal data of Mo-4: $C_{78}H_{148}B_2Mo_2N_4Na_2O_{10}P_4$, M = 1685.36, monoclinic, space group $P2_1/n$, a = 12.1273(11), b = 30.112(3), c = 29.714(3) Å, β = 100.836(2)°, V = 10657.4(17) Å^3, T = 150(2) K, Z = 4, 187828 reflections measured, 25687 independent reflections (R_{int} = 0.039), final R values ($I > 2\sigma(I)$): R_1 = 0.0496, wR_2 = 0.1157, final R values (all data): R_1 = 0.0603, wR_2 = 0.1228, 895 parameters.

CCDC 1916492 contains the supplementary crystallographic data for this paper. These data are provided free of charge by The Cambridge Crystallographic Data Centre.

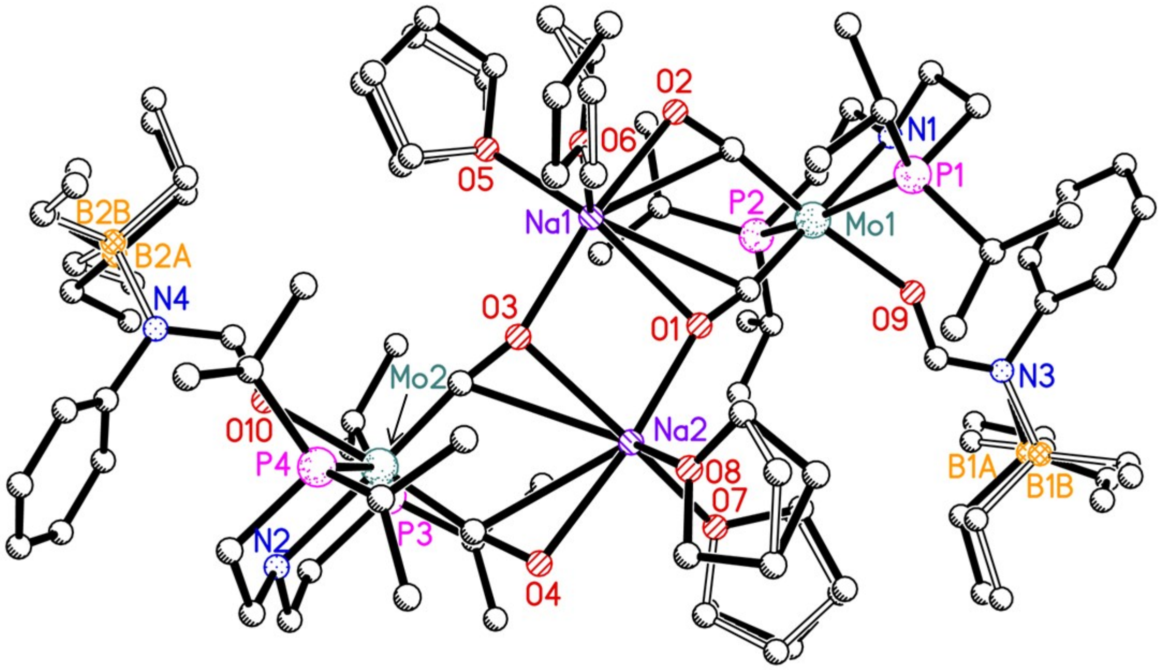

Ball and stick representation of Mo-4. Hydrogen atoms are omitted for clarity. Lower occupancy sites are depicted with open bonds.

Table 1. Further Optimisation of the General Catalytic Procedure

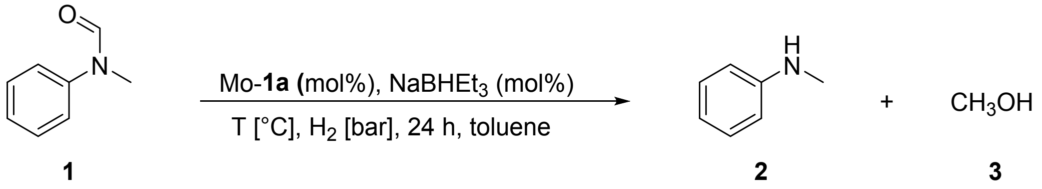

Entry[a,b]	T [°C]	H_2 (bar)	Mo-1a (mol%)	$NaBHEt_3$ (mol%)	Conv. (%)[c]	2 (%)[c]
1	80	30	5	10	20	18
2	80	50	5	5	87	85
3	100	50	5	5	>99	99
4	100	50	2.5	5	94	92
5	100	50	2.5	2.5	91	89

[a]Standard reaction conditions: N-methylformanilide 1 (67.6 mg, 0.5 mmol), Mo-1a (2.5-5 mol%), $NaBHEt_3$ (2.5-5 mol%) was used as commercially available stock solution (1M in THF), 2 mL toluene, 30-50 bar H_2, 24 h. [b]Yield of 3 was not determined. [c]Conversion of 1 and yield of 2 were determined by GC using hexadecane as internal standard.

Table 2. Screening of Aliphatic Formamides

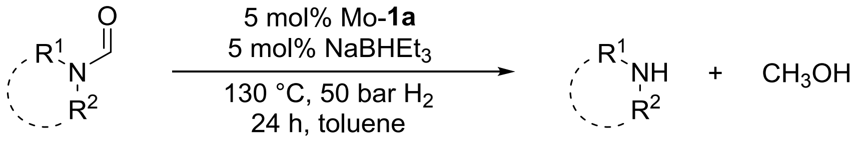

4a-c **5a-c** **3**

Entry[a,b]	Formamide	Conversion [%][c]	Yield 5 [%][d]
1	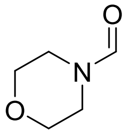 4a	<1	<1
2	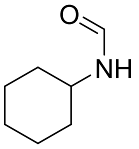 4b	<1	<1
3	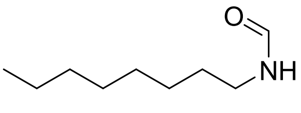 4c	6	4

[a]Standard reaction conditions: *N*-Methylformanilide (0.5 mmol), Mo-1a (12.5 mg, 5 mol%), $NaBHEt_3$ (50 µL, stock solution 0.5 M in THF, 5 mol%), 2 mL toluene, 50 bar H_2, 24 h. [b]Yield of 3 was not determined. [c]Conversions of *N*-methylformanilides were determined by GC using hexadecane as internal standard. [d]Yields were determined by GC using hexadecane as internal standard.

General Procedure for the Synthesis of *N*-Methylated Anilines[5]

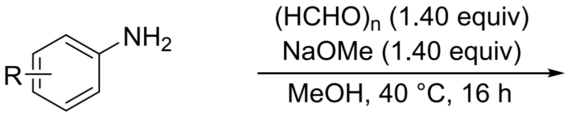

To a solution of anilines (10.0 mmol, 1.00 equiv) and sodium methoxide (14.0 mmol, 756 mg, 1.4 equiv) in MeOH (25 mL) was added paraformaldehyde (14.0 mmol, 420 mg, 1.40 equiv) in one portion. The reaction mixture was warmed to 40 °C and stirred for 16 h. Solid $NaBH_4$ (10.0 mmol, 378 mg, 1.00 equiv) was added to the reaction mixture and the reaction was stirred at 40 °C for further 3 h. The reaction mixture was quenched with 50 mL saturated aqueous $NaHCO_3$ and subsequently diluted with 50 mL of Et_2O. The phases were separated and the aqueous layer was washed with diethyl ether (3 x 20 mL). The organic layers were combined, washed with brine, dried over Na_2SO_4 and concentrated under reduced pressure. Purification by column chromatography (Ethylacetate/Heptane, gradient 0:100 → 100:0) on silica gel afforded *N*-methylanilines.

Characterization of *N*-methylanilines

N-methyl-4-(methylthio)aniline

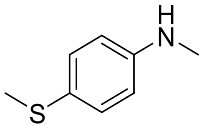

According to the general procedure, 4-(methylthio)aniline (1.39 g, 10.0 mmol) gave the title compound as colorless oil (1.41 g, 92 %).

^{1}H-NMR (400 MHz, $CDCl_3$) δ = 7.40 – 6.96 (m, 2H), 6.61 – 6.24 (m, 2H), 3.65 (s, 1H), 2.82 – 2.65 (m, 3H), 2.41 – 2.20 (m, 3H).

^{13}C-NMR (101 MHz, $CDCl_3$) δ = 148.3, 131.7, 124.1, 113.1, 30.8, 19.4.

N-methyl-[1,1'-biphenyl]-4-amine

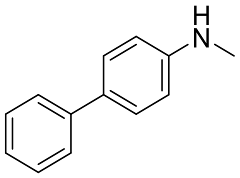

According to the general procedure, [1,1'-biphenyl]-4-amine (1.69 g, 10.0 mmol) gave the title compound as pale yellow solid (1.50 g, 82 %).

^{1}H-NMR (400 MHz, $CDCl_3$) δ = 7.61 – 7.52 (m, 2H), 7.49 – 7.46 (m, 2H), 7.43 – 7.36 (m, 2H), 7.33 – 7.21 (m, 1H), 6.74 – 6.65 (m, 2H), 3.82 (s, 1H), 2.89 (s, 3H).

^{13}C-NMR (101 MHz, $CDCl_3$) δ = 148.9, 141.4, 130.3, 128.8, 128.0, 126.4, 126.2, 112.8, 30.9.

(*E*)-*N*-methyl-4-styrylaniline

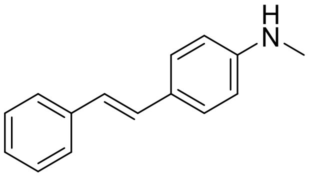

According to the general procedure, (*E*)-4-styrylaniline (1.95 g, 10.0 mmol) gave the title compound as an off white solid (1.74 g, 86 %).

^{1}H-NMR (300 MHz, $CDCl_3$) δ = 7.52 – 7.44 (m, 2H), 7.43 – 7.29 (m, 4H), 7.24 – 7.17 (m, 1H), 7.04 (d, *J* = 16.3 Hz, 1H), 6.91 (d, *J* = 16.3 Hz, 1H), 6.68 – 6.55 (m, 2H), 3.85 (s, 1H), 2.87 (s, 3H).

^{13}C-NMR (75 MHz, $CDCl_3$) δ = 149.1, 138.3, 129.0, 128.7, 127.9, 126.6, 126.2, 124.6, 112.6, 30.8.

4-(benzyloxy)-*N*-methylaniline

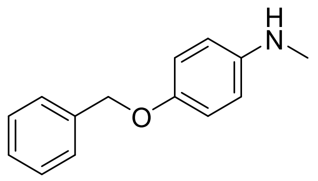

According to the general procedure, 4-(benzyloxy)aniline (1.99 g, 10.0 mmol) gave the title compound as white solid (1.66 g, 74 %).

^{1}H-NMR (400 MHz, $CDCl_3$) δ = 7.38 – 7.31 (m, 2H), 7.31 – 7.26 (m, 2H), 7.25 – 7.18 (m, 1H), 6.78 (d, *J* = 8.9 Hz, 2H), 6.48 (d, *J* = 8.9 Hz, 2H), 4.90 (s, 2H), 3.35 (s, 1H), 2.70 (s, 3H).

^{13}C-NMR (101 MHz, $CDCl_3$) δ = 151.3, 144.0, 137.7, 128.5, 127.8, 127.5, 116.2, 113.6, 70.9, 31.6.

2-Fluoro-*N*-methylaniline

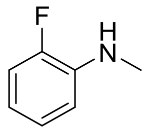

According to the general procedure, 2-fluoroaniline (1.11 g, 10.0 mmol) gave the title compound as colorless oil (1.05 g, 84 %).

^{1}H-NMR (300 MHz, $CDCl_3$) δ = 7.09 – 6.85 (m, 1H), 6.81 – 6.57 (m, 4H), 3.94 (s, 1H), 2.89 (s, 3H).

^{13}C-NMR (75 MHz, $CDCl_3$) δ = 124.7 (d, *J* = 3.4 Hz), 116.5 (d, *J* = 6.9 Hz), 114.3 (d, *J* = 18.2 Hz), 111.6 (d, *J* = 3.4 Hz), 30.4.

^{19}F-NMR (282 MHz, $CDCl_3$) δ -137.3.

N^1,N^1,N^4-trimethylbenzene-1,4-diamine

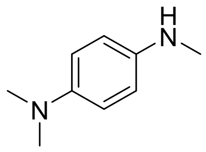

According to the general procedure, N^1,N^1-dimethylbenzene-1,4-diamine (1.36 g, 10.0 mmol) gave the title compound as a reddish oil (1.02 g, 68 %).

^{1}H-NMR (400 MHz, $CDCl_3$) δ = 6.78 (d, *J* = 8.3 Hz, 2H), 6.63 (d, *J* = 6.5 Hz, 2H), 3.17 – 2.50 (m, 9H).

^{13}C-NMR (75 MHz, $CDCl_3$) δ = 144.1, 142.1, 116.0, 113.9, 42.4, 31.7.

Synthesis of *N*-methyl-*N*-(4-(methylamino)phenyl)benzamide

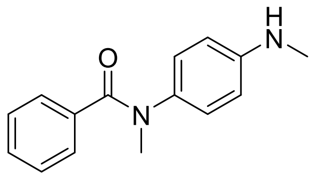

N[1],*N*[4]-dimethylbenzene-1,4-diamine (500 mg, 3.67 mmol, 1.00 equiv) and (445.8 mg, 0.61 mL, 4.40 mmol, 1.20 equiv) were dissolved in 10 mL of dry DCM and subsequently cooled to 0 °C in an ice bath. Benzoyl chloride (515.9 mg, 0.43 mL, 3.67 mmol, 1.00 equiv) was slowly added dropwise over 10 minutes and the reaction mixture was allowed to warm to room temperature overnight. The reaction mixture was quenched with 50 mL of water, stirred for 20 minutes at room temperature and afterwards diluted with 50 mL of DCM. The phases were separated and the aqueous phase was washed two times with 15 mL of DCM. The organic phases were combined, washed with brine and finally dried over Na_2SO_4. The drying agent was filtered off and the solvent was removed under reduced pressure. The crude product in form of a greenish solid was purified by column chromatography (Ethylacetate/Heptane, gradient 0:100 → 100:0) on silica gel to afford the title compound (285.58 mg, 32 %) as a white solid.

^{1}H-NMR (400 MHz, $CDCl_3$) δ = 7.40 – 7.22 (m, 2H), 7.15 (m, 3H), 6.82 (d, *J* = 8.2 Hz, 2H), 6.39 (d, *J* = 8.8 Hz, 2H), 3.42 (s, 3H), 2.72 (s, 3H).

^{13}C-NMR (101 MHz, $CDCl_3$) δ = 170.8, 147.8, 136.5, 134.5, 129.2, 128.7, 127.7, 127.6, 112.4, 38.7, 30.6.

IR (ATR, cm^{-1}): 3377 (m), 3026 (w), 2926 (w), 1864 (w), 1630 (s), 1613 (s), 1574 (w), 1523 (s), 1445 (m), 1422 (m), 1377 (m), 1327 (s), 1295 (m), 1270 (w), 1175 (w), 1153 (w), 1102 (w), 1069 (w), 1029 (w), 1013 (w), 919 (w), 876 (w), 816 (s), 790 (m), 719 (s), 694 (s), 638 (w), 571 (s), 531 (m), 494 (w), 448 (w), 391 (m).

Anal. Cacld. for $C_{15}H_{16}N_2O$: C, 74.97, H, 6.71, N, 11.66. Found: C, 74.94, H, 6.69, N, 11.68.

General Procedure for the Synthesis of *N*-Methylated Formanilides[4]

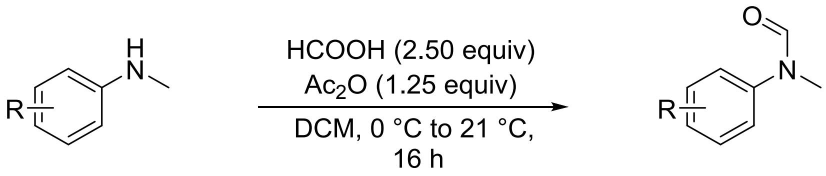

A mixture of formic acid (575 mg, 943 µL, 12.5 mmol, 2.50 equiv.) and Ac_2O (638 mg, 590 µL, 6.25 mmol, 1.25 equiv) was stirred at r.t. for 1 h and subsequently added to a solution of *N*-methylaniline (5 mmol, 1.00 equiv) in DCM (5 mL) at 0 °C. The resulting mixture was allowed to warm to room temperature and stirred for 16 h. The reaction mixture was quenched with 25 mL saturated aqueous $NaHCO_3$ and diluted with 25 mL of DCM. The layers were separated and the aqueous phase was washed with DCM (3 x 10 mL). The organic phases were combined, washed with brine and dried over Na_2SO_4. The solvent was evaporated under reduced pressure and the obtained residue was purified by column chromatography (Ethylacetate/Heptane, gradient 0:100 → 100:0) on silica gel to afford *N*-methylformanilides.

N-(2-fluorophenyl)-*N*-methylformamide

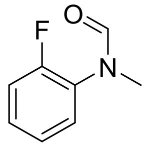

According to the general procedure, 2-fluoro-*N*-methylaniline (625.8 mg, 5.0 mmol) gave the title compound as colorless liquid (214.4 mg, 28 %).

^{1}H-NMR (300 MHz, $CDCl_3$) δ = 8.19 (s, 1H), 7.29 – 7.18 (m, 1H), 7.17 – 7.07 (m, 3H), 3.20 (s, 3H).

^{13}C-NMR (75 MHz, $CDCl_3$) δ = 162.9 (d, *J* = 2.3 Hz), 159.1, 155.8, 129.0 (d, *J* = 7.8 Hz), 127.4 (d, *J* = 1.3 Hz), 125.0 (d, *J* = 4.0 Hz), 117.1 (d, *J* = 20.1 Hz), 32.9.

^{19}F-NMR (282 MHz, $CDCl_3$) δ = –122.6.

N-(3-fluorophenyl)-*N*-methylformamide

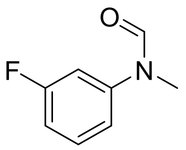

According to the general procedure, 3-fluoro-*N*-methylaniline (625.8 mg, 5.0 mmol) gave the title compound as colorless liquid (753.2 mg, 96 %).

^{1}H-NMR (300 MHz, $CDCl_3$) δ = 8.52 (s, 1H), 7.37 (m, 1H), 7.03 – 6.80 (m, 3H), 3.31 (s, 3H).

^{13}C-NMR (75 MHz, $CDCl_3$) δ = 165.0, 162.1, 143.9 (d, *J* = 9.8 Hz), 131.0 (d, *J* = 9.3 Hz), 117.5 (d, *J* = 3.2 Hz), 113.2 (d, *J* = 21.0 Hz), 109.4 (d, *J* = 24.1 Hz), 32.0.

^{19}F-NMR (282 MHz, $CDCl_3$) δ = -110.5.

IR (ATR, cm^{-1}): 3071 (w), 2897 (w), 1670 (s), 1607 (s), 1558 (s), 1493 (m), 1413 (w), 1324 (m), 1256 (m), 1198 (m), 1163 (w), 1107 (m), 1072 (m), 994 (m), 890 (w), 854 (m), 781 (s), 718 (w), 688 (s), 665 (w), 615 (w), 522 (m), 489 (m),458 (w), 409 (m).

Anal. Cacld. for C_8H_8FNO: C, 53.21; H, 3.97; N, 6.89. Found: C, 53.19; H, 3.99; N, 6.90.

N-(3-methoxyphenyl)-*N*-methylformamide

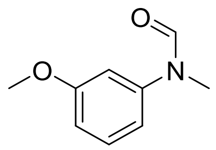

According to the general procedure, 4-methoxy-*N*-methylaniline (685.9 mg, 5.0 mmol) gave the title compound as colorless liquid (743.4 mg, 90 %).

^{1}H-NMR (400 MHz, $CDCl_3$) δ = 8.42 (s, 1H), 7.27 – 7.18 (m, 1H), 6.72 (m, 2H), 6.63 (m, 1H), 3.76 (s, 3H), 3.23 (s, 3H).

^{13}C-NMR (101 MHz, $CDCl_3$) δ = 162.4, 160.6, 143.5, 130.5, 114.6, 111.5, 108.7, 55.6, 32.1.

N-methyl-*N*-(3-(trifluoromethyl)phenyl)formamide

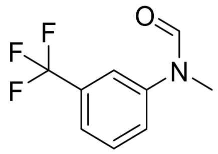

According to the general procedure, *N*-methyl-3-(trifluoromethyl)aniline (875.8 mg, 5.0 mmol) gave the title compound as colorless liquid (893.9 mg, 88 %).

^{1}H-NMR (300 MHz, $CDCl_3$) δ = 7.42 – 7.13 (m, 1H), 7.09 – 6.85 (m, 1H), 6.86 – 6.66 (m, 2H), 3.91 (s, 1H), 2.87 (s, 3H).

^{13}C-NMR (75 MHz, $CDCl_3$) δ = 149.5, 131.7 (d, *J* = 31.6 Hz), 129.7, 126.4, 122.7, 115.6, 113.7 (q, *J* = 3.9 Hz), 108.5 (q, *J* = 3.9 Hz), 30.6.

^{19}F-NMR (282 MHz, $CDCl_3$) δ = -62.9.

IR (ATR, cm^{-1}): 3072 (w), 2886 (w), 1675 (s), 1613 (m), 1594 (m), 1496 (m), 1456 (m), 1415 (w),1326 (s), 1296 (w), 1283 (w), 1256 (m), 1165 (m), 1117 (s), 1097 (s), 1071 (s), 1001 (m), 986 (m), 885 (m), 851 (w), 797 (m), 774 (w), 735 (w), 691 (s), 656 (w), 533 (w), 475 (w), 422 (w).

Anal. Cacld. for $C_9H_8F_3NO$: C, 53.21, H, 3.97, N, 6.89. Found: C, 53.23, H, 3.96, N, 6.93.

N-methyl-*N*-(4-(trifluoromethyl)phenyl)formamide

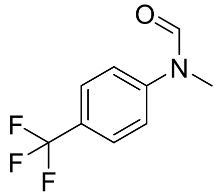

According to the general procedure, *N*-methyl-4-(trifluoromethyl)aniline (875.8 mg, 5.0 mmol) gave the title compound as a pale white solid (995.5mg, 98 %).

^{1}H-NMR (300 MHz, $CDCl_3$) δ = 8.53 (s, 1H), 7.67 – 7.53 (m, 2H), 7.28 – 7.17 (m, 2H), 3.29 (s, 3H).

^{13}C-NMR (75 MHz, $CDCl_3$) δ = 161.8, 145.1, 126.9 (q, *J* = 32.5 Hz), 125.6, 123.8 (q, *J* = 269 Hz) 121.4, 31.6.

^{19}F-NMR (282 MHz, $CDCl_3$) δ = -62.4.

N-(4-fluorophenyl)-*N*-methylformamide

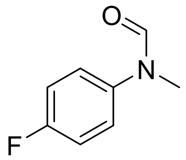

According to the general procedure, 4-fluoro-*N*-methylaniline (625.8 mg, 5.0 mmol) gave the title compound as colorless liquid (719.6 mg, 94 %).

^{1}H-NMR (300 MHz, $CDCl_3$) δ = 8.37 (s, 1H), 7.20 – 7.01 (m, 4H), 3.28 (s, 3H).

^{13}C-NMR (75 MHz, $CDCl_3$) δ = 162.8, 159.5, 138.4 (d, *J* = 3.1 Hz), 124.7 (d, *J* = 8.5 Hz), 116.6 (d, *J* = 22.8 Hz), 32.6.

^{19}F-NMR (282 MHz, $CDCl_3$) δ = -115.4.

N-methyl-*N*-(*p*-tolyl)formamide

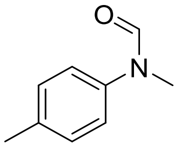

According to the general procedure, *N*,4-dimethylaniline (605.9 mg, 5.0 mmol) gave the title compound as colorless liquid (611.7 mg, 82 %).

^{1}H-NMR (300 MHz, $CDCl_3$) = δ 8.34 (s, 1H), 7.17 – 7.09 (m, 2H), 7.03 – 6.94 (m, 2H), 3.21 (s, 3H), 2.29 (s, 3H).

^{13}C-NMR (75 MHz, $CDCl_3$) δ = 162.4, 139.8, 136.5, 130.2, 122.7, 32.3, 21.0.

N-([1,1'-biphenyl]-4-yl)-*N*-methylformamide

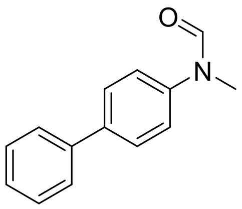

According to the general procedure, *N*-methyl-[1,1'-biphenyl]-4-amine (916.3 mg, 5.0 mmol) gave the title compound as white solid (918.9 mg, 87 %).

^{1}HNMR (400 MHz, $CDCl_3$) δ = 8.48 (s, 1H), 7.59 – 7.48 (m, 4H), 7.42 – 7.34 (m, 2H), 7.33 – 7.27 (m, 1H), 7.21 – 7.15 (m, 2H), 3.29 (s, 3H).

^{13}C-NMR (101 MHz, $CDCl_3$) δ = 162.2, 141.3, 139.9, 139.4 128.9, 128.3, 127.6, 127.0, 122.5, 32.1.

IR (ATR, cm^{-1}): 3317 (w), 3072 (w), 3029 (w), 2901 (w), 1661 (m), 1603 (m), 1566 (m), 1552 (m), 1481 (m). 1451 (m), 1399 (m), 1339 (m), 1314 (m), 1278 (m), 1258 (m), 1106 (m), 1038 (w), 1023 (w), 1006 (w), 973 (m), 913 (w), 840 (m), 765 (s), 744 (w), 724 (m), 693 (m), 648 (w), 566 (m), 499 (m), 468 (m), 401 (m).

Anal. Cacld. for $C_{14}H_{13}NO$: C, 79.59; H, 6.20; N, 6.63. Found: C, 79.64; H, 6.24; N, 6.60.

N-(4-methoxyphenyl)-*N*-methylformamide

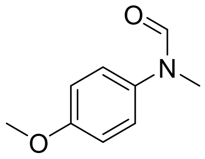

According to the general procedure, 4-methoxy-*N*-methylaniline (685.9 mg, 5.0 mmol) gave the title compound as white solid (776.4 mg, 94 %).

^{1}HNMR (400 MHz, $CDCl_3$) δ = 8.26 (s, 1H), 7.09 – 6.96 (m, 2H), 6.91 – 6.77 (m, 2H), 3.74 (s, 3H), 3.19 (s, 3H).

^{13}C-NMR (75 MHz, $CDCl_3$) δ = 162.3, 158.2, 124.5, 114.7, 114.2, 55.4, 32.5.

N-methyl-*N*-(4-(methylthio)phenyl)formamide

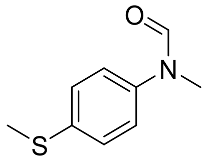

According to the general procedure, *N*-methyl-4-(methylthio)aniline (766.2 mg, 5.0 mmol) gave the title compound as white solid (815.6 mg, 90 %).

^{1}H-NMR (400 MHz, $CDCl_3$) δ = 8.36 (s, 1H), 7.24 – 7.19 (m, 2H), 7.05 – 7.00 (m, 2H), 3.22 (s, 3H), 2.42 (s, 3H).

^{13}C NMR (101 MHz, $CDCl_3$) δ = 162.1, 139.4, 136.8, 127.8, 123.0, 32.2, 16.1.

IR (ATR, cm^{-1}): 3495 (w), 2919 (w), 1664 (s), 1593 (m), 1566 (w), 1495 (s), 1436 (w), 1403 (w), 1334 (m), 1319 (m), 1306 (m), 1288 (m), 1257 (m), 1114 (s), 1054 (m), 1011 (m), 976 (m), 819 (s), 711 (m), 668 (w), 535 (m), 520 (m), 449 (w).

Anal. Cacld. for C_9H11NOS: C, 59.64, H, 6.12, N, 7.73. Found: C, 59.66, H, 6.09, N, 7.73.

N-(4-cyanophenyl)-*N*-methylformamide

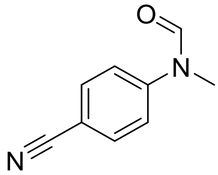

According to the general procedure, 4-(methylamino)benzonitrile (660.9 mg, 5.0 mmol) gave the title compound as white solid (624.7 mg, 78 %).

^{1}H-NMR (300 MHz, $CDCl_3$) δ = 8.59 (s, 1H), 7.71 – 7.52 (m, 3H), 7.26 – 7.15 (m, 2H), 3.29 (s, 3H).

^{13}C-NMR (75 MHz, $CDCl_3$) δ = 161.5, 145.9, 133.7, 121.1, 118.1, 109.4, 31.3.

N-methyl-*N*-(4-nitrophenyl)formamide

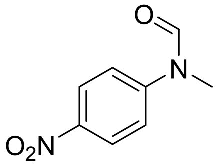

According to the general procedure, *N*-methyl-4-nitroaniline (660.9 mg, 5.0 mmol) gave the title compound as white solid (720.7 mg, 80 %).

^{1}H-NMR (300 MHz, $CDCl_3$) δ = 8.66 (s, 1H), 8.29 – 8.09 (m, 2H), 7.29 – 7.22 (m, 2H), 3.32 (s, 3H).

^{13}C-NMR (75 MHz, $CDCl_3$) δ = 161.5, 147.5, 125.4, 121.9, 120.4, 31.4.

N-methyl-*N*-(pyridin-4-yl)formamide

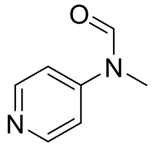

According to the general procedure, *N*-methylpyridin-4-amine (540.7 mg, 5.0 mmol) gave the title compound as white solid (449.3 mg, 66 %).

^{1}H-NMR (300 MHz, $CDCl_3$) δ = 8.75 (s, 1H), 8.57 – 8.45 (m, 2H), 7.14 – 6.95 (m, 2H), 3.26 (s, 3H).

^{13}C-NMR (75 MHz, $CDCl_3$) δ = 161.1, 151.1, 148.8, 113.7, 30.2.

IR (ATR, cm^{-1}): 3246 (w), 3095 (w), 3065 (w), 3029 (w), 2932 (w), 2784 (w), 2689 (w), 1648 (s), 1583 (s), 1559 (s), 1544 (s), 1511 (s), 1435 (m), 1417 (m), 1357 (m), 1326 (s), 1286 (m), 1228 (m), 1201 (s), 1121 (m), 1069 (m), 992 (m), 970 (m), 817 (s), 758 (m), 672 (m), 543 (s), 504 (s), 449 (s), 412 (s).

Anal. Cacld. for $C_7H_8N_2O$: C, 61.75, H, 5.92, N, 20.58. Found: C, 61.73, H, 5.91, N, 20.60.

Methyl-4-(*N*-methylformamido)benzoate

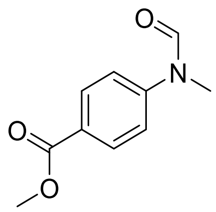

According to the general procedure, methyl-4-(methylamino)benzoate (825.9 mg, 5.0 mmol) gave the title compound as white solid (811.4 mg, 84 %).

^{1}H NMR (300 MHz, $CDCl_3$) δ = 7.92 – 7.83 (m, 2H), 6.62 – 6.49 (m, 2H), 4.28 (s, 1H), 3.85 (s, 3H), 2.89 (s, 3H).

^{13}C NMR (75 MHz, $CDCl_3$) δ = 167.5, 152.9, 131.6, 118.5, 111.3, 51.7, 30.3.

N-(4-(benzyloxy)phenyl)-*N*-methylformamide

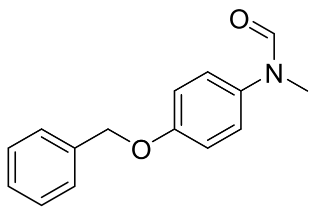

According to the general procedure, 4-(benzyloxy)-*N*-methylaniline (1.07 g, 5.0 mmol) gave the title compound as white solid (953.1 mg, 79 %).

^{1}HNMR (400 MHz, $CDCl_3$) δ = 8.28 (s, 1H), 7.38 – 7.23 (m, 5H), 7.06 – 6.99 (m, 2H), 6.96 – 6.90 (m, 2H), 5.01 (s, 2H), 3.20 (s, 3H).

^{13}C-NMR (101 MHz, $CDCl_3$) δ = 162.4, 157.4, 136.6, 135.5, 128.7, 128.2, 127.4, 124.6, 115.7, 70.3, 32.7.

IR (ATR, cm^{-1}): 3329 (w), 3070 (w), 3034 (w), 3009 (w), 2933 (w), 2889 (w), 1668 (s), 1584 (m), 1511 (s), 1466 (m), 1457 (m), 1431 (w), 1402 (w), 1383 (m), 1344 (m), 1294 (w), 1276 (m), 1232 (s), 1180 (m), 1115 (m), 1013 (s), 978 (m), 956 (w), 935 (w), 918 (w), 864 (m), 836 (s), 816 (m), 749 (s), 720 (w), 698 (s), 656 (m), 576 (w), 547 (m), 510 (m), 466 (w), 428 (m).

Anal. Cacld. for $C_{15}H_{15}NO_2$: C, 74.67, H, 6.21, N, 5.81. Found: C, 74.68, H, 6.22, N, 5.79.

(E)-N-methyl-N-(4-styrylphenyl)formamide

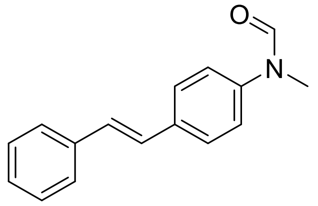

According to the general procedure, (E)-N-methyl-4-styrylaniline (1.05 g, 5.0 mmol) gave the title compound as white solid (961.1 mg, 81 %).

^{1}H-NMR (400 MHz, $CDCl_3$) δ = 8.68 (s, 1H), 7.74 – 7.66 (m, 5H), 7.57 – 7.50 (m, 2H), 7.47 – 7.42 (m, 2H), 7.35 – 7.31 (m, 2H), 3.50 (s, 3H).

^{13}C-NMR (101 MHz, $CDCl_3$) δ = 162.1, 141.3, 137.0, 135.6, 129.3, 128.8, 127.9, 127.6, 127.3, 126.6, 122.3, 32.0.

IR (ATR, cm^{-1}): 3023 (w), 2890 (w), 1665 (s), 1592 (m), 1568 (m), 1512 (m), 1447 (m), 1414 (w), 1330 (m), 1309 (m), 1260 (m), 1220 (w), 1190 (w), 1113 (m), 1072 (w), 1012 (w), 969 (s), 945 (m), 864 (w), 845 (w), 822 (s), 804 (w), 758 (s), 721 (m), 689 (s), 670 (m), 637 (w), 579 (w), 551 (s), 499 (w), 479 (w), 436 (w), 419 (m).

Anal. Cacld. for $C_{16}H_{15}NO$: C, 80.98, H, 6.37, N, 5.90. Found: C, 81.00, H, 6.39, N, 5.92.

N-(4-(dimethylamino)phenyl)-N-methylformamide

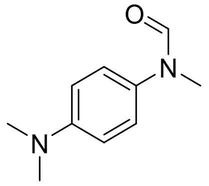

According to the general procedure, N^1,N^1,N^4-trimethylbenzene-1,4-diamine (751.2 mg, 5.0 mmol) gave the title compound as white solid (525.8 mg, 59 %).

^{1}H-NMR (400 MHz, $CDCl_3$) δ = 8.24 (s, 1H), 6.99 – 6.95 (m, 2H), 6.74 – 6.51 (m, 2H), 3.18 (s, 3H), 2.90 (s, 6H).

^{13}C-NMR (101 MHz, $CDCl_3$) δ = 162.6, 149.5, 131.5, 124.8, 112.9, 40.7, 32.9.

N-methyl-*N*-(4-(*N*-methylformamido)phenyl)benzamide

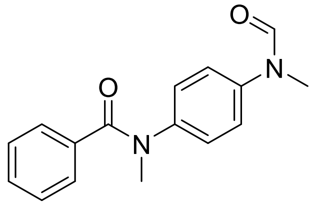

According to the general procedure, *N*-methyl-*N*-(4-(methylamino)phenyl)benzamide (250.0 mg, 1.04 mmol) gave the title compound as white solid (255.1 mg, 95 %).

^{1}H-NMR (300 MHz, CDCl$_3$) δ = 8.35 (s, 1H), 7.27 – 7.08 (m, 5H), 7.05 – 6.91 (m, 4H), 3.41 (s, 3H), 3.17 (s, 3H).

^{13}C-NMR (75 MHz, CDCl$_3$) δ = 170.6, 162.0, 142.9, 140.2, 135.7, 129.8, 128.6, 127.9, 122.5, 38.4, 31.9.

IR (ATR, cm^{-1}): 3063 (w), 2978 (w), 2913 (w), 2849 (w), 1659 (s), 1644 (s), 1600 (m), 1509 (s), 1443 (m), 1409 (m), 1362 (m), 1337 (s), 1314 (m), 1295 (m), 1260 (m), 1175 (w), 1158 (w), 1112 (s), 1073 (w), 1018 (m), 1006 (m), 977 (m), 919 (w), 854 (s), 817 (m), 784 (m), 738 (m), 718 (s), 694 (s), 671 (w), 666 (m), 591 (s), 555 (m), 517 (m), 476 (m), 448 (w), 410 (m).

Anal. Cacld. for $C_{16}H_{16}N_2O_2$: C, 71.62, H, 6.01, N, 10.44. Found: C, 71.64, H, 6.04, N, 10.47.

IR spectra of the previously unknown *N*-methylformanilides and *N*-methylanilines

N-methyl-*N*-(pyridin-4-yl)formamide

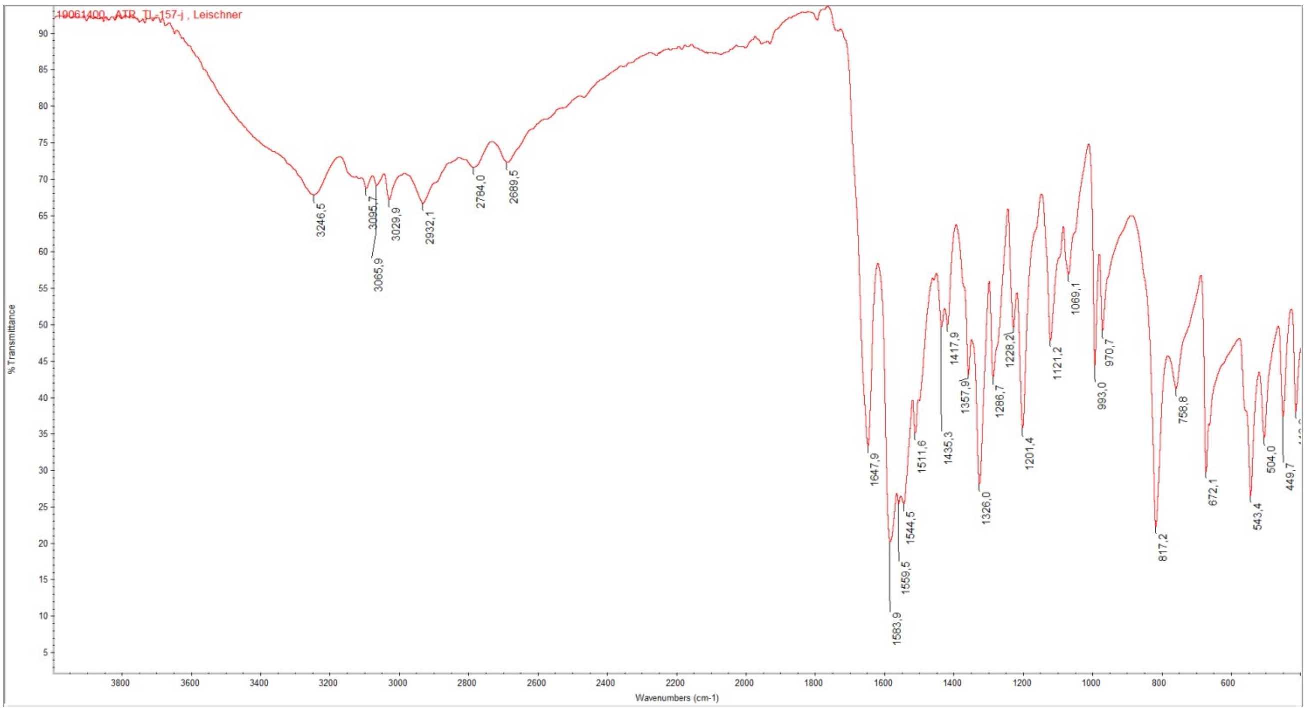

N-([1,1'-biphenyl]-4-yl)-*N*-methylformamide

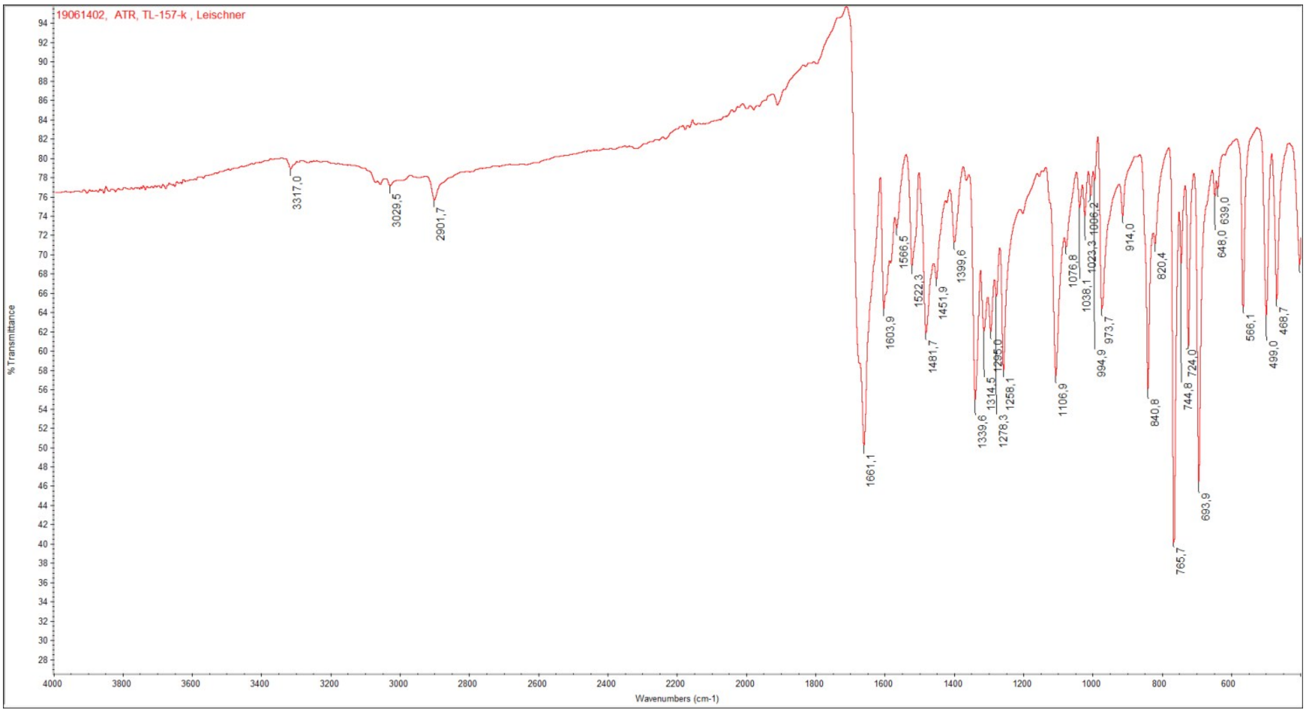

N-methyl-N-(4-(methylthio)phenyl)formamide

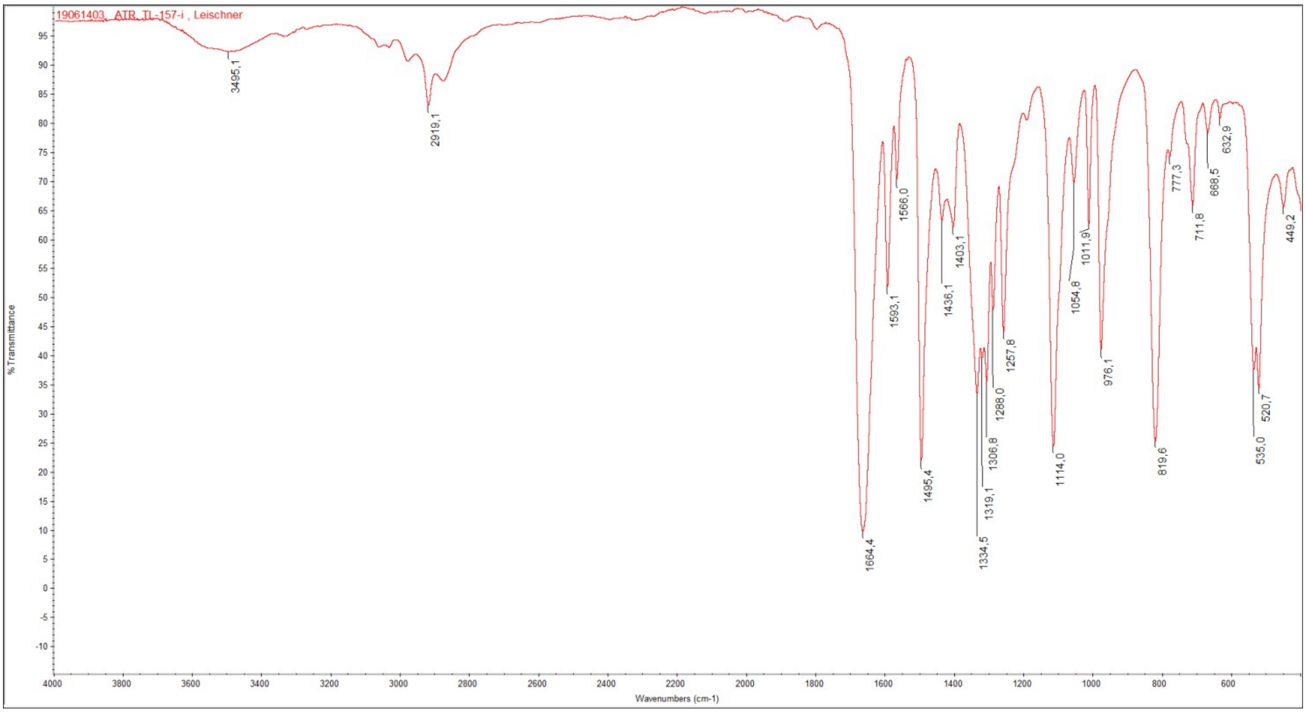

N-(4-(benzyloxy)phenyl)-*N*-methylformamide

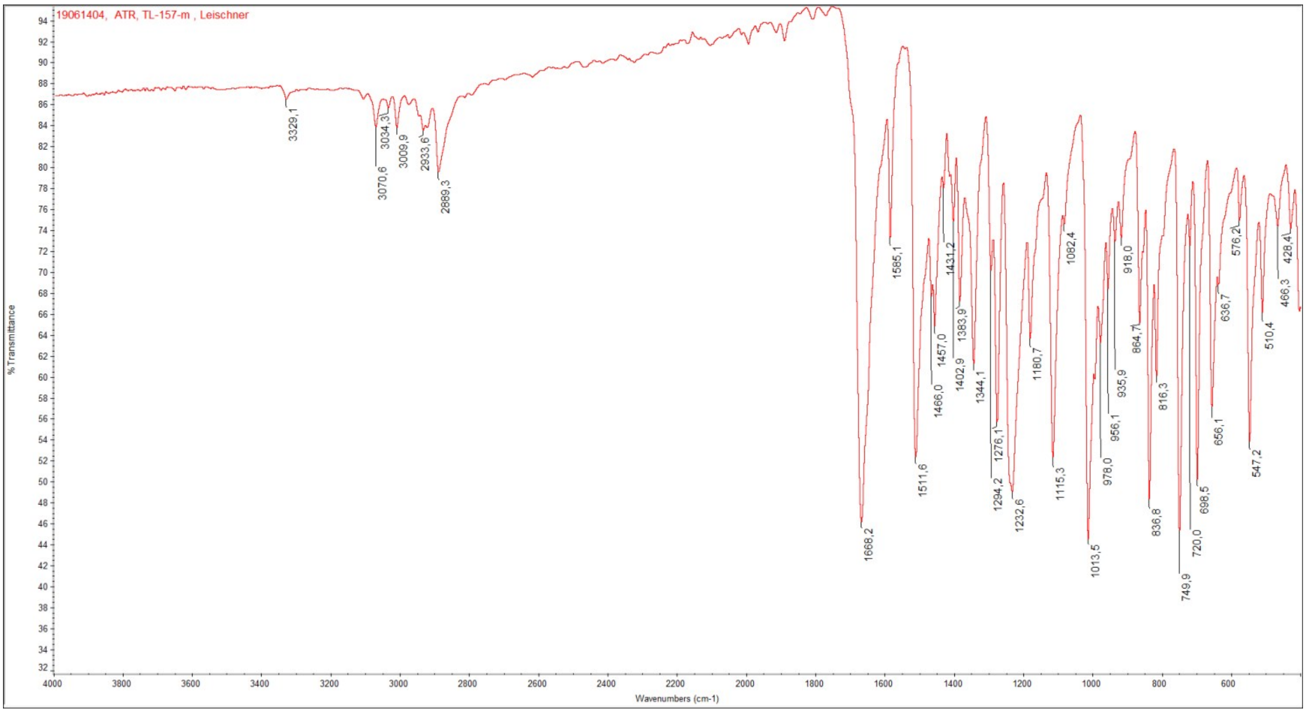

(*E*)-*N*-methyl-*N*-(4-styrylphenyl)formamide

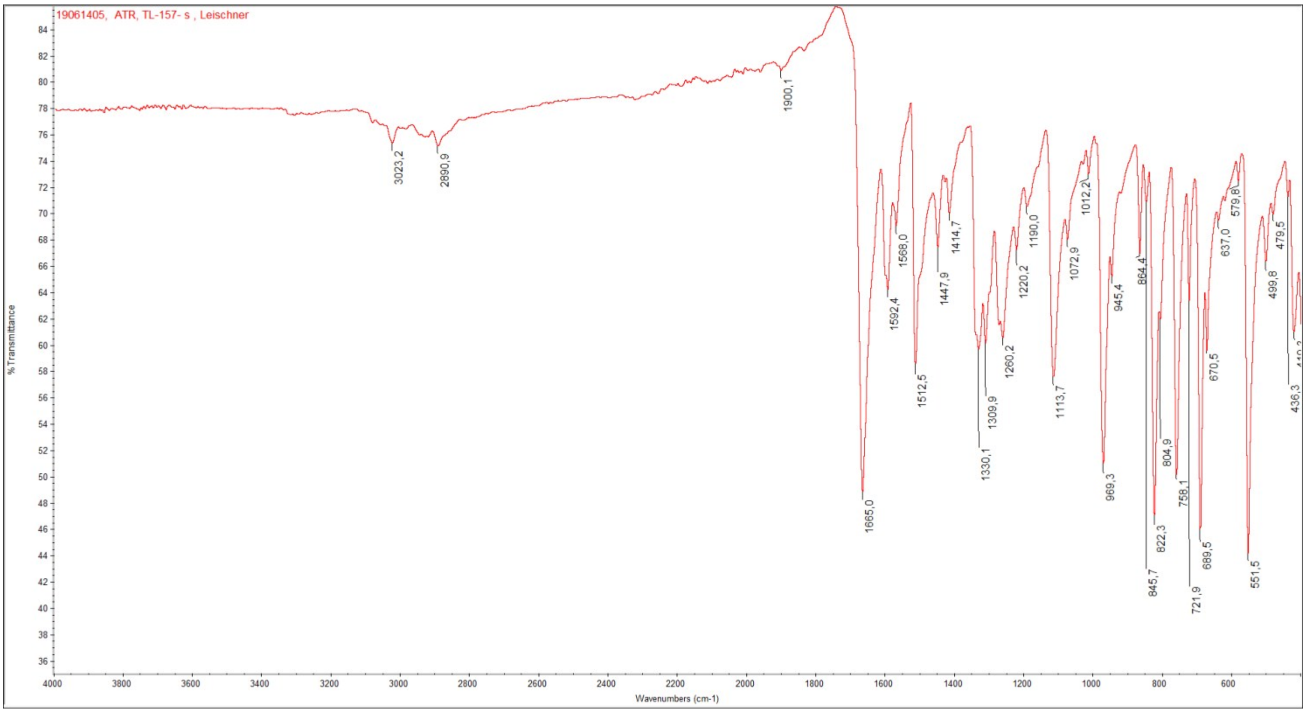

N-methyl-*N*-(3-(trifluoromethyl)phenyl)formamide

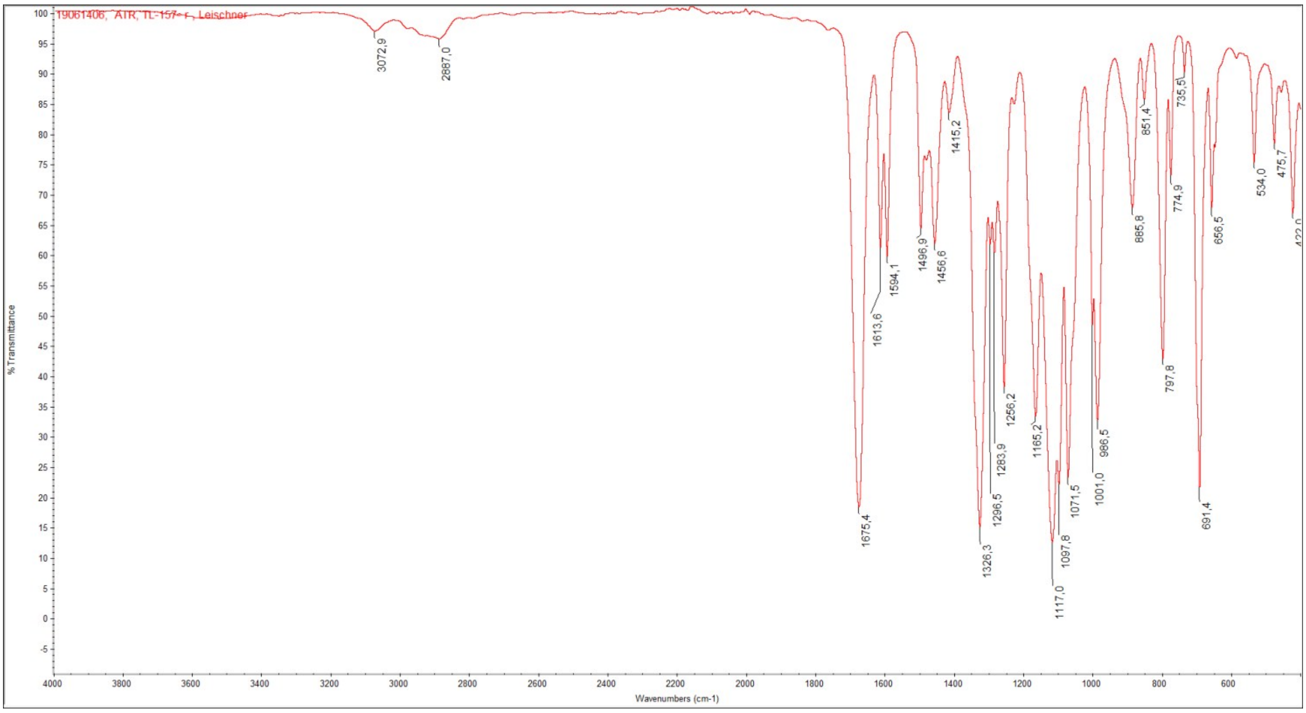

N-(3-fluorophenyl)-*N*-methylformamide

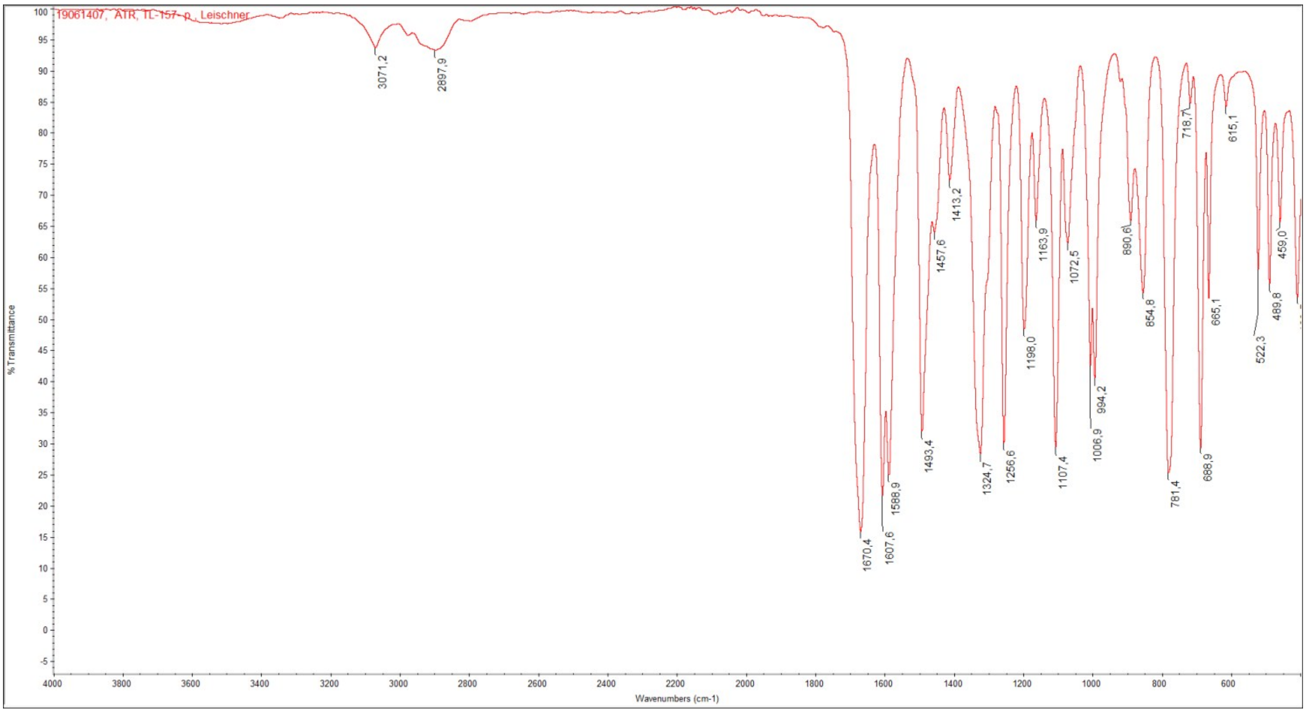

N-methyl-*N*-(4-(*N*-methylformamido)phenyl)benzamide

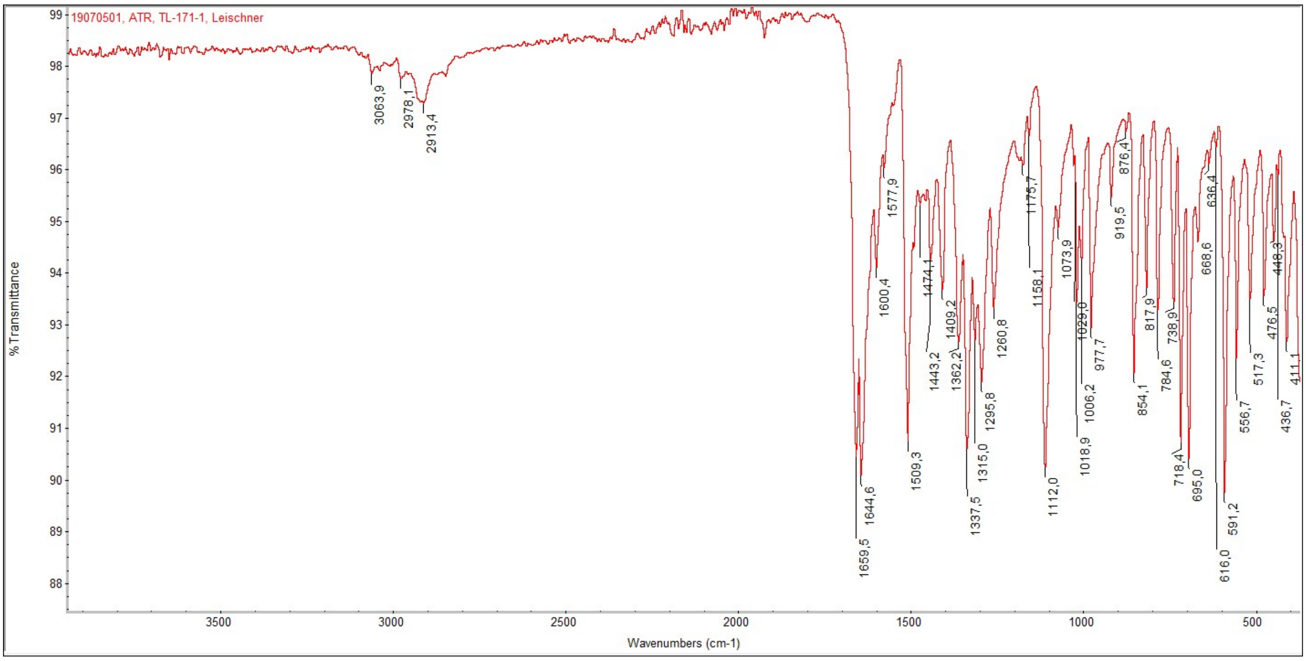

N-methyl-*N*-(4-(methylamino)phenyl)benzamide

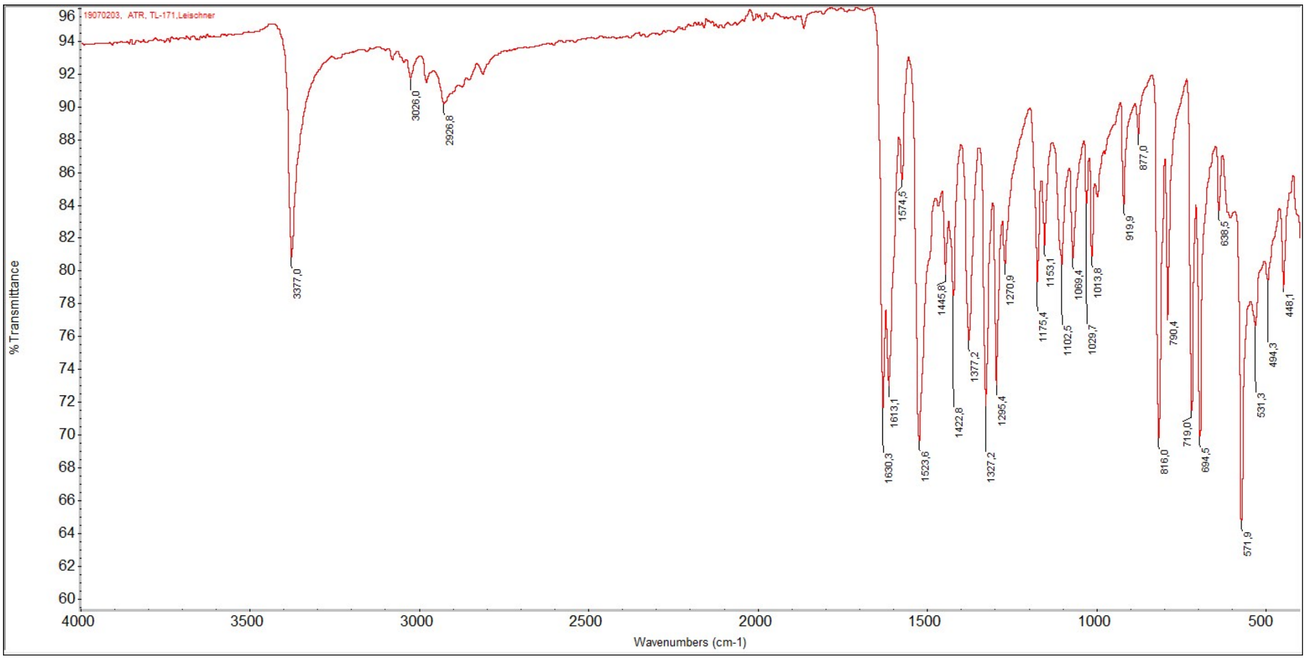

NMR spectra of the previously unknown *N*-methylformanilides

N-methyl-*N*-(pyridin-4-yl)formamide

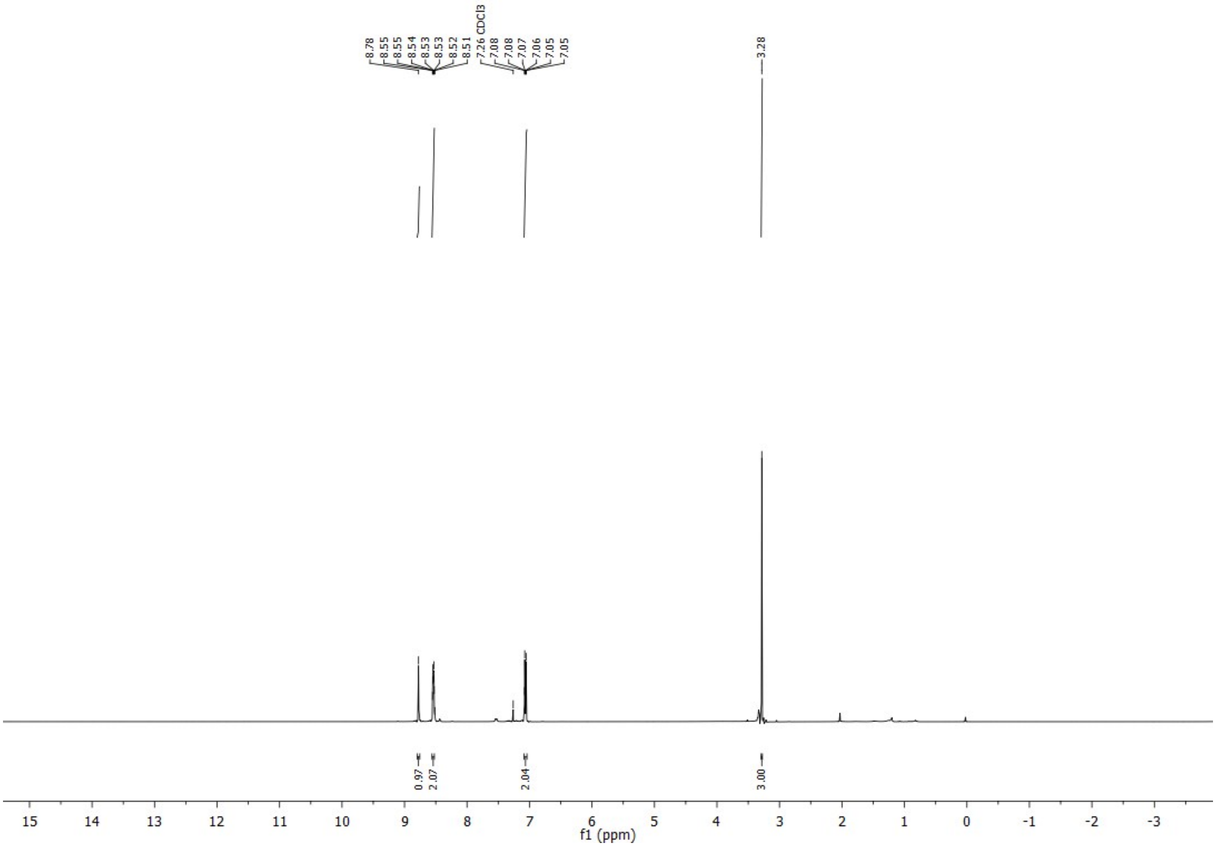

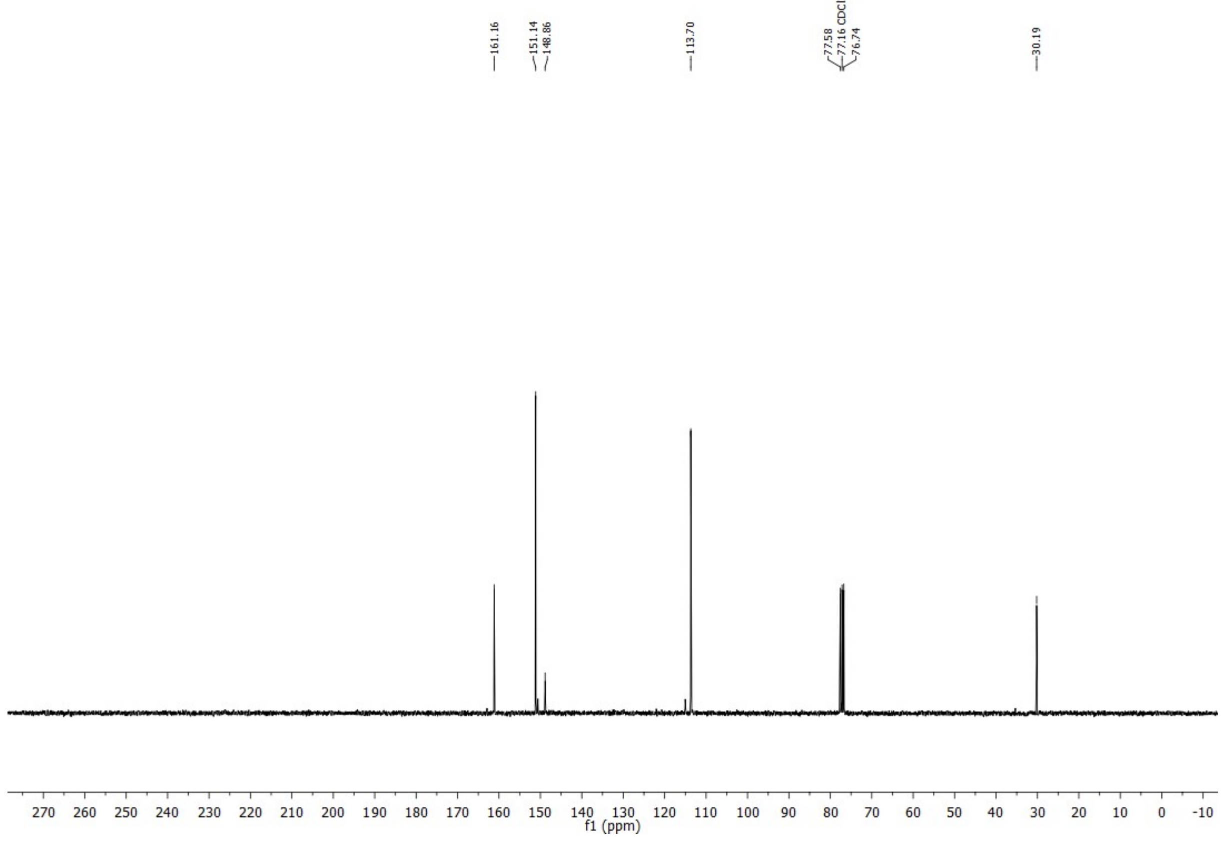

161.16
151.14
148.86
113.70
77.58
77.16 CDCl3
76.74
30.19
270 260 250 240 230 220 210 200 190 180 170 160 150 140 130 120 110 100 90 80 70 60 50 40 30 20 10 0 -10
f1 (ppm)

N-([1,1'-biphenyl]-4-yl)-*N*-methylformamide

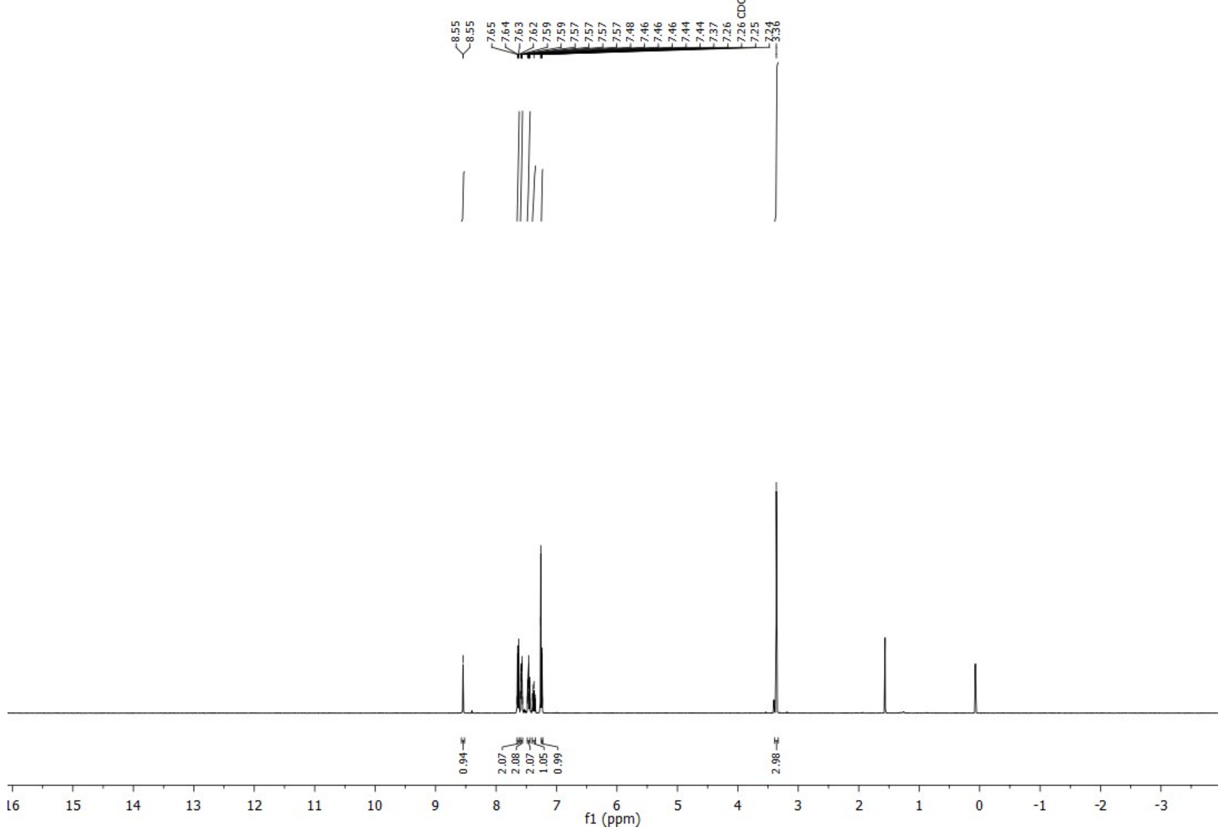

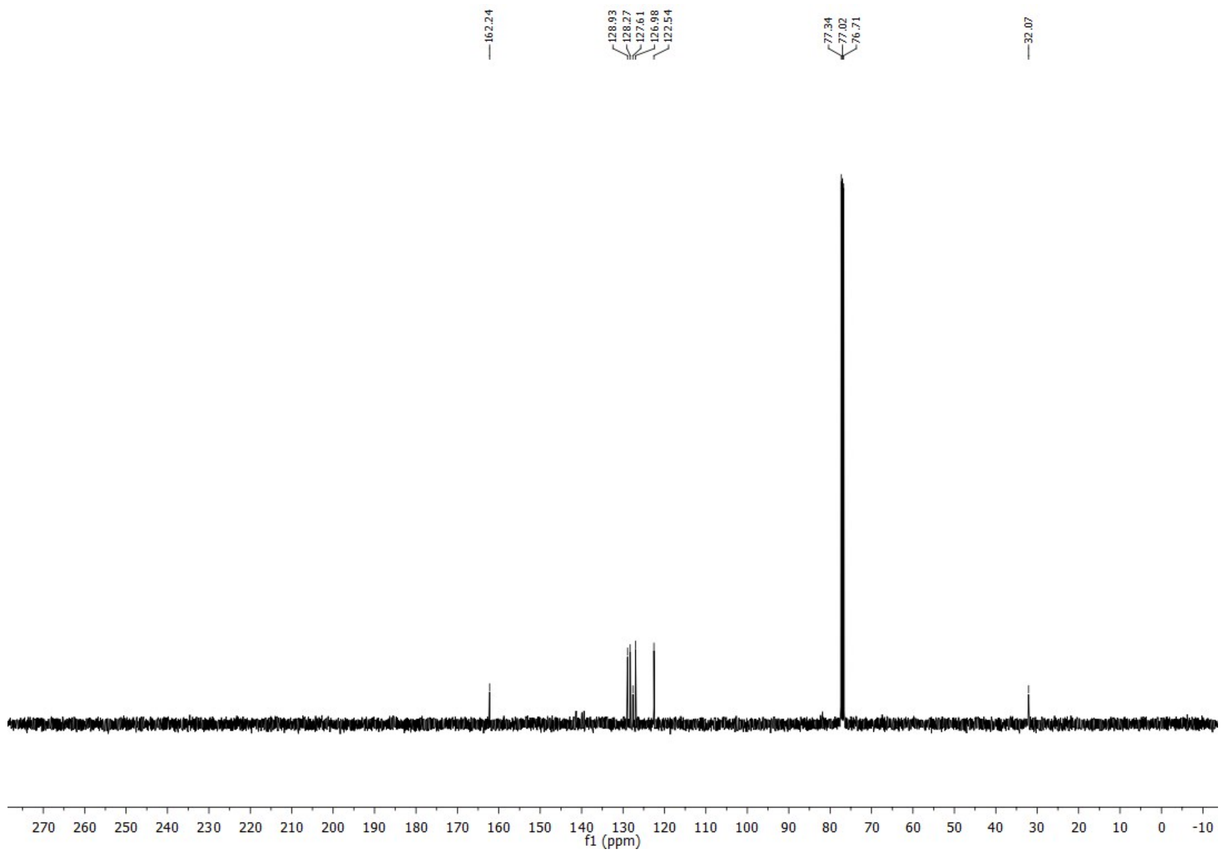
162.24
128.93
128.27
127.61
126.98
122.54
77.34
77.02
76.71
32.07
270 260 250 240 230 220 210 200 190 180 170 160 150 140 130 120 110 100 90 80 70 60 50 40 30 20 10 0 -10
f1 (ppm)

N-methyl-*N*-(4-(methylthio)phenyl)formamide

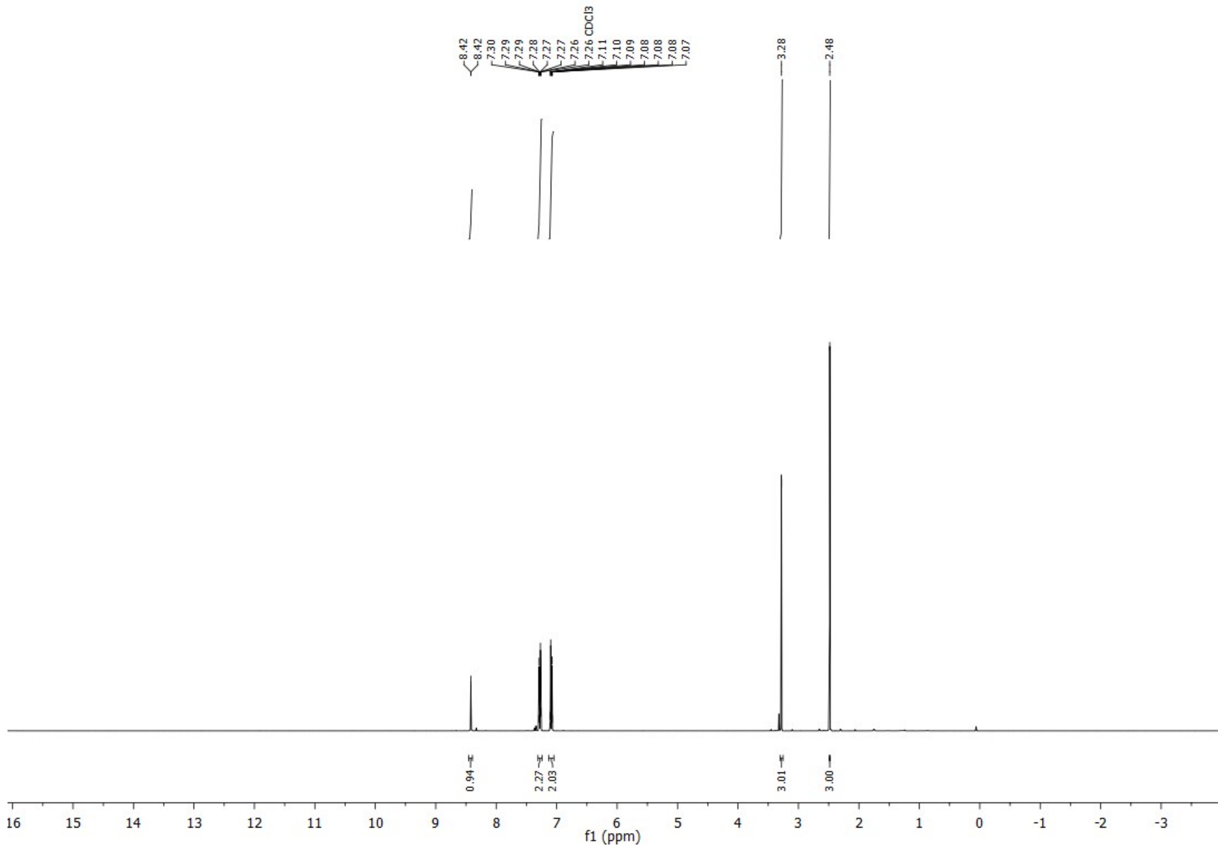

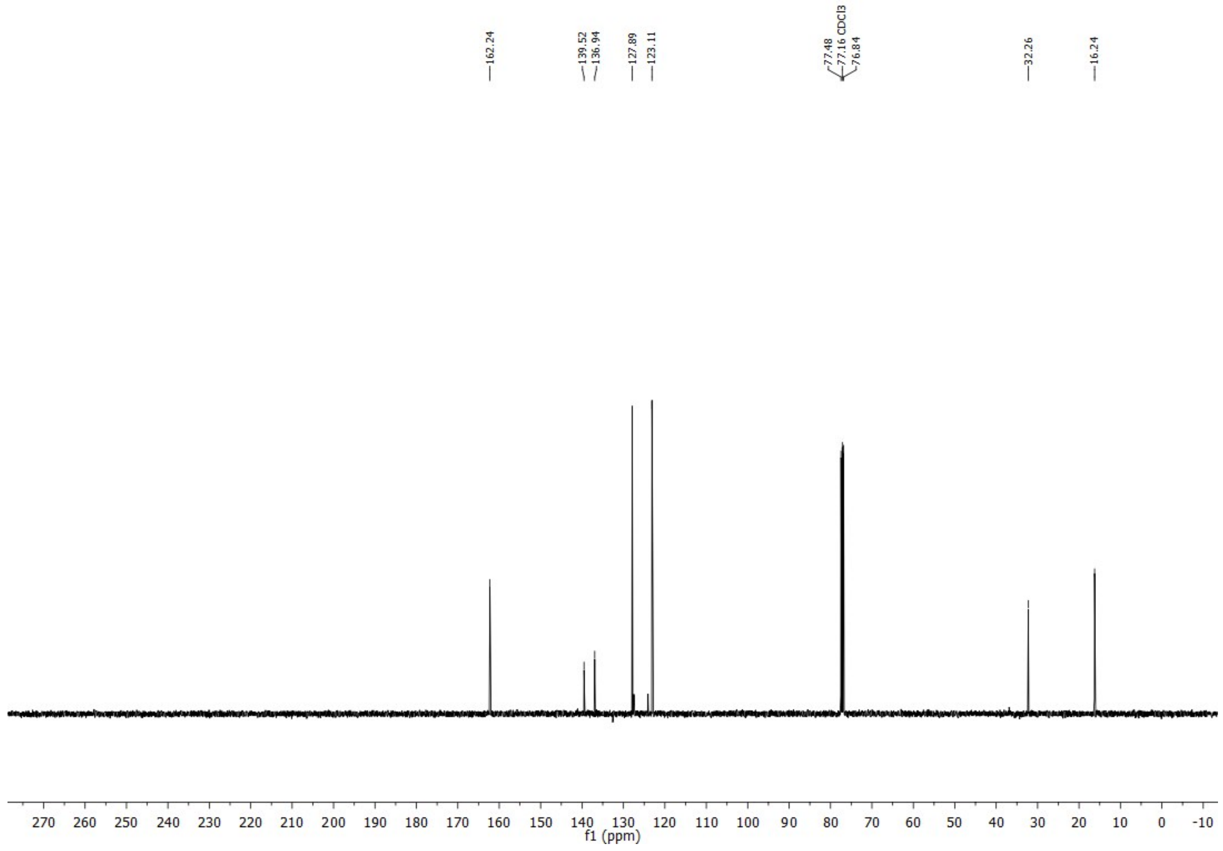
162.24
139.52
136.94
127.89
123.11
77.48
77.16 CDCl3
76.84
32.26
16.24
270 260 250 240 230 220 210 200 190 180 170 160 150 140 130 120 110 100 90 80 70 60 50 40 30 20 10 0 -10
f1 (ppm)

N-(4-(benzyloxy)phenyl)-*N*-methylformamide

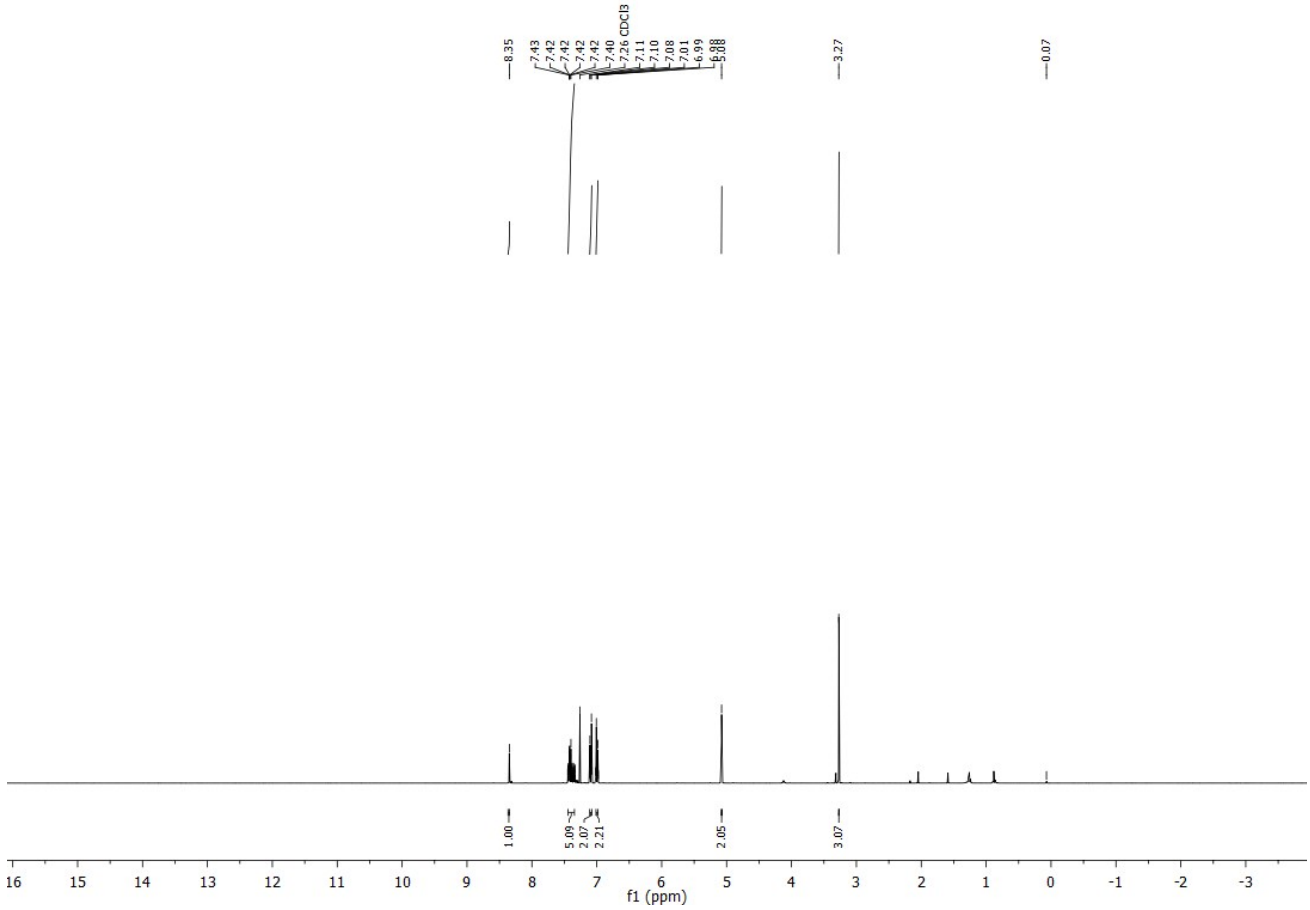

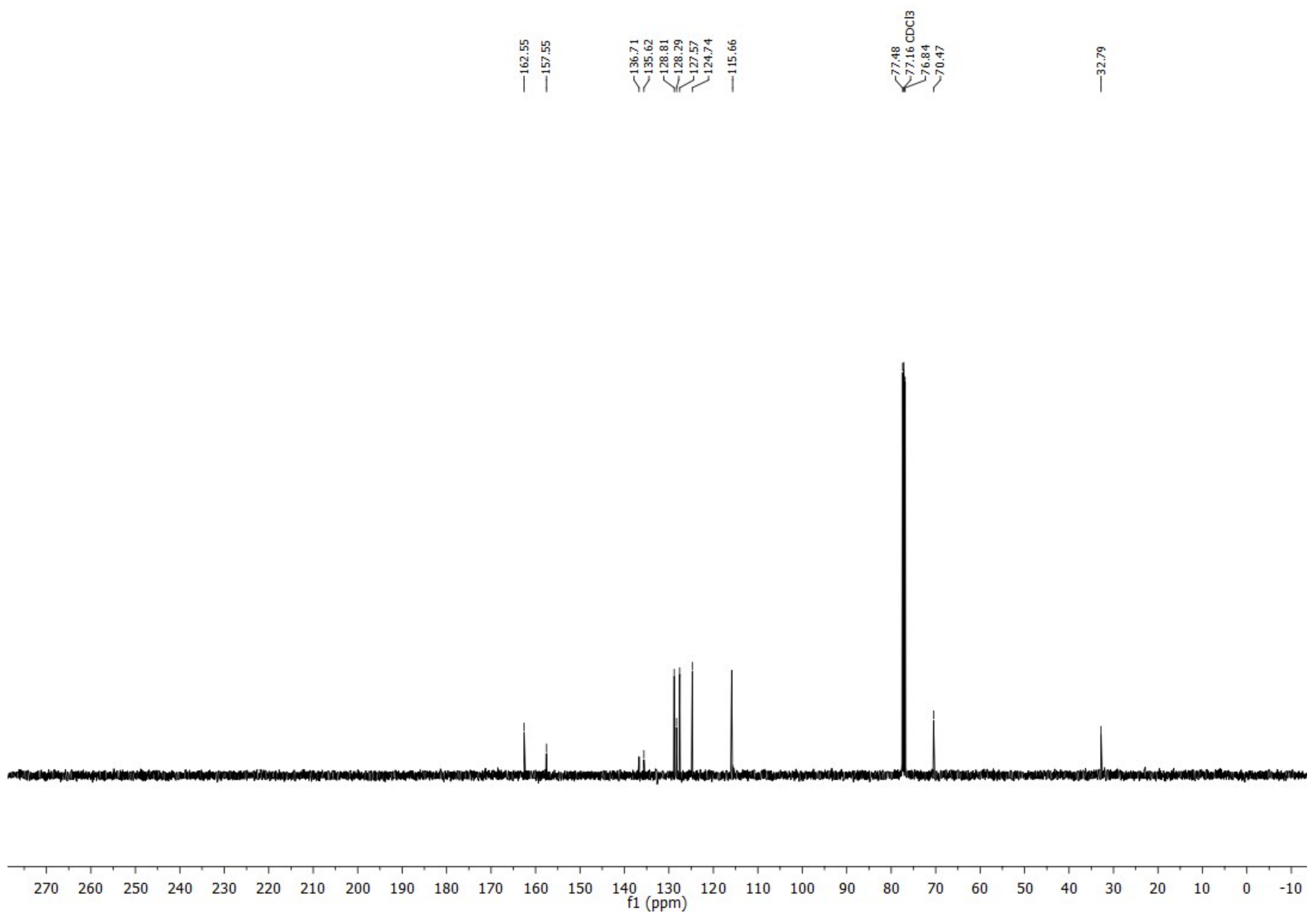
162.55
157.55
136.71
135.62
128.81
128.29
127.57
124.74
115.66
77.48
77.16 CDCl3
76.84
70.47
32.79
270 260 250 240 230 220 210 200 190 180 170 160 150 140 130 120 110 100 90 80 70 60 50 40 30 20 10 0 -10
f1 (ppm)

(*E*)-*N*-methyl-*N*-(4-styrylphenyl)formamide

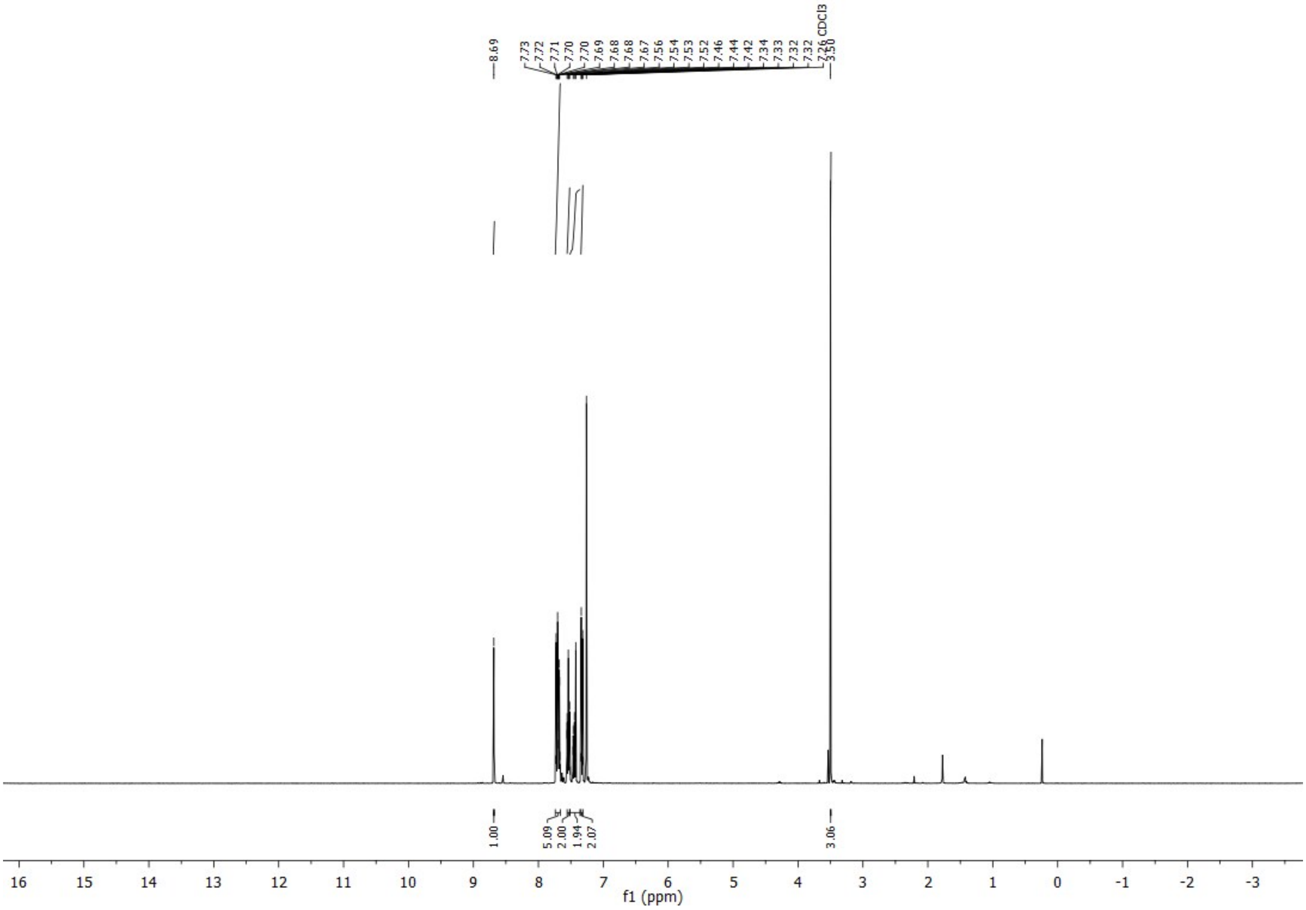

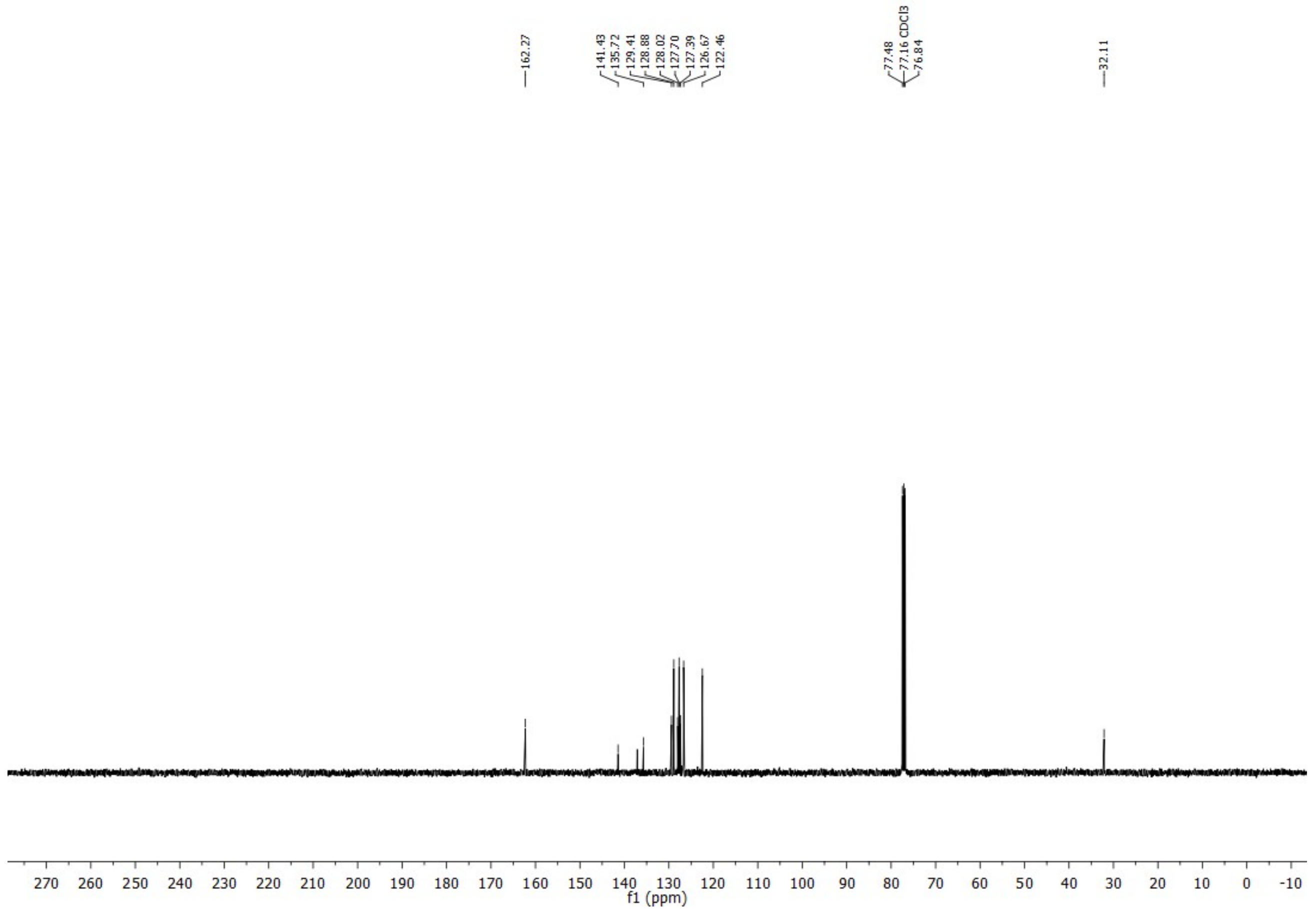
162.27
141.43
135.72
129.41
128.88
128.02
127.70
127.39
126.67
122.46
77.48
77.16 CDCl3
76.84
32.11
270 260 250 240 230 220 210 200 190 180 170 160 150 140 130 120 110 100 90 80 70 60 50 40 30 20 10 0 -10
f1 (ppm)

N-methyl-*N*-(3-(trifluoromethyl)phenyl)formamide

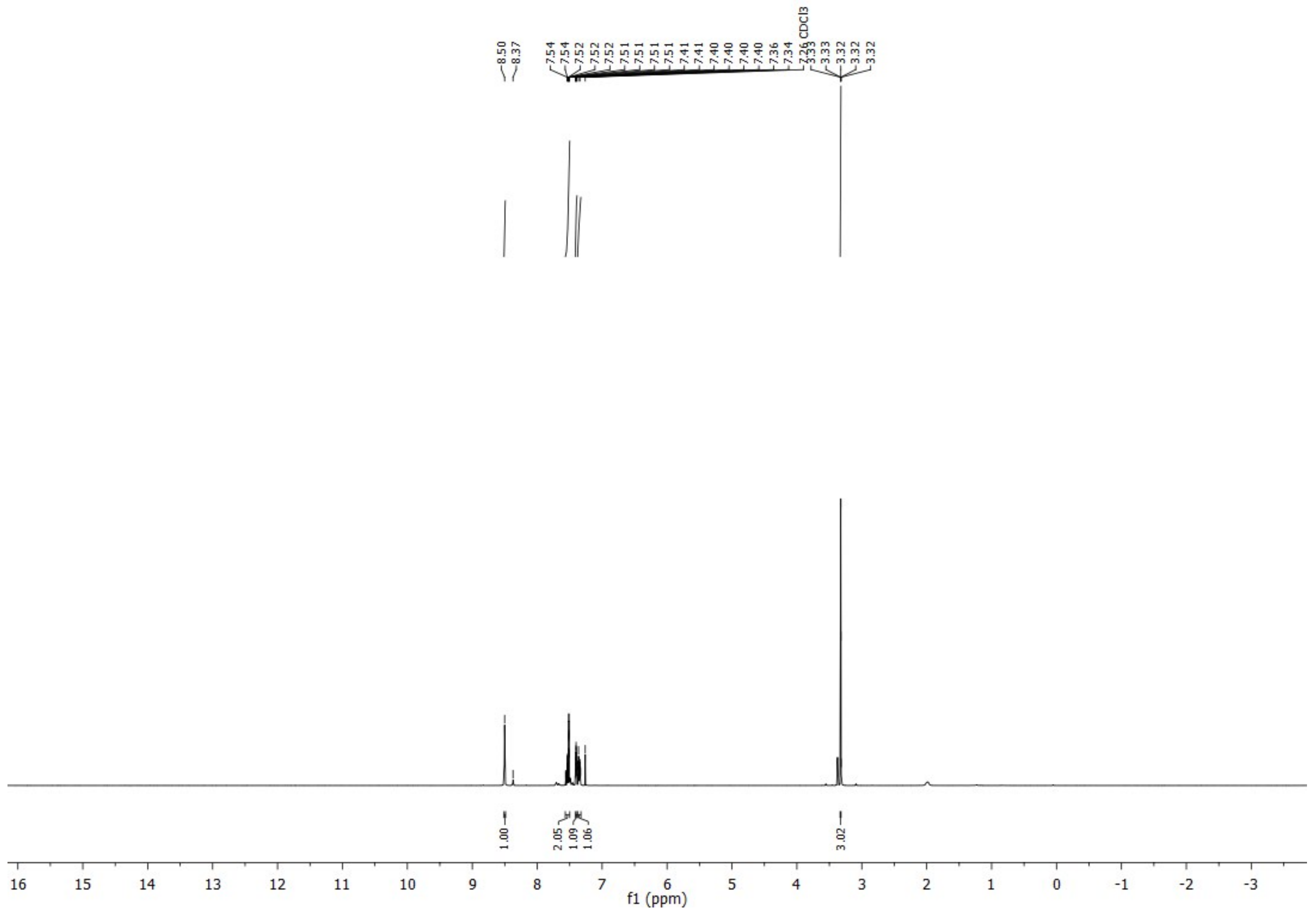

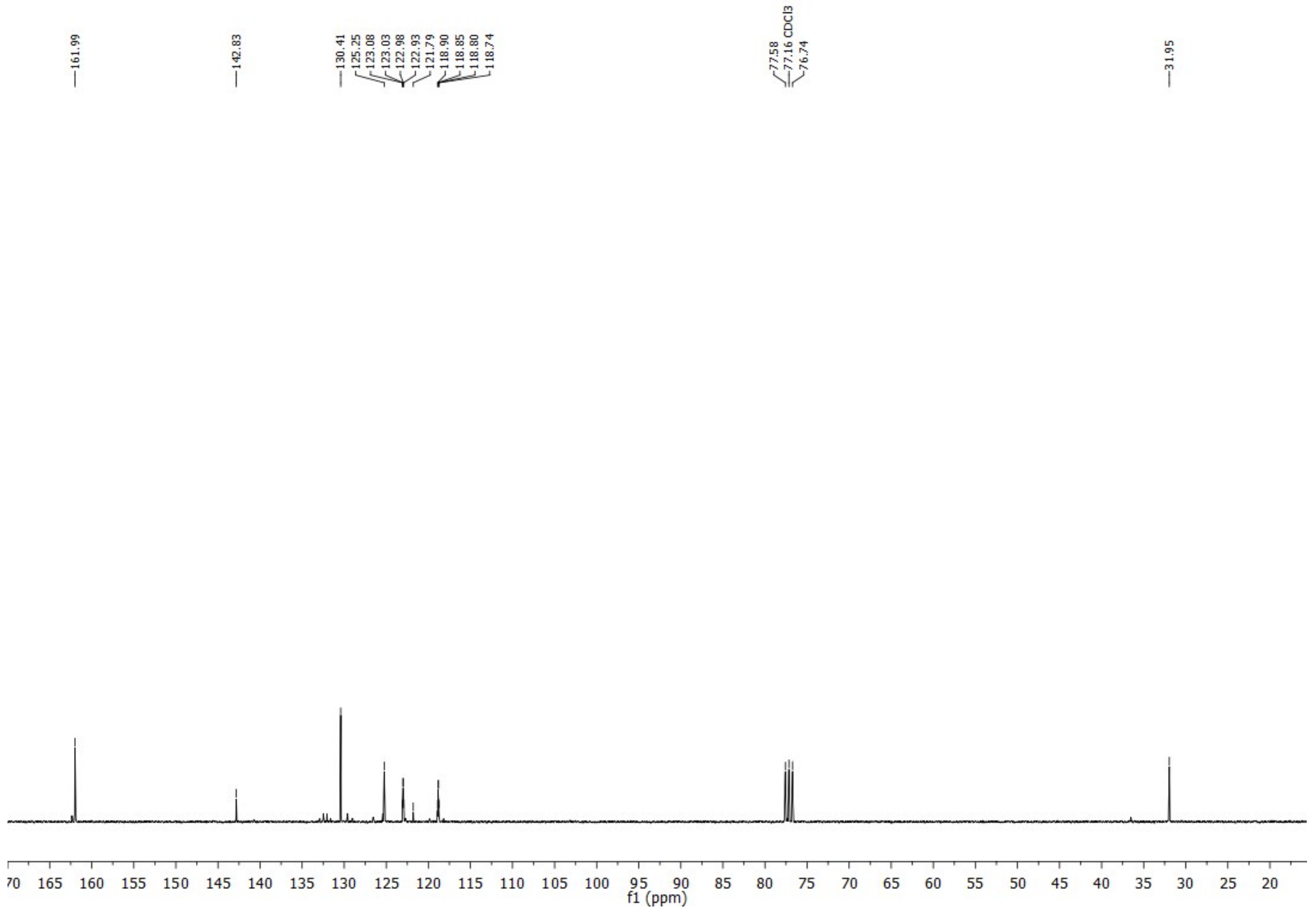

161.99
142.83
130.41
125.25
123.08
123.03
122.98
122.93
121.79
118.90
118.85
118.80
118.74
77.58
77.16 CDCl3
76.74
31.95
70 165 160 155 150 145 140 135 130 125 120 115 110 105 100 95 90 85 80 75 70 65 60 55 50 45 40 35 30 25 20
f1 (ppm)

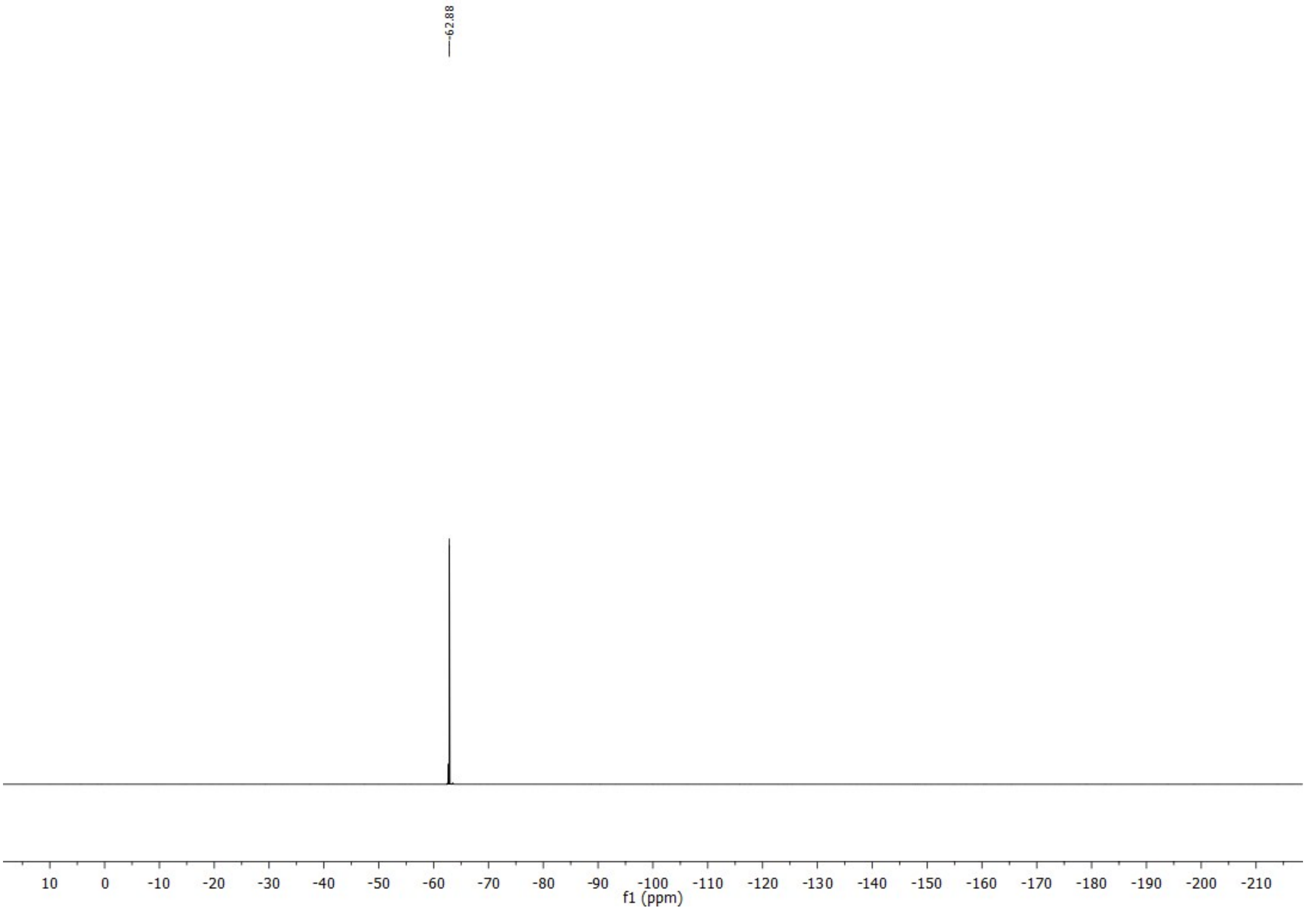
-62.88
10
0
-10
-20
-30
-40
-50
-60
-70
-80
-90
-100
-110
-120
-130
-140
-150
-160
-170
-180
-190
-200
-210
f1 (ppm)

N-(3-fluorophenyl)-*N*-methylformamide

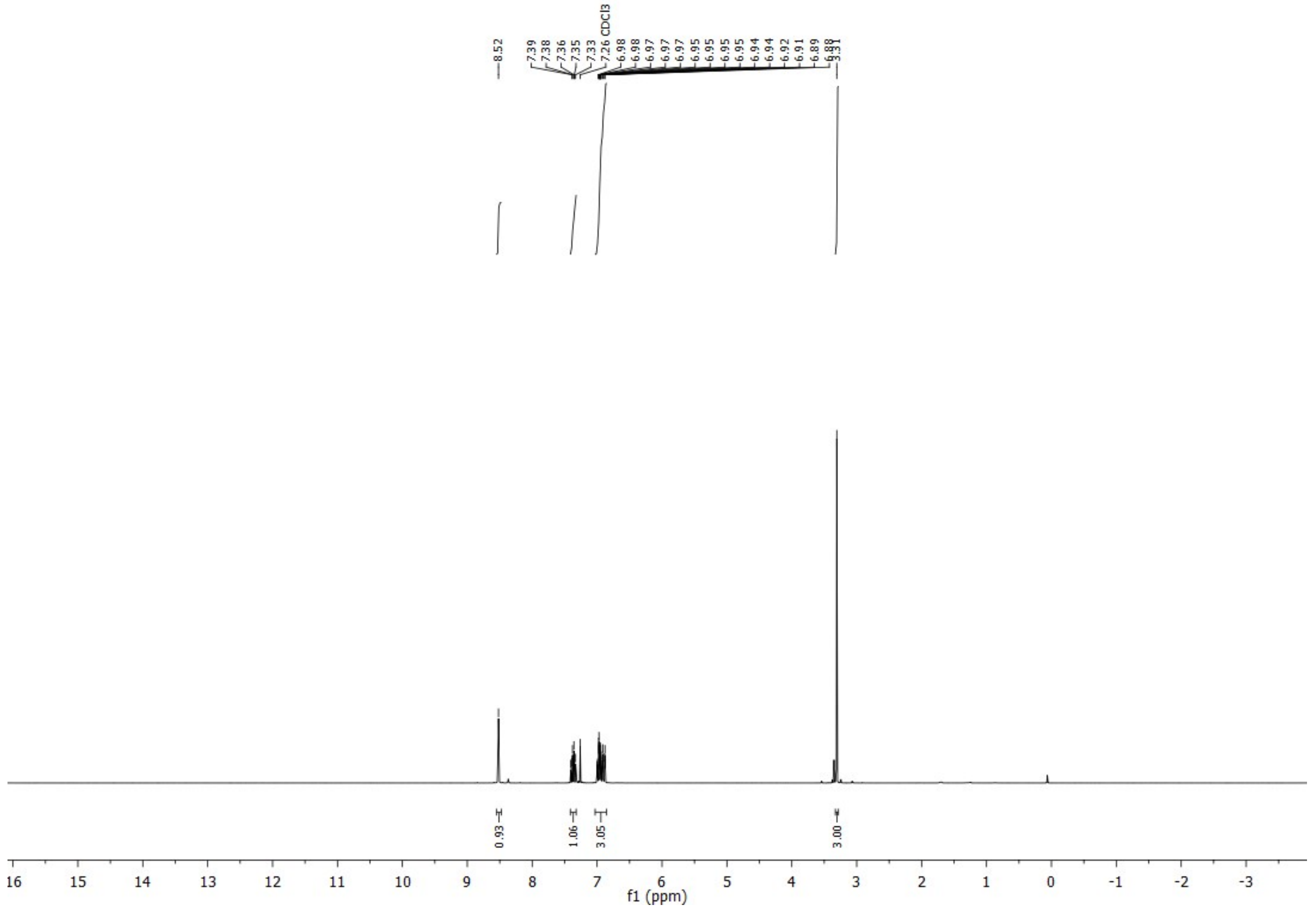

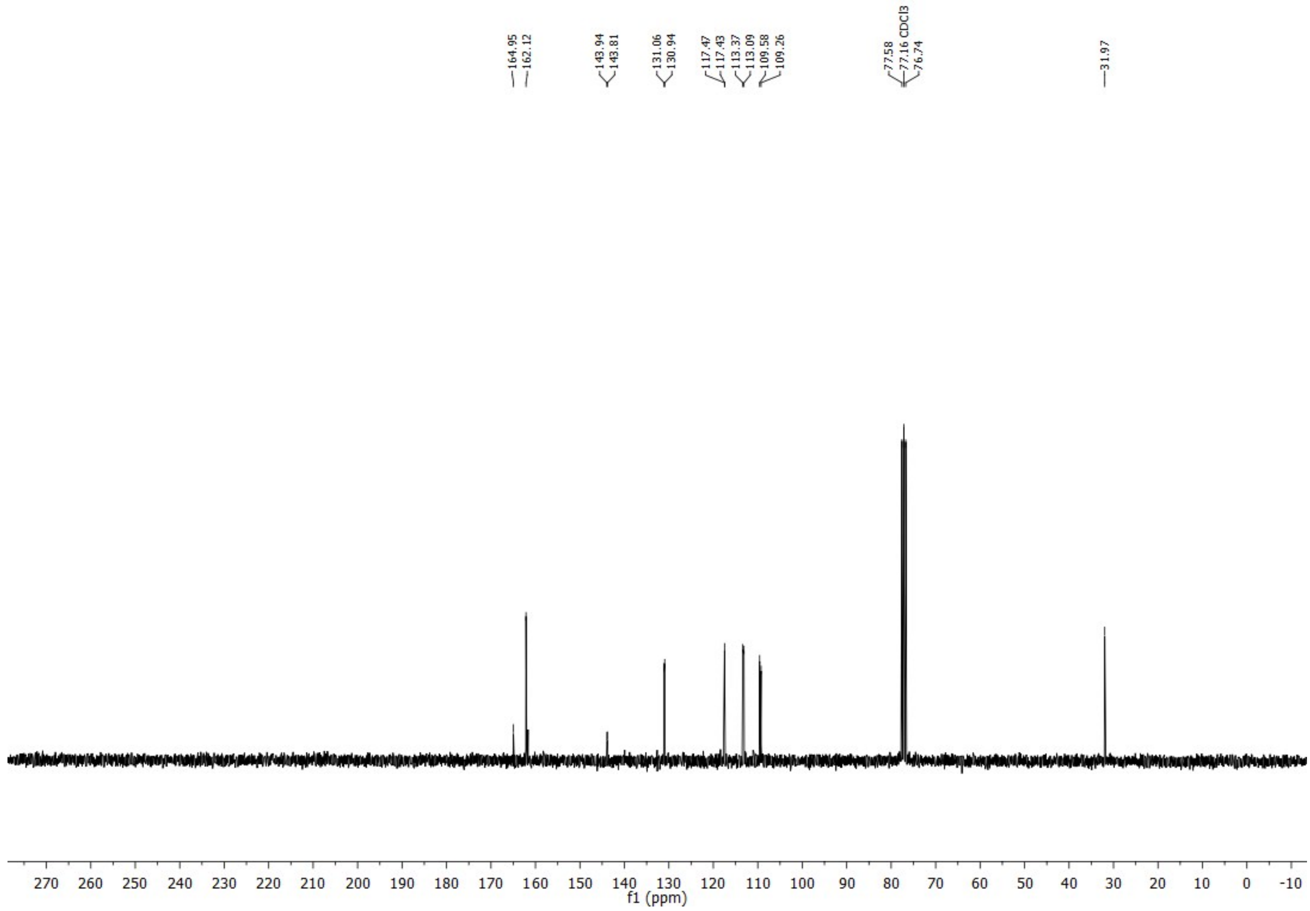
164.95
162.12
143.94
143.81
131.06
130.94
117.47
117.43
113.37
113.09
109.58
109.26
77.58
77.16 CDCl3
76.74
31.97
270 260 250 240 230 220 210 200 190 180 170 160 150 140 130 120 110 100 90 80 70 60 50 40 30 20 10 0 -10
f1 (ppm)

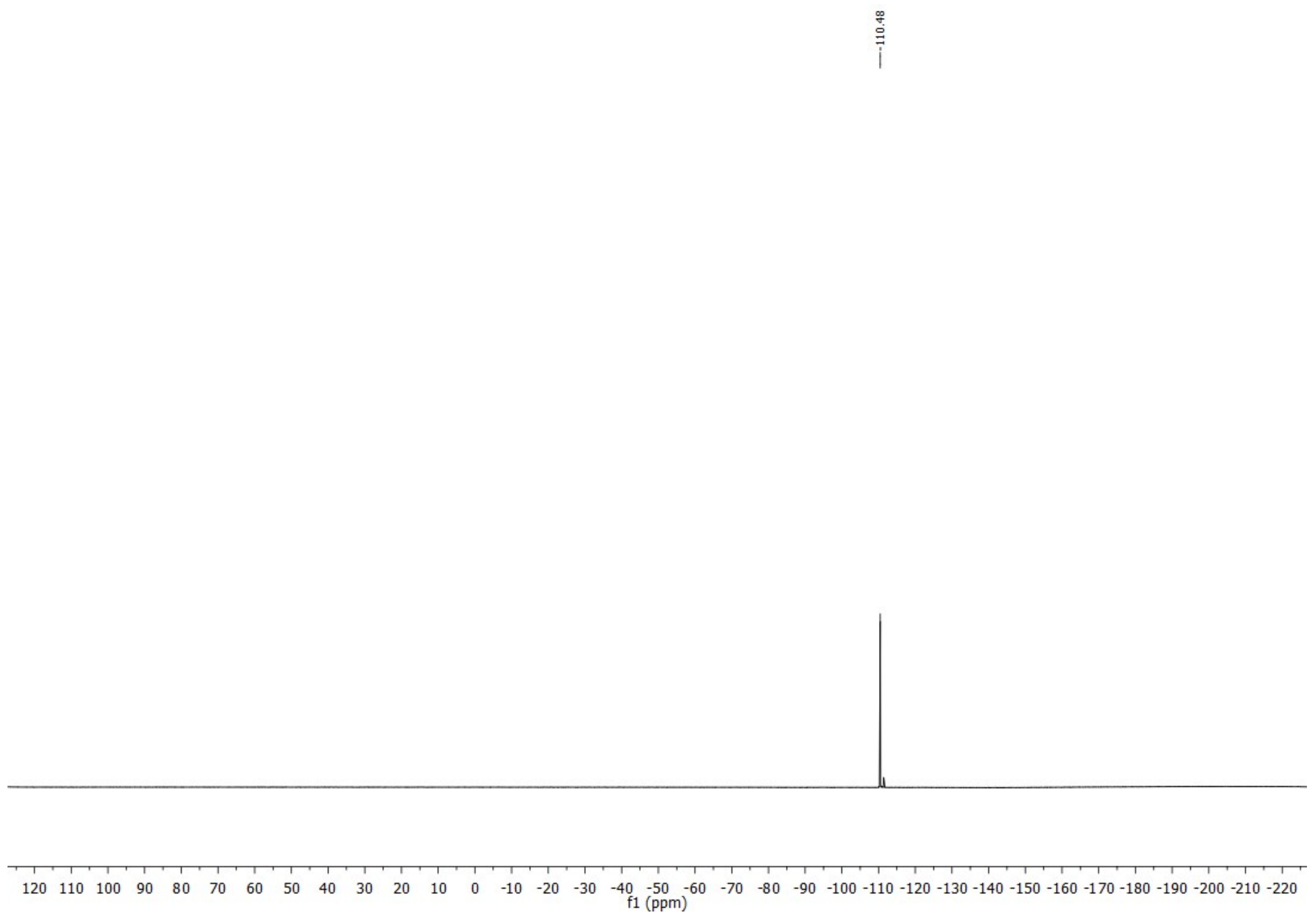
-110.48
120 110 100 90 80 70 60 50 40 30 20 10 0 -10 -20 -30 -40 -50 -60 -70 -80 -90 -100 -110 -120 -130 -140 -150 -160 -170 -180 -190 -200 -210 -220
f1 (ppm)

N-methyl-*N*-(4-(*N*-methylformamido)phenyl)benzamide

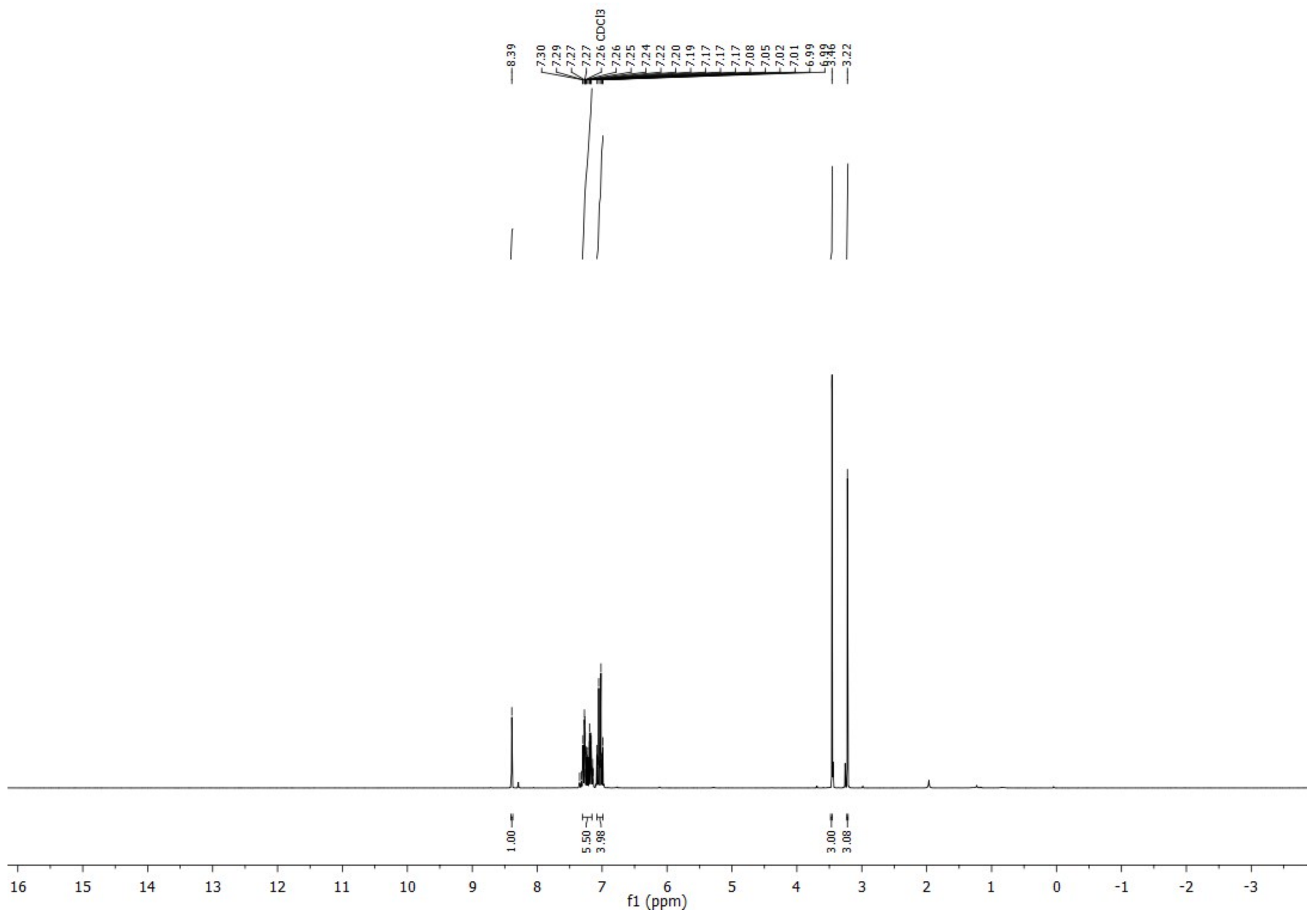

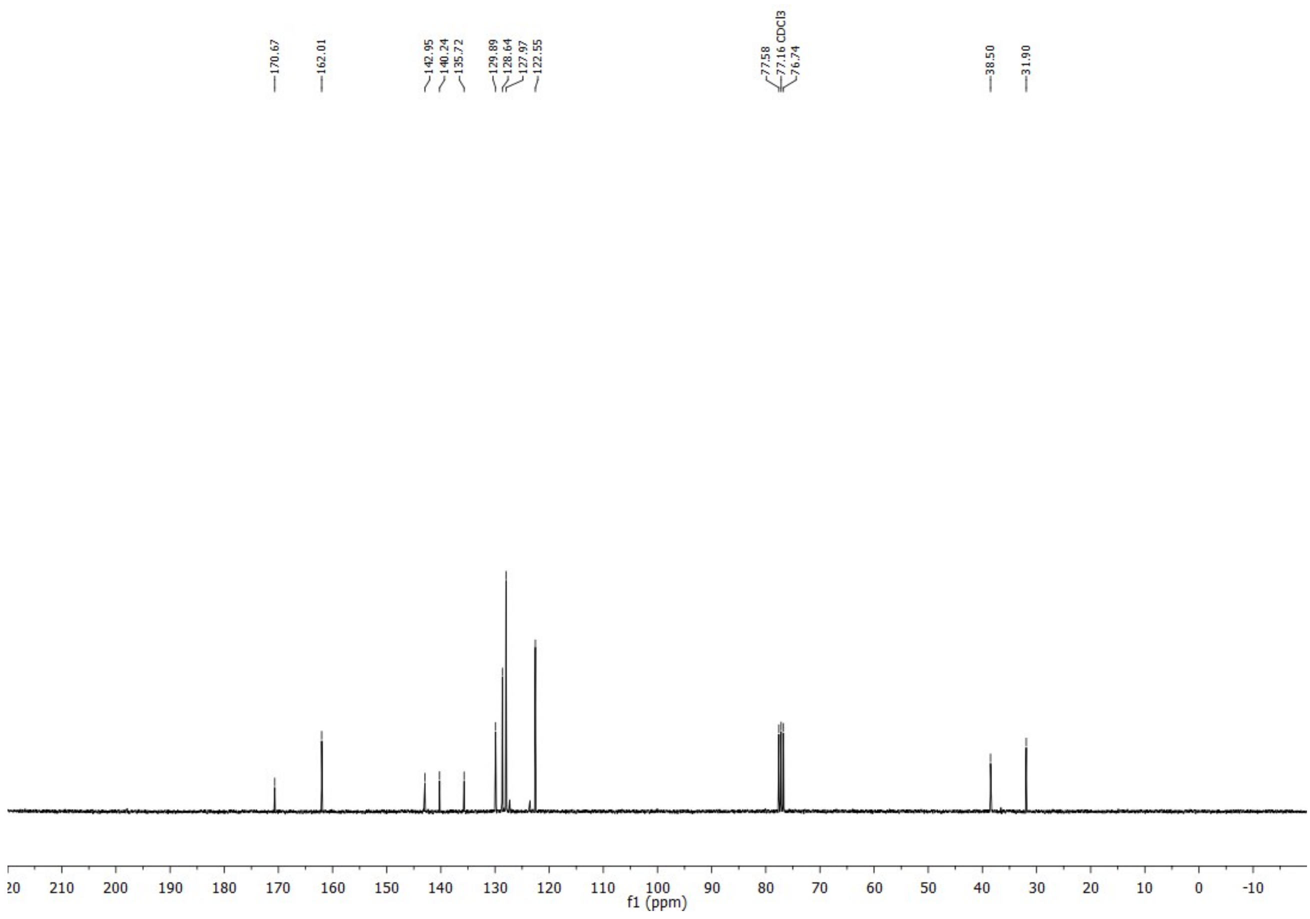

170.67
162.01
142.95
140.24
135.72
129.89
128.64
127.97
122.55
77.58
77.16 CDCl3
76.74
38.50
31.90
20
210
200
190
180
170
160
150
140
130
120
110
100
90
80
70
60
50
40
30
20
10
0
-10
f1 (ppm)

NMR spectra of the isolated products of the catalysis

N-Methylaniline

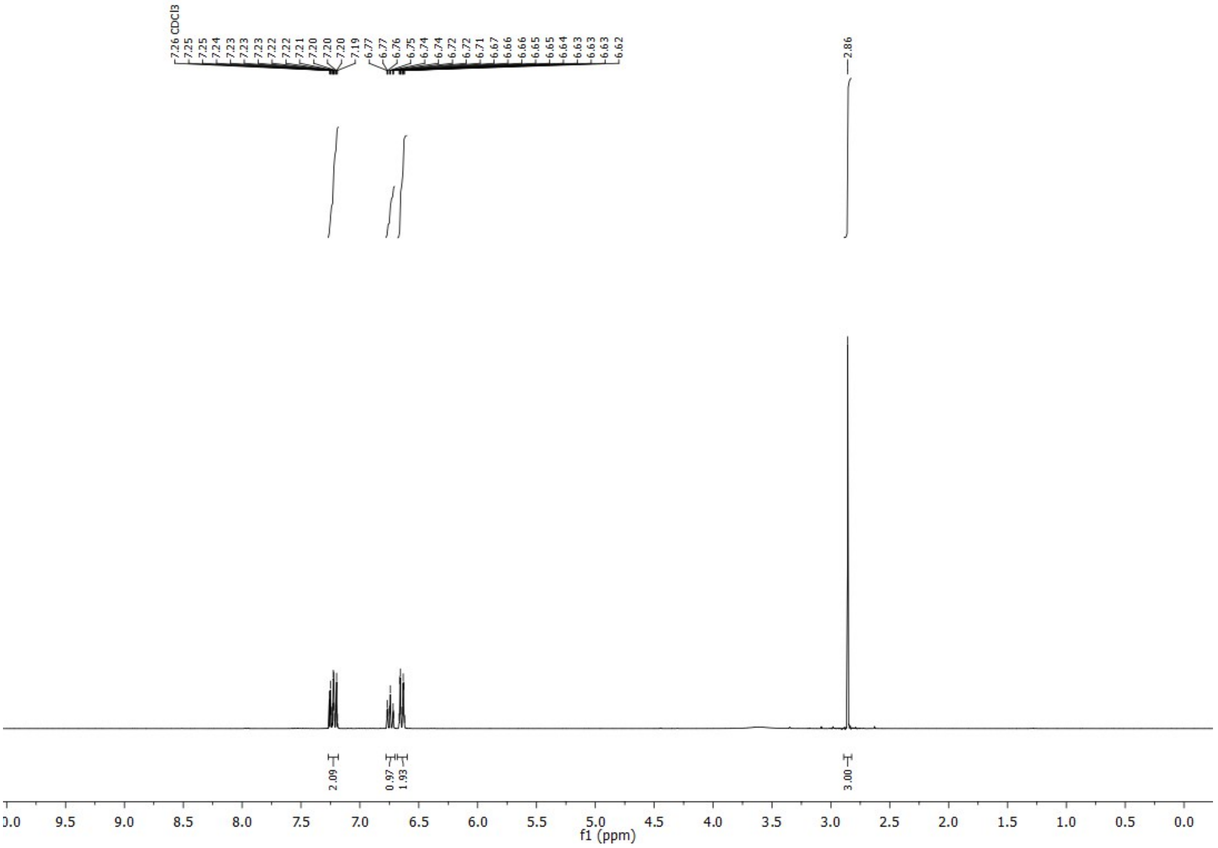

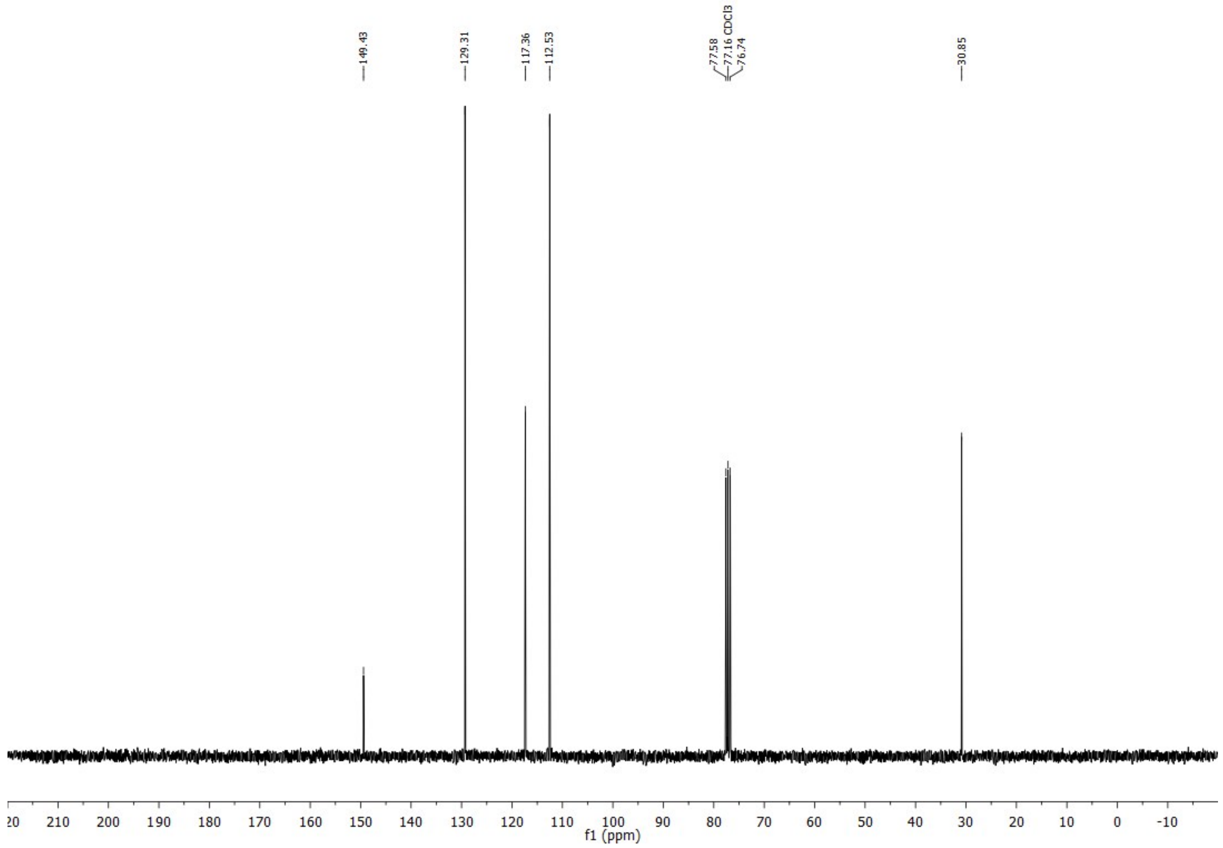
149.43
129.31
117.36
112.53
77.58
77.16 CDCl3
76.74
30.85
f1 (ppm)

4-Fluoro-*N*-methylaniline

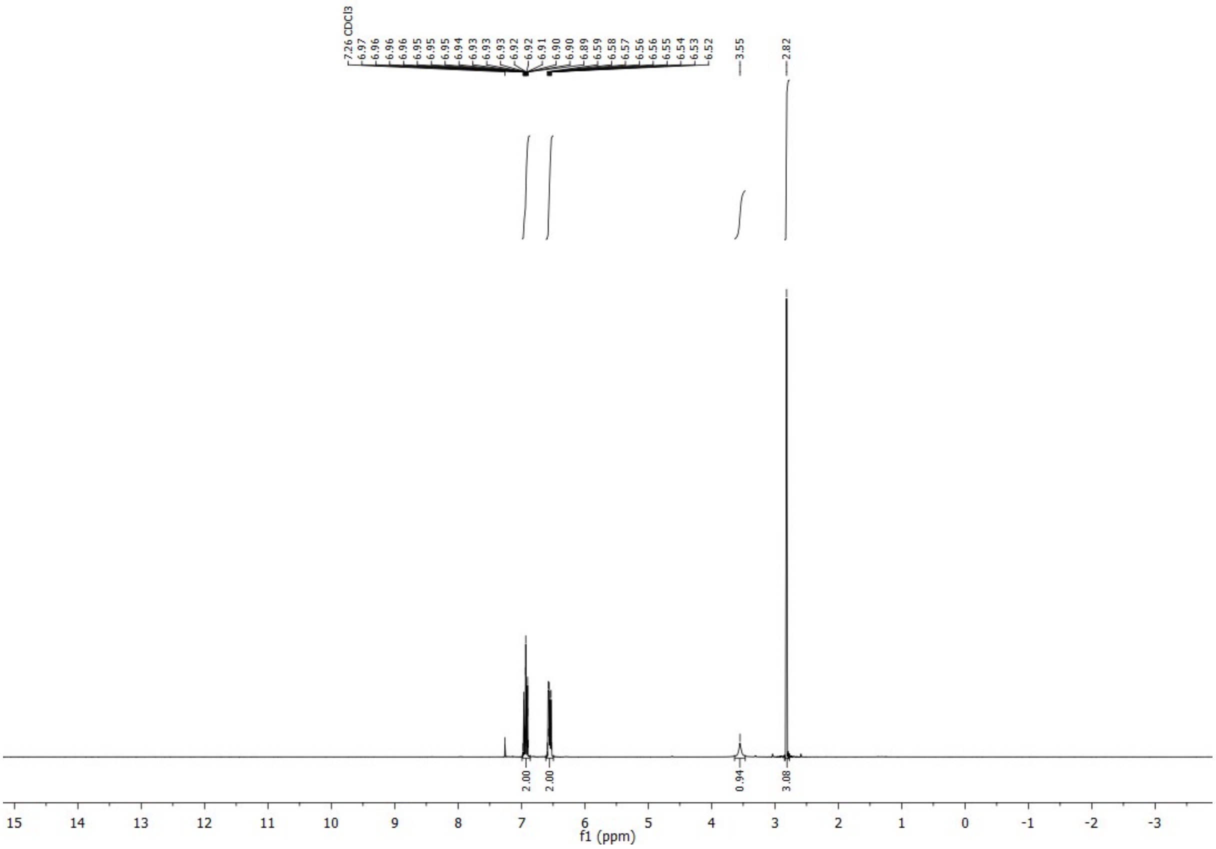

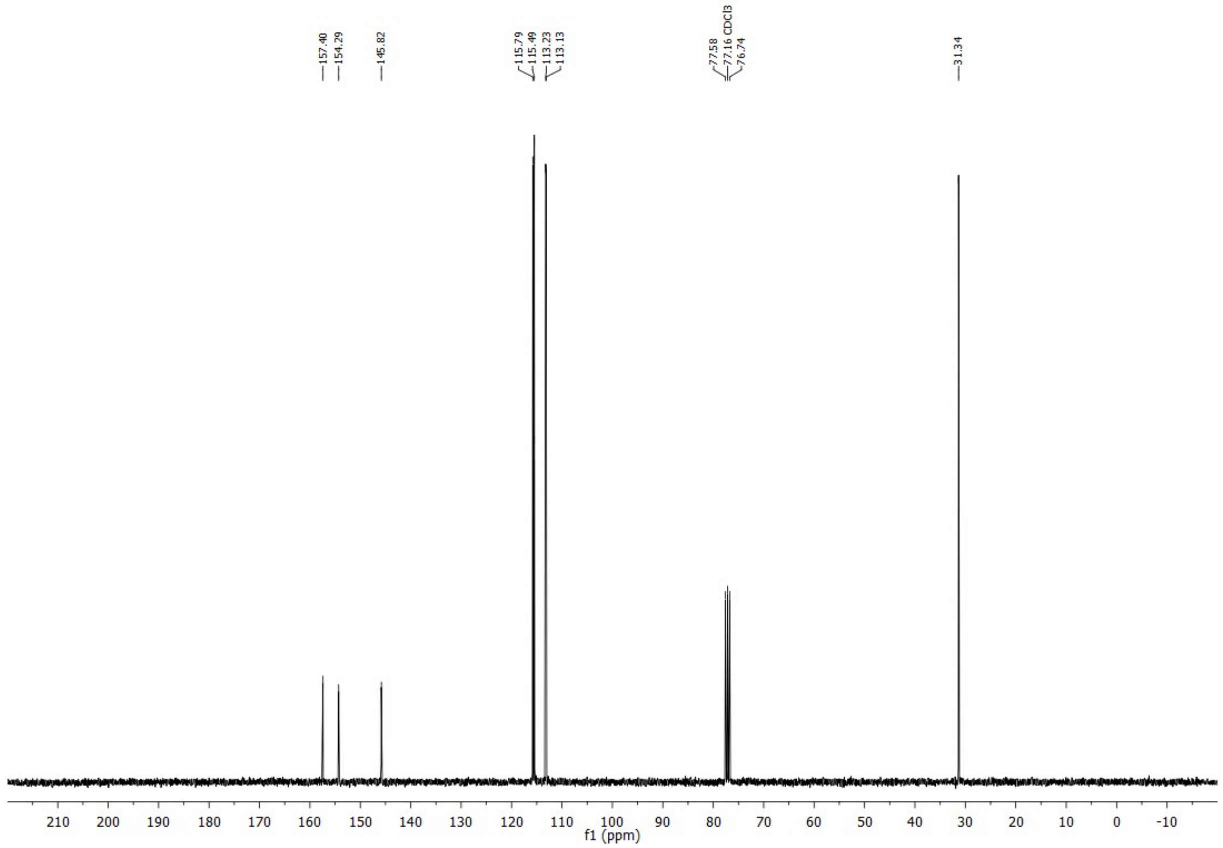
157.40
154.29
145.82
115.79
115.49
113.23
113.13
77.58
77.16 CDCl3
76.74
31.34
210 200 190 180 170 160 150 140 130 120 110 100 90 80 70 60 50 40 30 20 10 0 -10
f1 (ppm)

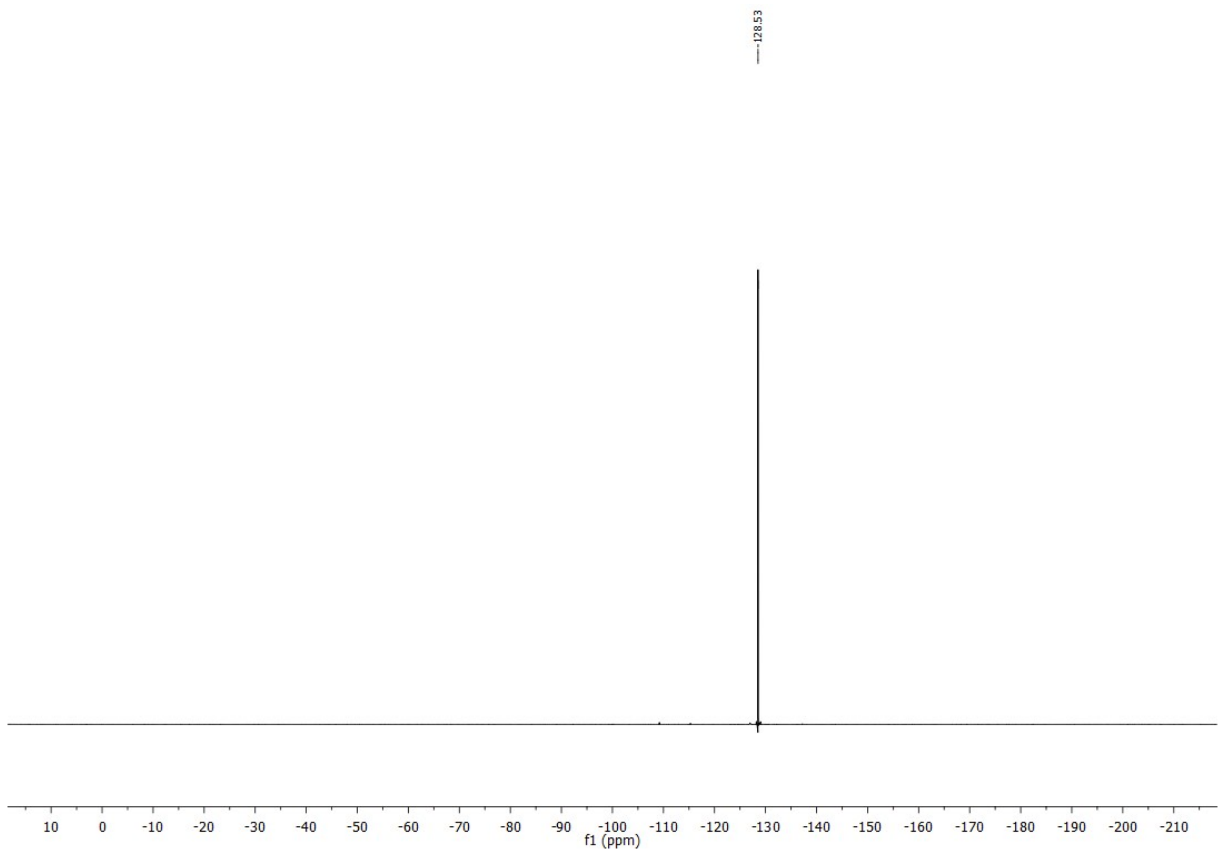
-128.53
10 0 -10 -20 -30 -40 -50 -60 -70 -80 -90 -100 -110 -120 -130 -140 -150 -160 -170 -180 -190 -200 -210
f1 (ppm)

N,4-dimethylaniline

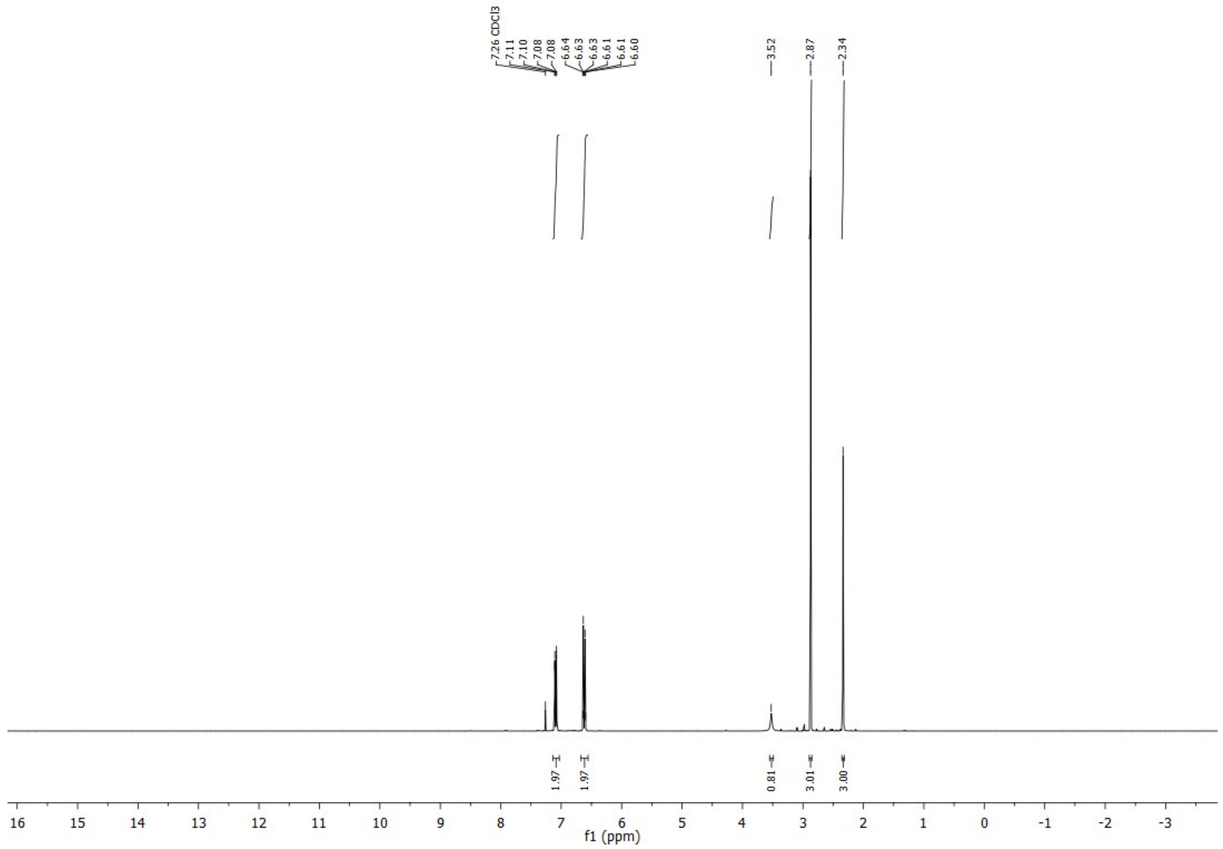

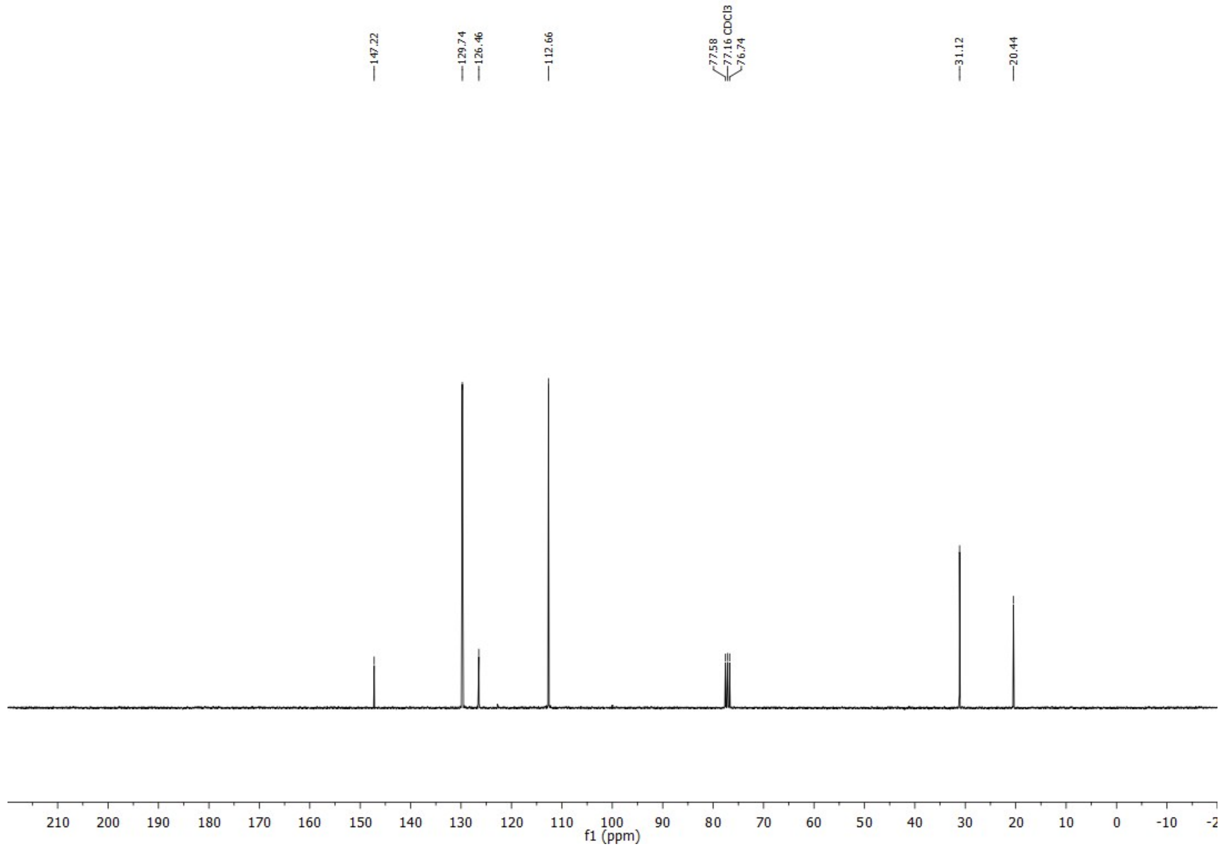
147.22
129.74
126.46
112.66
77.58
77.16 CDCl3
76.74
31.12
20.44
210 200 190 180 170 160 150 140 130 120 110 100 90 80 70 60 50 40 30 20 10 0 -10
f1 (ppm)

4-methoxy-*N*-methylaniline

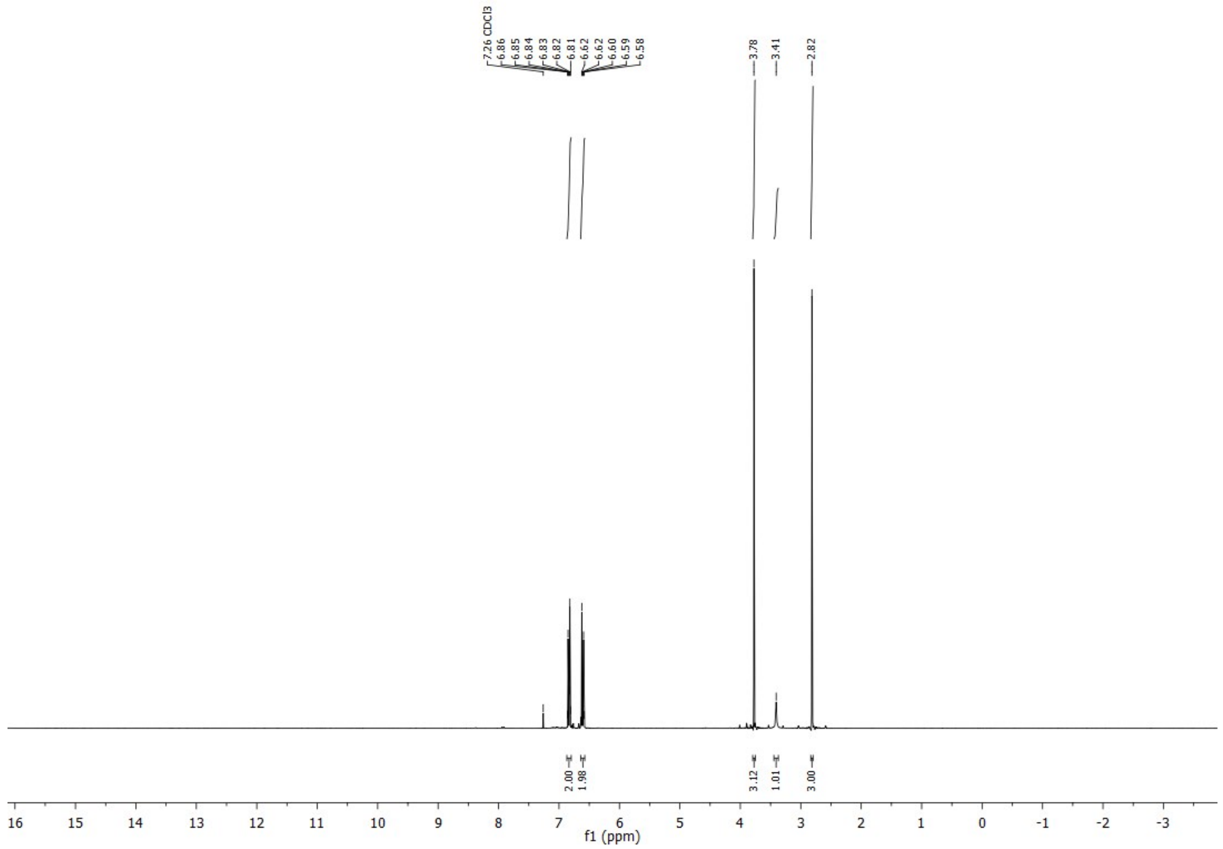

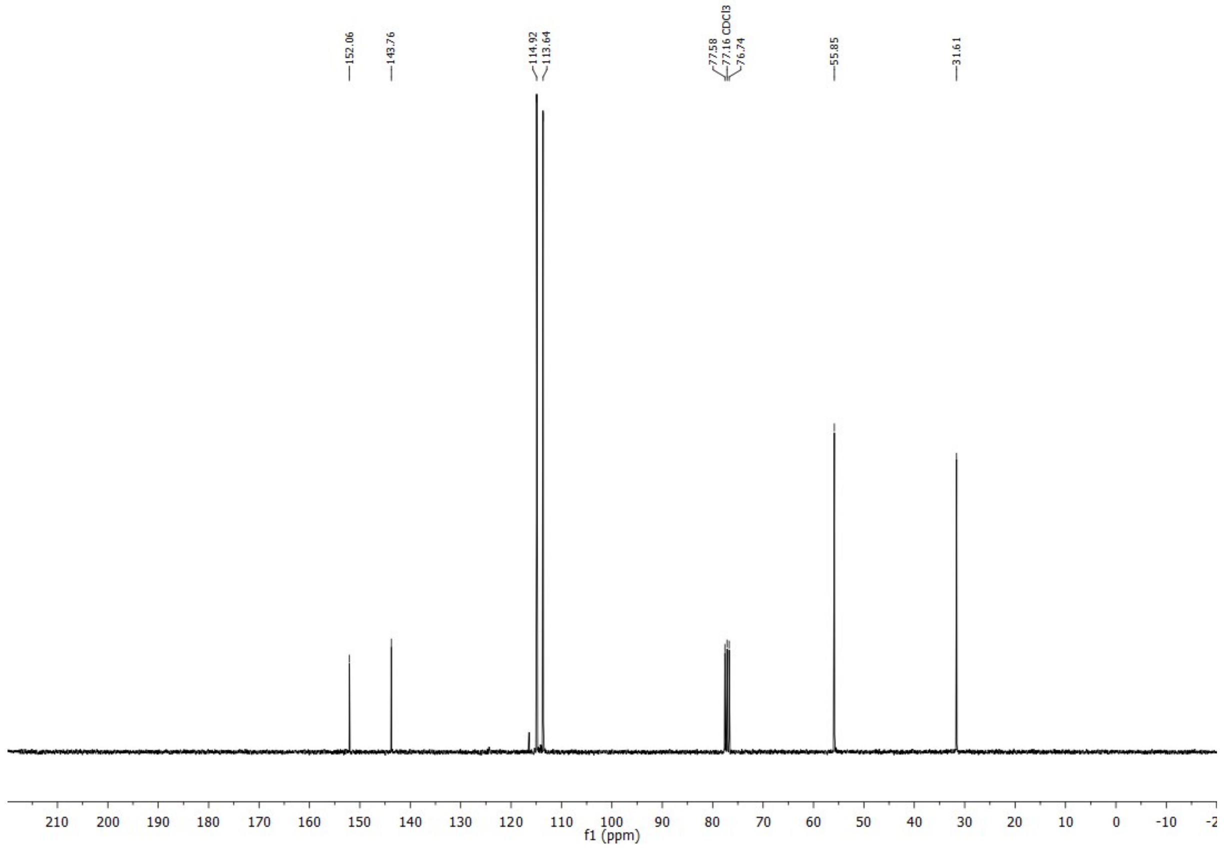
152.06
143.76
114.92
113.64
77.58
77.16 CDCl3
76.74
55.85
31.61
210 200 190 180 170 160 150 140 130 120 110 100 90 80 70 60 50 40 30 20 10 0 -10
f1 (ppm)

N-methyl-4-(trifluoromethyl)aniline

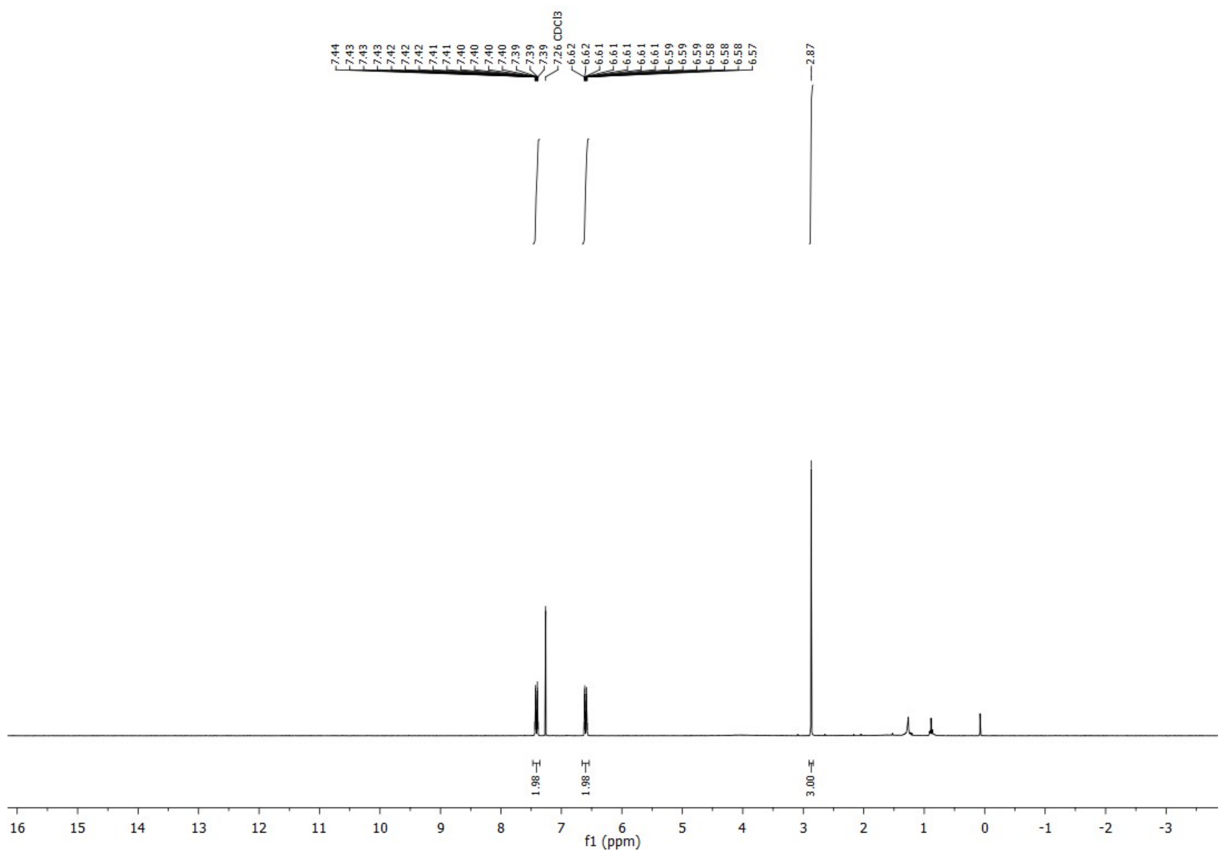

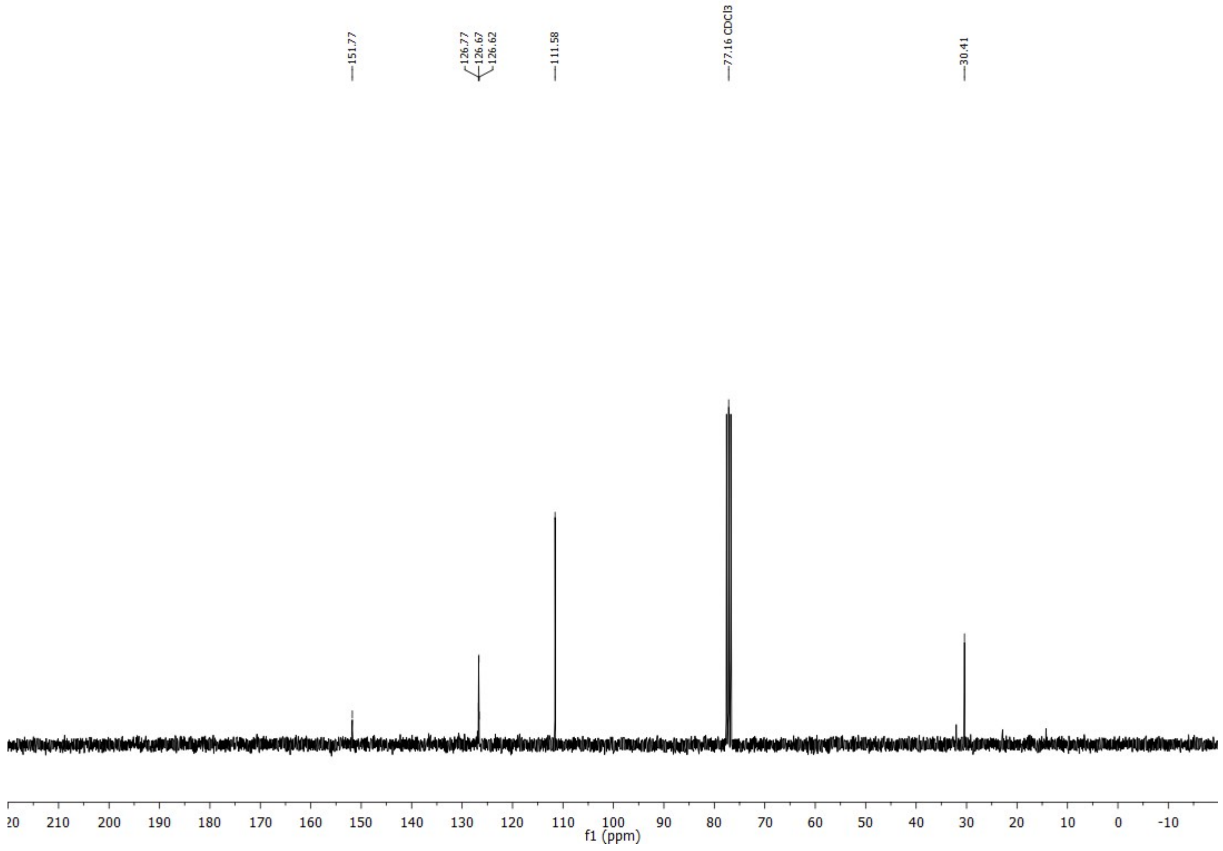
151.77
126.77
126.67
126.62
111.58
77.16 CDCl3
30.41
f1 (ppm)

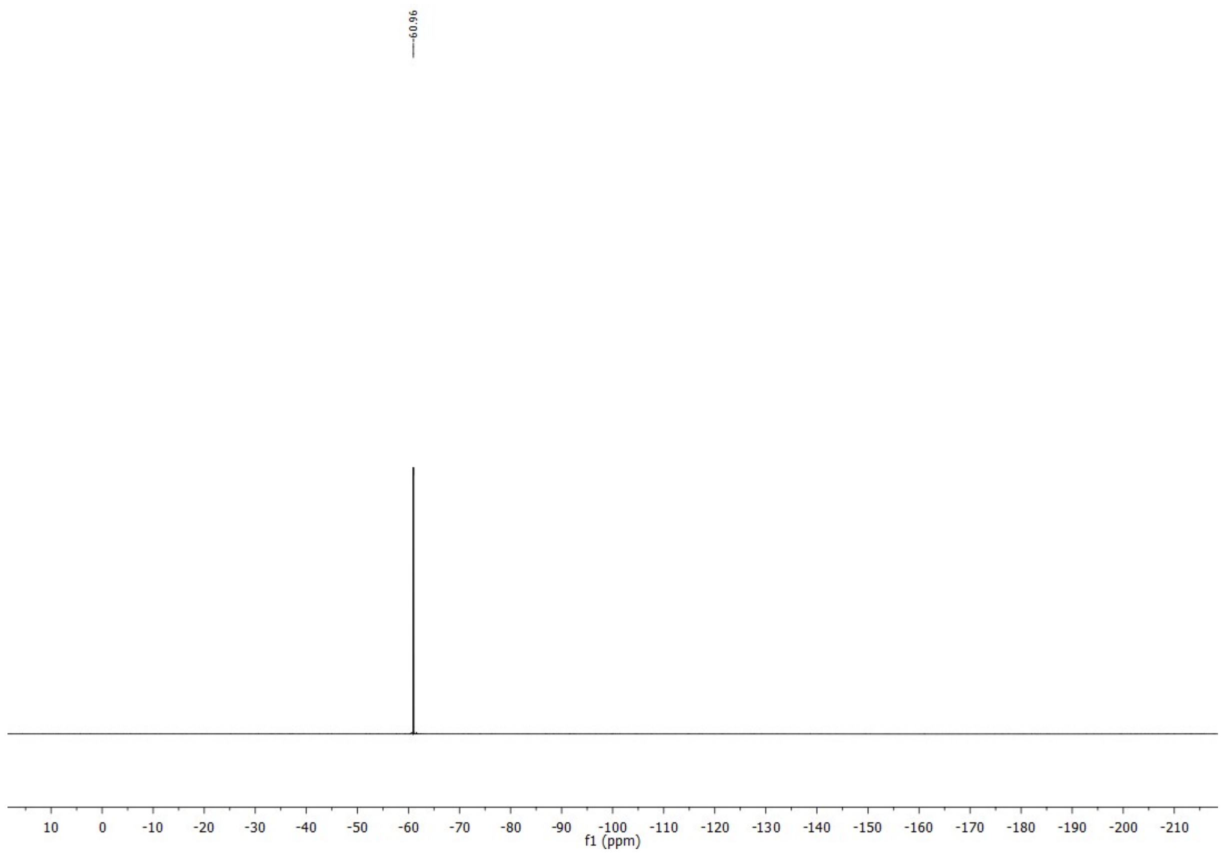
-60.96
10 0 -10 -20 -30 -40 -50 -60 -70 -80 -90 -100 -110 -120 -130 -140 -150 -160 -170 -180 -190 -200 -210
f1 (ppm)

Methyl-4-(methylamino)benzoate

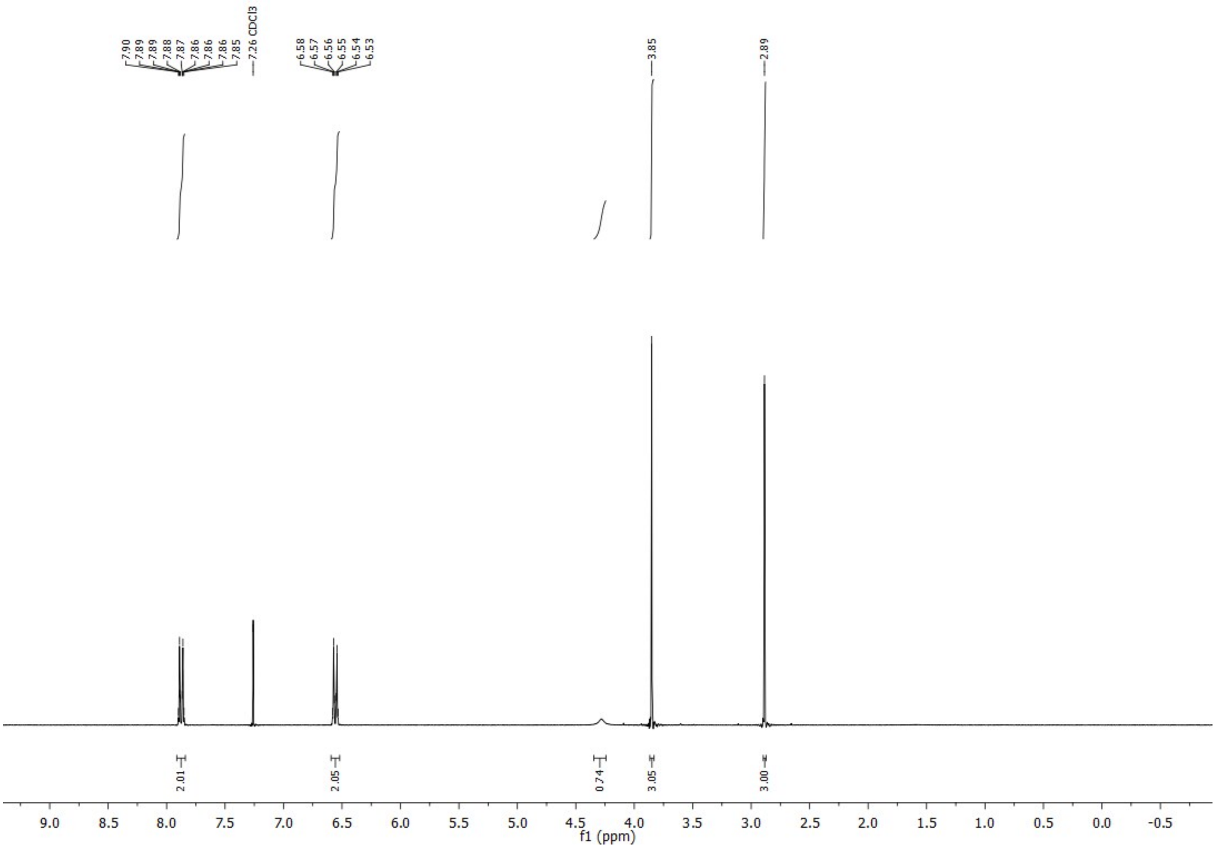

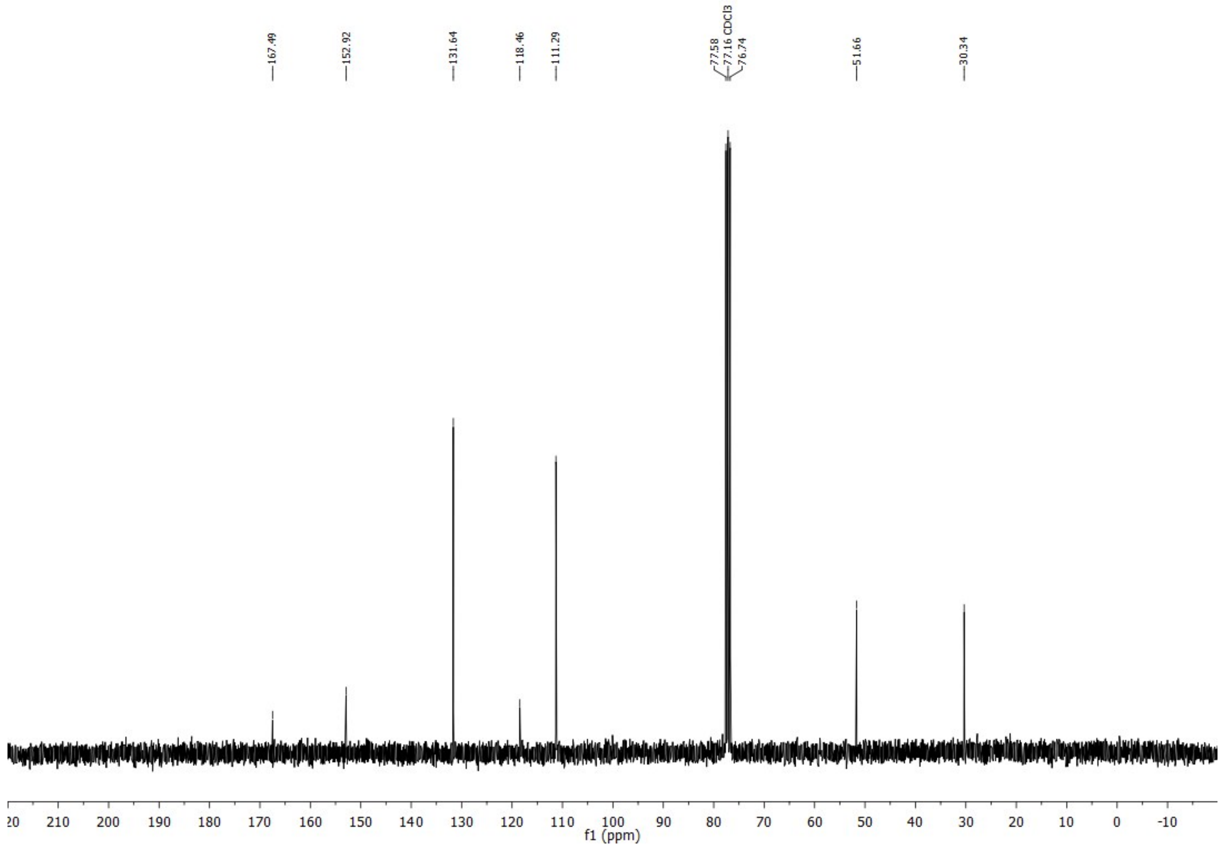

167.49
152.92
131.64
118.46
111.29
77.58
77.16 CDCl3
76.74
51.66
30.34
20 210 200 190 180 170 160 150 140 130 120 110 100 90 80 70 60 50 40 30 20 10 0 -10
f1 (ppm)

N-methyl-3-methoxyaniline

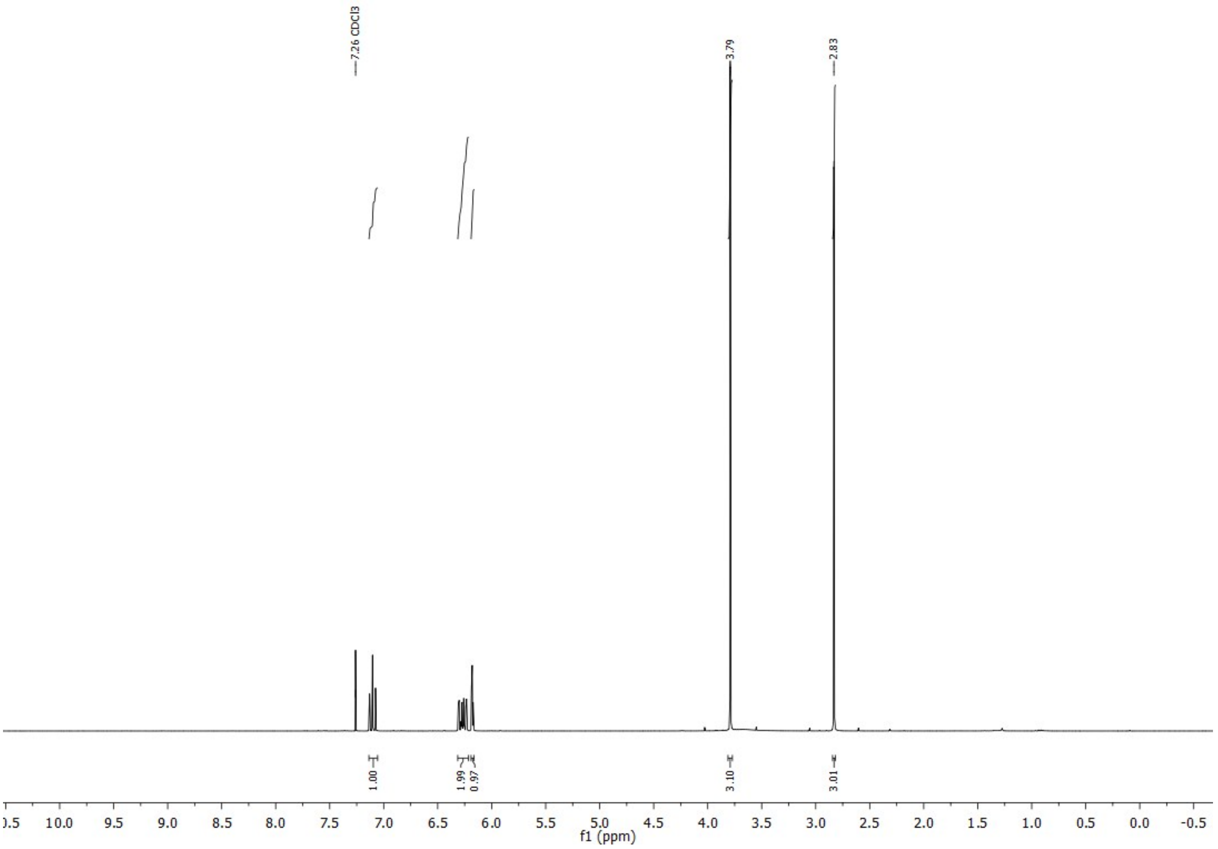

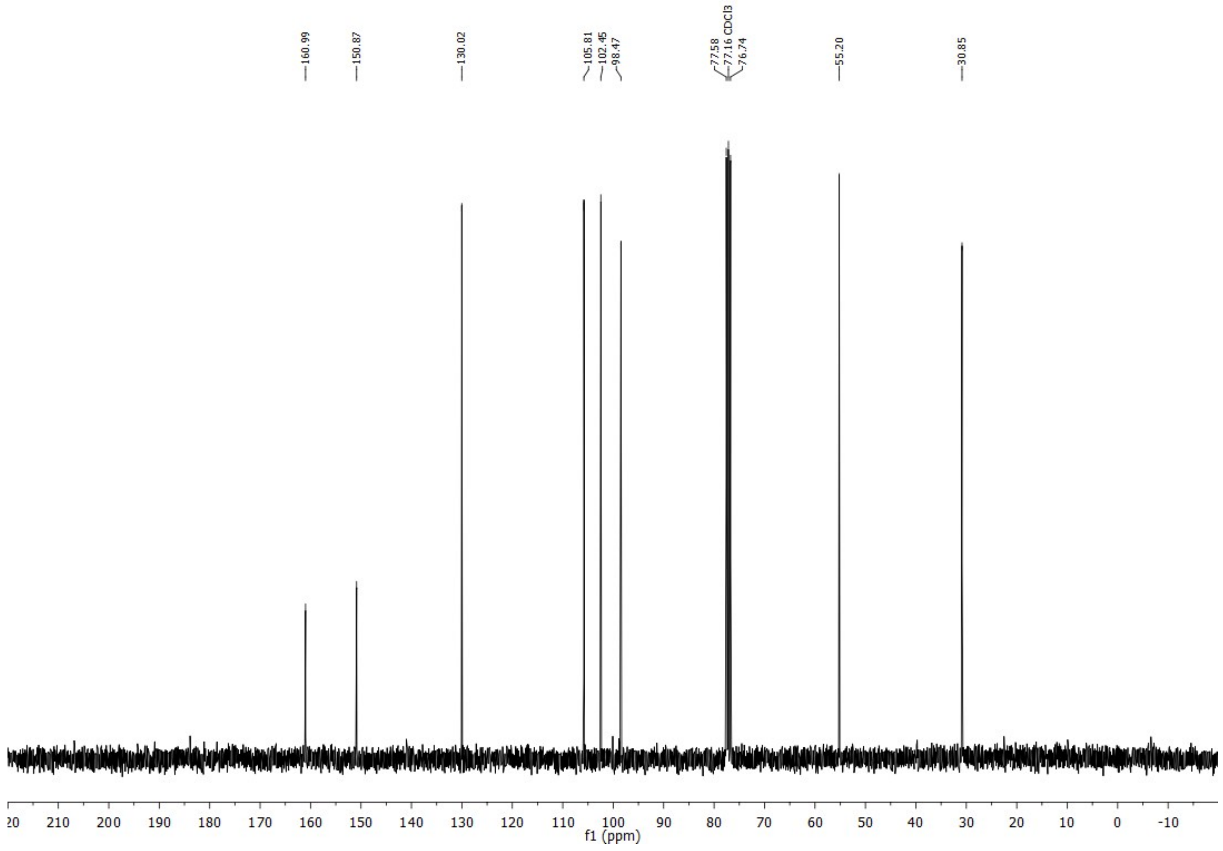
160.99
150.87
130.02
105.81
102.45
98.47
77.58
77.16 CDCl3
76.74
55.20
30.85
f1 (ppm)

N-methyl-3-(trifluoromethyl)aniline

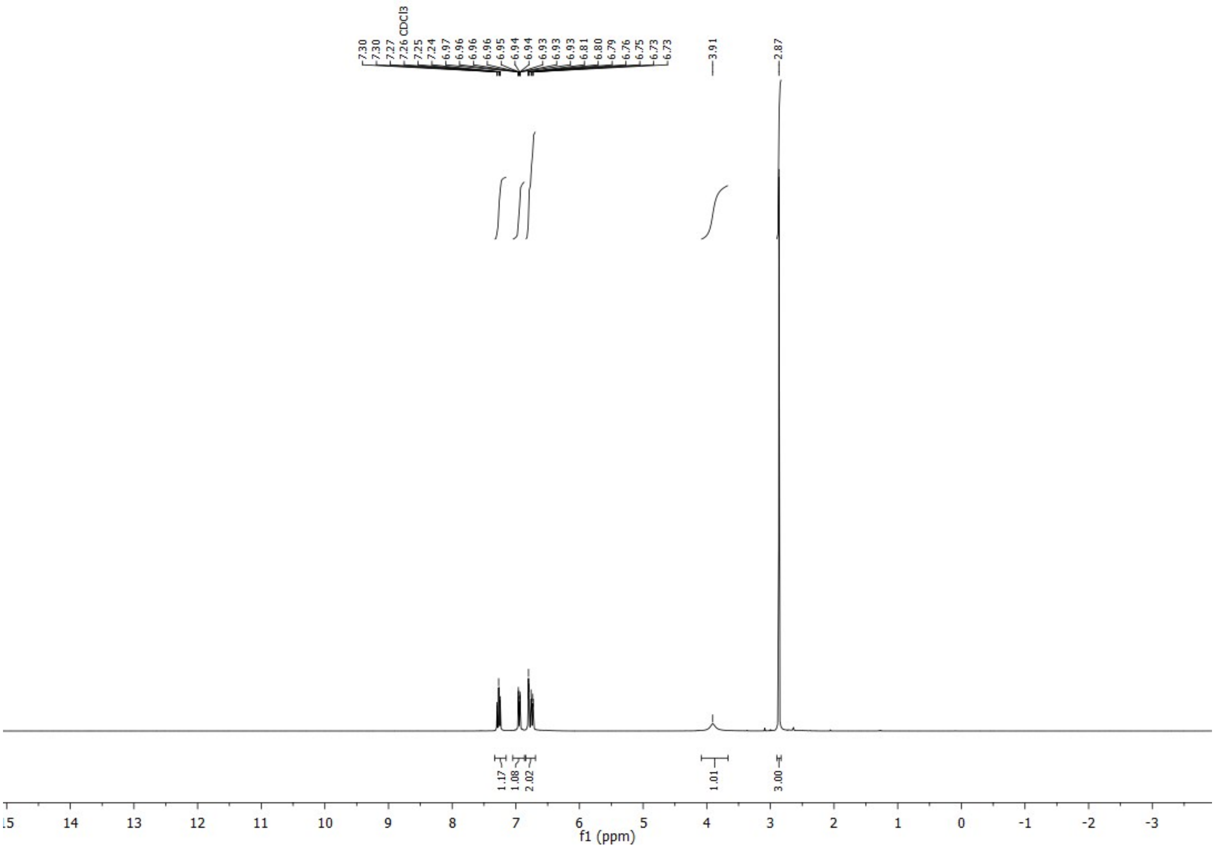

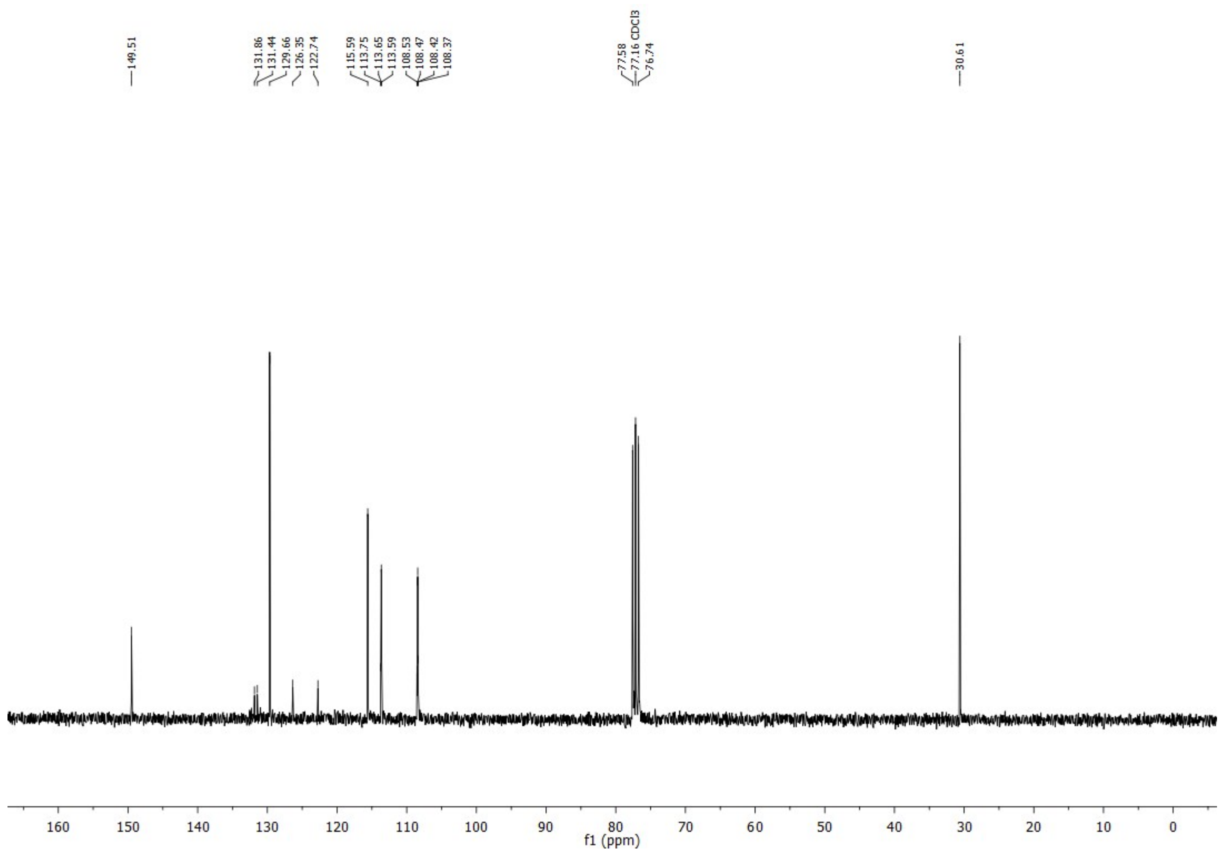
149.51
131.86
131.44
129.66
126.35
122.74
115.59
113.75
113.65
113.59
108.53
108.47
108.42
108.37
77.58
77.16 CDCl3
76.74
30.61
f1 (ppm)

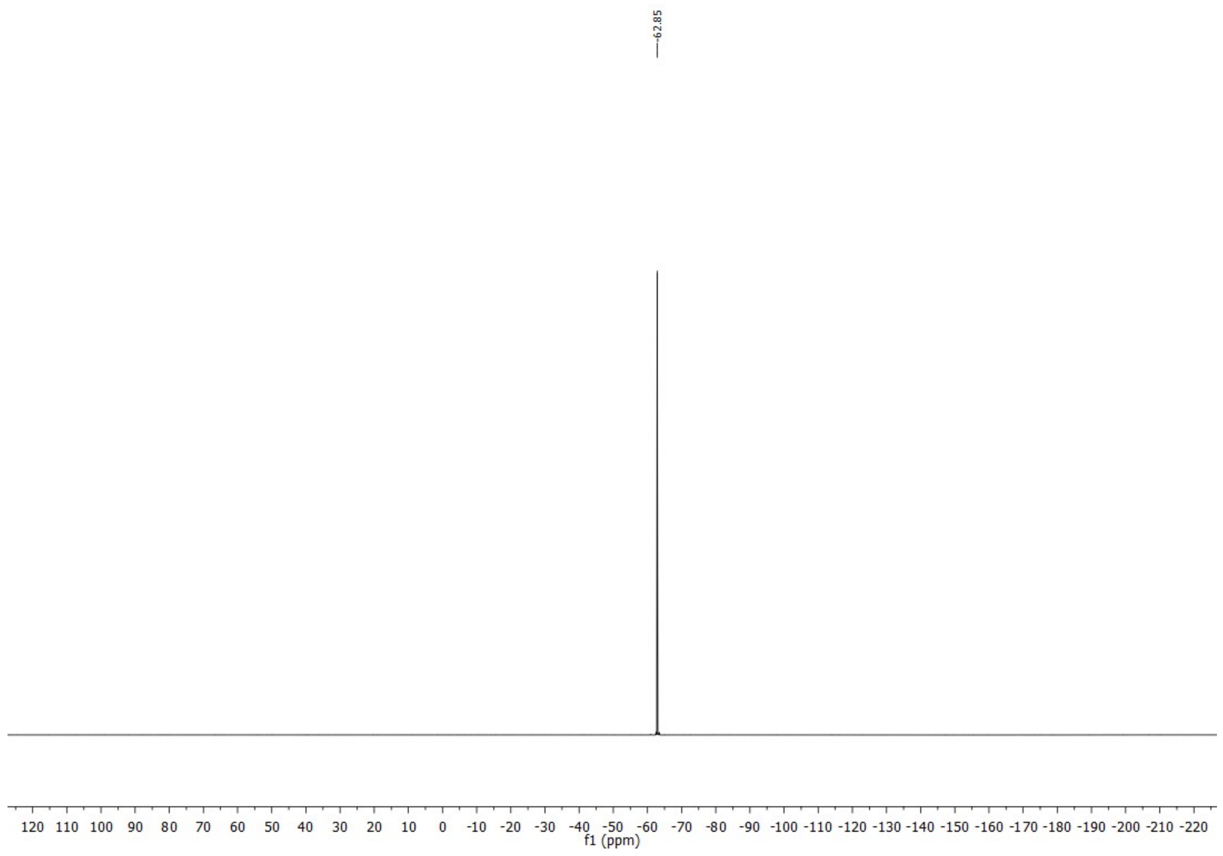
-62.85
120 110 100 90 80 70 60 50 40 30 20 10 0 -10 -20 -30 -40 -50 -60 -70 -80 -90 -100 -110 -120 -130 -140 -150 -160 -170 -180 -190 -200 -210 -220
f1 (ppm)

N-methyl-3-fluoroaniline

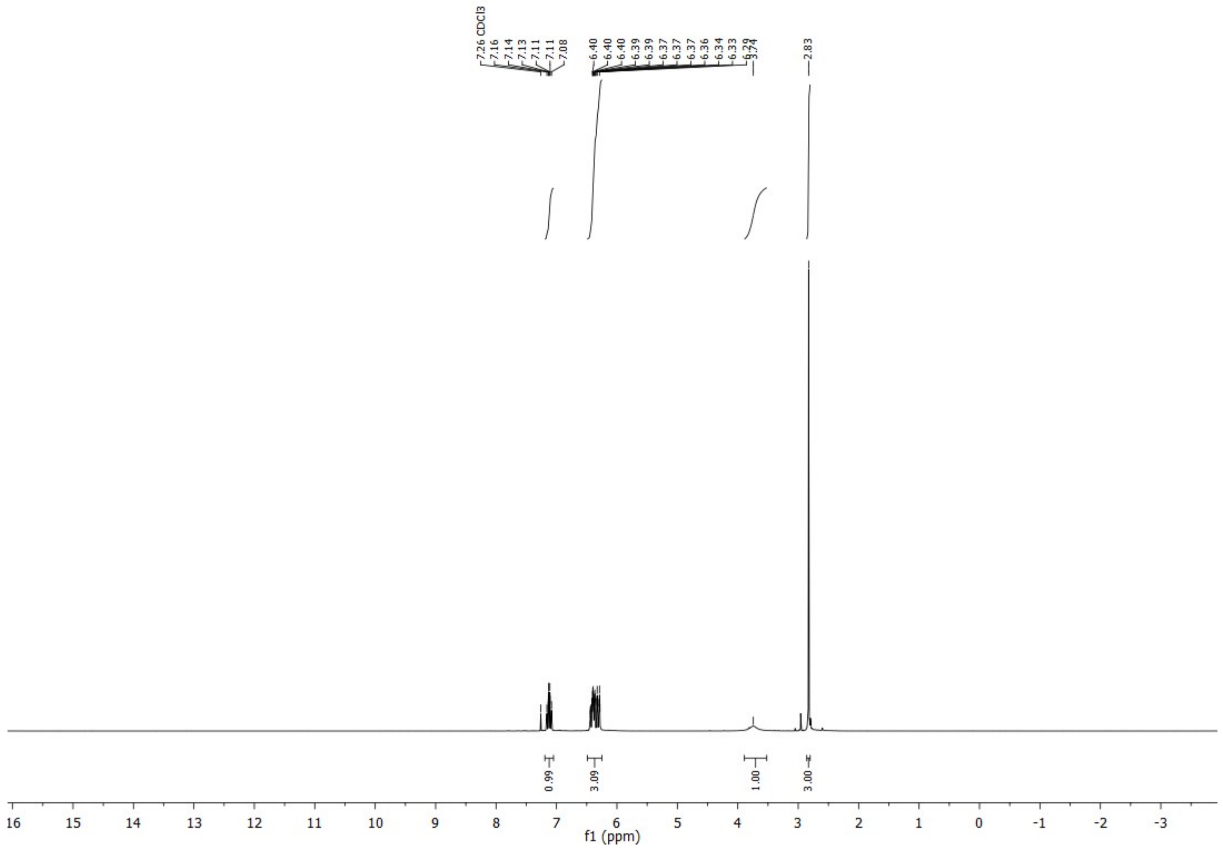

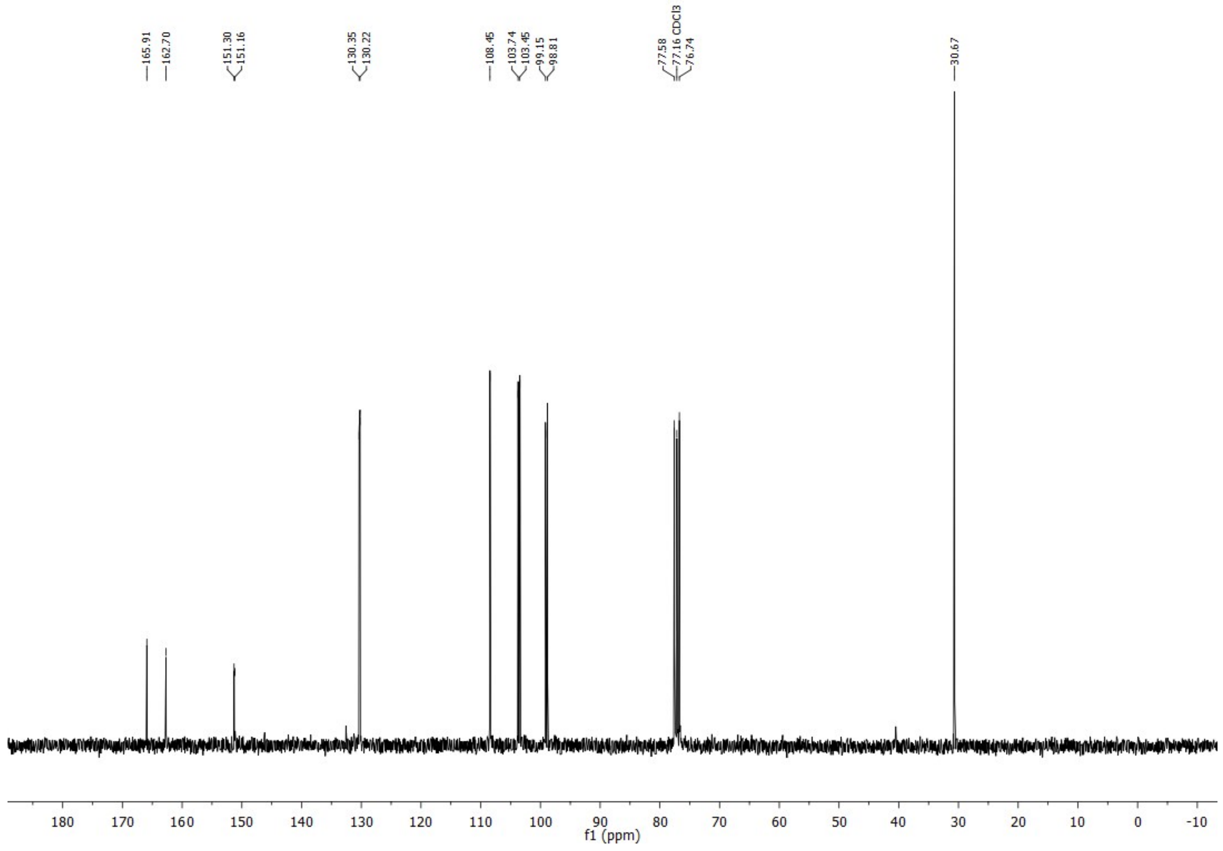
165.91
162.70
151.30
151.16
130.35
130.22
108.45
103.74
103.45
99.15
98.81
77.58
77.16 CDCl3
76.74
30.67
f1 (ppm)

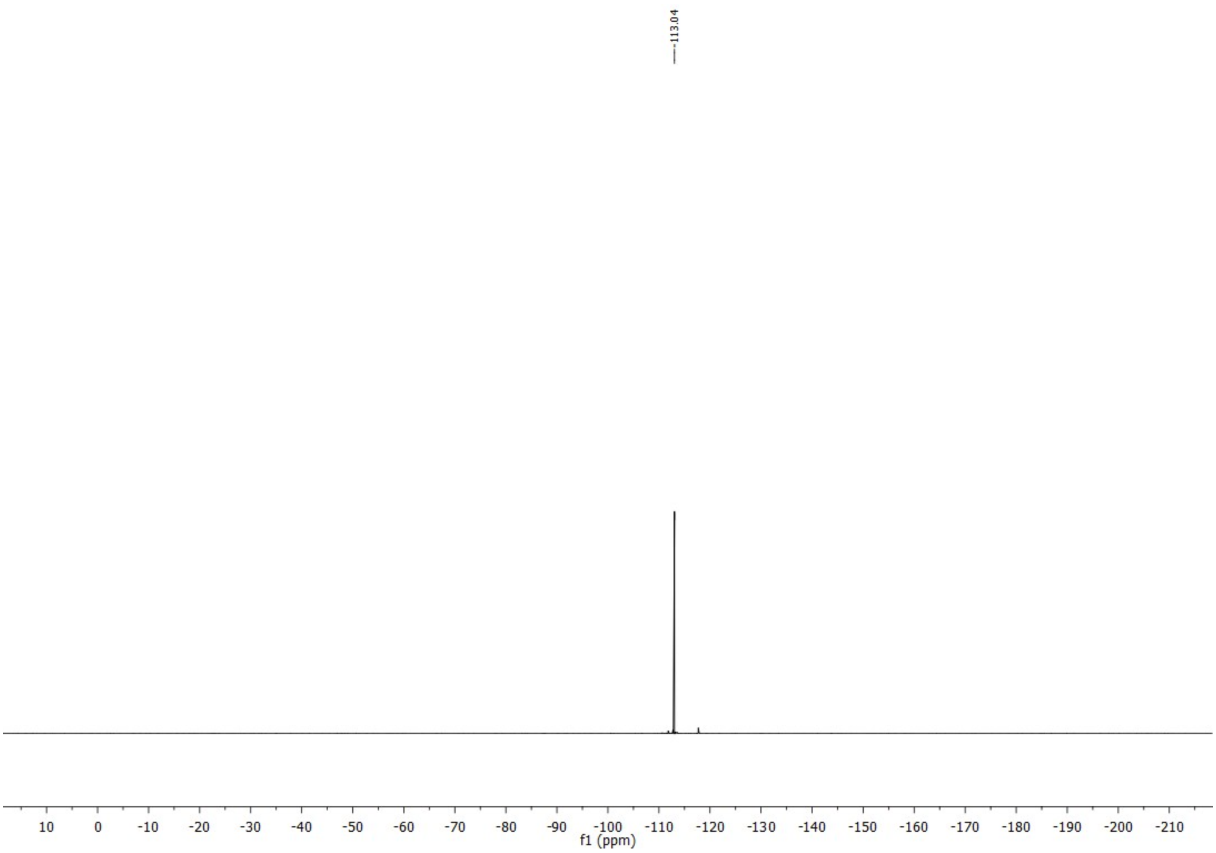
-113.04
10 0 -10 -20 -30 -40 -50 -60 -70 -80 -90 -100 -110 -120 -130 -140 -150 -160 -170 -180 -190 -200 -210
f1 (ppm)

N-methyl-[1,1'-biphenyl]-4-amine

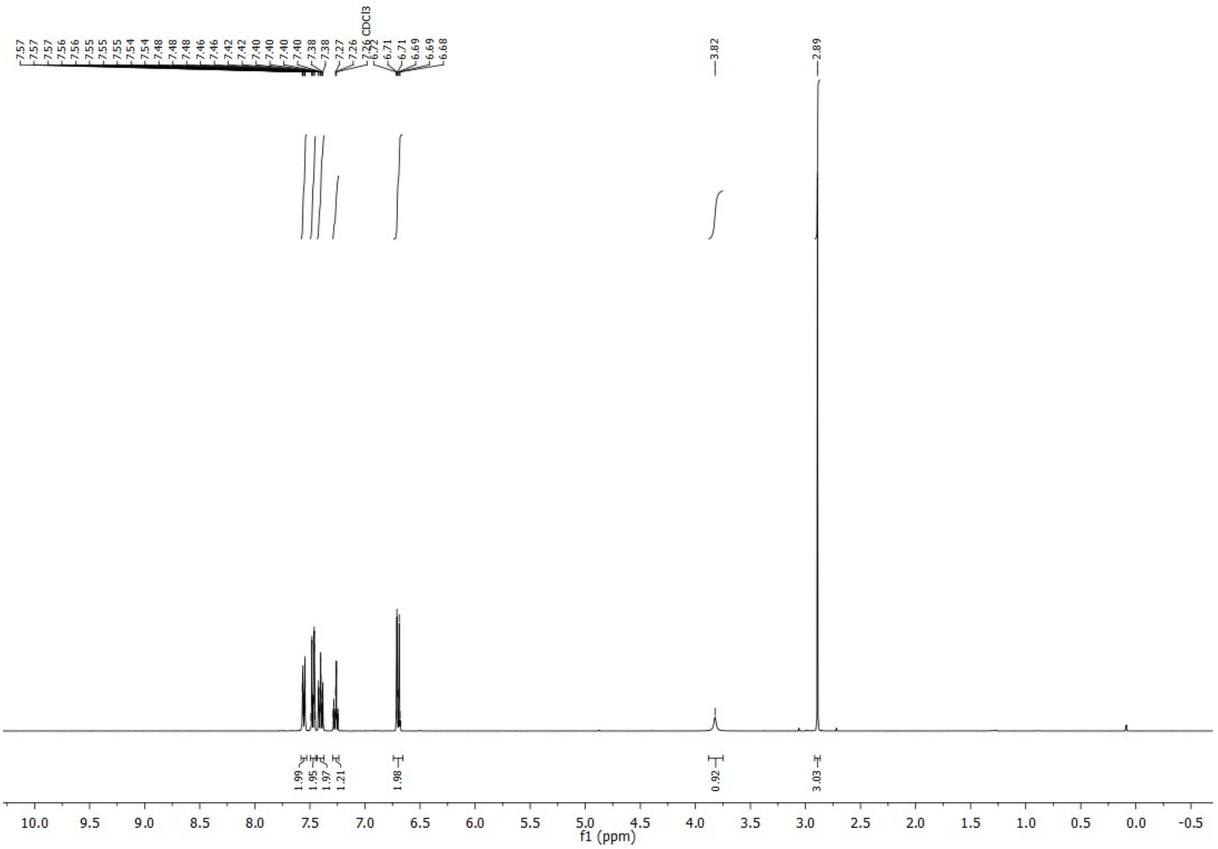

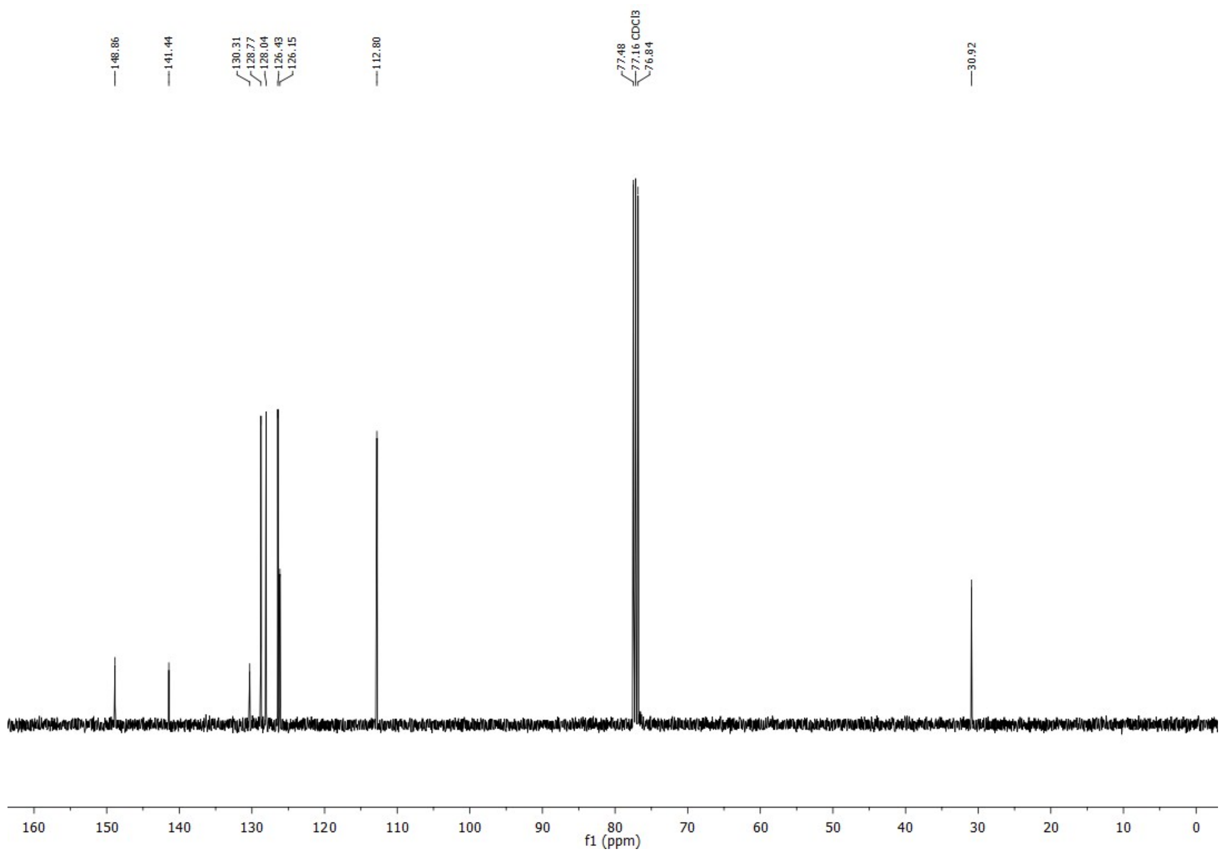

148.86
141.44
130.31
128.77
128.04
126.43
126.15
112.80
77.48
77.16 CDCl3
76.84
30.92
160
150
140
130
120
110
100
90
80
70
60
50
40
30
20
10
0
f1 (ppm)

N-methyl-4-(methylthio)aniline

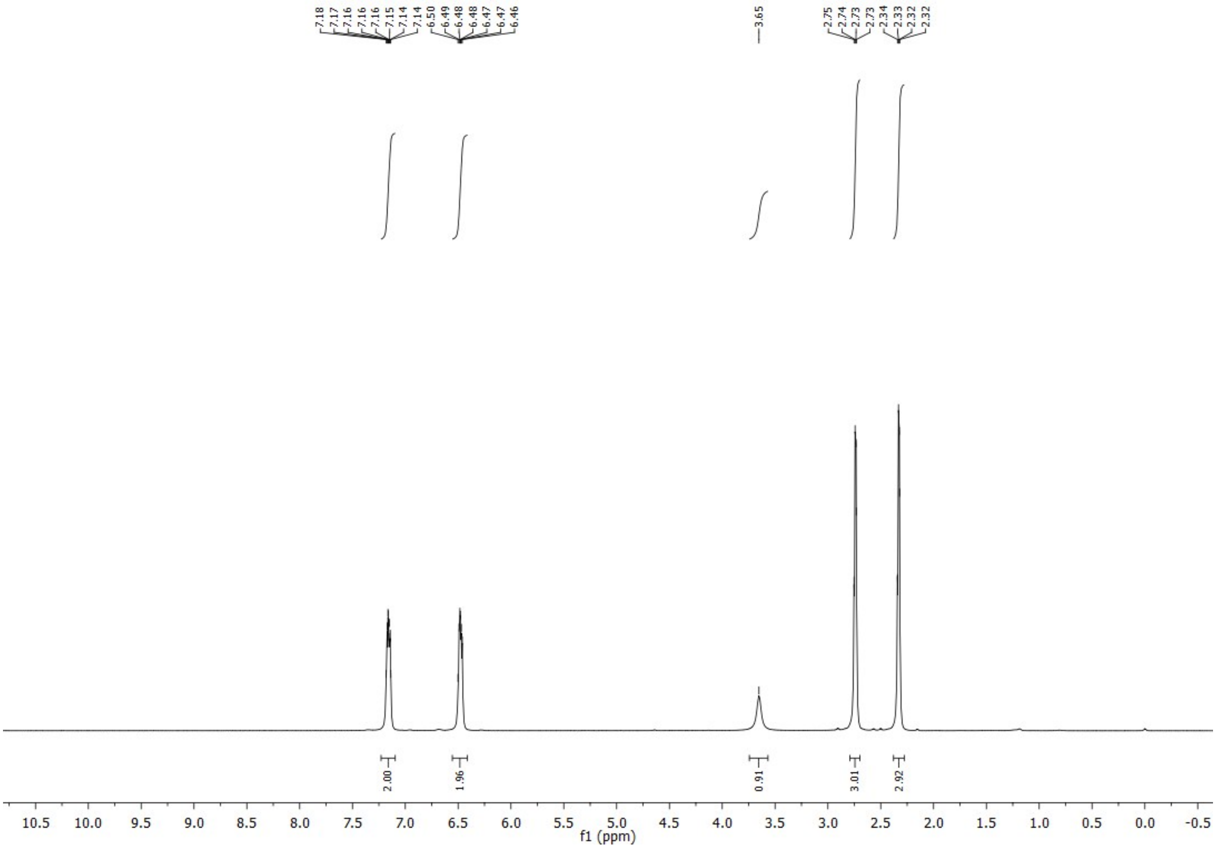

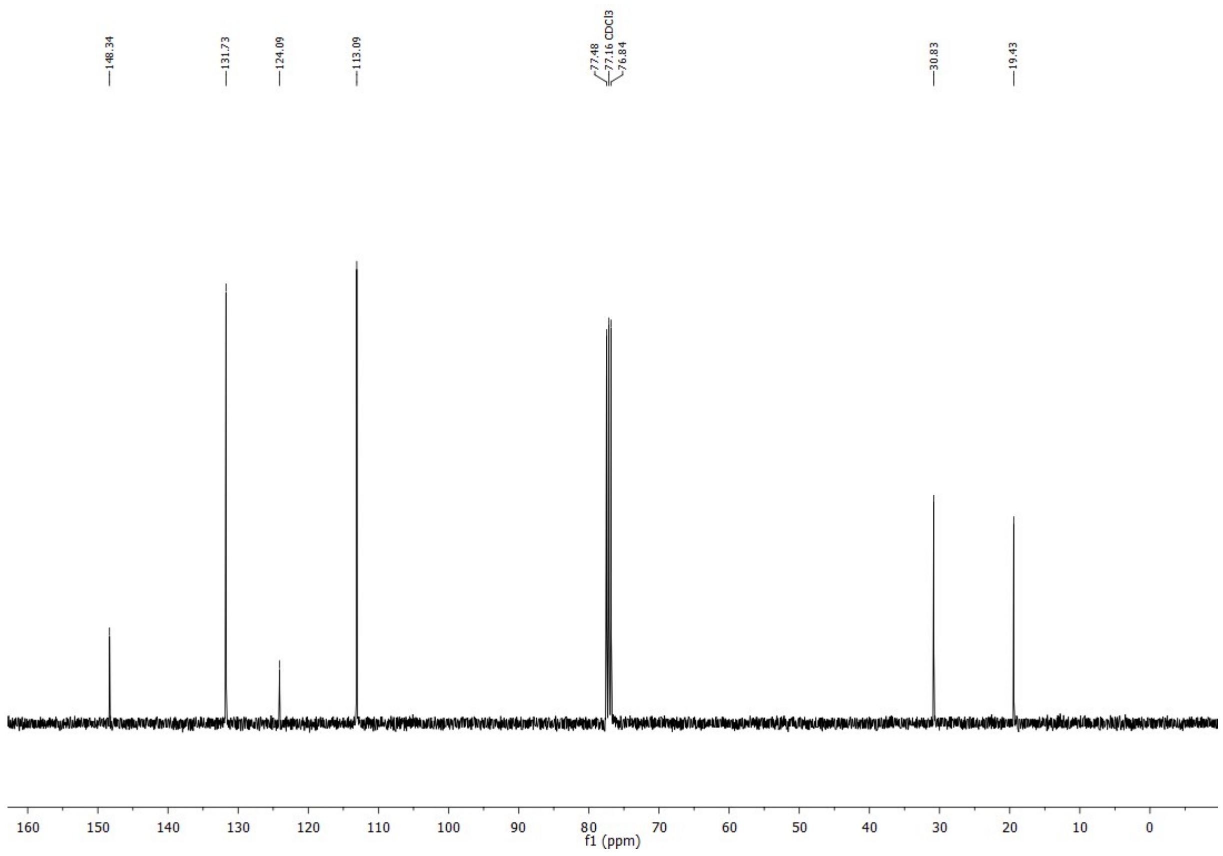
148.34
131.73
124.09
113.09
77.48
77.16 CDCl3
76.84
30.83
19.43
160 150 140 130 120 110 100 90 80 70 60 50 40 30 20 10 0
f1 (ppm)

(*E*)-*N*-methyl-4-styrylaniline

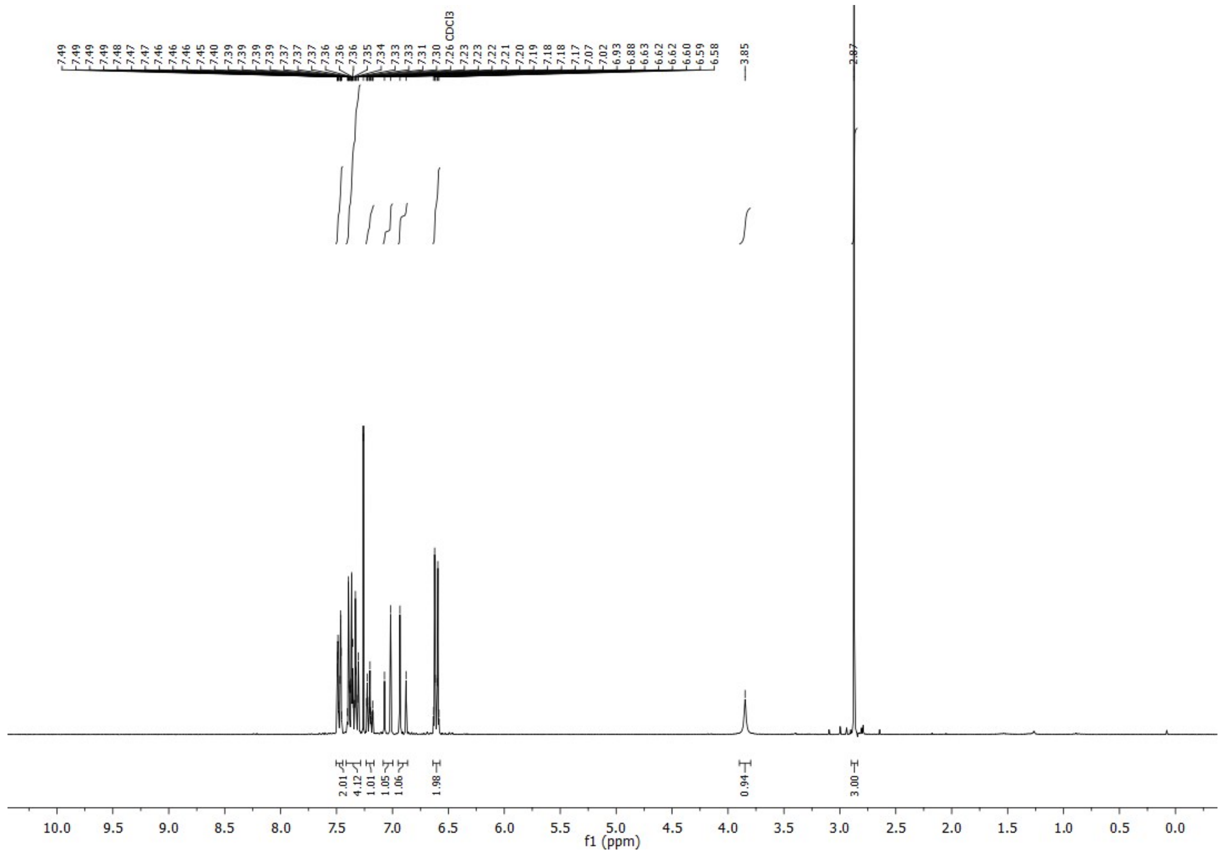

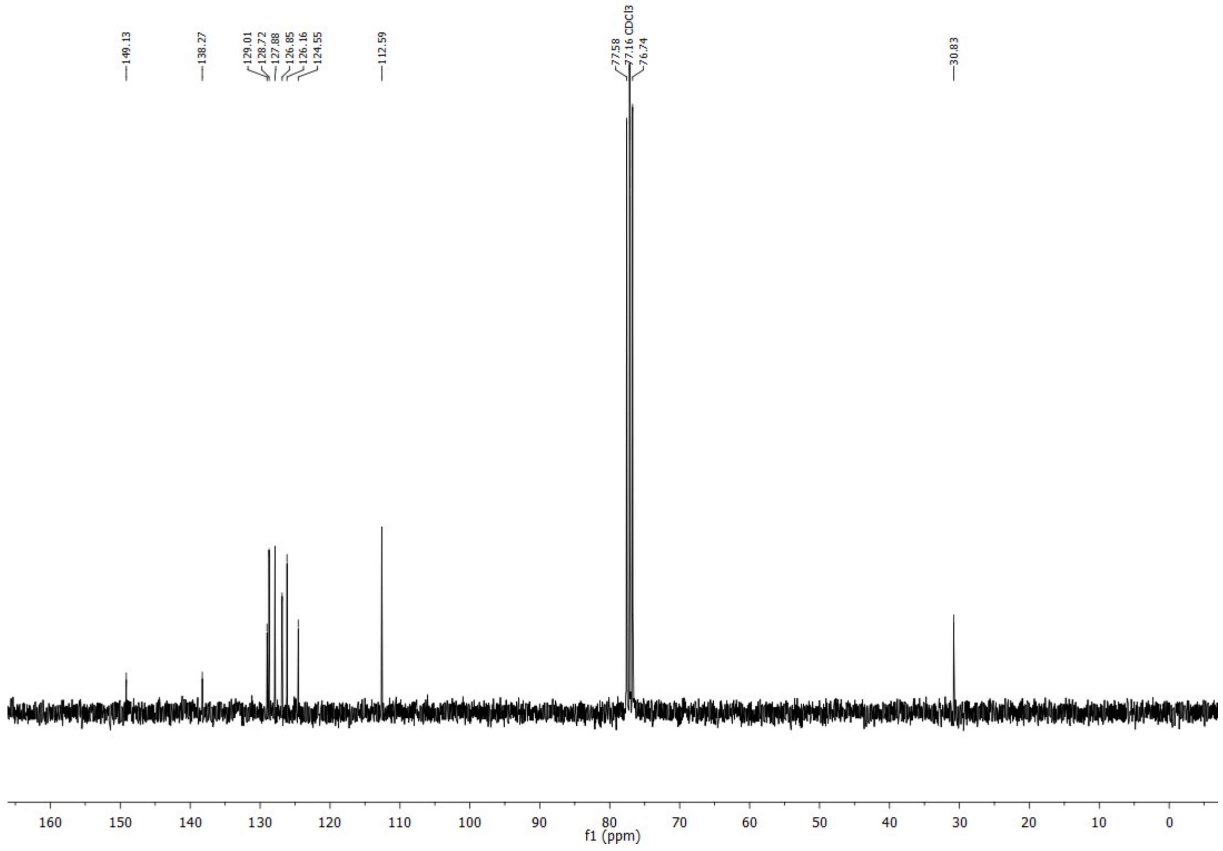
149.13
138.27
129.01
128.72
127.88
126.85
126.16
124.55
112.59
77.58
77.16 CDCl3
76.74
30.83
f1 (ppm)

4-(benzyloxy)-*N*-methylaniline

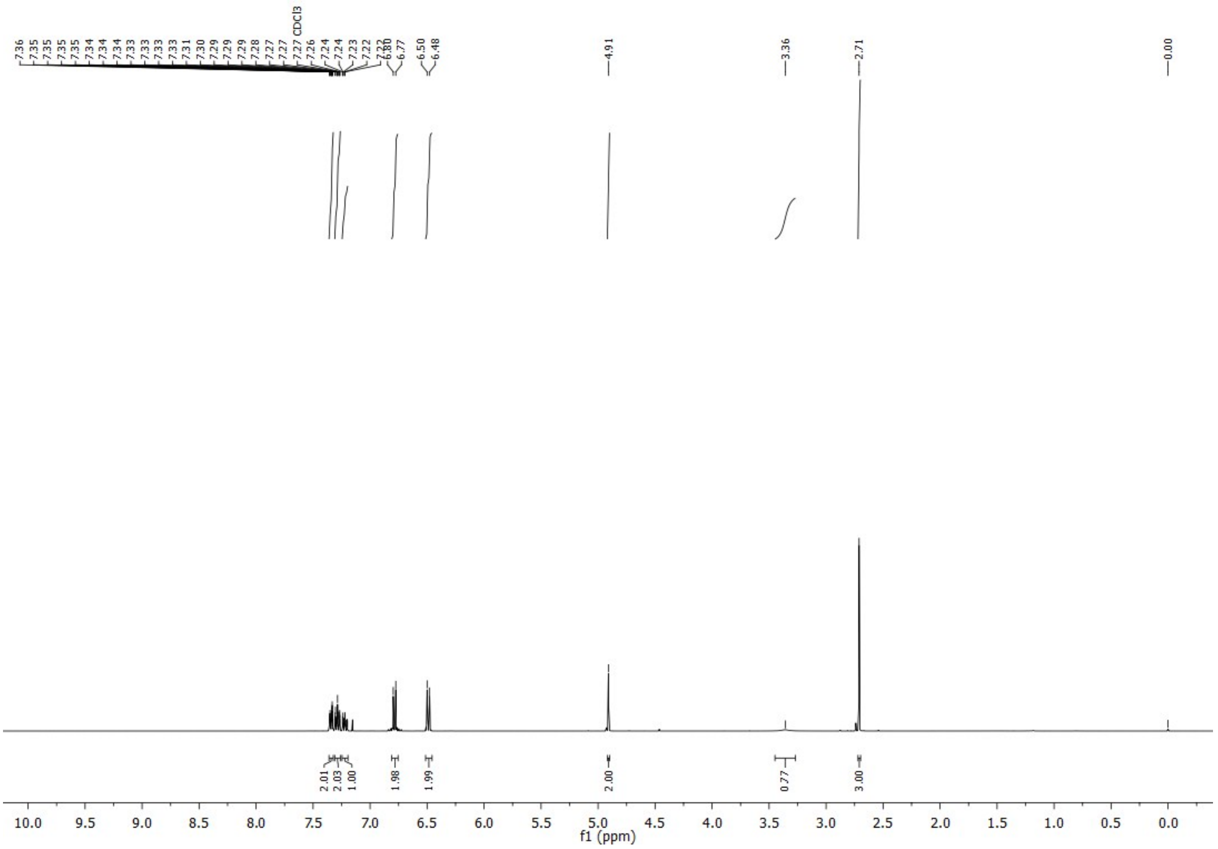

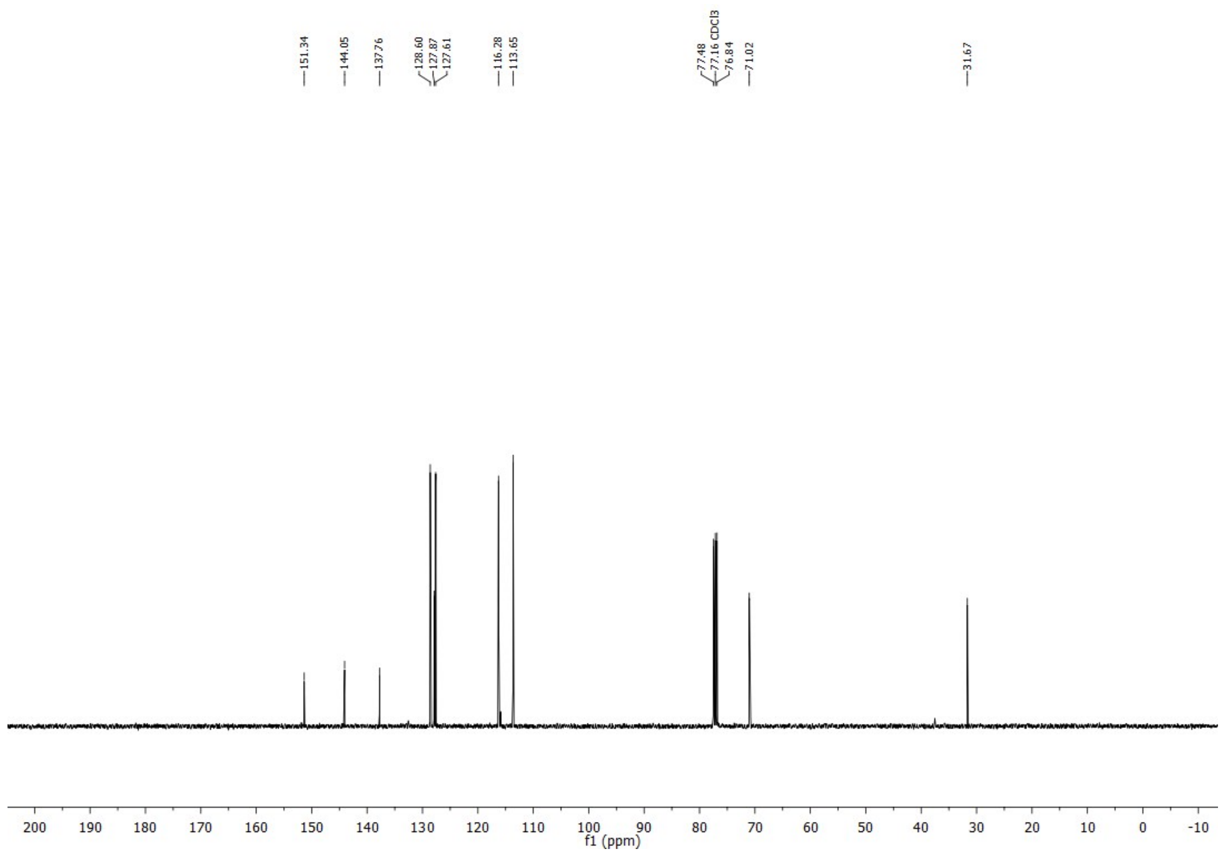

151.34
144.05
137.76
128.60
127.87
127.61
116.28
113.65
77.48
77.16 CDCl3
76.84
71.02
31.67
200 190 180 170 160 150 140 130 120 110 100 90 80 70 60 50 40 30 20 10 0 -10
f1 (ppm)

N[1],*N*[1],*N*[4]-trimethylbenzene-1,4-diamine

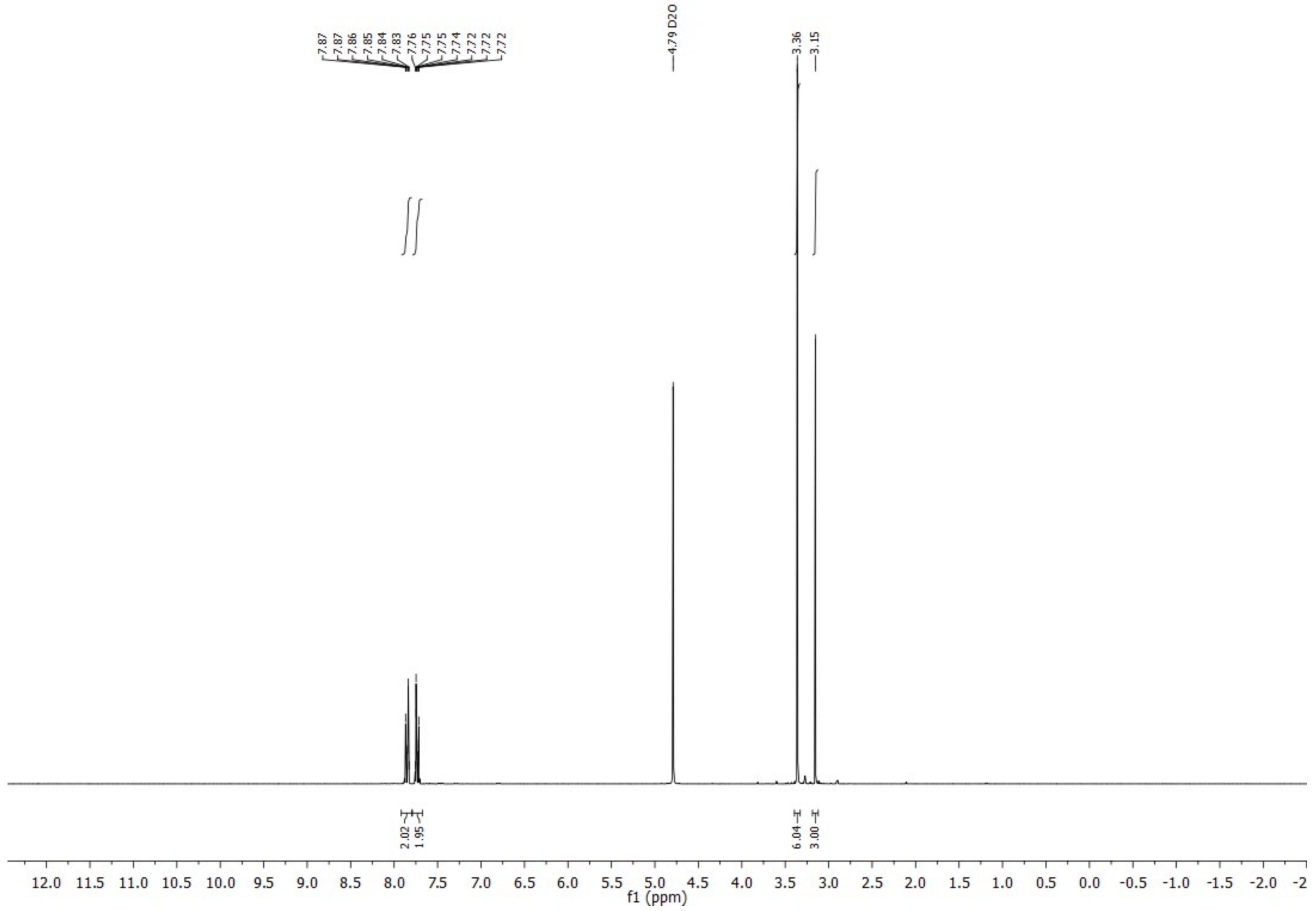

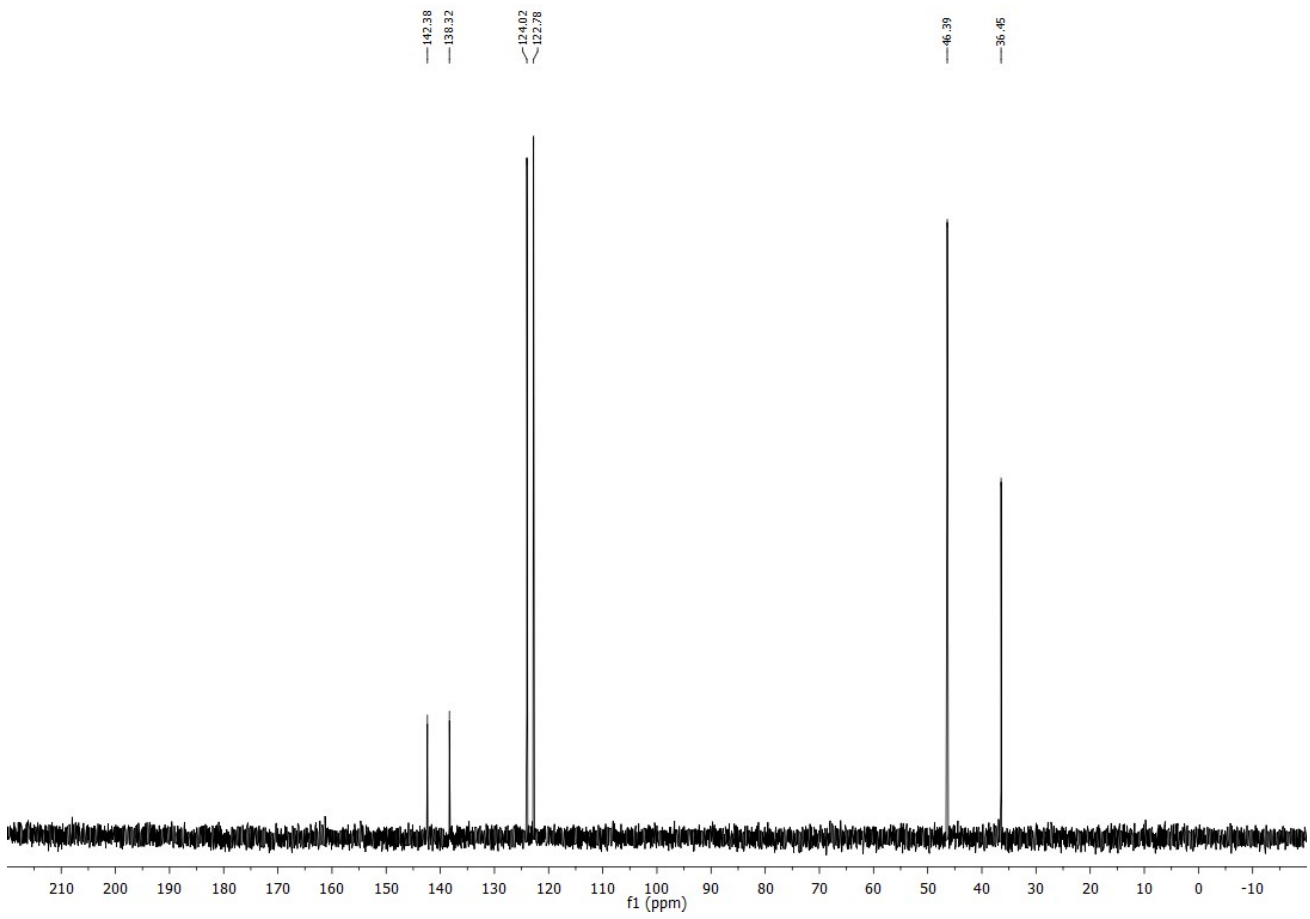
142.38
138.32
124.02
122.78
46.39
36.45
210
200
190
180
170
160
150
140
130
120
110
100
90
80
70
60
50
40
30
20
10
0
-10
f1 (ppm)

N,*N*-diphenylamine

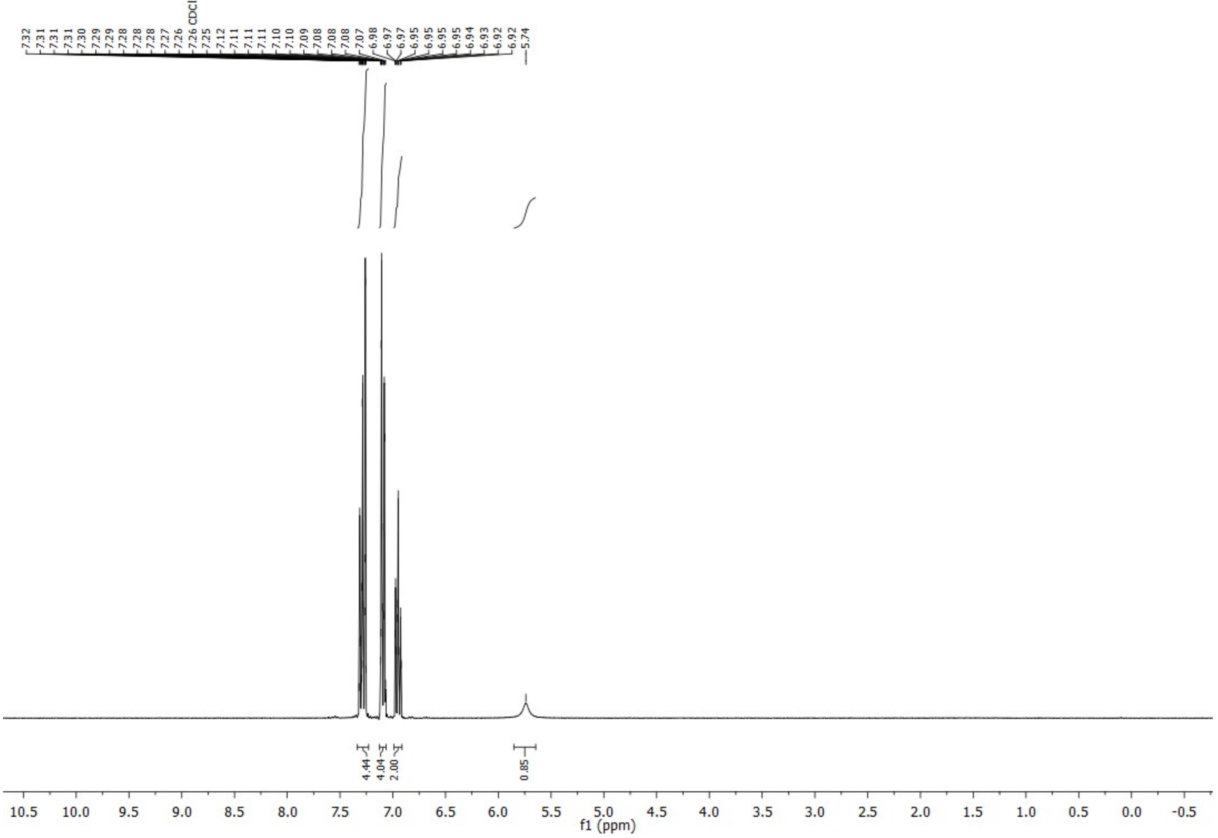

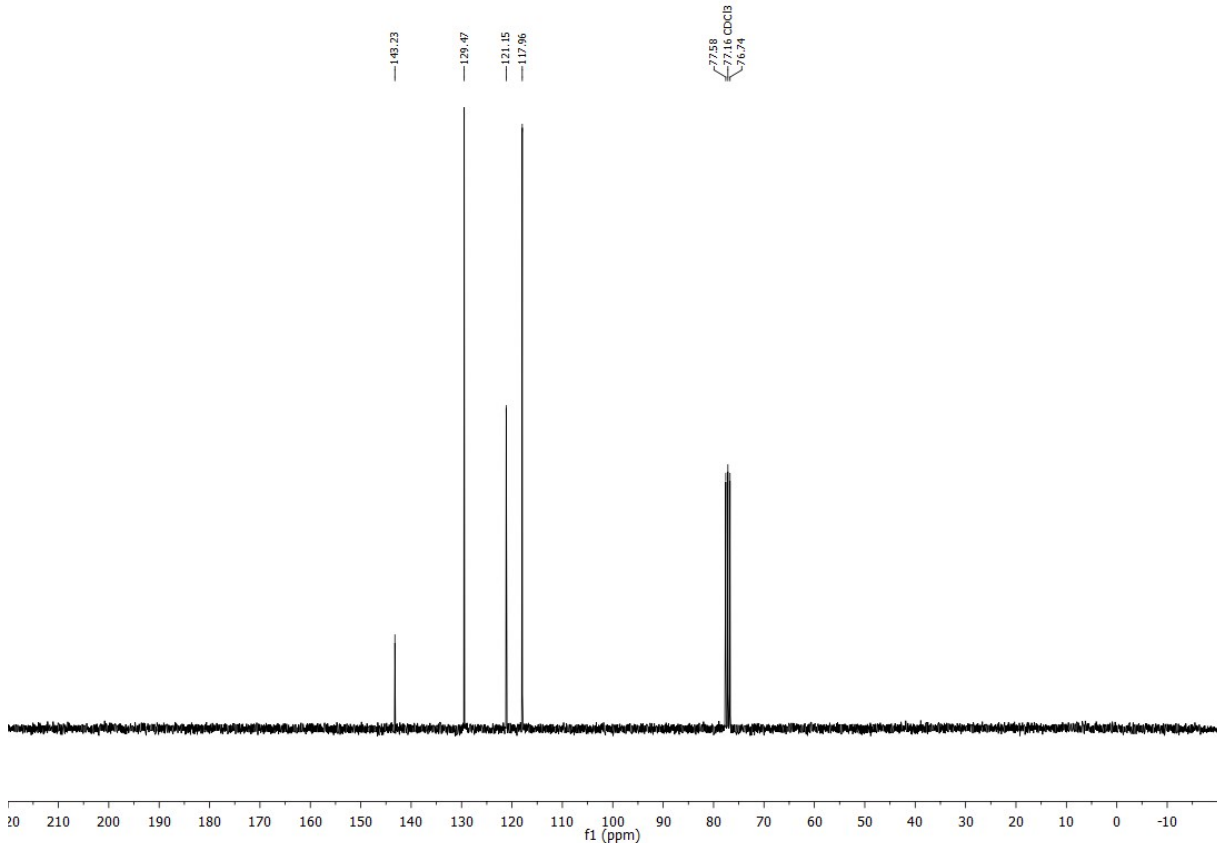
143.23
129.47
121.15
117.96
77.58
77.16 CDCl3
76.74
f1 (ppm)

N-methylpyridin-4-amine

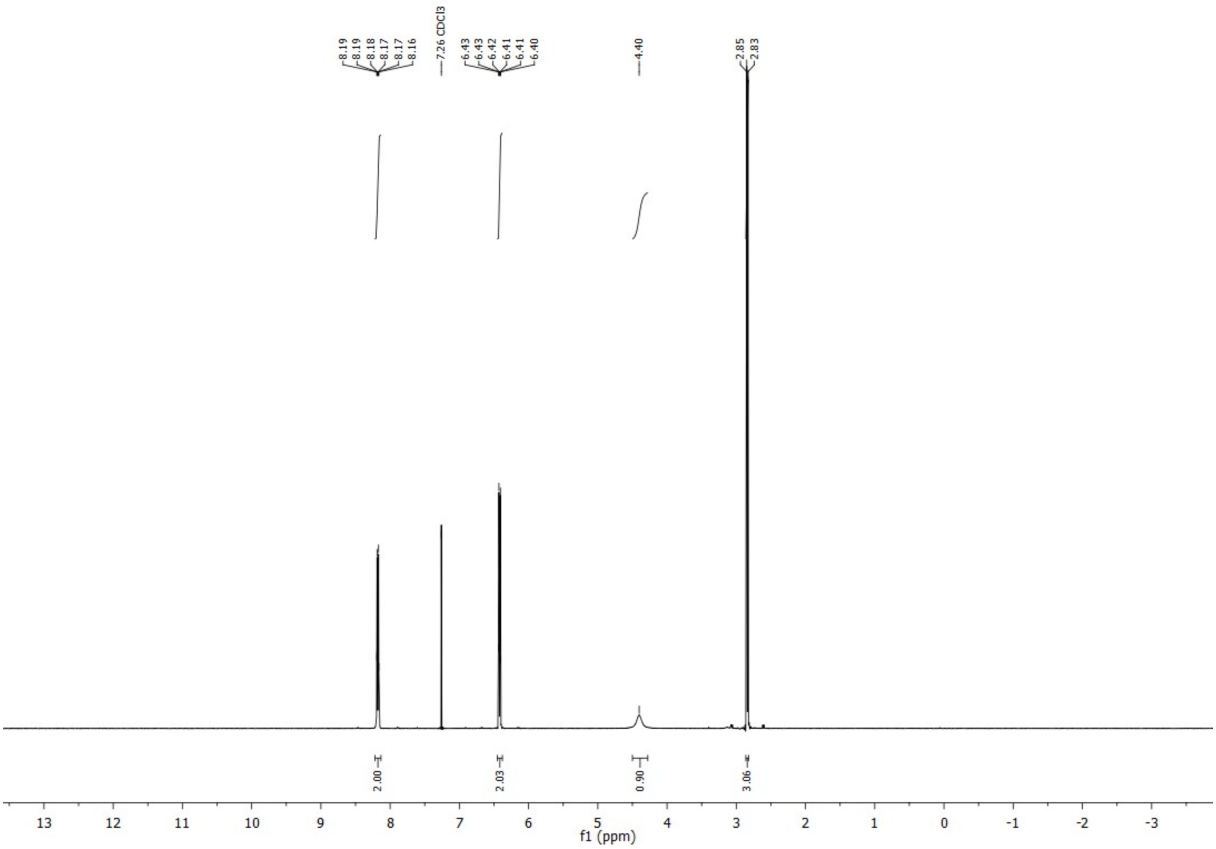

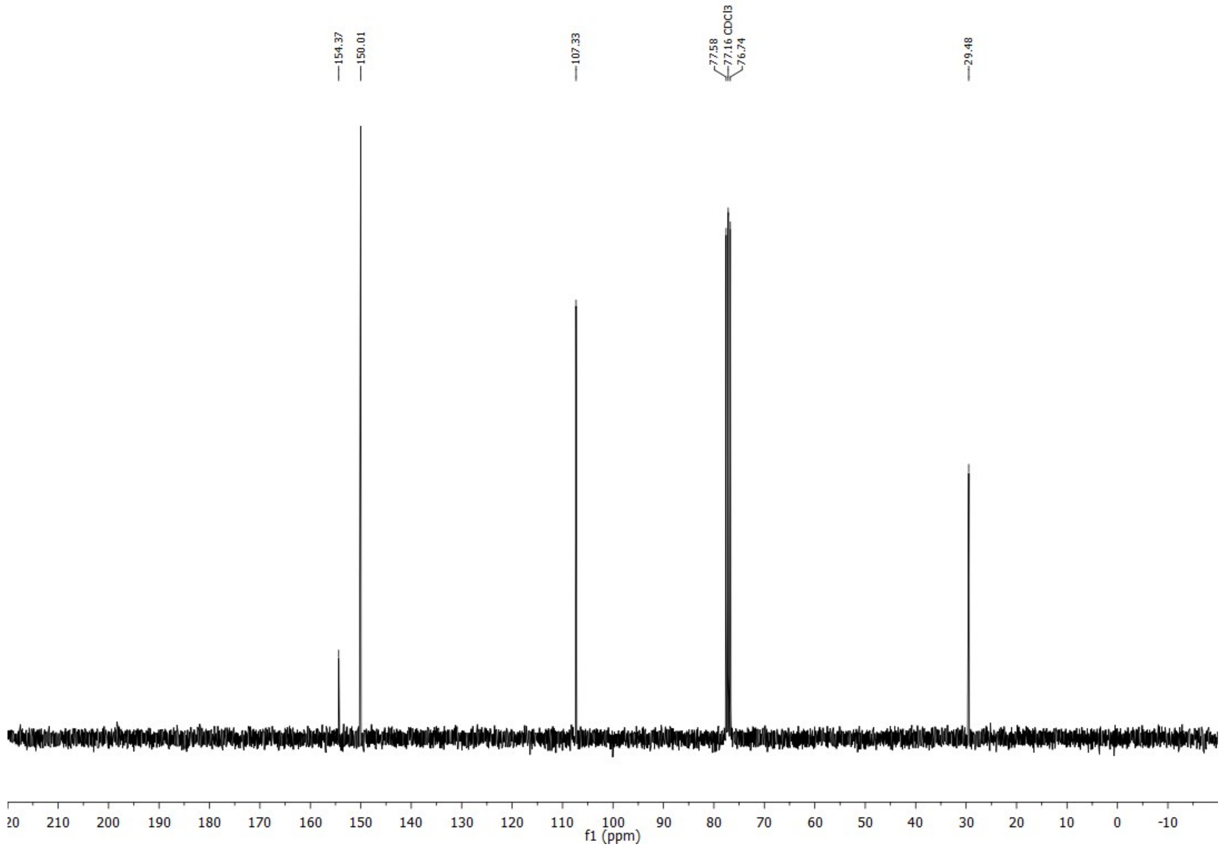
154.37
150.01
107.33
77.58
77.16 CDCl3
76.74
29.48
20 210 200 190 180 170 160 150 140 130 120 110 100 90 80 70 60 50 40 30 20 10 0 -10
f1 (ppm)

N-methyl-*N*-(4-(methylamino)phenyl)benzamide

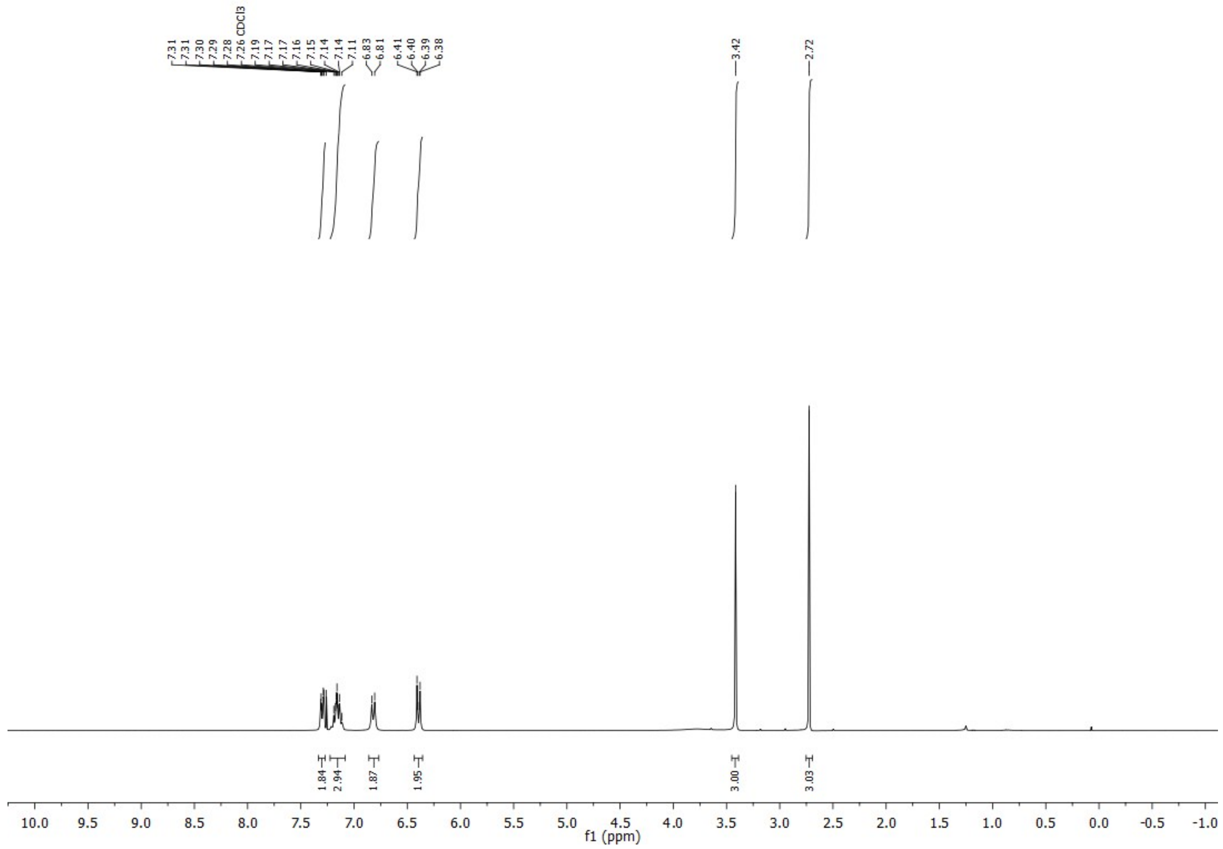

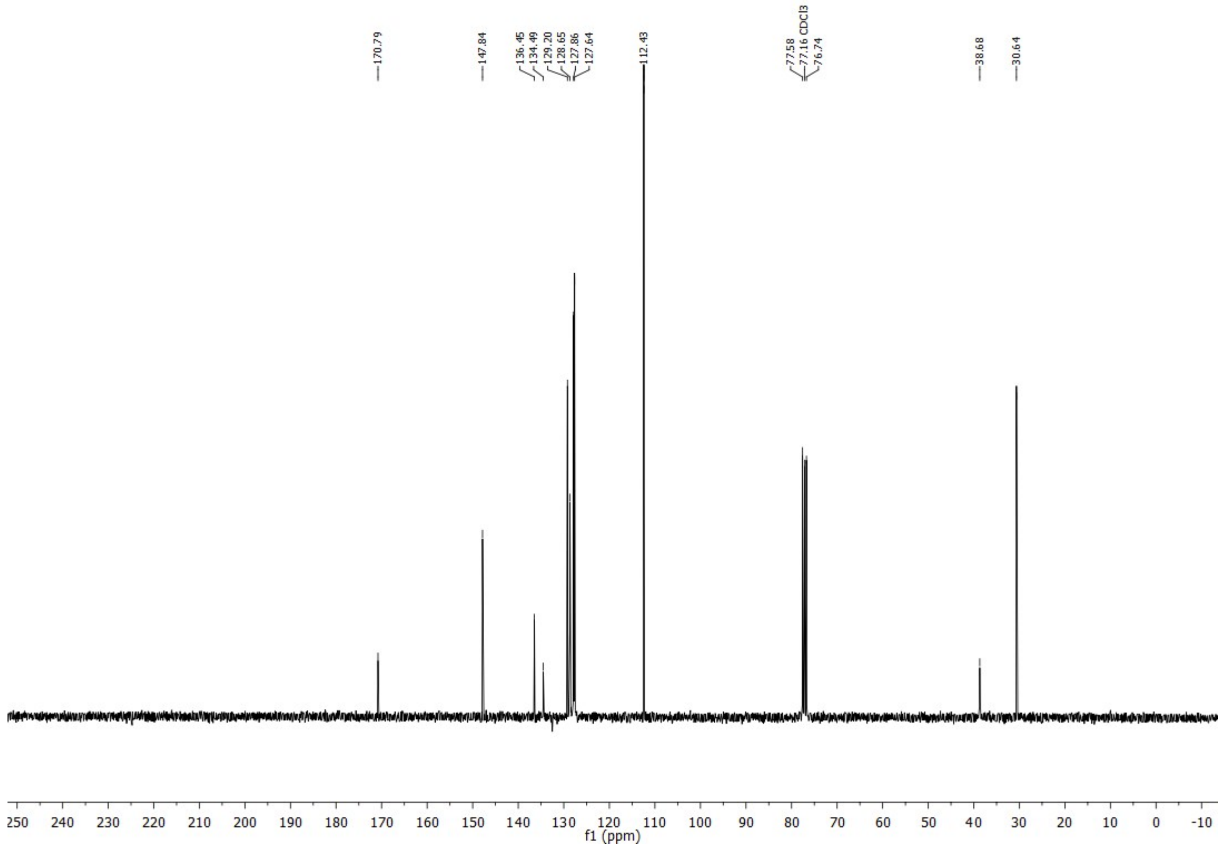
170.79
147.84
136.45
134.49
129.20
128.65
127.86
127.64
112.43
77.58
77.16 CDCl3
76.74
38.68
30.64
250 240 230 220 210 200 190 180 170 160 150 140 130 120 110 100 90 80 70 60 50 40 30 20 10 0 -10
f1 (ppm)

Computational Details

General Computational Information.

DFT calculations were carried out with the Gaussian09 software package.[10] The hybrid meta-GGA M06[11] functional was selected on the basis of geometry (Figure S1 Table S1) benchmark, using X-Ray crystal structures as references. Structures were fully optimized without any geometry or symmetry constraints, combining the double-z LANL2DZ (on Mo, including relativistic effects)[12] and 6-31+G** (on all other elements)[13] basis sets. Vibrational frequencies were computed at the same level of theory to classify all stationary points as either saddle points (transition states, with a single imaginary frequency) or energy minima (reactants, intermediates and products, with only real frequencies). These calculations were also used to obtain the thermochemistry corrections (zero-point, thermal and entropy energies) at the experimental p = 50 atm and T = 373 K. The energy of the optimized geometries was refined by single point calculations with triple-z quality basis sets, including the LANL2TZ[12] on Mo and the 6-311+G** on all other elements.[14] The energies reported in the text were obtained by adding the thermochemistry corrections to the refined potential energies. The solvation effects of toluene were included in both the geometry optimizations and energy refinements using the continuum SMD model.[15] The ultrafine (99,590) grid was used in all calculations to increase numerical accuracy and to facilitate convergence. A data set collection of input files and computational results is available in the ioChem-BD repository and can be accessed online via https://iochem-bd.bsc.es/browse/handle/100/193698.[16] The complex reaction mechanisms inferred from the calculations were interpreted by means of quantitative microkinetic models (Figure S4, Figure S5 and Table S2), simulated with the COPASI software.[17] Time course simulation were carried with the LSODA algorithm.

DFT functional benchmark

In a previous work of the group, the hydrogenation of amides by an iron (II) Noyori-type bifunctional catalyst was studied by using the M06 functional.[6] This method was selected based on a method benchmark using X-ray geometries and CCSD(T) energies. In order to obtain comparable results, the same functional was initially chosen for this study. This functional was found to give geometries in good agreement with those experimentally obtained for complexes Mo-1a (RMSD = 0.037 Å), Mo-1c (RMSD = 0.031 Å), and Mo-4 (RMSD = 0.030 Å) and therefore was selected for this study. The geometry optimization and energies of the possible spin states for these species were consistent with a doublet for Mo-1a, and a singlet ground state for Mo-1c and Mo-4, respectively.

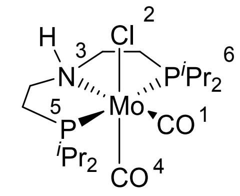

Mo-1a

(doblet) 0.0
(quadruplet) 44.2
(sextuplet) 78.1

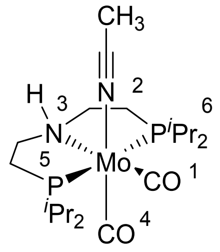

Mo-1c

(singlet) 0.0
(triplet) 37.8
(quintuplet) 76.9

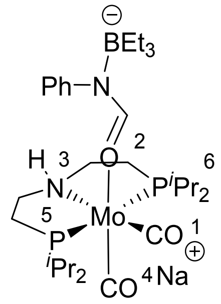

Mo-4

(singlet) 0.0
(triplet) 45.0
(quintuplet) -----

Figure S1. Mo-complexes used for the geometry benchmark using M06 with the labels used in Table S1, and the corresponding free energies for the first and second excited states. Mo-4 quintuplet did not converge.

Mo-1a

	Mo-CO^1	Mo-Cl^2	Mo-NH^3	Mo-CO^4	Mo-P^5	Mo-P^6	RMSD
<u>Experimental</u>	1.9536	2.5817	2.3029	1.9118	2.5002	2.4878	
<u>M06</u>							
(doublet)	1.9785	2.5814	2.3754	1.9549	2.5099	2.5099	0.0373
(quadruplet)	2.2574	2.6006	2.5649	1.9931	2.5313	2.5314	0.1687
(sextuplet)	2.2645	2.5904	2.5645	2.2881	2.6643	2.5793	0.2388

Mo-1c

	Mo-CO^1	Mo-NCCH$_3$^{2}	Mo-NH^3	Mo-CO^4	Mo-P^5	Mo-P^6	RMSD
<u>Experimental</u>	1.9203	2.2274	2.3227	1.9155	2.4389	2.4296	
<u>M06</u>							
(singlet)	1.9496	2.2247	2.3793	1.9490	2.4485	2.4485	0.0307
(triplet)	1.9775	2.2606	2.3677	1.9873	2.5054	2.5083	0.0608
(quintuplet)	2.3050	2.2898	3.2786	2.0528	2.6364	2.6682	0.4436

Mo-4

	Mo-CO^1	Mo-OR^2	Mo-NH^3	Mo-CO^4	Mo-P^5	Mo-P^6	RMSD
<u>Experimental</u>	1.8893	2.2391	2.3230	1.8893	2.4406	2.4421	
<u>M06</u>							
(singlet)	1.9116	2.2477	2.3694	1.8967	2.4777	2.4794	0.0304
(triplet)	2.0950	2.2919	2.4984	1.8675	2.5240	2.5271	0.1229

Table S1. Root mean square deviation of distances (in Å) of optimized geometries with respect experimental single crystal X-ray diffraction geometries, for Mo-1a, Mo-1c and Mo-4 molecules.

Comparison Iron system vs Molybdenum system.

In this work, a mechanism in which a methoxide intermediate is involved in the hemiaminal C-N bond cleavage (Mo-ts-12-13) has been proposed with Mo. This mechanism differs from the one previously proposed with Fe, in which the N of the hemiaminal is coordinated to Fe during the C-N bond cleavage (Fe-ts-C^{H}N^{H}). We have calculated ts-12-13 with Fe (see Figure S2) and has a higher energy than ts-C^{H}N^{H}, indicating that the methoxide mechanism is not preferred with Fe. The higher stability of the methoxide intermediate with Mo (Mo-9a) compared with (Fe-9a) may explain this difference in reactivity.

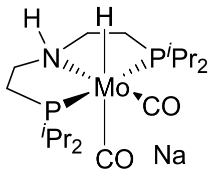

Mo-5
0.0

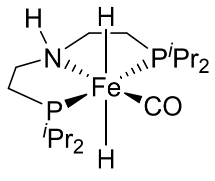

Fe-5
0.0

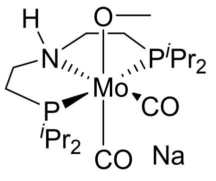

Mo-9a
-9.7

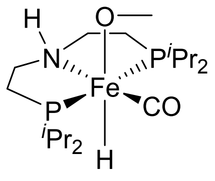

Fe-9a
3.3

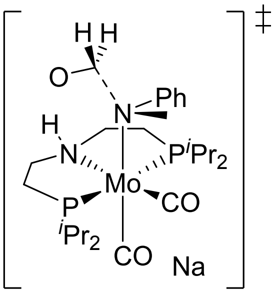

Mo-ts-C^{H}N^{Me}
[22.9]

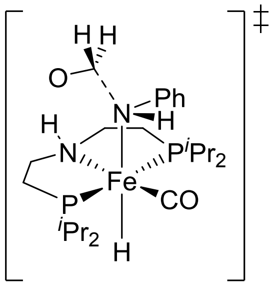

Fe-ts-C^{H}N^{H}
[24.9]

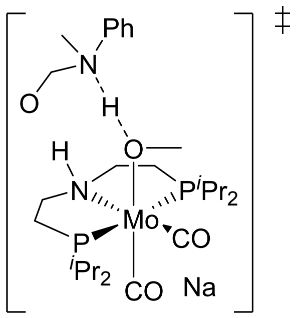

Mo-ts-12-13
[9.4]

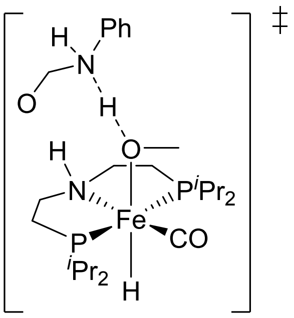

Fe-ts-12-13
[25.2]

Figure S2. Computed free energies, in kcal mol^{-1}, for selected TSs and minima involved in the hemiaminal C-N bond cleavage step with Mo and Fe-systems.

Catalyst recovery mechanism.

The mechanism of catalyst recovery by addition of H_2 to the methoxide complex Mo-9a is shown in Fig. S3. In this pathway, methanol assists the activation of the Mo-H2 complex (Mo-14) by acting as a proton-shuttle. The global energy barrier for the catalyst recovery mechanism is 23.0 kcal mol^{-1}, which is similar to the global barrier for the hydride transfer with *N*-methylformanilide (5) (22.8 kcal mol^{-1}). This result suggests that both hydride transfer and catalyst recovery should be considered as rate limiting processes in the hydrogenation of amides catalyzed by Mo.

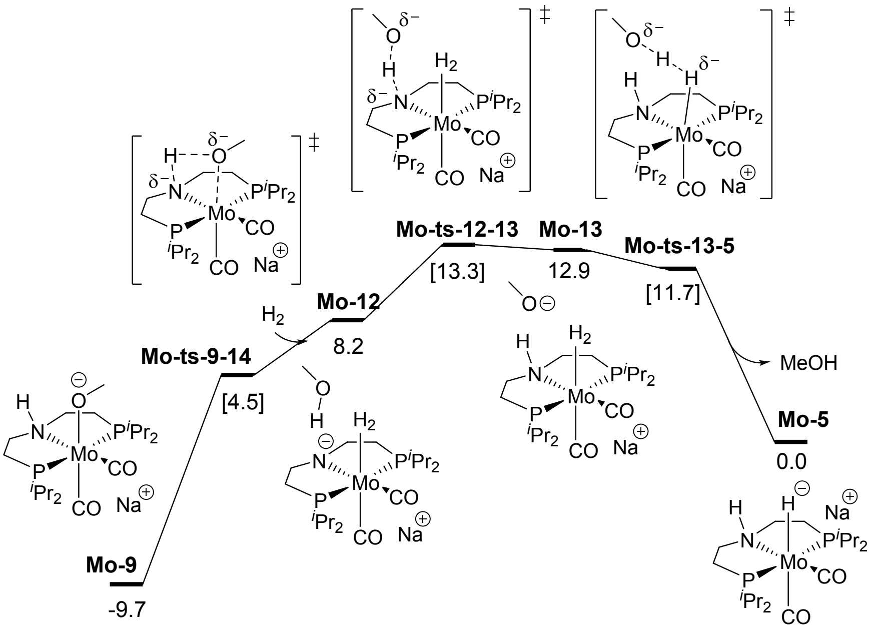

Figure. S3 Reaction pathway of the MeOH assisted hemiaminal proton transfer and posterior C-N bond cleavage. Gibbs energies in toluene (SMD) at 50 bar and 373 K are given in kcal mol^{-1}.

Hydrogenation of formaldehyde

The free energy profile for the formaldehyde reduction is represented in Figure S4.

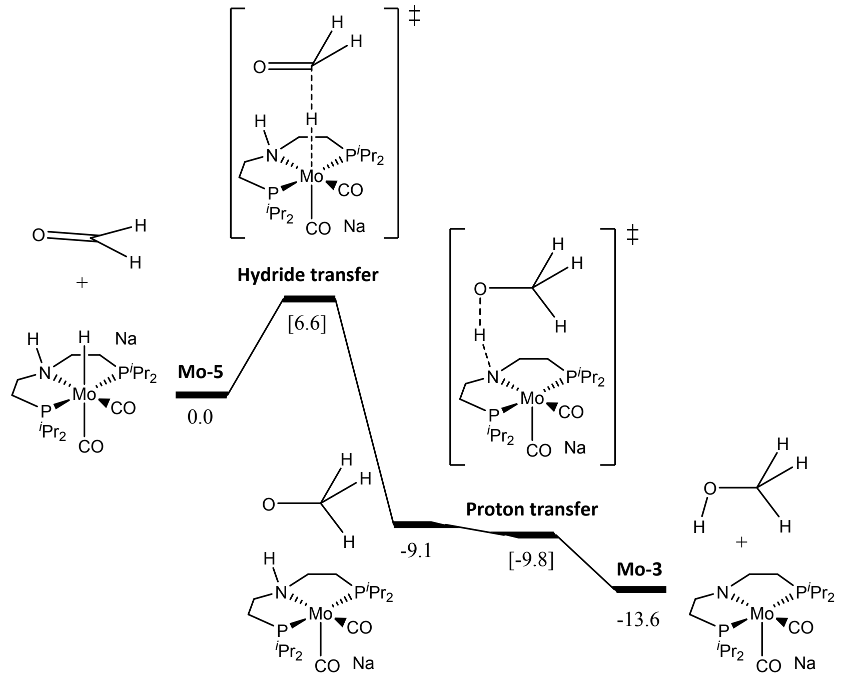

Figure S4 Free energy profile in kcal mol^{-1} for the formaldehyde hydrogenation to methanol by Mo-5.

Mo-9a and Mo-ts-6-7 with Li^+, Na^+ and K^+

The energy barrier for the hydride transfer involves Mo-ts-6-7 and the methoxy intermediate Mo-9a, which is the ground state. The energy barriers computed for Na^+, K^+ and Li^+ is 22.9, 23.0, and 28.8 kcal/mol, respectively. Therefore, the difference in energy barrier for Li+ and K+ is 5.8 kcal/mol instead of 9 kcal/mol. In order to analyse these differences in energy barrier, the stability of Mo-9a and Mo-ts-6-7 with the different cations have been evaluated using isodesmic reactions (Figures S5). The energies of these reactions and the geometrical analysis of Mo-9a and Mo-ts-6-7 (Figures S6) suggest that the higher energy barrier for Li+ is due to a higher stabilization of the ground state (Mo-9a) with this cation, probably due to stronger electrostatic interaction of Li+ with the OMe group.

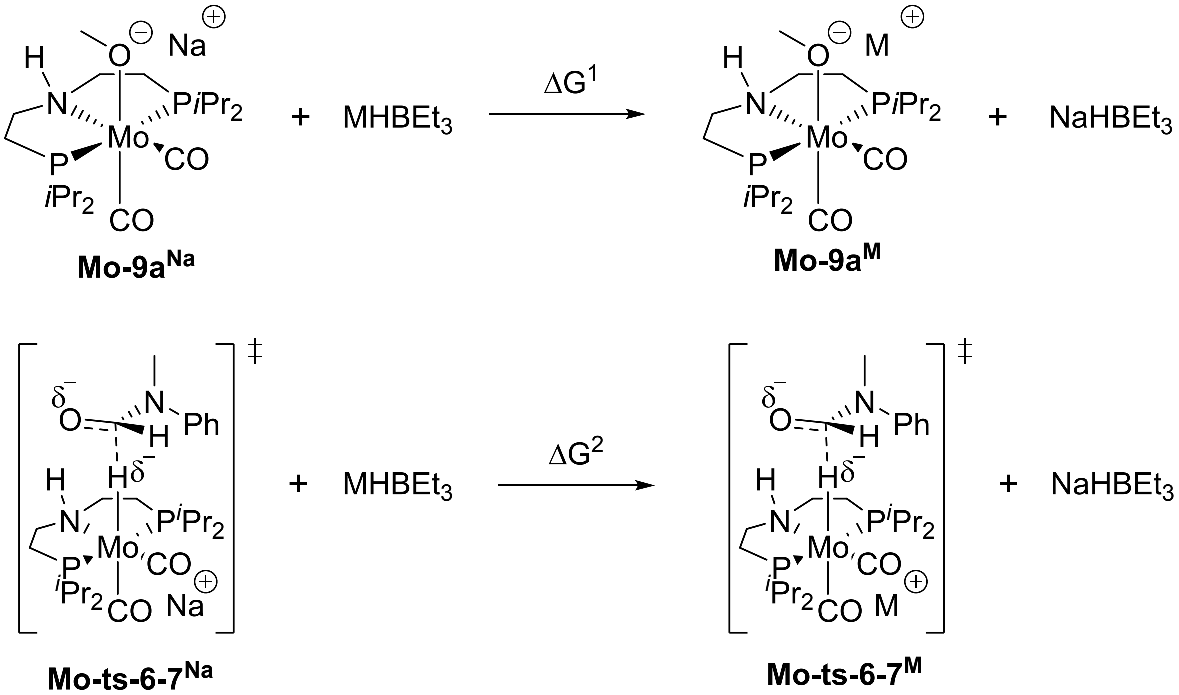

M	ΔG^1 (kcal mol^{-1})	ΔG^2 (kcal mol^{-1})
Li^+	- 4.6	1.4
K^+	- 0.9	-0.7

Figure S5. Free energies (kcal mol^{-1}) for the comparative isodesmic reaction between Li^+, Na^+ and K^+ in Mo-9a and Mo-ts-6-7

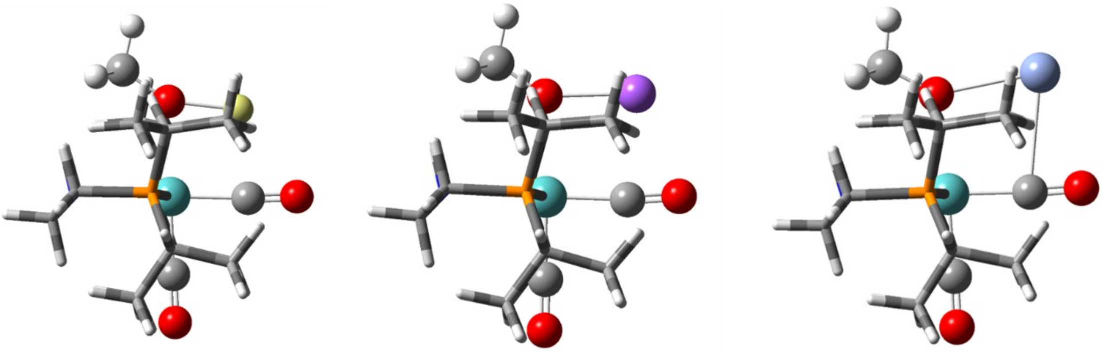

Mo-9a^{Li} **Mo-9a^{Na}** **Mo-9a^{K}**

Distances	O-Li 1.74 Å	O-Na 2.14 Å	O-K 2.48 Å
	O-Mo 2.36 Å	O-Mo 2.36 Å	O-Mo 2.35 Å

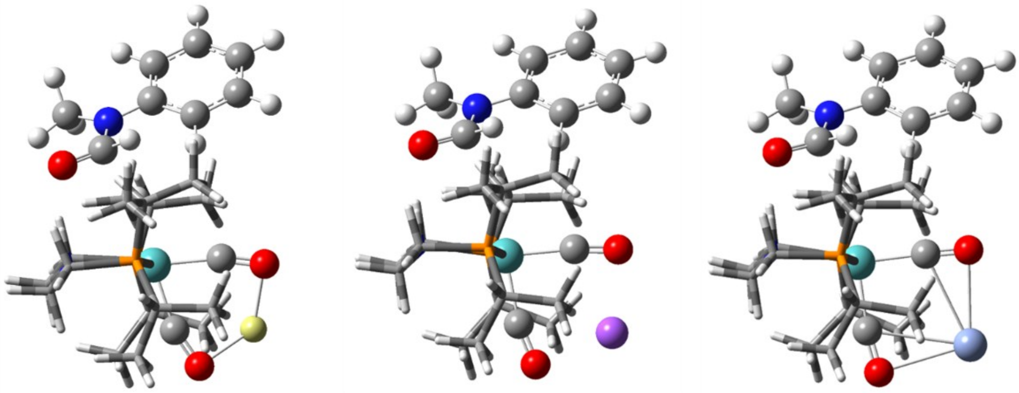

Mo-ts-6-7^{Li} **Mo-ts-6-7^{Na}** **Mo-ts-6-7^{K}**

Angles	C-Mo-C 78.5	C-Mo-C 84.7	C-Mo-C 89.2

Figure S6. Optimized geometries for Mo-9a and Mo-ts-6-7 with selected distances and angles in Å. PNP ligand depicted in tubes/wireframes for visual clarity. Mo (turquoise), C (grey), O (red), N (blue), H (white), P (orange), Li (Litium), Na (purple), K (lavender).

Microkinetic model

Microkinetic models were constructed with the COPASI software (version 4.22).[7] The initial concentrations used in the simulations were those reported in the experiments (0.25 M of N-methylformanilide, 0.207 M of hydrogen and 0, 0.125 and 0.5 M of ethanol). The concentration of hydrogen was kept constant, in line with the effectively constant pressure of hydrogen used in the reactor (50 atm). H_2 concentration was approximated using the molar fraction of H_2 in a saturated solution of H_2 in toluene at 50 atm and 100 ºC assuming incompresibility of THF and that $[H_2] << [toluene]$.[8] As in the experiments, simulations were carried out for a total time of 24 hours at T = 373 K. The models were based on deterministic time course simulations with the LSODA algorithm.[9]

Two microkinetic models were constructed: 1) assuming a barrierless catalyst activation; 2) including a catalyst activation process with an energy barrier estimated to fit the experimental conversions. We have not studied computationally the catalyst activation process due to the complexity and little experimental information obtained for this reaction.

1) *N-methylformanilide with EtOH poisoning assuming barrierless catalyst activation.*

The N-methylformanilide conversion *vs* time traces using Mo-5 as catalyst were obtained by running a microkinetic model described below. A concentration of 12.5 mM of Mo-5 was used. The elementary steps of the mechanism underlying the microkinetic model are given in Figure S7 and Figure S8, together with the $\Delta G^{\ddagger}$ values derived from the DFT calculations in Table S2.

2) *N-methylformanilide with EtOH poisoning assuming a catalyst activation.*

The N-methylformanilide conversion *vs* time traces using Mo-1a as catalyst were obtained by running a microkinetic model described below. A concentration of 12.5 mM of Mo-1a and 12.5 mM of $NaHBEt_3$ were used. The elementary steps of the mechanism underlying the microkinetic model are given in Figure S7 and Figure S8, together with the $\Delta G^{\ddagger}$ values

derived from the DFT calculations in Table S2. Mo-ts-6-7, was optimized to 25.1 kcal mol^{-1} to fit experimental conversions.

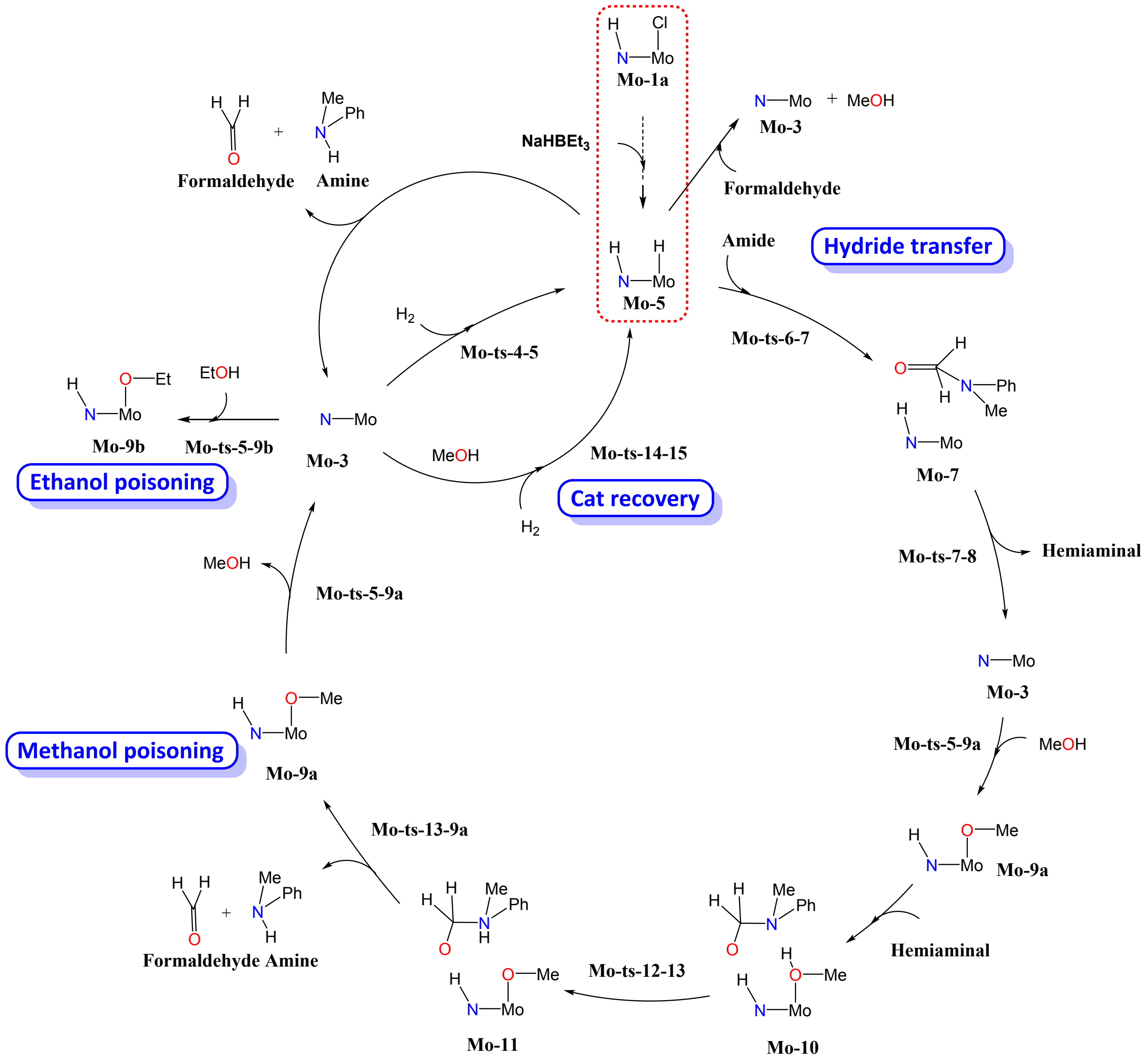

Figure S7. Reactions included in the microkinetic model of N-methylformanilide deaminative hydrogenation. In red dotted square, catalyst activation reaction.

Reaction 1: **Mo-5 + Amide** $\rightleftharpoons$ **Mo-7**

Reaction 2: **Mo-7** $\rightleftharpoons$ **Mo-3 + Hemiaminal**

Reaction 3: **Mo-9a + Hemiaminal** $\rightleftharpoons$ **Mo-10**

Reaction 4: **Mo-10** $\rightleftharpoons$ **Mo-11**

Reaction 5: **Mo-11** $\rightleftharpoons$ **Mo-9a + Formaldehyde + Amine**

Reaction 6: **Mo-9a** $\rightleftharpoons$ **Mo-3 + MeOH**

Reaction 7: **Mo-3 +** H_2 $\rightleftharpoons$ **Mo-5**

Reaction 8: **Mo-5 + Formaldehyde** $\rightleftharpoons$ **Mo-3 + MeOH**

Reaction 9: **Mo-3 + MeOH +** H_2 $\rightleftharpoons$ **Mo-5 + MeOH**

Reaction 10: **Mo-3 + EtOH** $\rightleftharpoons$ **Mo-9b**

Reaction 11: **Mo-5 + Amide** $\rightleftharpoons$ **Mo-3 + Amine + Formaldehyde**

Reaction 12: **Mo-1a +** $NaHBEt_3$ $\rightarrow$ **Mo-5**

Figure S8 Reactions used in the microkinetic simulations. In red dotted square, reaction included in the model to estimate the catalyst activation.

	ΔG‡ Forward (kcal mol-1)	ΔG‡ Backwards (kcal mol-1)
Reaction 1	13.1	6.5
Reaction 2	3.1	0.4
Reaction 3	7.4	0.1[a]
Reaction 4	5.9	0.2[b]
Reaction 5	0.2[b]	13.9
Reaction 6	14.2[c]	2.8
Reaction 7	18.6	20.3
Reaction 8	6.6	20.2
Reaction 9	11.6	13.3
Reaction 10	5.0[c]	18.3
Reaction 11	22.9	14.4

Table S2. Reactions and corresponding Gibbs energies (kcal mol^{-1}) used in the microkinetic model of the deaminative hydrogenation of DMF. In red, reaction included to fit the experimental conversions. [a]TS raised to 5.2 to get positive energies. [b] TSs raised to 11 kcal mol^{-1} to get positive energies. [c] Estimated low energy barrier.

Evaluation of the position of the Na cation

The cation location was determined by computing the energy of selected species (Mo-3, Mo-4, Mo-5, and Mo-9a) with the cation in different positions (interacting with two CO ligands, P_{2CO}; or interacting with CO and a lone pair, $P_{CO/LP}$; see scheme S9). The location yielding the lowest energy was the one used in the energy profiles, and is the one represented in the Schemes of the manuscript. In most cases, small energy differences (<2 kcal/mol) are obtained when comparing P_{2CO} and $P_{CO/LP}$ structures.

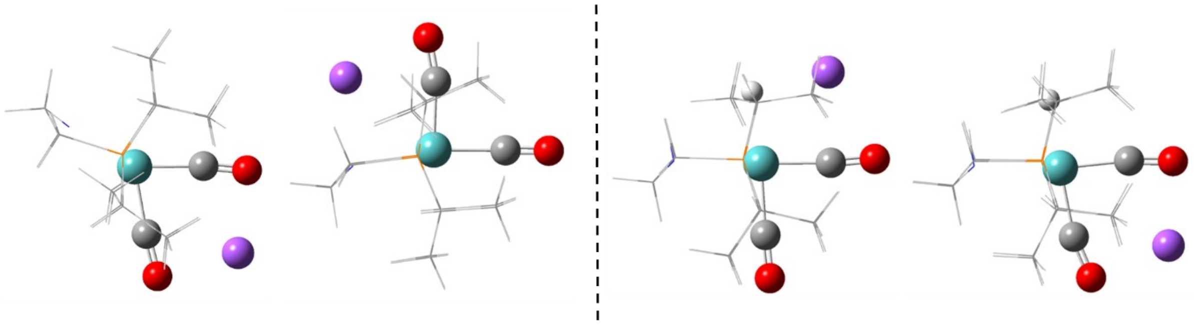

Mo-3 | Mo-5

	P_{2CO}	P_{CO}	$P_{CO/H}$	P_{2CO}
**ΔG**	0.0	1.0	0.0	1.1

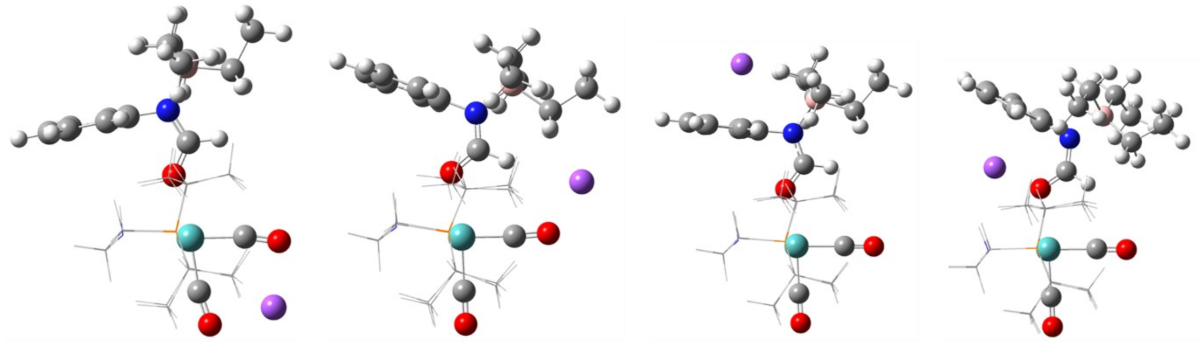

Mo-4

	P_{2CO}	P_{CO}	P_{LP}	P_{LP}
**ΔG**	0.0	4.0	17.3	19.3

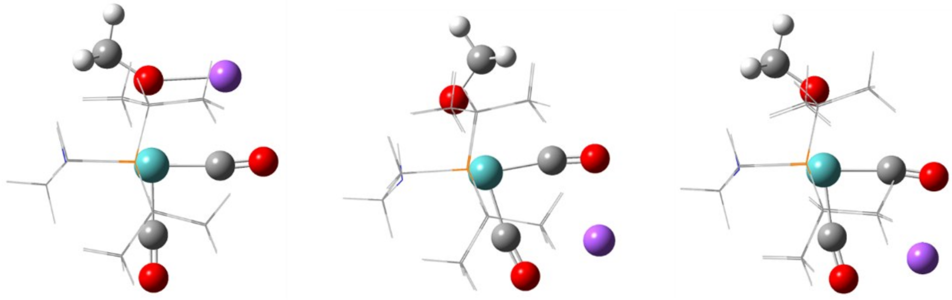

Mo-9a

	$P_{CO/LP}$	P_{2CO}	P_{2CO}
**ΔG**	0.0	1.9	7.9

Figure S9. Evaluation of multiple Na^{+} positions and their relative free energies (kcal mol^{-1}). PNP ligand depicted in a wireframe representation for more clarity. Legend: Mo (turquoise), C (grey), O (red), N (blue), H (white), Na (purple), P (orange)

Cartesian coordinates of optimized geometries, and corresponding corrected Gibbs energies.

Mo-1a

Free energy = -1332276.3 kcal mol-1

Atom	X	Y	Z
C	2.9667	11.4369	21.6566
H	2.0179	11.2285	22.1694
H	3.6238	11.9346	22.3851
C	2.7374	12.3541	20.4661
H	2.0212	11.9109	19.7594
H	2.3094	13.3131	20.8043
C	3.7853	13.5278	18.6088
H	3.3399	14.4673	18.9775
H	3.0543	13.0678	17.9282
C	5.0924	13.8178	17.8888
H	5.7780	14.3473	18.5670
H	4.9188	14.4775	17.0279
C	4.8249	9.4300	22.6507
H	5.3174	10.3894	22.8739
C	5.9256	8.4195	22.3477
H	6.5105	8.2289	23.2576
H	6.6155	8.7928	21.5822
H	5.5294	7.4547	22.0064
C	3.9790	9.0107	23.8457
H	3.5354	8.0160	23.7033
H	3.1671	9.7175	24.0619
H	4.6090	8.9542	24.7434
C	2.4518	8.6153	21.0365
H	2.0495	8.5262	22.0582
C	2.9922	7.2626	20.5858
H	2.1643	6.5586	20.4287
H	3.6680	6.8129	21.3220
H	3.5402	7.3461	19.6366
C	1.3257	9.0808	20.1206
H	1.6682	9.2074	19.0863
H	0.8692	10.0216	20.4519
H	0.5297	8.3246	20.1074
C	7.7481	12.7038	17.4701
H	7.8061	13.1768	18.4629
C	8.1752	13.7099	16.4096
H	9.1848	14.0786	16.6346
H	7.5129	14.5842	16.3604
H	8.2157	13.2570	15.4097

C	8.6611	11.4833	17.4997
H	8.5882	10.8803	16.5856
H	8.4373	10.8332	18.3533
H	9.7061	11.8078	17.5942
C	5.5327	12.0656	15.5764
H	5.9883	12.9365	15.0787
C	4.0291	12.1091	15.3291
H	3.8338	12.0251	14.2519
H	3.5693	13.0460	15.6666
H	3.5120	11.2739	15.8173
C	6.1378	10.7856	15.0105
H	5.8235	9.9045	15.5877
H	7.2335	10.8054	15.0033
H	5.8065	10.6373	13.9742
C	3.9841	9.7166	17.9068
C	6.1984	8.9899	18.6989
Cl	6.8480	11.7371	20.8012
Mo	5.1838	10.6163	19.1531
N	3.9897	12.5876	19.7251
O	3.3393	9.0900	17.1464
O	6.7357	8.0013	18.3784
P	3.8258	9.8715	21.1346
P	5.9399	12.2394	17.3873
H	4.6767	12.9947	20.3699

Mo-1c

Free energy = -1126719.4 kcal mol-1

Atom	X	Y	Z
C	5.3592	6.9515	-1.2530
H	5.9101	6.7508	-2.1826
H	5.0724	8.0155	-1.2801
C	6.2506	6.7073	-0.0451
H	6.5918	5.6630	-0.0135
H	7.1508	7.3459	-0.0968
C	6.3412	6.7451	2.4053
H	7.2405	7.3862	2.3720
H	6.6829	5.7009	2.3791
C	5.5403	7.0234	3.6680
H	5.2510	8.0870	3.6842
H	6.1594	6.8523	4.5597
C	2.6082	7.1215	-2.0559
H	2.7514	8.0550	-1.4854
C	2.9613	7.3915	-3.5125

H	2.7672	6.5119	-4.1410
H	4.0114	7.6788	-3.6531
H	2.3426	8.2081	-3.9114
C	1.1449	6.7163	-1.9127
H	0.4862	7.5554	-2.1798
H	0.9023	6.3977	-0.8908
H	0.8888	5.8870	-2.5848
C	4.0493	4.5486	-2.3233
H	4.1101	4.9884	-3.3320
C	2.8733	3.5802	-2.2593
H	3.0357	2.7353	-2.9426
H	1.9217	4.0503	-2.5345
H	2.7561	3.1759	-1.2446
C	5.3566	3.8171	-2.0415
H	5.3596	3.3673	-1.0413
H	6.2352	4.4685	-2.1301
H	5.4857	3.0014	-2.7661
C	2.8578	7.2070	4.6751
H	2.9511	8.1260	4.0715
C	1.3903	6.7920	4.6565
H	1.1934	5.9765	5.3645
H	1.0700	6.4498	3.6641
H	0.7492	7.6334	4.9569
C	3.3210	7.5162	6.0926
H	2.7303	8.3405	6.5172
H	4.3771	7.8111	6.1439
H	3.1812	6.6525	6.7566
C	4.3270	4.6457	4.8970
H	4.4604	5.1113	5.8869
C	3.1556	3.6708	4.9457
H	2.9640	3.2415	3.9528
H	2.2247	4.1430	5.2815
H	3.3751	2.8434	5.6345
C	5.6140	3.9128	4.5367
H	5.8006	3.1151	5.2689
H	6.4932	4.5694	4.5451
H	5.5459	3.4391	3.5500
C	1.8729	8.6988	1.3280
C	1.0547	9.8919	1.3531
H	1.6554	10.7796	1.1313
H	0.5967	10.0166	2.3406
H	0.2560	9.8145	0.6072
C	1.7865	4.7791	1.3748
C	4.3027	3.9401	1.2921
Mo	3.4906	5.7019	1.2990
N	5.5122	6.9551	1.2056

H	5.2104	7.9330	1.2016
N	2.4930	7.7143	1.3125
O	0.7476	4.2116	1.4199
O	4.7541	2.8427	1.2898
P	3.7674	5.9623	-1.1314
P	3.9484	6.0275	3.6942

Mo-3

Gibbs energy: -1144943.2 kcal mol-1

Atom	X	Y	Z
Mo	0.0002	0.2101	0.1437
P	-2.4134	-0.2170	0.3672
P	2.4140	-0.2166	0.3659
C	-3.2799	-1.2684	-0.9177
C	-2.4669	-1.2753	1.8902
C	-3.6300	1.1400	0.7965
C	0.0004	2.0925	0.3755
C	-0.0000	0.7527	-1.6940
C	3.2796	-1.2687	-0.9189
C	2.4685	-1.2742	1.8894
C	3.6308	1.1407	0.7937
C	-2.4726	-2.5382	-1.1651
C	-3.4762	-0.5127	-2.2262
H	-4.2618	-1.5474	-0.5021
C	-1.1812	-2.0837	2.0458
H	-3.3544	-1.9248	1.9232
H	-2.5460	-0.5648	2.7262
C	-5.0753	0.6801	0.9359
C	-3.5106	2.3442	-0.1316
H	-3.2617	1.4563	1.7864
O	0.0006	3.3070	0.4342
O	-0.0002	1.2207	-2.8159
C	2.4722	-2.5386	-1.1652
C	3.4752	-0.5136	-2.2280
H	4.2618	-1.5475	-0.5039
C	1.1831	-2.0829	2.0460
H	3.3562	-1.9235	1.9222
H	2.5479	-0.5633	2.7251
C	3.5105	2.3444	-0.1349
C	5.0762	0.6809	0.9322
H	3.2632	1.4574	1.7837
H	-2.3732	-3.1586	-0.2667
H	-2.9578	-3.1519	-1.9365
H	-1.4602	-2.2952	-1.5192
H	-2.5259	-0.0870	-2.5796
H	-3.8438	-1.1937	-3.0064
H	-4.2033	0.3033	-2.1409

N	0.0007	-1.3878	1.5589
H	-1.2923	-3.0684	1.5441
H	-1.0619	-2.3287	3.1208
H	-5.5047	0.3938	-0.0339
H	-5.6980	1.4930	1.3348
H	-5.1805	-0.1750	1.6158
H	-3.8038	2.1053	-1.1621
H	-4.1660	3.1557	0.2144
H	-2.4831	2.7276	-0.1438
H	2.3736	-3.1587	-0.2665
H	2.9567	-3.1526	-1.9368
H	1.4594	-2.2958	-1.5184
H	2.5246	-0.0881	-2.5810
H	3.8422	-1.1951	-3.0081
H	4.2024	0.3024	-2.1435
H	1.2944	-3.0680	1.5451
H	1.0641	-2.3270	3.1213
H	3.8023	2.1049	-1.1656
H	4.1666	3.1559	0.2099
H	2.4832	2.7281	-0.1460
H	5.5050	0.3945	-0.0379
H	5.6991	1.4940	1.3304
H	5.1821	-0.1739	1.6122
Na	-0.0005	3.3467	-1.8991

Mo-3-alt1

Gibbs energy: -1144942.2 kcal mol-1

Atom	X	Y	Z
Mo	0.0004	-0.0073	0.2471
P	2.4120	-0.1218	-0.2000
P	-2.4112	-0.1241	-0.1996
C	3.3714	-1.6708	0.2297
C	2.4795	-0.1068	-2.0724
C	3.5288	1.3180	0.2232
C	-0.0005	1.8324	0.5534
C	0.0009	-0.2789	2.1963
C	-3.3693	-1.6738	0.2305
C	-2.4790	-0.1094	-2.0720
C	-3.5292	1.3147	0.2234
C	2.5647	-2.8836	-0.2237
C	3.6434	-1.7553	1.7266
H	4.3257	-1.6461	-0.3216
C	1.1874	-0.6568	-2.6718
H	3.3547	-0.6475	-2.4620
H	2.6118	0.9484	-2.3689
C	4.9775	1.1499	-0.2156
C	3.4214	1.7311	1.6868

H	3.0728	2.1258	-0.3767
O	-0.0012	3.0457	0.4974
O	0.0013	-0.4446	3.3669
C	-2.5626	-2.8860	-0.2243
C	-3.6393	-1.7588	1.7278
H	-4.3242	-1.6494	-0.3195
C	-1.1864	-0.6583	-2.6715
H	-3.3538	-0.6510	-2.4614
H	-2.6124	0.9456	-2.3688
C	-3.4223	1.7279	1.6871
C	-4.9778	1.1455	-0.2154
H	-3.0739	2.1230	-0.3764
H	2.4067	-2.9096	-1.3087
H	3.0783	-3.8137	0.0550
H	1.5776	-2.8970	0.2653
H	2.7181	-1.6201	2.3051
H	4.0550	-2.7413	1.9830
H	4.3629	-1.0035	2.0696
N	0.0003	-0.1530	-1.9954
H	1.2204	-1.7679	-2.6501
H	1.1661	-0.4044	-3.7547
H	5.4866	0.3720	0.3692
H	5.5355	2.0836	-0.0584
H	5.0714	0.8864	-1.2771
H	3.9453	1.0299	2.3481
H	3.8769	2.7196	1.8349
H	2.3770	1.7821	2.0187
H	-2.4066	-2.9119	-1.3096
H	-3.0750	-3.8165	0.0554
H	-1.5745	-2.8988	0.2628
H	-2.7134	-1.6233	2.3052
H	-4.0502	-2.7450	1.9844
H	-4.3587	-1.0074	2.0719
H	-1.2182	-1.7695	-2.6493
H	-1.1656	-0.4063	-3.7544
H	-3.9464	1.0267	2.3482
H	-3.8780	2.7164	1.8351
H	-2.3780	1.7791	2.0193
H	-5.4863	0.3671	0.3693
H	-5.5366	2.0787	-0.0581
H	-5.0714	0.8820	-1.2770
Na	-0.0020	2.2588	-1.8821

Mo-3-1atm

Gibbs energy: -1144946.1 kcal mol-1

Atom	X	Y	Z
Mo	-0.0000	0.0416	0.1876

P	-2.4138	-0.4382	0.2285
P	2.4136	-0.4382	0.2288
C	-3.2799	-0.9127	-1.3626
C	-2.4678	-2.0020	1.2256
C	-3.6304	0.6485	1.1476
C	0.0002	1.6890	1.1273
C	0.0003	1.2508	-1.2987
C	3.2796	-0.9136	-1.3622
C	2.4676	-2.0015	1.2266
C	3.6304	0.6489	1.1472
C	-2.4726	-1.9890	-2.0804
C	-3.4758	0.2892	-2.2786
H	-4.2620	-1.3302	-1.0870
C	-1.1821	-2.8080	1.0578
H	-3.3553	-2.6138	1.0054
H	-2.5470	-1.6688	2.2710
C	-5.0757	0.1706	1.0985
C	-3.5107	2.1174	0.7556
H	-3.2624	0.5586	2.1830
O	0.0004	2.7870	1.6498
O	0.0006	2.1153	-2.1534
C	2.4722	-1.9902	-2.0793
C	3.4756	0.2878	-2.2789
H	4.2617	-1.3311	-1.0865
C	1.1821	-2.8078	1.0590
H	3.3553	-2.6133	1.0068
H	2.5468	-1.6678	2.2718
C	3.5105	2.1177	0.7545
C	5.0757	0.1711	1.0980
H	3.2626	0.5595	2.1826
H	-2.3735	-2.9079	-1.4906
H	-2.9576	-2.2577	-3.0289
H	-1.4601	-1.6285	-2.3131
H	-2.5253	0.8181	-2.4402
H	-3.8431	-0.0383	-3.2612
H	-4.2029	1.0093	-1.8854
N	-0.0000	-1.9784	0.8772
H	-1.2932	-3.5232	0.2152
H	-1.0632	-3.4486	1.9553
H	-5.5049	0.2806	0.0932
H	-5.6985	0.7670	1.7798
H	-5.1813	-0.8804	1.3961
H	-3.8035	2.2944	-0.2874
H	-4.1661	2.7329	1.3876
H	-2.4831	2.4757	0.8924
H	2.3733	-2.9089	-1.4892
H	2.9569	-2.2593	-3.0279
H	1.4595	-1.6298	-2.3119
H	2.5252	0.8167	-2.4407
H	3.8428	-0.0402	-3.2613

H	4.2028	1.0080	-1.8860
H	1.2936	-3.5236	0.2170
H	1.0628	-3.4476	1.9569
H	3.8026	2.2941	-0.2888
H	4.1665	2.7334	1.3857
H	2.4832	2.4762	0.8919
H	5.5048	0.2808	0.0926
H	5.6986	0.7677	1.7791
H	5.1814	-0.8798	1.3958
Na	0.0001	3.7234	-0.4877

Mo-ts-3-5

Gibbs energy: -1145659.3 kcal mol-1

Atom	X	Y	Z
Mo	-0.0037	-0.0192	0.1687
P	-2.4200	-0.5326	0.1515
P	2.4135	-0.5407	0.1551
C	-3.6127	0.2589	-1.0517
C	-2.4606	-2.3360	-0.3257
C	-3.3318	-0.5711	1.7911
C	-0.0151	1.6859	1.0440
C	-0.0020	1.0854	-1.3854
C	3.6125	0.2424	-1.0480
C	2.4469	-2.3446	-0.3206
C	3.3237	-0.5818	1.7953
C	-3.1878	-0.0128	-2.4897
C	-3.7235	1.7569	-0.7970
H	-4.5950	-0.2111	-0.8845
C	-1.2036	-2.7138	-1.1042
H	-3.3688	-2.5905	-0.8903
H	-2.4910	-2.9073	0.6140
C	-4.8116	-0.9178	1.6925
C	-3.1014	0.6906	2.6145
H	-2.8118	-1.3954	2.3068
O	-0.0266	2.8275	1.4514
O	0.0011	1.9378	-2.2490
C	3.1824	-0.0227	-2.4857
C	3.7390	1.7386	-0.7902
H	4.5906	-0.2372	-0.8833
C	1.1901	-2.7183	-1.1014
H	3.3552	-2.6034	-0.8831
H	2.4730	-2.9151	0.6197
C	3.1056	0.6857	2.6130
C	4.7999	-0.9443	1.6996
H	2.7945	-1.3985	2.3137
H	-3.1743	-1.0834	-2.7270
H	-3.8899	0.4679	-3.1850

H	-2.1861	0.3871	-2.6949
H	-2.7275	2.2245	-0.8185
H	-4.3362	2.2362	-1.5731
H	-4.1779	1.9899	0.1732
N	-0.0066	-2.2529	-0.4434
H	-1.2671	-2.2960	-2.1344
H	-1.1870	-3.8174	-1.2276
H	-5.3912	-0.0972	1.2475
H	-5.2280	-1.0952	2.6937
H	-4.9954	-1.8216	1.0970
H	-3.5174	1.5856	2.1324
H	-3.5854	0.5951	3.5966
H	-2.0326	0.8733	2.7769
H	3.1565	-1.0928	-2.7239
H	3.8888	0.4506	-3.1817
H	2.1848	0.3881	-2.6889
H	2.7459	2.2133	-0.7912
H	4.3407	2.2170	-1.5752
H	4.2119	1.9648	0.1727
H	1.2573	-2.3014	-2.1319
H	1.1700	-3.8219	-1.2244
H	3.5415	1.5720	2.1324
H	3.5786	0.5856	3.6000
H	2.0382	0.8858	2.7646
H	5.3886	-0.1318	1.2517
H	5.2137	-1.1218	2.7020
H	4.9747	-1.8525	1.1082
H	-0.0067	-1.9165	0.9789
H	-0.0059	-1.3698	1.7420
Na	0.0641	3.6542	-0.7020

Mo-4

Gibbs energy: -1560952.6 kcal mol-1

Atom	X	Y	Z
B	4.2894	0.1903	-0.7936
C	-1.3103	2.3046	2.0479
C	-1.8838	1.0287	2.6485
C	-1.7701	-1.4269	2.5401
C	-1.0887	-2.5857	1.8262
C	0.0598	3.2292	-0.3076
C	0.2433	4.6217	0.2795
C	0.3011	3.2252	-1.8132
C	-2.9019	3.5800	-0.0632
C	-4.1364	3.1833	0.7377
C	-3.2426	3.6918	-1.5440
C	0.3414	-3.1903	-0.5907
C	0.5241	-3.1370	-2.1029

C	0.6700	-4.5755	-0.0498
C	-2.5791	-3.7764	-0.4422
C	-3.7915	-3.6739	0.4759
C	-3.0161	-3.6255	-1.8959
C	-1.8103	-0.0056	-2.3040
C	-3.5642	-0.1485	-0.5352
C	1.6532	0.0959	-0.6340
C	2.7381	0.1300	1.3990
C	2.6503	1.3267	2.1136
C	2.4951	1.3116	3.4983
C	2.4249	0.1006	4.1852
C	2.5252	-1.0961	3.4767
C	2.6810	-1.0815	2.0923
C	5.1050	-1.1398	-0.3069
C	4.5192	-2.4731	-0.7665
C	4.0179	0.1962	-2.4050
C	5.3083	0.2412	-3.2214
C	5.0366	1.5519	-0.2867
C	4.3852	2.8640	-0.7182
Mo	-1.6730	-0.0593	-0.3996
N	-1.3583	-0.1484	1.9411
N	2.8256	0.1425	-0.0286
O	-2.0717	0.0082	-3.4842
O	-4.7473	-0.1920	-0.8112
O	0.5516	0.0476	-0.0252
P	-1.5195	2.3519	0.1866
P	-1.2994	-2.4807	-0.0319
H	-0.2274	2.3350	2.2455
H	-1.7448	3.1893	2.5327
H	-1.6350	0.9750	3.7232
H	-2.9798	1.0036	2.5607
H	-1.5161	-1.4476	3.6150
H	-2.8646	-1.4938	2.4588
H	-1.4309	-3.5474	2.2308
H	-0.0058	-2.5296	2.0178
H	0.8077	2.5583	0.1458
H	0.1051	4.6481	1.3684
H	1.2595	4.9862	0.0715
H	-0.4525	5.3460	-0.1670
H	0.0665	2.2540	-2.2684
H	1.3554	3.4531	-2.0266
H	-0.3003	3.9889	-2.3242
H	-2.5391	4.5537	0.3034
H	-3.9516	3.1605	1.8185
H	-4.5056	2.1936	0.4368
H	-4.9444	3.9070	0.5634
H	-4.0834	4.3820	-1.6965
H	-3.5342	2.7082	-1.9429
H	-2.4022	4.0501	-2.1495
H	1.0427	-2.4652	-0.1448

H	-0.0410	-3.9357	-2.6032
H	1.5826	-3.2851	-2.3595
H	0.2049	-2.1773	-2.5303
H	0.0203	-5.3455	-0.4885
H	1.7031	-4.8459	-0.3107
H	0.5827	-4.6420	1.0423
H	-2.1052	-4.7611	-0.3012
H	-3.5497	-3.8776	1.5257
H	-4.5541	-4.4039	0.1717
H	-4.2486	-2.6768	0.4156
H	-2.1808	-3.6426	-2.6054
H	-3.7126	-4.4284	-2.1731
H	-3.5384	-2.6667	-2.0247
H	5.1969	-1.1433	0.7944
H	6.1467	-1.0656	-0.6657
H	4.9903	-3.3463	-0.2902
H	3.4385	-2.5318	-0.5523
H	4.6226	-2.6092	-1.8530
H	3.4450	-0.6950	-2.7228
H	3.3927	1.0577	-2.7057
H	5.9092	1.1305	-2.9822
H	5.9469	-0.6308	-3.0191
H	5.1258	0.2597	-4.3059
H	5.1365	1.5405	0.8138
H	6.0782	1.5357	-0.6526
H	4.8370	3.7514	-0.2498
H	3.3100	2.8813	-0.4705
H	4.4501	3.0111	-1.8062
H	2.7546	-2.0158	1.5349
H	2.6978	2.2729	1.5742
H	2.4291	2.2526	4.0417
H	2.3059	0.0892	5.2667
H	2.4833	-2.0483	4.0028
H	-0.3324	-0.1016	1.9567
H	1.6732	0.1024	-1.7329
Na	-4.3565	-0.0147	-3.0836

Mo-4-alt1

Gibbs energy: -1560948.6 kcal mol-1

Atom	X	Y	Z
B	4.0443	-0.4564	-0.8172
C	-1.3439	2.6780	1.9318
C	-2.2451	1.6122	2.5414
C	-2.5635	-0.8256	2.7148
C	-1.9850	-2.1653	2.2762
C	0.4738	3.1591	-0.2573
C	0.8269	4.4873	0.3982

C	0.7895	3.1875	-1.7488
C	-2.4016	3.8333	-0.5860
C	-3.7366	3.8285	0.1514
C	-2.6416	3.6274	-2.0794
C	-0.3475	-3.3643	0.2193
C	0.1238	-3.4447	-1.2297
C	-0.4393	-4.7526	0.8368
C	-3.3125	-3.3751	-0.0449
C	-4.6169	-2.8221	0.5178
C	-3.4130	-3.5171	-1.5596
C	-1.2012	-0.0773	-2.0875
C	-3.4851	0.3040	-0.7704
C	1.5140	-0.2607	-0.1327
C	3.0225	0.0075	1.6130
C	3.1571	1.3062	2.1064
C	3.3579	1.5232	3.4672
C	3.4260	0.4445	4.3469
C	3.2931	-0.8530	3.8564
C	3.0910	-1.0713	2.4954
C	4.7878	-1.8396	-0.3759
C	3.9773	-3.1238	-0.5257
C	3.4294	-0.5524	-2.3443
C	4.5015	-0.7356	-3.4167
C	5.0584	0.8109	-0.6661
C	4.5289	2.1613	-1.1400
Mo	-1.6381	0.0931	-0.2546
N	-1.8106	0.2778	2.1015
N	2.7870	-0.2168	0.2187
O	-0.8420	-0.1935	-3.2463
O	-4.6198	0.4318	-1.0976
O	0.5181	-0.1139	0.6174
P	-1.2547	2.5084	0.0692
P	-1.8680	-2.2787	0.4081
H	-0.3185	2.5277	2.3061
H	-1.6515	3.6830	2.2513
H	-2.2258	1.6842	3.6433
H	-3.2896	1.7405	2.2226
H	-2.5421	-0.7470	3.8163
H	-3.6121	-0.7226	2.4003
H	-2.5637	-2.9938	2.7076
H	-0.9584	-2.2563	2.6657
H	1.0875	2.3677	0.2053
H	0.6981	4.4699	1.4876
H	1.8794	4.7387	0.1996
H	0.2222	5.3136	-0.0007
H	0.4374	2.2787	-2.2575
H	1.8728	3.2809	-1.9108
H	0.3185	4.0497	-2.2405
H	-1.9078	4.8056	-0.4206
H	-3.6382	4.0532	1.2203

H	-4.2422	2.8597	0.0444
H	-4.4017	4.5895	-0.2790
H	-3.3171	4.4039	-2.4642
H	-3.1086	2.6508	-2.2636
H	-1.7214	3.6647	-2.6725
H	0.3993	-2.7772	0.7808
H	-0.4917	-4.1391	-1.8163
H	1.1586	-3.8128	-1.2766
H	0.0731	-2.4633	-1.7224
H	-1.1286	-5.3975	0.2743
H	0.5451	-5.2421	0.8163
H	-0.7731	-4.7337	1.8821
H	-3.1249	-4.3618	0.4092
H	-4.6134	-2.7576	1.6129
H	-5.4509	-3.4795	0.2364
H	-4.8350	-1.8253	0.1138
H	-2.5500	-4.0336	-1.9958
H	-4.3101	-4.0905	-1.8305
H	-3.4863	-2.5306	-2.0390
H	5.1315	-1.7479	0.6694
H	5.7202	-1.9392	-0.9588
H	4.4844	-4.0072	-0.1111
H	2.9989	-3.0500	-0.0217
H	3.7675	-3.3508	-1.5825
H	2.7272	-1.4101	-2.3956
H	2.8620	0.3809	-2.5465
H	5.2207	0.0935	-3.4088
H	5.0767	-1.6551	-3.2459
H	4.1085	-0.8000	-4.4458
H	5.3794	0.8975	0.3865
H	5.9895	0.5822	-1.2133
H	5.1923	3.0013	-0.8875
H	3.5440	2.3851	-0.6996
H	4.3926	2.1870	-2.2322
H	2.9821	-2.0827	2.1069
H	3.1037	2.1469	1.4158
H	3.4641	2.5402	3.8396
H	3.5860	0.6141	5.4095
H	3.3475	-1.7015	4.5355
H	-0.8216	0.1681	2.3475
H	1.3336	-0.4446	-1.2005
Na	1.1980	-0.5176	-3.9928

Mo-4-alt2

Gibbs energy: -1560935.3 kcal mol-1

Atom	X	Y	Z
B	4.3422	-0.0797	-1.0702
C	-1.1688	2.5918	2.0571

C	-1.7834	1.3929	2.7667
C	-1.7815	-1.0649	2.8699
C	-1.1499	-2.3148	2.2721
C	0.2306	3.3009	-0.3486
C	0.4301	4.7289	0.1413
C	0.4566	3.2070	-1.8536
C	-2.7159	3.6859	-0.1962
C	-3.9467	3.4410	0.6700
C	-3.0895	3.5882	-1.6717
C	0.2733	-3.2166	-0.0488
C	0.4691	-3.3227	-1.5566
C	0.5199	-4.5576	0.6298
C	-2.6716	-3.6060	0.0769
C	-3.8876	-3.3670	0.9653
C	-3.0846	-3.5422	-1.3913
C	-1.5793	-0.0796	-2.0835
C	-3.4458	0.0207	-0.2070
C	1.7460	-0.0124	-0.5308
C	3.1599	-0.1434	1.2964
C	3.2360	1.0480	2.0304
C	3.4303	1.0195	3.4122
C	3.5489	-0.2015	4.0804
C	3.4574	-1.3927	3.3577
C	3.2616	-1.3646	1.9755
C	5.2356	-1.4279	-0.7567
C	4.5875	-2.7445	-1.1821
C	3.8531	-0.0830	-2.6257
C	4.9979	-0.0740	-3.6367
C	5.1696	1.3067	-0.7353
C	4.4535	2.5979	-1.1293
Mo	-1.5295	0.0339	-0.1514
N	-1.2669	0.1385	2.1992
N	3.0113	-0.1107	-0.1163
O	-1.6230	-0.1645	-3.2677
O	-4.6383	0.0119	-0.2465
O	0.7652	0.0392	0.2398
P	-1.3625	2.4543	0.1903
P	-1.3320	-2.3414	0.3999
H	-0.0865	2.6127	2.2649
H	-1.5828	3.5282	2.4565
H	-1.5768	1.4451	3.8519
H	-2.8757	1.3803	2.6432
H	-1.5836	-1.0208	3.9569
H	-2.8726	-1.0705	2.7356
H	-1.5501	-3.2188	2.7516
H	-0.0677	-2.2985	2.4818
H	0.9778	2.6517	0.1417
H	0.2761	4.8385	1.2228
H	1.4521	5.0709	-0.0827
H	-0.2534	5.4240	-0.3656

H	0.2081	2.2138	-2.2505
H	1.5064	3.4231	-2.1011
H	-0.1548	3.9401	-2.3964
H	-2.3225	4.6922	0.0239
H	-3.7447	3.5608	1.7419
H	-4.3526	2.4345	0.5060
H	-4.7359	4.1591	0.4071
H	-3.8960	4.2953	-1.9107
H	-3.4434	2.5761	-1.9118
H	-2.2481	3.8045	-2.3404
H	1.0115	-2.4910	0.3336
H	-0.1594	-4.1143	-1.9868
H	1.5120	-3.5797	-1.7951
H	0.2230	-2.3859	-2.0734
H	-0.1649	-5.3319	0.2576
H	1.5401	-4.9121	0.4164
H	0.4110	-4.5113	1.7214
H	-2.2550	-4.6017	0.3042
H	-3.6647	-3.4738	2.0343
H	-4.6738	-4.0957	0.7240
H	-4.3087	-2.3671	0.7967
H	-2.2485	-3.7045	-2.0816
H	-3.8490	-4.3021	-1.6057
H	-3.5100	-2.5576	-1.6275
H	5.5151	-1.5704	0.3098
H	6.2065	-1.3258	-1.2720
H	5.1628	-3.6296	-0.8763
H	3.5757	-2.8454	-0.7594
H	4.4721	-2.7976	-2.2722
H	3.2156	-0.9605	-2.8325
H	3.2080	0.7862	-2.8394
H	5.6397	0.8117	-3.5223
H	5.6510	-0.9522	-3.5297
H	4.6367	-0.0718	-4.6742
H	5.4484	1.4376	0.3330
H	6.1410	1.2656	-1.2578
H	4.9754	3.5037	-0.7898
H	3.4318	2.6331	-0.7178
H	4.3508	2.6785	-2.2189
H	3.1793	-2.2915	1.4081
H	3.1375	1.9967	1.5038
H	3.4805	1.9532	3.9685
H	3.6951	-0.2242	5.1578
H	3.5283	-2.3492	3.8713
H	-0.2442	0.1422	2.2764
H	1.6017	0.0164	-1.6205
Na	5.8407	-0.0767	1.8848

Mo-4-alt3

Gibbs energy: -1560933.3 kcal mol-1

Atom	X	Y	Z
B	-4.0119	0.3011	-1.5181
C	2.1314	-2.3752	2.0537
C	2.8077	-1.1050	2.5509
C	2.6552	1.3580	2.6050
C	1.7825	2.5337	2.1949
C	0.2494	-3.3658	0.1019
C	0.3192	-4.7244	0.7854
C	-0.2504	-3.4985	-1.3317
C	3.1181	-3.3557	-0.5712
C	4.4888	-3.1229	0.0555
C	3.1852	-3.0783	-2.0712
C	-0.2069	3.4004	0.2973
C	-0.6419	3.6671	-1.1396
C	-0.3129	4.6722	1.1292
C	2.6397	3.7758	-0.3711
C	4.0242	3.7012	0.2641
C	2.7551	3.5703	-1.8799
C	1.2911	0.1424	-2.0778
C	3.4566	0.2557	-0.5607
C	-1.5968	0.1449	-0.4043
C	-3.3374	-0.7764	0.8281
C	-3.3572	-2.1752	0.8943
C	-3.7448	-2.8200	2.0695
C	-4.1222	-2.0747	3.1890
C	-4.1214	-0.6801	3.1208
C	-3.7302	-0.0328	1.9474
C	-5.0169	1.3713	-0.8036
C	-4.4078	2.7188	-0.4246
C	-3.1982	0.9325	-2.7781
C	-4.1202	1.3293	-3.9305
C	-4.8103	-1.0665	-1.9064
C	-4.0032	-2.1143	-2.6686
Mo	1.5948	0.1154	-0.1621
N	2.0165	0.0896	2.1912
N	-2.8851	-0.1233	-0.3485
O	1.1374	0.1635	-3.2514
O	4.6218	0.3400	-0.7985
O	-0.7349	-0.1051	0.5026
P	1.7899	-2.2827	0.1993
P	1.4514	2.5173	0.3465
H	1.1603	-2.4976	2.5705
H	2.7185	-3.2589	2.3399
H	2.9691	-1.1512	3.6426
H	3.7928	-0.9865	2.0810
H	2.8299	1.3626	3.6956
H	3.6365	1.4010	2.1149

H	2.2117	3.4807	2.5495
H	0.7989	2.4364	2.6852
H	-0.4967	-2.7437	0.6287
H	0.6648	-4.6705	1.8259
H	-0.6742	-5.1982	0.7921
H	0.9907	-5.4087	0.2505
H	-0.2973	-2.5292	-1.8466
H	-1.2551	-3.9457	-1.3496
H	0.3988	-4.1620	-1.9185
H	2.8264	-4.4058	-0.4033
H	4.5276	-3.3839	1.1203
H	4.8065	-2.0789	-0.0617
H	5.2360	-3.7462	-0.4544
H	3.8998	-3.7624	-2.5491
H	3.5256	-2.0516	-2.2573
H	2.2206	-3.1926	-2.5788
H	-0.8867	2.6420	0.7275
H	-0.0690	4.4960	-1.5773
H	-1.7011	3.9576	-1.1715
H	-0.5126	2.7916	-1.7906
H	0.3611	5.4569	0.7589
H	-1.3342	5.0756	1.0683
H	-0.0888	4.5138	2.1911
H	2.2120	4.7719	-0.1688
H	4.0171	3.9148	1.3400
H	4.6858	4.4413	-0.2063
H	4.4810	2.7159	0.1050
H	1.7882	3.5857	-2.3950
H	3.3834	4.3565	-2.3207
H	3.2220	2.6019	-2.1010
H	-5.4937	0.9179	0.0850
H	-5.8593	1.5501	-1.4940
H	-5.0897	3.3579	0.1562
H	-3.4868	2.6028	0.1728
H	-4.1212	3.2914	-1.3184
H	-2.6227	1.8291	-2.4865
H	-2.4426	0.2247	-3.1665
H	-4.6920	0.4707	-4.3104
H	-4.8570	2.0840	-3.6184
H	-3.5700	1.7513	-4.7832
H	-5.2534	-1.5275	-1.0044
H	-5.6861	-0.7828	-2.5156
H	-4.5309	-3.0732	-2.7815
H	-3.0403	-2.3275	-2.1766
H	-3.7498	-1.7690	-3.6804
H	-3.7186	1.0547	1.8881
H	-3.0593	-2.7476	0.0161
H	-3.7589	-3.9075	2.1063
H	-4.4328	-2.5776	4.1018
H	-4.4320	-0.0913	3.9814

H	1.1391	0.0502	2.7171
H	-1.2587	0.6307	-1.3268
Na	-1.2151	-1.2016	2.4137

Mo-4-gas-singlet

Gibbs energy: --1561133.2 kcal mol-1

Atom	X	Y	Z
B	4.3704	-0.0580	-1.0330
C	-0.7800	2.4695	2.0347
C	-1.3385	1.2701	2.7882
C	-1.3823	-1.1877	2.8312
C	-0.8633	-2.4324	2.1237
C	0.3361	3.1634	-0.5245
C	0.6465	4.5838	-0.0713
C	0.3974	3.0356	-2.0431
C	-2.5472	3.7056	0.0466
C	-3.6924	3.4411	1.0182
C	-3.0653	3.7371	-1.3867
C	0.2520	-3.2290	-0.4069
C	0.3308	-3.1381	-1.9273
C	0.5238	-4.6461	0.0803
C	-2.6473	-3.6854	0.1622
C	-3.8010	-3.3490	1.1010
C	-3.1421	-3.7654	-1.2774
C	-1.9763	-0.0355	-2.0562
C	-3.4731	0.0178	-0.0703
C	1.7408	-0.0455	-0.7504
C	2.9183	0.0659	1.2258
C	2.9429	1.3014	1.8758
C	2.8506	1.3702	3.2645
C	2.7330	0.2055	4.0208
C	2.7293	-1.0311	3.3767
C	2.8246	-1.1005	1.9889
C	5.1401	-1.3972	-0.5055
C	4.4732	-2.7245	-0.8630
C	4.0192	-0.1210	-2.6256
C	5.2730	-0.1691	-3.4977
C	5.1871	1.2947	-0.6264
C	4.5611	2.6077	-1.0942
Mo	-1.5800	-0.0079	-0.1864
N	-0.9725	0.0218	2.1037
N	2.9369	-0.0023	-0.2006
O	-2.4196	-0.0495	-3.1809
O	-4.6833	0.0290	-0.2006
O	0.6655	-0.0300	-0.0906
P	-1.2131	2.4116	0.2117
P	-1.2829	-2.4200	0.2962

H	0.3189	2.4460	2.0987
H	-1.1068	3.4081	2.5025
H	-0.9562	1.2675	3.8248
H	-2.4365	1.3069	2.8404
H	-1.0037	-1.1605	3.8690
H	-2.4811	-1.1861	2.8797
H	-1.2282	-3.3417	2.6202
H	0.2350	-2.4484	2.1958
H	1.1037	2.4892	-0.1110
H	0.6281	4.6962	1.0206
H	1.6485	4.8758	-0.4146
H	-0.0630	5.3062	-0.4986
H	0.0770	2.0435	-2.3846
H	1.4268	3.1999	-2.3908
H	-0.2345	3.7843	-2.5392
H	-2.0886	4.6770	0.2925
H	-3.3732	3.4793	2.0666
H	-4.1460	2.4578	0.8373
H	-4.4755	4.2000	0.8892
H	-3.8780	4.4684	-1.4885
H	-3.4576	2.7466	-1.6625
H	-2.2876	3.9926	-2.1150
H	1.0324	-2.5636	-0.0028
H	-0.3147	-3.8821	-2.4125
H	1.3591	-3.3363	-2.2597
H	0.0375	-2.1470	-2.2952
H	-0.1964	-5.3606	-0.3421
H	1.5234	-4.9670	-0.2435
H	0.4884	-4.7362	1.1738
H	-2.2189	-4.6569	0.4575
H	-3.4993	-3.3433	2.1553
H	-4.5987	-4.0963	0.9958
H	-4.2297	-2.3659	0.8660
H	-2.3630	-4.0844	-1.9786
H	-3.9799	-4.4704	-1.3597
H	-3.4921	-2.7755	-1.6071
H	5.2861	-1.3434	0.5883
H	6.1634	-1.3919	-0.9178
H	4.9460	-3.5944	-0.3839
H	3.4078	-2.7336	-0.5719
H	4.4926	-2.9048	-1.9475
H	3.4003	-1.0044	-2.8700
H	3.4149	0.7502	-2.9410
H	5.9061	0.7154	-3.3402
H	5.8929	-1.0450	-3.2595
H	5.0433	-0.2166	-4.5713
H	5.3428	1.3308	0.4666
H	6.2058	1.2191	-1.0431
H	5.0642	3.4996	-0.6929
H	3.4980	2.6755	-0.8028

H	4.5797	2.6940	-2.1900
H	2.8282	-2.0650	1.4806
H	3.0345	2.2105	1.2815
H	2.8730	2.3409	3.7568
H	2.6628	0.2597	5.1050
H	2.6570	-1.9492	3.9575
H	0.0483	0.0017	1.9861
H	1.7142	-0.0966	-1.8478
Na	-4.5768	-0.0057	-2.4659

Mo-4-gas-triplet

Gibbs energy: -1561088.2 kcal mol-1

Atom	X	Y	Z
B	4.4070	-0.2704	-0.9750
C	-0.7729	2.5252	2.0186
C	-1.3474	1.3435	2.7800
C	-1.4173	-1.0835	2.8811
C	-0.9360	-2.3587	2.2100
C	0.3694	3.1437	-0.5412
C	0.7033	4.5671	-0.1139
C	0.4595	2.9780	-2.0539
C	-2.5151	3.7724	0.0130
C	-3.6917	3.4935	0.9422
C	-2.9803	3.8302	-1.4377
C	0.1147	-3.2762	-0.2996
C	0.2014	-3.2332	-1.8209
C	0.3278	-4.6900	0.2263
C	-2.7998	-3.6207	0.3271
C	-3.9351	-3.1768	1.2435
C	-3.2944	-3.7541	-1.1090
C	-1.5372	-0.0909	-2.3803
C	-3.4481	0.0820	-0.2574
C	1.7756	-0.1290	-0.7265
C	2.9411	0.1832	1.2302
C	3.0505	1.4766	1.7439
C	2.9331	1.7043	3.1140
C	2.7032	0.6429	3.9874
C	2.6166	-0.6537	3.4804
C	2.7401	-0.8818	2.1122
C	5.0758	-1.6017	-0.3043
C	4.3242	-2.9098	-0.5448
C	4.0708	-0.4659	-2.5598
C	5.3282	-0.6775	-3.4019
C	5.3167	1.0527	-0.6880
C	4.8014	2.3531	-1.3025
Mo	-1.5821	0.0129	-0.2883
N	-0.9656	0.0885	2.1317

N	2.9723	-0.0412	-0.1791
O	-2.1629	-0.1129	-3.4147
O	-4.6627	0.1219	-0.4023
O	0.6996	-0.0319	-0.0765
P	-1.2052	2.4622	0.2068
P	-1.3880	-2.4072	0.4018
H	0.3254	2.4905	2.0842
H	-1.0933	3.4749	2.4675
H	-0.9959	1.3680	3.8278
H	-2.4473	1.3807	2.7997
H	-1.0523	-1.0487	3.9243
H	-2.5168	-1.0468	2.9175
H	-1.3139	-3.2459	2.7356
H	0.1627	-2.3970	2.2614
H	1.1074	2.4581	-0.0943
H	0.6746	4.7036	0.9750
H	1.7159	4.8281	-0.4504
H	0.0165	5.2958	-0.5664
H	0.1606	1.9727	-2.3787
H	1.4959	3.1387	-2.3813
H	-0.1648	3.7090	-2.5844
H	-2.0566	4.7354	0.2907
H	-3.4049	3.5122	2.0005
H	-4.1407	2.5141	0.7296
H	-4.4682	4.2569	0.8018
H	-3.8001	4.5512	-1.5520
H	-3.3509	2.8426	-1.7526
H	-2.1801	4.1182	-2.1284
H	0.9189	-2.6290	0.0889
H	-0.5054	-3.9299	-2.2903
H	1.2095	-3.5314	-2.1393
H	0.0066	-2.2291	-2.2203
H	-0.4142	-5.3870	-0.1871
H	1.3181	-5.0527	-0.0805
H	0.2797	-4.7531	1.3209
H	-2.4136	-4.5924	0.6751
H	-3.6348	-3.1401	2.2975
H	-4.7731	-3.8821	1.1685
H	-4.3062	-2.1828	0.9603
H	-2.5314	-4.1539	-1.7861
H	-4.1673	-4.4180	-1.1557
H	-3.5976	-2.7686	-1.4939
H	5.2093	-1.4504	0.7820
H	6.1027	-1.7062	-0.6941
H	4.7289	-3.7588	0.0254
H	3.2570	-2.8178	-0.2747
H	4.3452	-3.1956	-1.6064
H	3.3999	-1.3281	-2.7310
H	3.5248	0.4068	-2.9650
H	6.0170	0.1747	-3.3182

H	5.8860	-1.5649	-3.0708
H	5.1077	-0.8127	-4.4700
H	5.4561	1.1898	0.3992
H	6.3337	0.8590	-1.0692
H	5.3605	3.2425	-0.9768
H	3.7400	2.5254	-1.0526
H	4.8513	2.3265	-2.4003
H	2.6791	-1.8928	1.7087
H	3.2251	2.3050	1.0579
H	3.0229	2.7185	3.4992
H	2.6116	0.8213	5.0566
H	2.4580	-1.4942	4.1546
H	0.0549	0.0538	2.0233
H	1.7479	-0.2930	-1.8143
Na	-4.2714	0.0300	-2.6320

Mo-5

Gibbs energy: -1145679.5 kcal mol-1

Atom	X	Y	Z
Mo	-0.0057	0.3248	0.0005
P	2.3996	0.0461	-0.3930
P	-2.4092	0.0389	-0.3988
C	3.5855	-0.5435	0.9364
C	2.4503	-1.3045	-1.6924
C	3.3935	1.3676	-1.2955
C	-0.0091	1.7510	1.2707
C	-0.0057	-0.8998	1.5292
C	-3.5963	-0.5554	0.9274
C	-2.4524	-1.3110	-1.6992
C	-3.4052	1.3579	-1.3026
C	3.3687	-2.0104	1.2898
C	3.4279	0.3195	2.1854
H	4.6066	-0.4281	0.5373
C	1.2282	-2.2050	-1.6024
H	3.3741	-1.8975	-1.6496
H	2.4451	-0.7950	-2.6691
C	4.7411	0.9313	-1.8569
C	3.5556	2.6315	-0.4583
H	2.7171	1.5944	-2.1364
O	-0.0112	2.7116	2.0004
O	-0.0058	-1.6029	2.4843
C	-3.3761	-2.0220	1.2799
C	-3.4440	0.3068	2.1777
H	-4.6169	-0.4426	0.5262
C	-1.2281	-2.2083	-1.6061
H	-3.3748	-1.9064	-1.6596
H	-2.4455	-0.8007	-2.6755

C	-3.5748	2.6199	-0.4640
C	-4.7494	0.9174	-1.8689
H	-2.7271	1.5885	-2.1411
H	3.5424	-2.6848	0.4421
H	4.0654	-2.3075	2.0858
H	2.3531	-2.1819	1.6686
H	2.4129	0.2275	2.5936
H	4.1358	-0.0016	2.9621
H	3.6032	1.3851	1.9965
N	-0.0010	-1.4004	-1.6498
H	1.2165	-2.7624	-0.6547
H	1.2400	-2.9480	-2.4205
H	5.4545	0.6862	-1.0585
H	5.1906	1.7459	-2.4429
H	4.6659	0.0604	-2.5191
H	4.3525	2.5143	0.2888
H	3.8377	3.4858	-1.0897
H	2.6417	2.8899	0.0964
H	-3.5461	-2.6961	0.4313
H	-4.0738	-2.3218	2.0742
H	-2.3610	-2.1910	1.6609
H	-2.4296	0.2173	2.5880
H	-4.1527	-0.0173	2.9525
H	-3.6220	1.3721	1.9895
H	-1.2175	-2.7660	-0.6585
H	-1.2356	-2.9510	-2.4244
H	-4.3736	2.4984	0.2803
H	-3.8582	3.4740	-1.0949
H	-2.6637	2.8811	0.0940
H	-5.4641	0.6675	-1.0732
H	-5.2005	1.7314	-2.4544
H	-4.6687	0.0482	-2.5327
H	-0.0004	-0.8650	-2.5199
H	-0.0056	1.4583	-1.6083
Na	-0.0105	3.3625	-0.6095

Mo-5-alt1

Gibbs energy: -1145678.5 kcal mol-1

Atom	X	Y	Z
Mo	0.0049	0.0001	-0.1672
P	-2.3960	-0.1772	0.3323
P	2.3985	-0.1745	0.3434
C	-3.6597	-0.9049	-0.8504
C	-2.4464	-1.4098	1.7470
C	-3.2837	1.2790	1.1156
C	0.0338	1.4000	-1.4650
C	0.0131	-1.1822	-1.6992

C	3.6546	-0.9062	-0.8443
C	2.4477	-1.3988	1.7645
C	3.2908	1.2838	1.1190
C	-3.2975	-2.3325	-1.2397
C	-3.7867	-0.0348	-2.0943
H	-4.6259	-0.9226	-0.3209
C	-1.2288	-2.3237	1.7335
H	-3.3722	-2.0015	1.7438
H	-2.4463	-0.8244	2.6795
C	-4.7444	1.0423	1.4748
C	-3.1049	2.5581	0.3069
H	-2.7011	1.4000	2.0432
O	0.0606	2.1874	-2.3859
O	0.0105	-1.7124	-2.7946
C	3.3251	-2.3541	-1.1863
C	3.7296	-0.0706	-2.1159
H	4.6319	-0.8826	-0.3365
C	1.2343	-2.3182	1.7456
H	3.3765	-1.9857	1.7732
H	2.4367	-0.8077	2.6934
C	3.1094	2.5619	0.3096
C	4.7532	1.0473	1.4718
H	2.7131	1.4072	2.0495
H	-3.2736	-3.0121	-0.3787
H	-4.0430	-2.7299	-1.9428
H	-2.3172	-2.3756	-1.7320
H	-2.8002	0.1106	-2.5610
H	-4.4441	-0.5094	-2.8361
H	-4.1937	0.9599	-1.8791
N	0.0012	-1.5269	1.6565
H	-1.2410	-2.9888	0.8578
H	-1.2246	-2.9637	2.6347
H	-5.3786	0.9878	0.5792
H	-5.1253	1.8750	2.0827
H	-4.8980	0.1209	2.0522
H	-3.6736	2.5356	-0.6324
H	-3.4654	3.4231	0.8810
H	-2.0496	2.7271	0.0612
H	3.3554	-3.0131	-0.3098
H	4.0567	-2.7440	-1.9079
H	2.3303	-2.4400	-1.6424
H	2.7411	-0.0241	-2.5971
H	4.4320	-0.5176	-2.8332
H	4.0527	0.9607	-1.9325
H	1.2562	-2.9867	0.8728
H	1.2257	-2.9545	2.6493
H	3.6607	2.5324	-0.6399
H	3.4874	3.4252	0.8749
H	2.0517	2.7401	0.0821
H	5.3838	0.9979	0.5732

H	5.1354	1.8776	2.0820
H	4.9104	0.1233	2.0441
H	-0.0040	-0.8492	2.4216
H	-0.0277	1.2112	1.2987
Na	-0.0380	0.3565	-3.8240

Mo-5-1atm

Gibbs energy: -1145682.4 kcal mol-1

Atom	X	Y	Z
Mo	-0.0001	0.1467	-0.0901
P	-2.4045	-0.1074	0.3250
P	2.4044	-0.1071	0.3250
C	-3.5910	-0.7901	-0.9583
C	-2.4515	-1.3640	1.7156
C	-3.3993	1.2725	1.1343
C	-0.0004	1.4807	-1.4568
C	0.0000	-1.1817	-1.5295
C	3.5908	-0.7908	-0.9579
C	2.4512	-1.3628	1.7164
C	3.3995	1.2733	1.1332
C	-3.3723	-2.2778	-1.2087
C	-3.4363	-0.0162	-2.2648
H	-4.6118	-0.6486	-0.5670
C	-1.2281	-2.2668	1.6872
H	-3.3744	-1.9599	1.7156
H	-2.4459	-0.7875	2.6544
C	-4.7455	0.8745	1.7266
C	-3.5644	2.4746	0.2112
H	-2.7222	1.5585	1.9564
O	-0.0007	2.3880	-2.2519
O	0.0000	-1.9498	-2.4332
C	3.3725	-2.2788	-1.2069
C	3.4357	-0.0183	-2.2652
H	4.6117	-0.6488	-0.5670
C	1.2282	-2.2662	1.6879
H	3.3745	-1.9582	1.7174
H	2.4447	-0.7856	2.6548
C	3.5661	2.4738	0.2083
C	4.7450	0.8753	1.7271
H	2.7220	1.5609	1.9544
H	-3.5440	-2.8916	-0.3158
H	-4.0696	-2.6308	-1.9812
H	-2.3570	-2.4738	-1.5760
H	-2.4216	-0.1350	-2.6669
H	-4.1446	-0.3919	-3.0163
H	-3.6130	1.0598	-2.1506
N	-0.0001	-1.4590	1.6767

H	-1.2167	-2.8890	0.7807
H	-1.2377	-2.9508	2.5552
H	-5.4595	0.5732	0.9482
H	-5.1956	1.7274	2.2549
H	-4.6682	0.0521	2.4479
H	-4.3620	2.3044	-0.5249
H	-3.8470	3.3704	0.7816
H	-2.6516	2.6949	-0.3614
H	3.5445	-2.8918	-0.3135
H	4.0697	-2.6324	-1.9792
H	2.3571	-2.4755	-1.5738
H	2.4209	-0.1377	-2.6669
H	4.1439	-0.3947	-3.0164
H	3.6123	1.0578	-2.1521
H	1.2174	-2.8887	0.7816
H	1.2379	-2.9500	2.5561
H	4.3641	2.3018	-0.5268
H	3.8489	3.3704	0.7776
H	2.6539	2.6941	-0.3654
H	5.4591	0.5715	0.9498
H	5.1955	1.7289	2.2538
H	4.6664	0.0545	2.4501
H	-0.0005	-0.8641	2.5073
H	0.0000	1.3898	1.4356
Na	0.0008	3.2196	0.3062

Mo-9a

Gibbs energy: -1217535.0 kcal mol-1

Atom	X	Y	Z
C	-2.4594	0.0944	2.0263
H	-3.3796	0.5777	2.3820
H	-2.4589	-0.9244	2.4454
C	-1.2302	0.8336	2.5347
H	-1.2064	1.8665	2.1601
H	-1.2449	0.8853	3.6381
C	1.2276	0.8323	2.5370
H	1.2405	0.8835	3.6404
H	1.2055	1.8653	2.1628
C	2.4571	0.0923	2.0304
H	2.4551	-0.9267	2.4491
H	3.3771	0.5747	2.3879
C	-3.3573	-1.7101	-0.0283
H	-2.6355	-2.4328	0.3882
C	-3.5748	-2.0599	-1.4964
H	-3.8550	-3.1167	-1.6060
H	-2.6823	-1.8701	-2.1098
H	-4.3911	-1.4670	-1.9317

C	-4.6695	-1.8054	0.7396
H	-5.4067	-1.0761	0.3757
H	-4.5463	-1.6457	1.8173
H	-5.1174	-2.8012	0.6097
C	-3.5901	1.1989	-0.4987
H	-4.6145	0.8267	-0.3298
C	-3.3578	1.3891	-1.9962
H	-4.0807	2.1103	-2.4022
H	-3.4539	0.4607	-2.5708
H	-2.3487	1.7802	-2.1824
C	-3.4319	2.5369	0.2159
H	-2.4135	2.9296	0.0993
H	-3.6648	2.4837	1.2864
H	-4.1145	3.2755	-0.2267
C	3.3606	-1.7106	-0.0230
H	2.6384	-2.4350	0.3898
C	4.6699	-1.8060	0.7499
H	5.1199	-2.8007	0.6191
H	4.5420	-1.6494	1.8275
H	5.4074	-1.0746	0.3911
C	3.5848	-2.0575	-1.4909
H	4.4029	-1.4636	-1.9213
H	2.6957	-1.8659	-2.1088
H	3.8657	-3.1140	-1.6015
C	3.5922	1.1978	-0.4924
H	4.6162	0.8248	-0.3228
C	3.4342	2.5351	0.2233
H	4.1180	3.2736	-0.2176
H	3.6655	2.4807	1.2940
H	2.4164	2.9290	0.1057
C	3.3617	1.3895	-1.9899
H	2.3528	1.7806	-2.1771
H	3.4587	0.4616	-2.5653
H	4.0852	2.1111	-2.3943
C	0.0021	1.8449	-0.5834
C	0.0011	-0.2883	-2.1963
Mo	0.0007	-0.0639	-0.3045
N	-0.0012	0.1695	2.0651
O	0.0027	3.0244	-0.7440
O	0.0016	-0.5471	-3.3758
P	-2.4115	-0.0995	0.1612
P	2.4124	-0.1010	0.1652
H	-0.0021	-0.7843	2.4349
O	-0.0023	-2.3888	0.0735
C	-0.0009	-2.9767	1.3054
H	-0.8885	-2.7179	1.9351
H	0.8903	-2.7215	1.9315
H	-0.0035	-4.0858	1.2538
Na	0.0159	-2.7662	-2.0339

Mo-9a-alt1

Gibbs energy: -1217527.1 kcal mol-1

Atom	X	Y	Z
C	-2.4629	0.0883	2.0931
H	-3.3765	0.5887	2.4435
H	-2.4904	-0.9348	2.4990
C	-1.2296	0.8083	2.6247
H	-1.2217	1.8608	2.3073
H	-1.2302	0.7976	3.7294
C	1.2301	0.8083	2.6249
H	1.2306	0.7977	3.7296
H	1.2223	1.8608	2.3074
C	2.4635	0.0882	2.0934
H	2.4905	-0.9349	2.4991
H	3.3770	0.5884	2.4443
C	-3.2509	-1.7171	-0.0339
H	-2.6993	-2.3540	0.6758
C	-2.9708	-2.2692	-1.4268
H	-3.3878	-3.2821	-1.5203
H	-1.8901	-2.3232	-1.6038
H	-3.4249	-1.6571	-2.2181
C	-4.7322	-1.7566	0.3150
H	-5.3368	-1.1988	-0.4138
H	-4.9465	-1.3475	1.3118
H	-5.0967	-2.7932	0.3027
C	-3.6737	1.2141	-0.3158
H	-4.6467	0.9025	0.0968
C	-3.7630	1.2546	-1.8362
H	-4.4266	2.0667	-2.1645
H	-4.1455	0.3206	-2.2639
H	-2.7681	1.4267	-2.2753
C	-3.3371	2.5952	0.2335
H	-2.3636	2.9467	-0.1317
H	-3.3145	2.6208	1.3301
H	-4.0972	3.3207	-0.0889
C	3.2517	-1.7170	-0.0333
H	2.6980	-2.3539	0.6747
C	4.7323	-1.7576	0.3185
H	5.0963	-2.7944	0.3065
H	4.9449	-1.3489	1.3158
H	5.3385	-1.1999	-0.4090
C	2.9739	-2.2683	-1.4270
H	3.4306	-1.6565	-2.2171
H	1.8936	-2.3211	-1.6063
H	3.3899	-3.2816	-1.5199
C	3.6756	1.2142	-0.3144
H	4.6481	0.9029	0.0995

C	3.3380	2.5954	0.2340
H	4.0982	3.3210	-0.0879
H	3.3142	2.6214	1.3305
H	2.3648	2.9465	-0.1324
C	3.7669	1.2541	-1.8348
H	2.7725	1.4250	-2.2753
H	4.1510	0.3203	-2.2615
H	4.4301	2.0668	-2.1625
C	0.0001	1.9590	-0.5981
C	-0.0004	0.0036	-2.1772
Mo	0.0005	0.0872	-0.2686
N	0.0003	0.1896	2.1025
O	0.0001	3.1211	-0.9793
O	-0.0017	0.1300	-3.3815
P	-2.4148	-0.0616	0.2223
P	2.4161	-0.0613	0.2226
H	0.0003	-0.7948	2.3830
O	0.0023	-2.1269	0.0872
C	0.0002	-2.8421	1.2359
H	-0.8896	-2.6743	1.9059
H	0.8882	-2.6749	1.9087
H	0.0002	-3.9390	1.0509
Na	0.0035	2.4362	-3.1729

Mo-9a-alt2

Gibbs energy: -1217533.1 kcal mol-1

Atom	X	Y	Z
C	-2.4399	-0.3619	-2.0844
H	-3.3714	-0.8522	-2.3975
H	-2.3984	0.6081	-2.6023
C	-1.2270	-1.1838	-2.4970
H	-1.2594	-2.1933	-2.0615
H	-1.2101	-1.3026	-3.5957
C	1.2322	-1.1792	-2.4983
H	1.2146	-1.2981	-3.5969
H	1.2689	-2.1884	-2.0626
C	2.4423	-0.3525	-2.0870
H	2.3964	0.6173	-2.6048
H	3.3754	-0.8391	-2.4012
C	-3.3414	1.6640	-0.2559
H	-2.6174	2.3074	-0.7830
C	-3.5268	2.2114	1.1548
H	-3.8251	3.2680	1.1158
H	-2.6064	2.1469	1.7496
H	-4.3218	1.6737	1.6919
C	-4.6638	1.6714	-1.0127
H	-5.4181	1.0403	-0.5220

H	-4.5667	1.3325	-2.0510
H	-5.0754	2.6902	-1.0427
C	-3.6109	-1.1870	0.5095
H	-4.6305	-0.8450	0.2674
C	-3.4348	-1.2090	2.0264
H	-4.2236	-1.8138	2.4951
H	-3.4573	-0.2118	2.4812
H	-2.4690	-1.6728	2.2720
C	-3.4227	-2.5957	-0.0424
H	-2.3920	-2.9447	0.1112
H	-3.6562	-2.6705	-1.1110
H	-4.0863	-3.2962	0.4837
C	3.3375	1.6770	-0.2593
H	2.6102	2.3173	-0.7857
C	4.6590	1.6899	-1.0175
H	5.0662	2.7105	-1.0483
H	4.5624	1.3503	-2.0556
H	5.4167	1.0623	-0.5275
C	3.5221	2.2251	1.1511
H	4.3199	1.6907	1.6873
H	2.6026	2.1568	1.7469
H	3.8160	3.2830	1.1118
C	3.6197	-1.1729	0.5055
H	4.6375	-0.8266	0.2621
C	3.4366	-2.5823	-0.0463
H	4.1037	-3.2801	0.4790
H	3.6692	-2.6561	-1.1151
H	2.4076	-2.9357	0.1085
C	3.4455	-1.1958	2.0226
H	2.4820	-1.6638	2.2694
H	3.4643	-0.1985	2.4775
H	4.2375	-1.7972	2.4903
C	0.0058	-1.6882	1.1028
C	0.0016	0.6472	2.0581
Mo	0.0018	0.0225	0.2549
N	0.0016	-0.5353	-2.0285
O	0.0086	-2.6826	1.8104
O	0.0019	0.8862	3.2469
P	-2.4175	0.0371	-0.2560
P	2.4203	0.0463	-0.2585
H	-0.0004	0.4537	-2.3143
O	-0.0024	1.9151	-0.9366
C	-0.0047	3.1494	-0.3393
H	0.8791	3.3352	0.3149
H	-0.0069	3.9706	-1.0884
H	-0.8885	3.3315	0.3160
Na	0.0070	-1.3741	3.7137

Mo-9a-Li

Gibbs energy: -1120433.4 kcal mol-1

Atom	X	Y	Z
C	2.4623	-0.7684	2.0061
H	3.3792	-1.3304	2.2299
H	2.4701	0.1165	2.6623
C	1.2306	-1.6045	2.3205
H	1.2081	-2.5188	1.7114
H	1.2398	-1.9168	3.3797
C	-1.2278	-1.6033	2.3222
H	-1.2367	-1.9139	3.3820
H	-1.2059	-2.5187	1.7147
C	-2.4594	-0.7675	2.0068
H	-2.4666	0.1188	2.6610
H	-3.3763	-1.3288	2.2324
C	3.3650	1.4810	0.4631
H	2.6670	2.0610	1.0894
C	3.5337	2.2259	-0.8560
H	3.8389	3.2654	-0.6745
H	2.6104	2.2390	-1.4522
H	4.3140	1.7673	-1.4788
C	4.7021	1.3659	1.1841
H	5.4313	0.7912	0.5968
H	4.6186	0.8922	2.1696
H	5.1370	2.3634	1.3405
C	3.5930	-1.2278	-0.7074
H	4.6164	-0.9204	-0.4359
C	3.3843	-1.0288	-2.2068
H	4.1114	-1.6274	-2.7729
H	3.4945	0.0149	-2.5233
H	2.3775	-1.3517	-2.5029
C	3.4168	-2.7005	-0.3530
H	2.4013	-3.0466	-0.5847
H	3.6258	-2.9159	0.7019
H	4.1092	-3.3097	-0.9499
C	-3.3642	1.4779	0.4598
H	-2.6660	2.0590	1.0849
C	-4.7010	1.3639	1.1817
H	-5.1368	2.3614	1.3354
H	-4.6162	0.8934	2.1686
H	-5.4299	0.7866	0.5967
C	-3.5349	2.2207	-0.8602
H	-4.3198	1.7643	-1.4789
H	-2.6157	2.2273	-1.4636
H	-3.8341	3.2623	-0.6807
C	-3.5909	-1.2332	-0.7054
H	-4.6144	-0.9271	-0.4328
C	-3.4120	-2.7053	-0.3497
H	-4.1052	-3.3161	-0.9442

H	-3.6184	-2.9195	0.7060
H	-2.3966	-3.0505	-0.5832
C	-3.3844	-1.0350	-2.2052
H	-2.3761	-1.3526	-2.5018
H	-3.5010	0.0077	-2.5227
H	-4.1087	-1.6381	-2.7700
C	0.0010	-1.8277	-0.9733
C	0.0030	0.5974	-2.0165
Mo	0.0004	-0.0527	-0.2144
N	0.0015	-0.8476	2.0173
O	0.0006	-2.9197	-1.4414
O	0.0043	1.0655	-3.1242
P	2.4166	-0.1249	0.2445
P	-2.4147	-0.1281	0.2438
H	0.0025	-0.0072	2.6017
O	-0.0096	2.1258	0.6812
C	0.0001	2.4475	2.0111
H	0.8934	2.0623	2.5560
H	-0.8843	2.0610	2.5698
H	0.0004	3.5419	2.1793
Li	-0.0502	2.5618	-0.9994

Mo-9a-K

Gibbs energy: -1492153.5 kcal mol-1

Atom	X	Y	Z
C	2.4587	-0.7552	1.9970
H	3.3772	-1.3147	2.2211
H	2.4602	0.1277	2.6555
C	1.2271	-1.5943	2.3037
H	1.2026	-2.5007	1.6827
H	1.2398	-1.9209	3.3590
C	-1.2267	-1.5930	2.3048
H	-1.2393	-1.9185	3.3605
H	-1.2029	-2.5002	1.6849
C	-2.4576	-0.7533	1.9975
H	-2.4575	0.1309	2.6542
H	-3.3766	-1.3114	2.2234
C	3.3993	1.4808	0.4514
H	2.6886	2.1016	1.0226
C	3.6478	2.1614	-0.8902
H	3.9611	3.2049	-0.7451
H	2.7582	2.1458	-1.5354
H	4.4532	1.6631	-1.4477
C	4.7013	1.3581	1.2325
H	5.4179	0.6953	0.7278
H	4.5538	0.9776	2.2499
H	5.1870	2.3406	1.3232

C	3.5709	-1.2221	-0.7242
H	4.6010	-0.9056	-0.4886
C	3.3187	-1.0548	-2.2211
H	4.0319	-1.6622	-2.7958
H	3.4138	-0.0174	-2.5622
H	2.3046	-1.3872	-2.4793
C	3.4138	-2.6901	-0.3417
H	2.3940	-3.0441	-0.5397
H	3.6551	-2.8891	0.7096
H	4.0922	-3.3049	-0.9494
C	-3.4020	1.4780	0.4467
H	-2.6909	2.1031	1.0127
C	-4.7012	1.3559	1.2327
H	-5.1912	2.3368	1.3167
H	-4.5489	0.9845	2.2527
H	-5.4162	0.6854	0.7358
C	-3.6570	2.1517	-0.8972
H	-4.4649	1.6503	-1.4482
H	-2.7710	2.1334	-1.5472
H	-3.9704	3.1957	-0.7559
C	-3.5692	-1.2274	-0.7227
H	-4.5998	-0.9119	-0.4877
C	-3.4101	-2.6945	-0.3373
H	-4.0887	-3.3111	-0.9429
H	-3.6498	-2.8915	0.7147
H	-2.3902	-3.0480	-0.5361
C	-3.3173	-1.0628	-2.2200
H	-2.3022	-1.3927	-2.4773
H	-3.4157	-0.0265	-2.5635
H	-4.0285	-1.6738	-2.7933
C	0.0007	-1.7820	-0.9429
C	0.0001	0.6387	-2.0184
Mo	0.0000	0.0018	-0.2176
N	0.0005	-0.8320	2.0144
O	0.0011	-2.8940	-1.3725
O	-0.0000	1.0942	-3.1341
P	2.4132	-0.1042	0.2386
P	-2.4130	-0.1058	0.2378
H	0.0011	0.0002	2.6091
O	-0.0009	2.1332	0.7757
C	0.0057	2.3631	2.1193
H	0.8961	1.9472	2.6569
H	-0.8815	1.9507	2.6650
H	0.0090	3.4460	2.3722
K	-0.0117	3.3653	-1.3806

Mo-9b

Gibbs energy: -1242180.6 kcal mol-1

Atom	X	Y	Z
C	-2.4614	-0.3524	-2.1096
H	-3.3804	-0.8317	-2.4737
H	-2.4629	0.6728	-2.5123
C	-1.2313	-1.0821	-2.6288
H	-1.2089	-2.1221	-2.2737
H	-1.2430	-1.1133	-3.7330
C	1.2256	-1.0813	-2.6278
H	1.2387	-1.1117	-3.7320
H	1.2031	-2.1215	-2.2735
C	2.4550	-0.3518	-2.1065
H	2.4578	0.6733	-2.5094
H	3.3744	-0.8317	-2.4686
C	-3.3551	1.4197	-0.0285
H	-2.6378	2.1418	-0.4536
C	-3.5493	1.7680	1.4431
H	-3.8096	2.8293	1.5595
H	-2.6528	1.5596	2.0443
H	-4.3703	1.1880	1.8867
C	-4.6770	1.5258	-0.7780
H	-5.4160	0.8044	-0.4028
H	-4.5700	1.3638	-1.8571
H	-5.1133	2.5260	-0.6430
C	-3.6077	-1.4975	0.3828
H	-4.6284	-1.1238	0.1971
C	-3.4043	-1.7007	1.8821
H	-4.1260	-2.4348	2.2666
H	-3.5253	-0.7793	2.4634
H	-2.3938	-2.0802	2.0842
C	-3.4343	-2.8278	-0.3425
H	-2.4185	-3.2221	-0.2097
H	-3.6446	-2.7630	-1.4170
H	-4.1266	-3.5708	0.0767
C	3.3421	1.4214	-0.0244
H	2.6276	2.1407	-0.4588
C	4.6699	1.5256	-0.7636
H	5.1021	2.5282	-0.6331
H	4.5723	1.3543	-1.8422
H	5.4079	0.8095	-0.3764
C	3.5240	1.7771	1.4470
H	4.3424	1.2007	1.9002
H	2.6236	1.5685	2.0423
H	3.7823	2.8392	1.5607
C	3.5984	-1.4960	0.3868
H	4.6187	-1.1222	0.1994
C	3.4249	-2.8274	-0.3363
H	4.1189	-3.5691	0.0825
H	3.6332	-2.7638	-1.4113
H	2.4098	-3.2226	-0.2012

C	3.3967	-1.6970	1.8865
H	2.3860	-2.0748	2.0906
H	3.5201	-0.7750	2.4666
H	4.1178	-2.4316	2.2712
C	-0.0045	-2.1652	0.4618
C	-0.0009	-0.0851	2.1334
Mo	-0.0047	-0.2507	0.2341
N	-0.0033	-0.4274	-2.1440
O	-0.0044	-3.3489	0.5891
O	0.0074	0.1325	3.3208
P	-2.4165	-0.1936	-0.2406
P	2.4061	-0.1936	-0.2375
H	-0.0035	0.5344	-2.4931
O	-0.0029	2.0765	-0.0830
C	-0.0039	2.7150	-1.2910
H	-0.8911	2.4439	-1.9203
H	0.8805	2.4414	-1.9232
Na	0.0262	2.4073	2.0373
C	-0.0014	4.2289	-1.1392
H	-0.0024	4.7427	-2.1104
H	-0.8901	4.5555	-0.5795
H	0.8904	4.5531	-0.5829

Mo-9b-alt1

Gibbs energy: -1242171.4 kcal mol-1

Atom	X	Y	Z
C	2.3805	0.7932	2.1099
H	3.2842	0.4641	2.6415
H	2.4083	1.8939	2.0942
C	1.1323	0.3353	2.8524
H	1.1197	-0.7581	2.9681
H	1.1123	0.7741	3.8663
C	-1.3236	0.3704	2.8140
H	-1.3441	0.8558	3.8061
H	-1.3163	-0.7160	2.9814
C	-2.5416	0.7900	2.0006
H	-2.5594	1.8873	1.9143
H	-3.4671	0.5007	2.5181
C	3.1744	1.6851	-0.5184
H	2.4931	2.4959	-0.2159
C	3.0878	1.5895	-2.0368
H	3.3294	2.5624	-2.4875
H	2.0746	1.3170	-2.3555
H	3.8000	0.8587	-2.4447
C	4.5882	2.0029	-0.0522
H	5.3077	1.2465	-0.3970
H	4.6750	2.0739	1.0399

H	4.9182	2.9655	-0.4677
C	3.6852	-1.1129	0.3109
H	4.6241	-0.6441	0.6442
C	3.8699	-1.6506	-1.1127
H	4.1338	-2.7187	-1.1009
H	4.6722	-1.1354	-1.6539
H	2.9618	-1.5119	-1.7229
C	3.3404	-2.2306	1.2880
H	2.4111	-2.7475	1.0105
H	3.2061	-1.8643	2.3134
H	4.1495	-2.9734	1.3130
C	-3.3319	1.4447	-0.7218
H	-2.8823	2.3595	-0.3022
C	-4.8405	1.5195	-0.5303
H	-5.2353	2.4345	-0.9933
H	-5.1333	1.5375	0.5286
H	-5.3550	0.6738	-1.0072
C	-2.9338	1.3877	-2.1936
H	-3.2298	0.4423	-2.6690
H	-1.8473	1.4986	-2.2986
H	-3.4247	2.2010	-2.7478
C	-3.6639	-1.3230	0.3167
H	-4.6589	-0.8880	0.5026
C	-3.3451	-2.2935	1.4479
H	-4.0856	-3.1053	1.4561
H	-3.3793	-1.8194	2.4367
H	-2.3554	-2.7498	1.3197
C	-3.6771	-2.0534	-1.0206
H	-2.6744	-2.4296	-1.2664
H	-4.0008	-1.4140	-1.8508
H	-4.3598	-2.9137	-0.9806
C	-0.0374	-2.0183	0.3556
C	-0.0872	-0.9564	-1.8521
Mo	-0.0428	-0.1750	-0.1069
N	-0.0783	0.7053	2.1029
O	0.0141	-3.2370	0.4560
O	-0.1009	-1.6571	-2.8529
P	2.3712	0.2203	0.3226
P	-2.4590	0.1048	0.2572
H	-0.0610	1.7179	1.9557
O	0.0340	1.9815	-0.6879
C	-0.1662	3.1240	0.0136
H	0.6383	3.3255	0.7830
H	-1.1150	3.1323	0.6204
Na	1.0282	-3.3101	-1.6370
C	-0.2108	4.3437	-0.8985
H	-1.0343	4.2427	-1.6191
H	-0.3479	5.2783	-0.3348
H	0.7235	4.4126	-1.4734

Mo-9b-alt2

Gibbs energy: -1242176.5 kcal mol-1

Atom	X	Y	Z
C	-2.4408	-1.5331	1.6666
H	-3.3737	-1.4682	2.2424
H	-2.3953	-2.5479	1.2433
C	-1.2302	-1.3293	2.5668
H	-1.2673	-0.3540	3.0741
H	-1.2130	-2.1057	3.3532
C	1.2300	-1.3295	2.5667
H	1.2126	-2.1059	3.3531
H	1.2673	-0.3543	3.0740
C	2.4404	-1.5336	1.6664
H	2.3948	-2.5484	1.2433
H	3.3734	-1.4689	2.2423
C	-3.3308	-1.4041	-1.0641
H	-2.6045	-2.2183	-1.2244
C	-3.5078	-0.6586	-2.3817
H	-3.7944	-1.3585	-3.1787
H	-2.5865	-0.1515	-2.6975
H	-4.3080	0.0924	-2.3123
C	-4.6535	-1.9977	-0.5967
H	-5.4060	-1.2195	-0.4056
H	-4.5549	-2.6000	0.3143
H	-5.0676	-2.6542	-1.3750
C	-3.6222	0.9891	0.6585
H	-4.6385	0.5769	0.5477
C	-3.4559	2.1758	-0.2877
H	-4.2366	2.9263	-0.1014
H	-3.4988	1.8964	-1.3469
H	-2.4825	2.6521	-0.1032
C	-3.4369	1.4577	2.0973
H	-2.4101	1.8094	2.2678
H	-3.6595	0.6758	2.8329
H	-4.1099	2.3009	2.3068
C	3.3302	-1.4050	-1.0643
H	2.6036	-2.2189	-1.2246
C	4.6527	-1.9991	-0.5969
H	5.0666	-2.6557	-1.3753
H	4.5538	-2.6014	0.3140
H	5.4055	-1.2213	-0.4057
C	3.5075	-0.6595	-2.3819
H	4.3080	0.0912	-2.3125
H	2.5864	-0.1521	-2.6977
H	3.7938	-1.3595	-3.1789
C	3.6224	0.9883	0.6582
H	4.6386	0.5759	0.5472

C	3.4374	1.4568	2.0970
H	4.1106	2.2999	2.3065
H	3.6600	0.6749	2.8326
H	2.4108	1.8087	2.2678
C	3.4561	2.1750	-0.2879
H	2.4828	2.6516	-0.1032
H	3.4987	1.8957	-1.3471
H	4.2370	2.9254	-0.1017
C	0.0002	1.7695	0.7081
C	-0.0000	1.0717	-1.7165
Mo	-0.0000	0.0439	-0.1062
N	-0.0001	-1.3731	1.7703
O	0.0004	2.9388	1.0578
O	0.0001	1.8648	-2.6337
P	-2.4177	-0.3704	0.1999
P	2.4175	-0.3710	0.1998
H	-0.0003	-2.2188	1.1834
O	-0.0002	-2.0789	-0.8129
C	-0.0004	-2.4394	-2.1386
H	0.8800	-2.0297	-2.6914
H	-0.8810	-2.0295	-2.6911
Na	0.0011	3.6288	-1.1442
C	-0.0007	-3.9527	-2.3015
H	-0.0009	-4.2526	-3.3593
H	0.8857	-4.3887	-1.8185
H	-0.8869	-4.3884	-1.8182

Mo_ts_9a_10

Gibbs energy: -1494325.5 kcal mol-1

Atom	X	Y	Z
C	-0.4502	2.0992	2.2594
H	-0.5346	3.0432	2.8139
H	-1.4325	1.6053	2.3193
C	0.5805	1.1713	2.8838
H	1.5779	1.6361	2.9119
H	0.2875	0.9444	3.9246
C	1.5377	-1.0645	2.7369
H	1.1921	-1.2669	3.7662
H	2.5544	-0.6474	2.8028
C	1.5117	-2.3551	1.9321
H	0.4768	-2.7321	1.9390
H	2.1455	-3.1275	2.3879
C	-1.8401	2.5662	-0.2186
H	-2.2538	1.5547	-0.0589
C	-1.8594	2.8479	-1.7164
H	-2.8709	2.6900	-2.1166
H	-1.1702	2.1994	-2.2736

H	-1.5905	3.8925	-1.9290
C	-2.7080	3.5694	0.5299
H	-2.3268	4.5948	0.4254
H	-2.7922	3.3497	1.6013
H	-3.7267	3.5672	0.1160
C	0.6645	4.0621	0.3289
H	-0.1496	4.7947	0.4533
C	1.3105	4.2551	-1.0401
H	1.6558	5.2913	-1.1584
H	0.6420	4.0217	-1.8770
H	2.1883	3.5984	-1.1228
C	1.7034	4.2894	1.4211
H	2.4949	3.5290	1.3767
H	1.2737	4.2772	2.4292
H	2.1825	5.2687	1.2842
C	1.0005	-3.4012	-0.7098
H	-0.0411	-3.0874	-0.5318
C	1.1970	-4.7926	-0.1227
H	0.5513	-5.5153	-0.6412
H	0.9455	-4.8420	0.9432
H	2.2304	-5.1467	-0.2444
C	1.2449	-3.4025	-2.2144
H	2.2348	-3.8137	-2.4583
H	1.1785	-2.3957	-2.6480
H	0.5033	-4.0360	-2.7201
C	3.7431	-2.5099	-0.0270
H	3.7798	-3.6106	-0.0706
C	4.5809	-2.0405	1.1569
H	5.6370	-2.2942	0.9909
H	4.2807	-2.5049	2.1030
H	4.5228	-0.9501	1.2761
C	4.3178	-1.9351	-1.3193
H	4.3844	-0.8415	-1.2279
H	3.7134	-2.1633	-2.2048
H	5.3330	-2.3183	-1.4914
C	2.8575	1.0926	0.0415
C	1.5432	0.5984	-2.0012
Mo	1.1553	0.3037	-0.1517
N	0.6587	-0.0708	2.1039
O	3.9557	1.6103	-0.0312
O	1.9511	0.8501	-3.1132
P	-0.1029	2.3601	0.4449
P	1.9424	-2.0309	0.1489
H	-0.3057	-0.4972	2.1579
H	-3.0070	0.1665	1.9586
O	-1.7131	-1.3067	2.5583
N	-3.2861	-1.5812	0.8088
C	-2.9126	-0.9287	2.1538
H	-3.7531	-1.1958	2.8485
C	-4.3628	-1.1020	0.0642

C	-4.7905	0.2428	0.1474
H	-4.3333	0.9222	0.8609
C	-5.7905	0.7325	-0.6844
H	-6.0848	1.7772	-0.5904
C	-6.4215	-0.0859	-1.6200
H	-7.2032	0.3049	-2.2671
C	-6.0340	-1.4210	-1.6912
H	-6.5176	-2.0919	-2.3997
C	-5.0350	-1.9254	-0.8644
H	-4.7655	-2.9735	-0.9570
C	-3.0811	-3.0174	0.8421
H	-3.9699	-3.5635	1.2041
H	-2.8140	-3.4149	-0.1472
H	-2.2476	-3.2015	1.5285
O	-0.9622	-0.7108	-0.5389
C	-1.5641	-0.8465	-1.8146
H	-1.8352	-1.8934	-2.0190
H	-2.4709	-0.2291	-1.9040
H	-0.8404	-0.5223	-2.5689
H	-1.6195	-0.9986	0.1388
Na	3.9499	1.7114	-2.3379

Mo-ts-9a-12

Gibbs energy: -1217520.8 kcal mol-1

Atom	X	Y	Z
C	-2.4604	-1.7331	1.4193
H	-3.3626	-1.7015	2.0473
H	-2.5131	-2.6612	0.8287
C	-1.1941	-1.7497	2.2696
H	-1.2431	-0.9360	3.0238
H	-1.1793	-2.6935	2.8545
C	1.1819	-1.7198	2.3095
H	1.1499	-2.6435	2.9250
H	1.2086	-0.8792	3.0356
C	2.4699	-1.7346	1.4917
H	2.5467	-2.6889	0.9509
H	3.3542	-1.6586	2.1404
C	-3.5112	-1.0157	-1.1757
H	-3.0518	-2.0030	-1.3408
C	-3.3857	-0.2377	-2.4804
H	-3.8326	-0.8069	-3.3075
H	-2.3377	-0.0321	-2.7332
H	-3.9134	0.7248	-2.4351
C	-4.9706	-1.2316	-0.7974
H	-5.5066	-0.2783	-0.6922
H	-5.0870	-1.7901	0.1404
H	-5.4862	-1.8008	-1.5836
C	-3.4684	0.9863	1.0025

H	-4.4915	0.5817	1.0545
C	-3.4790	2.2598	0.1648
H	-4.1565	3.0044	0.6053
H	-3.7981	2.0891	-0.8703
H	-2.4725	2.7012	0.1432
C	-3.0022	1.2892	2.4214
H	-1.9507	1.6035	2.4394
H	-3.1097	0.4276	3.0909
H	-3.6012	2.1080	2.8441
C	3.4303	-1.1898	-1.1850
H	2.9421	-2.1741	-1.2536
C	4.9081	-1.3966	-0.8814
H	5.3716	-2.0204	-1.6585
H	5.0747	-1.8981	0.0811
H	5.4590	-0.4459	-0.8655
C	3.2172	-0.4913	-2.5226
H	3.5975	0.5395	-2.5231
H	2.1532	-0.4488	-2.7839
H	3.7445	-1.0328	-3.3207
C	3.5777	0.9111	0.9169
H	4.5710	0.4470	1.0264
C	3.0972	1.3465	2.2965
H	3.7611	2.1264	2.6950
H	3.0940	0.5209	3.0184
H	2.0820	1.7615	2.2559
C	3.6872	2.1098	-0.0177
H	2.6889	2.5180	-0.2361
H	4.1596	1.8596	-0.9749
H	4.2818	2.9084	0.4473
C	0.0199	1.7122	0.6833
C	0.0577	1.0862	-1.7274
Mo	0.0212	0.0135	-0.1343
N	0.0054	-1.6260	1.4628
O	0.0337	2.8750	1.0456
O	0.0895	1.8964	-2.6287
P	-2.4141	-0.3302	0.1907
P	2.4463	-0.3912	0.1971
H	0.1068	-2.3458	0.1209
O	0.2581	-2.2721	-0.9318
C	-0.5868	-3.0810	-1.7026
H	-0.9643	-2.5223	-2.5714
H	-0.0505	-3.9663	-2.0791
H	-1.4541	-3.4444	-1.1247
Na	-0.0093	3.6440	-1.1237

Mo-12

Gibbs energy: -1218251.7 kcal mol-1

Atom	X	Y	Z
C	-2.4741	-1.7168	1.0540
H	-3.3814	-1.7703	1.6718
H	-2.4957	-2.5713	0.3601
C	-1.2042	-1.7955	1.8904
H	-1.2400	-1.0315	2.7004
H	-1.1917	-2.7768	2.4141
C	1.1538	-1.8244	1.8910
H	1.1115	-2.7987	2.4256
H	1.2144	-1.0529	2.6926
C	2.4242	-1.7942	1.0523
H	2.4172	-2.6547	0.3656
H	3.3306	-1.8711	1.6689
C	-3.4232	-0.7739	-1.4964
H	-2.8116	-1.6246	-1.8427
C	-3.4706	0.2602	-2.6144
H	-3.8468	-0.1959	-3.5401
H	-2.4824	0.6895	-2.8255
H	-4.1531	1.0852	-2.3666
C	-4.8205	-1.2890	-1.1751
H	-5.4955	-0.4722	-0.8850
H	-4.8252	-2.0343	-0.3706
H	-5.2609	-1.7639	-2.0628
C	-3.5435	1.0439	0.8337
H	-4.5788	0.7110	0.6571
C	-3.3555	2.4336	0.2310
H	-4.1118	3.1274	0.6233
H	-3.4174	2.4469	-0.8634
H	-2.3686	2.8230	0.5176
C	-3.2998	1.0975	2.3373
H	-2.2538	1.3446	2.5629
H	-3.5405	0.1541	2.8403
H	-3.9271	1.8786	2.7893
C	3.3847	-0.8978	-1.5122
H	2.7473	-1.7361	-1.8423
C	4.7719	-1.4457	-1.2021
H	5.1878	-1.9440	-2.0889
H	4.7674	-2.1802	-0.3875
H	5.4719	-0.6434	-0.9318
C	3.4437	0.1230	-2.6417
H	4.1497	0.9332	-2.4114
H	2.4636	0.5746	-2.8439
H	3.7961	-0.3520	-3.5672
C	3.5851	0.9257	0.8091
H	4.6070	0.5550	0.6302
C	3.3510	0.9984	2.3134
H	4.0088	1.7592	2.7567
H	3.5597	0.0505	2.8221
H	2.3160	1.2852	2.5424
C	3.4432	2.3170	0.1979

H	2.4724	2.7431	0.4873
H	3.4989	2.3211	-0.8970
H	4.2260	2.9862	0.5812
C	0.0303	1.6337	0.9990
C	0.0307	1.7104	-1.4675
Mo	0.0044	0.2090	-0.2578
N	-0.0230	-1.6404	1.0653
O	0.0500	2.6753	1.6172
O	0.0489	2.7670	-2.0604
P	-2.4280	-0.1978	-0.0137
P	2.4220	-0.2813	-0.0244
H	-0.0581	-2.8969	-0.1250
O	-0.0655	-3.6879	-0.7497
C	-0.1078	-4.8344	0.0470
H	-1.0870	-4.9808	0.5391
H	0.0737	-5.7168	-0.5811
H	0.6624	-4.8321	0.8393
Na	0.0702	4.0914	-0.2002
H	0.3722	-1.1564	-1.8145
H	-0.4028	-1.0388	-1.8917

Mo-ts-12-13

Gibbs energy: -1218246.5 kcal mol-1

Atom	X	Y	Z
C	-2.4450	1.7671	-1.1010
H	-3.3634	1.8456	-1.6982
H	-2.4064	2.6391	-0.4301
C	-1.2015	1.7833	-1.9780
H	-1.2308	0.9652	-2.7196
H	-1.1741	2.7298	-2.5505
C	1.2117	1.8088	-1.9619
H	1.1877	2.7761	-2.4999
H	1.2467	1.0170	-2.7308
C	2.4508	1.7663	-1.0789
H	2.4150	2.6251	-0.3917
H	3.3715	1.8494	-1.6722
C	-3.3887	0.9194	1.4858
H	-2.7362	1.7431	1.8189
C	-3.4764	-0.1020	2.6132
H	-3.8328	0.3787	3.5340
H	-2.5054	-0.5667	2.8295
H	-4.1912	-0.9022	2.3746
C	-4.7628	1.4934	1.1644
H	-5.4737	0.7071	0.8751
H	-4.7353	2.2391	0.3609
H	-5.1804	1.9879	2.0523
C	-3.5932	-0.9584	-0.7966

H	-4.6176	-0.6058	-0.5972
C	-3.4108	-2.3431	-0.1808
H	-4.1903	-3.0286	-0.5407
H	-3.4395	-2.3415	0.9151
H	-2.4393	-2.7522	-0.4924
C	-3.3911	-1.0381	-2.3053
H	-2.3596	-1.3215	-2.5544
H	-3.6190	-0.0957	-2.8162
H	-4.0524	-1.8063	-2.7297
C	3.3921	0.8798	1.4931
H	2.7488	1.7080	1.8327
C	4.7729	1.4407	1.1775
H	5.1945	1.9248	2.0692
H	4.7551	2.1918	0.3787
H	5.4754	0.6484	0.8844
C	3.4677	-0.1516	2.6122
H	4.1725	-0.9586	2.3671
H	2.4911	-0.6059	2.8253
H	3.8303	0.3174	3.5366
C	3.5792	-0.9718	-0.8126
H	4.6065	-0.6267	-0.6143
C	3.3690	-1.0326	-2.3210
H	4.0217	-1.8012	-2.7579
H	3.6023	-0.0859	-2.8213
H	2.3338	-1.3042	-2.5681
C	3.3924	-2.3618	-0.2103
H	2.4165	-2.7618	-0.5196
H	3.4288	-2.3712	0.8853
H	4.1653	-3.0483	-0.5824
C	-0.0079	-1.6049	-1.0177
C	-0.0073	-1.6890	1.4471
Mo	-0.0026	-0.1991	0.2364
N	0.0012	1.6526	-1.1557
O	-0.0125	-2.6384	-1.6536
O	-0.0098	-2.7387	2.0479
P	-2.4363	0.2730	0.0081
P	2.4326	0.2574	0.0094
H	0.0053	2.4823	-0.3643
O	0.0373	3.4411	0.6453
C	0.0482	4.6678	0.0426
H	-0.5498	4.7122	-0.9044
H	-0.3714	5.4748	0.6829
H	1.0675	5.0270	-0.2459
Na	-0.0084	-4.0625	0.1664
H	0.0027	1.6119	1.7114
H	-0.0038	0.9500	2.1080

Mo-ts-12-13-Li

Gibbs energy: -1121138.8 kcal mol-1

Atom	X	Y	Z
C	-2.4475	1.7806	-1.1145
H	-3.3630	1.8483	-1.7180
H	-2.4225	2.6528	-0.4441
C	-1.2023	1.8066	-1.9879
H	-1.2315	1.0006	-2.7423
H	-1.1700	2.7621	-2.5443
C	1.2092	1.8326	-1.9721
H	1.1783	2.8067	-2.4965
H	1.2453	1.0513	-2.7516
C	2.4503	1.7841	-1.0932
H	2.4266	2.6434	-0.4068
H	3.3681	1.8588	-1.6924
C	-3.3527	0.9253	1.4832
H	-2.7342	1.8028	1.7349
C	-3.3120	-0.0294	2.6710
H	-3.5870	0.5024	3.5917
H	-2.3168	-0.4685	2.8184
H	-4.0293	-0.8528	2.5529
C	-4.7756	1.3956	1.2108
H	-5.4485	0.5480	1.0197
H	-4.8430	2.0818	0.3573
H	-5.1723	1.9256	2.0875
C	-3.6221	-0.9065	-0.8501
H	-4.6242	-0.4566	-0.7645
C	-3.6126	-2.2549	-0.1401
H	-4.3524	-2.9314	-0.5895
H	-3.8357	-2.1788	0.9305
H	-2.6251	-2.7270	-0.2357
C	-3.2978	-1.0836	-2.3286
H	-2.2790	-1.4668	-2.4733
H	-3.3939	-0.1510	-2.8970
H	-3.9899	-1.8097	-2.7768
C	3.3556	0.8918	1.4898
H	2.7456	1.7732	1.7486
C	4.7832	1.3503	1.2218
H	5.1853	1.8675	2.1036
H	4.8570	2.0446	0.3754
H	5.4474	0.4981	1.0218
C	3.3046	-0.0719	2.6698
H	4.0087	-0.9053	2.5431
H	2.3033	-0.4970	2.8166
H	3.5903	0.4484	3.5938
C	3.6095	-0.9136	-0.8672
H	4.6135	-0.4676	-0.7851
C	3.2736	-1.0759	-2.3448
H	3.9582	-1.8022	-2.8043
H	3.3714	-0.1387	-2.9053

H	2.2515	-1.4513	-2.4858
C	3.6012	-2.2679	-0.1686
H	2.6108	-2.7352	-0.2579
H	3.8357	-2.2017	0.9002
H	4.3335	-2.9438	-0.6310
C	-0.0052	-1.6582	-1.0257
C	-0.0039	-1.8426	1.2041
Mo	-0.0022	-0.2271	0.1824
N	-0.0001	1.6572	-1.1673
O	-0.0077	-2.7666	-1.5398
O	-0.0039	-3.0118	1.5446
P	-2.4408	0.2768	-0.0192
P	2.4374	0.2659	-0.0185
H	0.0026	2.4604	-0.3551
O	0.0303	3.3912	0.6940
C	0.0462	4.6348	0.1266
H	-0.5133	4.6961	-0.8429
H	-0.4119	5.4184	0.7695
H	1.0703	5.0165	-0.1106
Li	-0.0075	-4.0175	-0.0859
H	0.0036	1.5347	1.7290
H	-0.0040	0.8659	2.1101

Mo-ts-12-13-K

Gibbs energy: -1492864.2 kcal mol-1

Atom	X	Y	Z
C	-2.4447	1.7609	-1.0963
H	-3.3626	1.8408	-1.6942
H	-2.4060	2.6319	-0.4242
C	-1.2002	1.7782	-1.9716
H	-1.2282	0.9599	-2.7135
H	-1.1729	2.7246	-2.5447
C	1.2112	1.8086	-1.9504
H	1.1876	2.7773	-2.4863
H	1.2498	1.0188	-2.7218
C	2.4476	1.7647	-1.0642
H	2.4056	2.6182	-0.3706
H	3.3704	1.8561	-1.6530
C	-3.3836	0.9141	1.4899
H	-2.7292	1.7376	1.8198
C	-3.4690	-0.1046	2.6197
H	-3.8296	0.3767	3.5387
H	-2.4962	-0.5638	2.8389
H	-4.1795	-0.9090	2.3817
C	-4.7579	1.4895	1.1721
H	-5.4719	0.7031	0.8904
H	-4.7327	2.2306	0.3642

H	-5.1704	1.9897	2.0592
C	-3.6051	-0.9573	-0.7941
H	-4.6273	-0.6033	-0.5855
C	-3.4195	-2.3452	-0.1873
H	-4.1948	-3.0327	-0.5527
H	-3.4478	-2.3499	0.9087
H	-2.4441	-2.7411	-0.5013
C	-3.4125	-1.0315	-2.3044
H	-2.3854	-1.3251	-2.5599
H	-3.6327	-0.0843	-2.8096
H	-4.0848	-1.7901	-2.7291
C	3.3828	0.8649	1.5023
H	2.7331	1.6861	1.8467
C	4.7606	1.4380	1.1949
H	5.1741	1.9230	2.0899
H	4.7406	2.1911	0.3982
H	5.4712	0.6524	0.9030
C	3.4622	-0.1740	2.6138
H	4.1635	-0.9814	2.3594
H	2.4852	-0.6263	2.8286
H	3.8310	0.2874	3.5398
C	3.5916	-0.9654	-0.8161
H	4.6159	-0.6231	-0.5994
C	3.3993	-1.0048	-2.3279
H	4.0637	-1.7609	-2.7692
H	3.6301	-0.0488	-2.8111
H	2.3691	-1.2810	-2.5905
C	3.3972	-2.3651	-0.2395
H	2.4198	-2.7466	-0.5652
H	3.4253	-2.3924	0.8565
H	4.1686	-3.0495	-0.6187
C	-0.0085	-1.5663	-1.0907
C	-0.0137	-1.6616	1.5086
Mo	-0.0042	-0.2166	0.2335
N	0.0002	1.6492	-1.1466
O	-0.0082	-2.5122	-1.8425
O	-0.0196	-2.6296	2.2258
P	-2.4350	0.2638	0.0101
P	2.4286	0.2467	0.0122
H	0.0008	2.4830	-0.3524
O	0.0260	3.4406	0.6460
C	0.0371	4.6649	0.0383
H	-0.5632	4.7066	-0.9071
H	-0.3800	5.4744	0.6770
H	1.0562	5.0219	-0.2537
K	0.0704	-4.2844	0.1491
H	0.0085	1.6006	1.6986
H	0.0027	0.9400	2.0978

Mo-13

Gibbs energy: -1218246.9 kcal mol-1

Atom	X	Y	Z
C	-2.4430	1.7699	-1.0995
H	-3.3707	1.8650	-1.6795
H	-2.3802	2.6377	-0.4248
C	-1.2177	1.7784	-2.0011
H	-1.2582	0.9581	-2.7374
H	-1.1905	2.7245	-2.5722
C	1.2109	1.7661	-2.0123
H	1.1871	2.7122	-2.5838
H	1.2373	0.9449	-2.7481
C	2.4440	1.7486	-1.1213
H	2.3959	2.6200	-0.4505
H	3.3674	1.8289	-1.7101
C	-3.3691	0.9195	1.4910
H	-2.6992	1.7267	1.8297
C	-3.4703	-0.1142	2.6059
H	-3.8161	0.3613	3.5334
H	-2.5060	-0.5964	2.8141
H	-4.1986	-0.8997	2.3598
C	-4.7333	1.5229	1.1815
H	-5.4573	0.7560	0.8732
H	-4.6924	2.2872	0.3963
H	-5.1431	2.0035	2.0805
C	-3.6086	-0.9515	-0.7955
H	-4.6294	-0.6008	-0.5754
C	-3.4168	-2.3423	-0.1963
H	-4.2026	-3.0235	-0.5502
H	-3.4282	-2.3514	0.8999
H	-2.4507	-2.7488	-0.5275
C	-3.4308	-1.0168	-2.3078
H	-2.4027	-1.2954	-2.5759
H	-3.6696	-0.0708	-2.8069
H	-4.0964	-1.7831	-2.7288
C	3.3680	0.9136	1.4734
H	2.7019	1.7273	1.8045
C	4.7351	1.5063	1.1566
H	5.1500	1.9907	2.0511
H	4.6968	2.2653	0.3662
H	5.4536	0.7327	0.8518
C	3.4656	-0.1115	2.5965
H	4.1896	-0.9029	2.3555
H	2.4993	-0.5873	2.8100
H	3.8153	0.3696	3.5196
C	3.5927	-0.9765	-0.8011
H	4.6164	-0.6291	-0.5887
C	3.4064	-1.0526	-2.3120

H	4.0652	-1.8258	-2.7308
H	3.6475	-0.1118	-2.8197
H	2.3751	-1.3274	-2.5718
C	3.3970	-2.3617	-0.1905
H	2.4274	-2.7655	-0.5148
H	3.4126	-2.3627	0.9057
H	4.1777	-3.0496	-0.5427
C	-0.0086	-1.6129	-1.0504
C	-0.0060	-1.7094	1.4212
Mo	-0.0036	-0.2174	0.2172
N	-0.0006	1.6351	-1.1989
O	-0.0119	-2.6414	-1.6934
O	-0.0083	-2.7599	2.0195
P	-2.4370	0.2698	0.0032
P	2.4308	0.2573	-0.0075
H	0.0151	2.4013	-0.4332
O	0.0683	3.3911	0.7588
C	0.0711	4.6196	0.1746
H	-0.1456	4.6000	-0.9308
H	-0.6866	5.3265	0.5922
H	1.0442	5.1664	0.2567
Na	-0.0109	-4.0694	0.1253
H	0.0031	1.5811	1.6996
H	-0.0118	0.9059	2.0806

Mo-ts-13-5

Gibbs energy: -1218248.2 kcal mol-1

Atom	X	Y	Z
C	-2.4014	1.7755	-1.2088
H	-3.3280	1.8591	-1.7921
H	-2.3110	2.6749	-0.5812
C	-1.1776	1.7109	-2.1098
H	-1.2384	0.8641	-2.8134
H	-1.1232	2.6347	-2.7156
C	1.2489	1.6116	-2.1205
H	1.2537	2.5183	-2.7530
H	1.2477	0.7441	-2.8015
C	2.4851	1.6093	-1.2344
H	2.4850	2.5302	-0.6321
H	3.4061	1.6115	-1.8324
C	-3.3195	1.1233	1.4325
H	-2.6184	1.9317	1.6983
C	-3.4315	0.1763	2.6208
H	-3.7346	0.7299	3.5195
H	-2.4815	-0.3286	2.8402
H	-4.1960	-0.5946	2.4486
C	-4.6701	1.7451	1.1013

H	-5.4205	0.9815	0.8535
H	-4.6185	2.4555	0.2676
H	-5.0522	2.2955	1.9721
C	-3.6454	-0.8818	-0.7374
H	-4.6533	-0.4742	-0.5593
C	-3.5166	-2.2302	-0.0342
H	-4.3186	-2.9091	-0.3549
H	-3.5502	-2.1578	1.0591
H	-2.5584	-2.6927	-0.3106
C	-3.4492	-1.0629	-2.2380
H	-2.4278	-1.3963	-2.4669
H	-3.6448	-0.1465	-2.8066
H	-4.1367	-1.8324	-2.6155
C	3.3748	0.9351	1.4168
H	2.7296	1.7885	1.6853
C	4.7591	1.4669	1.0684
H	5.1866	1.9943	1.9321
H	4.7447	2.1754	0.2316
H	5.4549	0.6550	0.8153
C	3.4374	-0.0081	2.6118
H	4.1477	-0.8286	2.4373
H	2.4586	-0.4477	2.8452
H	3.7860	0.5309	3.5028
C	3.5612	-1.1120	-0.7262
H	4.5932	-0.7701	-0.5484
C	3.3574	-1.2949	-2.2258
H	3.9954	-2.1109	-2.5927
H	3.6133	-0.3993	-2.8034
H	2.3176	-1.5649	-2.4549
C	3.3424	-2.4428	-0.0120
H	2.3548	-2.8410	-0.2849
H	3.3813	-2.3644	1.0807
H	4.0967	-3.1762	-0.3281
C	-0.0583	-1.6728	-0.9668
C	-0.0626	-1.6216	1.5097
Mo	-0.0165	-0.2047	0.2199
N	0.0359	1.5695	-1.3004
O	-0.0898	-2.7381	-1.5455
O	-0.0967	-2.6318	2.1724
P	-2.4311	0.3458	-0.0192
P	2.4272	0.1982	-0.0202
H	0.0495	2.3786	-0.5859
O	-0.0335	3.4153	0.5896
C	0.5155	4.5772	0.1440
H	0.6429	4.6223	-0.9736
H	-0.0851	5.4857	0.3905
H	1.5361	4.7932	0.5535
Na	-0.1285	-4.0522	0.3576
H	0.0554	1.6693	1.5715
H	-0.0337	1.0079	1.9790

H2

Gibbs energy: -734.6 kcal mol-1

Atom	X	Y	Z
H	0.0000	0.0000	1.1287
H	0.0000	0.0000	1.8713

H2-1atm

Gibbs energy: -737.5 kcal mol-1

Atom	X	Y	Z
H	0.0000	0.0000	1.1287
H	0.0000	0.0000	1.8713

Mo-6

Gibbs energy: -1421745.5 kcal mol-1

Atom	X	Y	Z
C	2.6579	-1.3592	-1.9962
H	3.6409	-1.6891	-2.3600
H	1.9343	-2.1403	-2.2762
C	2.2481	-0.0520	-2.6608
H	2.9897	0.7393	-2.4778
H	2.1727	-0.1935	-3.7542
C	0.4330	1.6034	-2.7716
H	0.3737	1.4494	-3.8646
H	1.1377	2.4278	-2.5882
C	-0.9488	1.9238	-2.2193
H	-1.6321	1.1044	-2.4926
H	-1.3510	2.8384	-2.6755
C	2.1436	-2.9715	0.3395
H	1.2412	-3.1101	-0.2790
C	1.7106	-3.0924	1.7955
H	1.2412	-4.0707	1.9712
H	0.9879	-2.3116	2.0630
H	2.5617	-3.0169	2.4857
C	3.1637	-4.0406	-0.0280
H	4.0552	-3.9847	0.6122
H	3.4939	-3.9726	-1.0728
H	2.7332	-5.0421	0.1137
C	4.4335	-1.0889	0.3016
H	4.9137	-2.0199	-0.0401
C	4.6131	-0.9542	1.8082

H	5.6695	-0.7890	2.0618
H	4.2737	-1.8394	2.3585
H	4.0355	-0.0955	2.1851
C	5.0895	0.0843	-0.4154
H	4.6066	1.0342	-0.1502
H	5.0575	-0.0170	-1.5072
H	6.1481	0.1554	-0.1282
C	-2.6805	1.4133	0.0124
H	-2.6374	0.3954	-0.4106
C	-3.7993	2.1897	-0.6703
H	-4.7583	1.6678	-0.5300
H	-3.6450	2.3139	-1.7501
H	-3.9228	3.1904	-0.2329
C	-2.9552	1.2774	1.5044
H	-3.0971	2.2599	1.9770
H	-2.1397	0.7575	2.0238
H	-3.8818	0.7076	1.6693
C	-0.9812	3.8345	0.0315
H	-1.9965	4.1939	-0.2017
C	0.0211	4.6130	-0.8121
H	0.0319	5.6688	-0.5068
H	-0.2135	4.5887	-1.8830
H	1.0383	4.2208	-0.6754
C	-0.6931	4.0644	1.5122
H	0.3511	3.7945	1.7260
H	-1.3302	3.4688	2.1769
H	-0.8257	5.1239	1.7723
C	2.2463	2.0347	0.5021
C	0.8299	0.5991	2.1525
Mo	0.9811	0.5887	0.2485
N	0.9646	0.4037	-2.1125
O	3.0010	2.8992	0.9066
O	0.8231	0.7180	3.3597
P	2.6112	-1.2134	-0.1271
P	-0.9344	1.9975	-0.3464
H	0.2881	-0.3516	-2.2663
H	-0.2281	-0.8441	0.0667
O	-1.1103	-1.7092	-2.9747
N	-3.0171	-1.7719	-1.7061
C	-1.7196	-2.0898	-1.9795
H	-1.2648	-2.7528	-1.2258
C	-3.6471	-2.1966	-0.5065
C	-2.9130	-2.3092	0.6807
H	-1.8627	-2.0138	0.7053
C	-3.5470	-2.7299	1.8452
H	-2.9661	-2.8061	2.7621
C	-4.9119	-3.0105	1.8537
H	-5.4035	-3.3250	2.7714
C	-5.6442	-2.8674	0.6780
H	-6.7121	-3.0749	0.6698

C	-5.0192	-2.4662	-0.4997
H	-5.6016	-2.3745	-1.4135
C	-3.7815	-1.0296	-2.6969
H	-4.4062	-1.6956	-3.3059
H	-4.4216	-0.2933	-2.1993
H	-3.0820	-0.5179	-3.3620
Na	2.4459	2.3630	3.0901

Mo-ts-6-7

Gibbs energy: -1421736.7 kcal mol-1

Atom	X	Y	Z
C	2.8043	-1.0675	-2.1420
H	3.8522	-1.2536	-2.4138
H	2.1976	-1.8537	-2.6146
C	2.3436	0.2847	-2.6658
H	2.9893	1.0972	-2.3012
H	2.3899	0.2932	-3.7698
C	0.4048	1.7767	-2.8064
H	0.4175	1.7058	-3.9089
H	1.0502	2.6222	-2.5260
C	-1.0202	1.9895	-2.3167
H	-1.6392	1.1420	-2.6520
H	-1.4540	2.8980	-2.7568
C	2.1356	-3.0386	-0.1588
H	1.3219	-3.1298	-0.8971
C	1.5738	-3.4221	1.2060
H	1.0379	-4.3798	1.1455
H	0.8787	-2.6649	1.5922
H	2.3709	-3.5488	1.9511
C	3.2614	-3.9769	-0.5733
H	4.0826	-3.9669	0.1568
H	3.6803	-3.7302	-1.5574
H	2.8939	-5.0113	-0.6251
C	4.2458	-1.0357	0.4081
H	4.8268	-1.8956	0.0382
C	4.1951	-1.0773	1.9302
H	5.1987	-0.9388	2.3560
H	3.7979	-2.0236	2.3164
H	3.5505	-0.2722	2.3125
C	4.9224	0.2432	-0.0688
H	4.3606	1.1328	0.2434
H	5.0354	0.2784	-1.1595
H	5.9299	0.3168	0.3637
C	-2.8642	1.4322	-0.1739
H	-2.8155	0.4073	-0.5730
C	-3.9343	2.2085	-0.9303
H	-4.9085	1.7109	-0.8123

H	-3.7342	2.2833	-2.0067
H	-4.0530	3.2287	-0.5391
C	-3.2146	1.3275	1.3050
H	-3.3684	2.3200	1.7534
H	-2.4375	0.8004	1.8735
H	-4.1532	0.7695	1.4302
C	-1.1161	3.8007	0.0140
H	-2.1253	4.1801	-0.2137
C	-0.0980	4.6099	-0.7806
H	-0.0766	5.6476	-0.4193
H	-0.3254	4.6457	-1.8525
H	0.9142	4.1999	-0.6559
C	-0.8417	3.9705	1.5074
H	0.2249	3.7835	1.6976
H	-1.4381	3.2979	2.1371
H	-1.0527	5.0015	1.8233
C	1.9255	1.8287	0.8012
C	0.3806	0.1257	1.9782
Mo	0.7458	0.4626	0.1347
N	0.9737	0.5620	-2.2075
O	2.5559	2.6457	1.4435
O	0.2246	0.0095	3.1737
P	2.5205	-1.2072	-0.2974
P	-1.0984	1.9830	-0.4491
H	0.3893	-0.2424	-2.4886
H	-0.5475	-0.8555	-0.4461
O	-0.5704	-1.8841	-2.6109
N	-2.5231	-1.8853	-1.3714
C	-1.1289	-2.0564	-1.5074
H	-0.7459	-2.7868	-0.7751
C	-3.1769	-2.1334	-0.1613
C	-2.4611	-2.2905	1.0413
H	-1.3826	-2.1656	1.0589
C	-3.1330	-2.5407	2.2321
H	-2.5506	-2.6477	3.1458
C	-4.5234	-2.6274	2.2722
H	-5.0411	-2.8215	3.2087
C	-5.2375	-2.4456	1.0908
H	-6.3249	-2.4983	1.0955
C	-4.5805	-2.1966	-0.1109
H	-5.1686	-2.0648	-1.0151
C	-3.2761	-1.4406	-2.5252
H	-3.9397	-2.2315	-2.9021
H	-3.8871	-0.5566	-2.2925
H	-2.5635	-1.1920	-3.3146
Na	1.4061	2.0284	3.3711

Mo-ts-6-7-alt1

Gibbs energy: -1421734.5 kcal mol-1

Atom	X	Y	Z
C	-2.3354	-1.7720	2.1216
H	-3.2623	-2.1049	2.6097
H	-1.6387	-2.6242	2.1454
C	-1.7188	-0.6062	2.8835
H	-2.4284	0.2298	2.9691
H	-1.4621	-0.9257	3.9096
C	0.1526	0.9717	2.9231
H	0.3905	0.6458	3.9516
H	-0.5454	1.8184	3.0006
C	1.4314	1.3672	2.2001
H	2.1086	0.4984	2.1867
H	1.9506	2.1769	2.7303
C	-2.4699	-3.0274	-0.4816
H	-1.5307	-3.4016	-0.0419
C	-2.2558	-2.9314	-1.9879
H	-1.9817	-3.9144	-2.3970
H	-1.4599	-2.2179	-2.2372
H	-3.1646	-2.6072	-2.5123
C	-3.5752	-4.0151	-0.1329
H	-4.5263	-3.7450	-0.6118
H	-3.7504	-4.0898	0.9483
H	-3.3121	-5.0202	-0.4911
C	-4.4398	-0.9570	0.2672
H	-4.9617	-1.8864	0.5451
C	-4.8569	-0.5495	-1.1403
H	-5.9161	-0.2578	-1.1592
H	-4.7225	-1.3536	-1.8734
H	-4.2637	0.3115	-1.4822
C	-4.8245	0.1228	1.2704
H	-4.2924	1.0622	1.0744
H	-4.6216	-0.1738	2.3067
H	-5.9014	0.3298	1.1992
C	2.7555	1.3893	-0.3525
H	2.7978	0.2981	-0.2122
C	3.9669	2.0070	0.3335
H	4.8882	1.6101	-0.1170
H	4.0108	1.7762	1.4047
H	3.9959	3.0998	0.2172
C	2.7757	1.6652	-1.8501
H	2.8056	2.7438	-2.0623
H	1.9015	1.2356	-2.3584
H	3.6782	1.2283	-2.3007
C	1.0263	3.6549	0.3646
H	2.0683	4.0083	0.4223
C	0.2484	4.2444	1.5350
H	0.1780	5.3360	1.4266
H	0.7206	4.0456	2.5042

H	-0.7783	3.8538	1.5640
C	0.4104	4.1385	-0.9478
H	-0.6777	3.9903	-0.9081
H	0.8041	3.6192	-1.8305
H	0.5880	5.2146	-1.0817
C	-2.1064	1.8597	-0.0455
C	-1.0037	0.5943	-1.9825
Mo	-0.8803	0.3855	-0.0873
N	-0.5236	-0.1077	2.1890
O	-2.8448	2.8028	-0.2525
O	-1.1632	0.8449	-3.1572
P	-2.6050	-1.3353	0.3218
P	1.0954	1.7802	0.4110
H	0.1507	-0.8887	2.1165
H	0.4432	-1.0721	-0.1928
O	1.4595	-2.1357	1.6659
N	2.2516	-2.5175	-0.5182
C	1.2332	-2.3752	0.4613
H	0.3788	-3.0119	0.1653
C	3.5687	-2.0645	-0.3426
C	4.3195	-1.6201	-1.4448
H	3.8701	-1.5751	-2.4329
C	5.6412	-1.2081	-1.2908
H	6.1930	-0.8679	-2.1658
C	6.2494	-1.2184	-0.0399
H	7.2809	-0.8943	0.0792
C	5.5097	-1.6586	1.0582
H	5.9673	-1.6850	2.0458
C	4.1942	-2.0830	0.9188
H	3.6337	-2.4263	1.7800
C	1.7524	-2.6890	-1.8657
H	1.5556	-1.7239	-2.3635
H	2.4541	-3.2774	-2.4657
H	0.8042	-3.2360	-1.8244
Na	-2.4005	2.7116	-2.5312

Mo-ts-6-7-Li

Gibbs energy: -1324629.2 kcal mol-1

Atom	X	Y	Z
C	2.7638	-1.1836	-2.1613
H	3.8038	-1.3906	-2.4475
H	2.1363	-1.9665	-2.6109
C	2.3171	0.1693	-2.6960
H	2.9835	0.9752	-2.3552
H	2.3424	0.1620	-3.8004
C	0.3983	1.6875	-2.8335
H	0.3804	1.5868	-3.9333

H	1.0675	2.5265	-2.5939
C	-1.0086	1.9429	-2.3136
H	-1.6545	1.1019	-2.6119
H	-1.4351	2.8496	-2.7639
C	2.0880	-3.1097	-0.1314
H	1.2722	-3.1998	-0.8678
C	1.5178	-3.4463	1.2419
H	0.9829	-4.4059	1.2091
H	0.8198	-2.6758	1.5949
H	2.3080	-3.5453	1.9980
C	3.1942	-4.0793	-0.5251
H	4.0189	-4.0669	0.2007
H	3.6126	-3.8648	-1.5170
H	2.8059	-5.1070	-0.5498
C	4.2551	-1.1375	0.3574
H	4.8152	-2.0060	-0.0247
C	4.2412	-1.1730	1.8805
H	5.2575	-1.0462	2.2782
H	3.8466	-2.1154	2.2790
H	3.6191	-0.3603	2.2813
C	4.9359	0.1303	-0.1427
H	4.3884	1.0285	0.1705
H	5.0322	0.1544	-1.2353
H	5.9502	0.1980	0.2741
C	-2.8126	1.4838	-0.1118
H	-2.7991	0.4448	-0.4755
C	-3.8806	2.2605	-0.8711
H	-4.8625	1.7896	-0.7156
H	-3.7029	2.2941	-1.9533
H	-3.9673	3.2958	-0.5127
C	-3.1290	1.4421	1.3781
H	-3.2379	2.4539	1.7955
H	-2.3567	0.9083	1.9470
H	-4.0822	0.9222	1.5461
C	-1.0066	3.8100	-0.0207
H	-2.0173	4.2006	-0.2199
C	-0.0064	4.5891	-0.8661
H	0.0428	5.6324	-0.5247
H	-0.2711	4.6097	-1.9297
H	1.0041	4.1668	-0.7694
C	-0.6789	4.0112	1.4595
H	0.4068	3.9013	1.6018
H	-1.2084	3.3140	2.1220
H	-0.9345	5.0307	1.7779
C	1.9920	1.7673	0.7882
C	0.5507	0.2894	2.0111
Mo	0.7841	0.4346	0.1277
N	0.9603	0.4781	-2.2170
O	2.5813	2.5510	1.5183
O	0.5443	0.4289	3.2213

P	2.5156	-1.2935	-0.3105
P	-1.0428	1.9834	-0.4468
H	0.3594	-0.3251	-2.4658
H	-0.5645	-0.8601	-0.3749
O	-0.5885	-1.9647	-2.4956
N	-2.5625	-1.8590	-1.3015
C	-1.1741	-2.0793	-1.3988
H	-0.8278	-2.7904	-0.6303
C	-3.2615	-2.0854	-0.1116
C	-2.5921	-2.2039	1.1209
H	-1.5164	-2.0618	1.1769
C	-3.3093	-2.4355	2.2892
H	-2.7652	-2.5166	3.2287
C	-4.6991	-2.5382	2.2748
H	-5.2516	-2.7165	3.1944
C	-5.3674	-2.3941	1.0619
H	-6.4532	-2.4615	1.0246
C	-4.6648	-2.1666	-0.1180
H	-5.2155	-2.0673	-1.0495
C	-3.2719	-1.4135	-2.4827
H	-3.9277	-2.2000	-2.8816
H	-3.8841	-0.5256	-2.2704
H	-2.5317	-1.1733	-3.2488
Li	1.2888	2.2260	2.9519

Mo-ts-6-7-K

Gibbs energy: -1696355.0 kcal mol-1

Atom	X	Y	Z
C	2.7770	-1.1588	-2.1382
H	3.8205	-1.3596	-2.4156
H	2.1593	-1.9458	-2.5944
C	2.3228	0.1890	-2.6778
H	2.9818	1.0011	-2.3372
H	2.3526	0.1786	-3.7825
C	0.3940	1.6876	-2.8287
H	0.3787	1.5797	-3.9284
H	1.0577	2.5326	-2.5944
C	-1.0155	1.9362	-2.3139
H	-1.6547	1.0905	-2.6129
H	-1.4450	2.8387	-2.7703
C	2.0805	-3.0909	-0.1346
H	1.2468	-3.1653	-0.8521
C	1.5458	-3.4589	1.2448
H	1.0152	-4.4207	1.2063
H	0.8526	-2.7020	1.6341
H	2.3587	-3.5721	1.9754
C	3.1717	-4.0594	-0.5720

H	4.0036	-4.0791	0.1459
H	3.5839	-3.8222	-1.5611
H	2.7720	-5.0819	-0.6225
C	4.2323	-1.1305	0.4117
H	4.7790	-2.0343	0.0989
C	4.1747	-1.0767	1.9337
H	5.1870	-1.0390	2.3599
H	3.6546	-1.9352	2.3757
H	3.6393	-0.1702	2.2503
C	4.9669	0.0900	-0.1286
H	4.4261	1.0164	0.1040
H	5.1172	0.0468	-1.2142
H	5.9622	0.1625	0.3321
C	-2.8395	1.4910	-0.1327
H	-2.8257	0.4447	-0.4759
C	-3.8947	2.2563	-0.9209
H	-4.8825	1.7991	-0.7606
H	-3.7086	2.2592	-2.0022
H	-3.9738	3.3019	-0.5915
C	-3.1779	1.4806	1.3525
H	-3.2631	2.5022	1.7511
H	-2.4272	0.9355	1.9391
H	-4.1476	0.9905	1.5162
C	-1.0309	3.8167	-0.0419
H	-2.0408	4.2079	-0.2459
C	-0.0272	4.5804	-0.8979
H	0.0280	5.6278	-0.5696
H	-0.2916	4.5887	-1.9617
H	0.9807	4.1545	-0.7952
C	-0.6970	4.0345	1.4325
H	0.3694	3.8208	1.5900
H	-1.2874	3.4022	2.1078
H	-0.8688	5.0826	1.7152
C	1.9951	1.8076	0.7087
C	0.4043	0.0368	1.9847
Mo	0.7648	0.4313	0.1440
N	0.9623	0.4858	-2.2045
O	2.6908	2.6582	1.2159
O	0.2223	-0.1997	3.1542
P	2.5001	-1.2670	-0.2895
P	-1.0599	1.9814	-0.4448
H	0.3691	-0.3207	-2.4597
H	-0.5599	-0.8634	-0.4185
O	-0.6034	-1.9519	-2.5510
N	-2.5603	-1.8767	-1.3220
C	-1.1692	-2.0806	-1.4450
H	-0.8069	-2.8002	-0.6915
C	-3.2280	-2.0863	-0.1127
C	-2.5250	-2.2059	1.1013
H	-1.4458	-2.0864	1.1222

C	-3.2107	-2.4033	2.2941
H	-2.6383	-2.4769	3.2173
C	-4.6021	-2.4789	2.3224
H	-5.1307	-2.6314	3.2607
C	-5.3033	-2.3429	1.1269
H	-6.3909	-2.3916	1.1221
C	-4.6323	-2.1438	-0.0764
H	-5.2088	-2.0450	-0.9923
C	-3.2950	-1.4287	-2.4860
H	-3.9664	-2.2115	-2.8663
H	-3.8954	-0.5354	-2.2617
H	-2.5714	-1.1951	-3.2699
K	1.6629	2.0184	3.6096

Mo-7

Gibbs energy: -1421743.2 kcal mol-1

Atom	X	Y	Z
Mo	0.7050	0.4261	0.0991
P	-1.1923	1.9117	-0.5167
P	2.5882	-1.1602	-0.2940
C	-1.2193	3.7279	-0.0663
C	-1.0962	1.8770	-2.3782
C	-2.9437	1.3299	-0.2328
C	0.3759	0.1352	1.9625
C	1.7877	1.8130	0.7634
C	4.2862	-0.8465	0.4162
C	2.8416	-1.0514	-2.1392
C	2.2890	-2.9966	-0.0862
C	-0.2033	4.5364	-0.8642
C	-0.9616	3.9155	1.4280
H	-2.2304	4.0939	-0.3069
C	0.3348	1.6815	-2.8554
H	-1.5493	2.7640	-2.8416
H	-1.6919	1.0061	-2.6945
C	-4.0193	2.0616	-1.0256
C	-3.3025	1.2750	1.2473
H	-2.8813	0.2928	-0.6012
O	0.2651	0.0727	3.1632
O	2.4071	2.6648	1.3688
C	4.8955	0.4369	-0.1344
C	4.2183	-0.7969	1.9377
H	4.9188	-1.6935	0.1063
C	2.3125	0.2674	-2.6837
H	3.8925	-1.1991	-2.4221
H	2.2585	-1.8716	-2.5818
C	1.7622	-3.3626	1.2972
C	3.4594	-3.8865	-0.4828

H	1.4716	-3.1550	-0.8095
H	-0.4197	4.5489	-1.9386
H	-0.2024	5.5804	-0.5214
H	0.8137	4.1451	-0.7209
H	0.1023	3.7318	1.6344
H	-1.1778	4.9503	1.7265
H	-1.5647	3.2511	2.0587
N	0.9349	0.4996	-2.2207
H	0.9578	2.5551	-2.6117
H	0.3462	1.5699	-3.9542
H	-4.1566	3.0941	-0.6744
H	-4.9837	1.5495	-0.8948
H	-3.8130	2.0951	-2.1024
H	-3.4964	2.2799	1.6503
H	-4.2201	0.6876	1.3883
H	-2.5138	0.8016	1.8468
H	5.0343	0.4055	-1.2218
H	5.8850	0.6018	0.3137
H	4.2760	1.3105	0.1058
H	3.5670	0.0276	2.2627
H	5.2167	-0.6254	2.3630
H	3.8226	-1.7203	2.3775
H	2.9323	1.1144	-2.3520
H	2.3447	0.2495	-3.7877
H	2.5701	-3.4059	2.0402
H	1.2919	-4.3551	1.2739
H	1.0168	-2.6440	1.6627
H	4.2813	-3.8165	0.2432
H	3.1430	-4.9382	-0.5057
H	3.8601	-3.6447	-1.4755
H	0.3680	-0.3466	-2.4995
Na	1.3906	2.1050	3.3873
C	-0.9673	-1.9927	-1.3909
H	-0.6418	-2.9337	-0.8771
O	-0.4917	-1.8073	-2.5907
H	-0.6435	-1.1705	-0.6094
N	-2.4622	-1.9763	-1.2892
C	-3.0857	-2.1890	-0.0854
C	-2.3468	-2.3853	1.1069
C	-4.4963	-2.1871	0.0224
C	-2.9922	-2.5718	2.3230
H	-1.2607	-2.3829	1.0890
C	-5.1223	-2.3802	1.2490
H	-5.1074	-2.0385	-0.8642
C	-4.3837	-2.5734	2.4154
H	-2.3858	-2.7125	3.2171
H	-6.2110	-2.3776	1.2883
H	-4.8798	-2.7222	3.3718
C	-3.2292	-1.7683	-2.4887
H	-2.5143	-1.6459	-3.3074

H	-3.8804	-2.6269	-2.7156
H	-3.8671	-0.8691	-2.4288

Mo-ts-7-8

Gibbs energy: -1421740.2 kcal mol-1

Atom	X	Y	Z
Mo	0.7572	0.3990	0.1016
P	-1.1372	1.9133	-0.4552
P	2.6502	-1.1516	-0.3153
C	-1.0628	3.7533	-0.1296
C	-1.1767	1.7924	-2.3117
C	-2.8917	1.4131	-0.0383
C	0.3322	-0.0862	1.9119
C	1.7465	1.6770	1.0563
C	4.3858	-0.7274	0.2287
C	2.7677	-1.1670	-2.1736
C	2.4462	-2.9816	0.0454
C	0.0582	4.4126	-0.9245
C	-0.8797	4.0474	1.3573
H	-2.0270	4.1688	-0.4646
C	0.2042	1.4770	-2.8681
H	-1.6017	2.6914	-2.7808
H	-1.8527	0.9543	-2.5410
C	-3.9879	2.2530	-0.6808
C	-3.1395	1.2426	1.4550
H	-2.9153	0.4082	-0.4892
O	0.1444	-0.2592	3.0946
O	2.2924	2.4613	1.8069
C	4.8097	0.6167	-0.3521
C	4.4974	-0.7229	1.7487
H	5.0451	-1.5080	-0.1835
C	2.1332	0.0867	-2.7617
H	3.8052	-1.2869	-2.5159
H	2.2059	-2.0471	-2.5176
C	1.9967	-3.2642	1.4736
C	3.6442	-3.8397	-0.3409
H	1.6055	-3.2384	-0.6218
H	-0.1057	4.3583	-2.0068
H	0.1348	5.4759	-0.6579
H	1.0279	3.9445	-0.7028
H	0.1533	3.8111	1.6510
H	-1.0327	5.1162	1.5592
H	-1.5789	3.4897	1.9945
N	0.8504	0.3944	-2.1289
H	0.8398	2.3837	-2.8591
H	0.0948	1.1959	-3.9360
H	-4.0688	3.2442	-0.2124

H	-4.9601	1.7560	-0.5485
H	-3.8411	2.3999	-1.7585
H	-3.2494	2.2124	1.9620
H	-4.0740	0.6880	1.6194
H	-2.3342	0.6772	1.9417
H	4.8240	0.6124	-1.4490
H	5.8235	0.8689	-0.0122
H	4.1389	1.4229	-0.0271
H	3.7282	-0.0785	2.1977
H	5.4785	-0.3372	2.0582
H	4.3885	-1.7242	2.1814
H	2.8219	0.9490	-2.6664
H	1.9927	-0.0715	-3.8503
H	2.7883	-3.0539	2.2041
H	1.7284	-4.3241	1.5828
H	1.1249	-2.6620	1.7560
H	4.4961	-3.6670	0.3312
H	3.3885	-4.9056	-0.2661
H	3.9825	-3.6567	-1.3688
H	0.0274	-0.7774	-2.4485
Na	0.7690	1.9532	3.4989
C	-1.1006	-2.0393	-1.4447
H	-0.8983	-3.1089	-1.2255
O	-0.5645	-1.6669	-2.6345
H	-0.6431	-1.4645	-0.5888
N	-2.5546	-1.8081	-1.3589
C	-3.2069	-2.0872	-0.1759
C	-2.4991	-2.4350	0.9976
C	-4.6138	-1.9975	-0.0817
C	-3.1699	-2.6614	2.1935
H	-1.4162	-2.5207	0.9896
C	-5.2668	-2.2326	1.1232
H	-5.2032	-1.7405	-0.9578
C	-4.5576	-2.5633	2.2765
H	-2.5857	-2.9162	3.0766
H	-6.3527	-2.1550	1.1547
H	-5.0747	-2.7460	3.2156
C	-3.3132	-1.6251	-2.5733
H	-2.6107	-1.4821	-3.3964
H	-3.9519	-2.4939	-2.8009
H	-3.9605	-0.7350	-2.5137

Mo-ts-7-8-alt1

Gibbs energy: -1421735.5 kcal mol-1

Atom	X	Y	Z
C	-2.5194	-1.3539	2.0511
H	-3.4654	-1.4674	2.5991

H	-2.0147	-2.3306	2.0620
C	-1.6185	-0.3269	2.7249
H	-2.1749	0.6074	2.9226
H	-1.3113	-0.7224	3.7128
C	0.4662	0.8402	2.6784
H	0.7647	0.3373	3.6193
H	-0.0703	1.7586	2.9771
C	1.7220	1.1857	1.8917
H	2.3358	0.2791	1.7789
H	2.3326	1.9284	2.4253
C	-2.9978	-2.6164	-0.5097
H	-2.1734	-3.1756	-0.0388
C	-2.7369	-2.6008	-2.0110
H	-2.7813	-3.6222	-2.4144
H	-1.7496	-2.1841	-2.2454
H	-3.4783	-2.0003	-2.5554
C	-4.2983	-3.3320	-0.1679
H	-5.1625	-2.8671	-0.6615
H	-4.4963	-3.3542	0.9117
H	-4.2551	-4.3745	-0.5118
C	-4.5241	-0.1939	0.2547
H	-5.1953	-0.9877	0.6187
C	-4.9357	0.1984	-1.1591
H	-5.9215	0.6837	-1.1514
H	-4.9981	-0.6637	-1.8342
H	-4.2158	0.9081	-1.5914
C	-4.6320	0.9921	1.2053
H	-3.9374	1.7955	0.9301
H	-4.4303	0.7127	2.2465
H	-5.6493	1.4058	1.1708
C	2.8818	1.3248	-0.7529
H	3.0958	0.3098	-0.3813
C	4.0753	2.2081	-0.4162
H	4.9869	1.7872	-0.8629
H	4.2510	2.2813	0.6652
H	3.9602	3.2260	-0.8147
C	2.6727	1.2089	-2.2575
H	2.4034	2.1693	-2.7200
H	1.8785	0.4909	-2.4989
H	3.5989	0.8662	-2.7409
C	1.3055	3.6019	0.2961
H	2.3346	3.8883	0.5634
C	0.3713	4.0942	1.3952
H	0.3285	5.1922	1.3855
H	0.7077	3.7922	2.3938
H	-0.6520	3.7206	1.2539
C	0.9455	4.2486	-1.0370
H	-0.1037	4.0309	-1.2828
H	1.5815	3.9093	-1.8636
H	1.0413	5.3413	-0.9717

C	-1.8305	2.0803	-0.4955
C	-0.9432	0.5643	-2.1771
Mo	-0.8270	0.5120	-0.2629
N	-0.4337	-0.0271	1.9141
O	-2.4451	3.0750	-0.8291
O	-1.0980	0.7312	-3.3656
P	-2.8003	-0.9154	0.2624
P	1.3001	1.7346	0.1656
H	0.1639	-1.1317	1.7825
H	0.4128	-1.4629	-0.3068
O	0.6956	-2.2258	1.5927
N	1.9026	-2.9492	-0.3598
C	0.6666	-2.4310	0.2738
H	-0.1114	-3.1554	-0.0516
C	3.1380	-2.3517	-0.0797
C	3.5394	-2.1242	1.2536
H	2.8583	-2.4013	2.0510
C	4.7734	-1.5539	1.5360
H	5.0508	-1.3888	2.5762
C	5.6642	-1.2134	0.5153
H	6.6335	-0.7766	0.7463
C	5.2892	-1.4562	-0.8016
H	5.9636	-1.2020	-1.6187
C	4.0446	-2.0089	-1.1008
H	3.7796	-2.1554	-2.1445
C	1.6457	-3.3096	-1.7359
H	1.4817	-2.4336	-2.3954
H	2.4713	-3.9018	-2.1440
H	0.7427	-3.9297	-1.7785
Na	-1.8564	2.8944	-3.0544

Mo-8

Gibbs energy: -1421741.2 kcal mol-1

Atom	X	Y	Z
C	2.8122	-1.1794	-2.1786
H	3.8423	-1.2507	-2.5560
H	2.3004	-2.1123	-2.4544
C	2.0746	-0.0032	-2.8040
H	2.7442	0.8776	-2.8752
H	1.8263	-0.2710	-3.8521
C	0.0811	1.2726	-2.8904
H	-0.0979	0.8362	-3.8959
H	0.6569	2.2014	-3.0751
C	-1.2696	1.6019	-2.2710
H	-1.9457	0.7433	-2.4036
H	-1.7352	2.4675	-2.7640
C	2.7813	-2.9120	0.1173

H	1.9787	-3.3000	-0.5330
C	2.3675	-3.1836	1.5579
H	2.3047	-4.2664	1.7341
H	1.3888	-2.7461	1.7869
H	3.0857	-2.7767	2.2817
C	4.0783	-3.6271	-0.2398
H	4.9024	-3.3179	0.4176
H	4.3890	-3.4482	-1.2772
H	3.9605	-4.7126	-0.1170
C	4.4645	-0.4484	0.1159
H	5.1930	-1.1384	-0.3398
C	4.6722	-0.4145	1.6248
H	5.6282	0.0696	1.8677
H	4.6912	-1.4165	2.0699
H	3.8751	0.1580	2.1194
C	4.6622	0.9375	-0.4891
H	3.8944	1.6394	-0.1321
H	4.6218	0.9259	-1.5854
H	5.6436	1.3385	-0.2012
C	-2.8783	1.4240	0.1034
H	-3.0090	0.4416	-0.3777
C	-3.9575	2.3605	-0.4223
H	-4.9506	1.9330	-0.2222
H	-3.8853	2.5220	-1.5057
H	-3.9239	3.3435	0.0684
C	-3.0196	1.1970	1.6041
H	-2.9124	2.1315	2.1756
H	-2.2960	0.4545	1.9667
H	-4.0200	0.8040	1.8328
C	-0.9699	3.6867	-0.2471
H	-1.8746	4.1307	-0.6928
C	0.2513	4.1969	-1.0044
H	0.3955	5.2688	-0.8108
H	0.1550	4.0719	-2.0889
H	1.1650	3.6748	-0.6829
C	-0.8789	4.1041	1.2168
H	0.0504	3.7204	1.6634
H	-1.7398	3.7716	1.8110
H	-0.8379	5.1988	1.2997
C	1.5799	1.4844	1.3851
C	0.2028	-0.4071	1.7726
Mo	0.7870	0.3412	0.1094
N	0.8574	0.3454	-2.0730
O	1.9680	2.1857	2.2960
O	-0.1315	-0.7329	2.8895
P	2.7648	-1.0883	-0.3213
P	-1.1306	1.8297	-0.4327
H	-0.0506	-1.1085	-2.3911
H	-0.6858	-1.8523	-0.5316
O	-0.5433	-1.9653	-2.5725

N	-2.5923	-1.8750	-1.3817
C	-1.1878	-2.3173	-1.4077
H	-1.1368	-3.4157	-1.2838
C	-3.3192	-2.0688	-0.2173
C	-2.7008	-2.4424	0.9961
H	-1.6303	-2.6203	1.0467
C	-3.4433	-2.5769	2.1636
H	-2.9234	-2.8576	3.0784
C	-4.8196	-2.3605	2.1772
H	-5.3935	-2.4759	3.0938
C	-5.4432	-2.0036	0.9833
H	-6.5188	-1.8345	0.9586
C	-4.7162	-1.8622	-0.1939
H	-5.2424	-1.5806	-1.1021
C	-3.3027	-1.8218	-2.6418
H	-2.5705	-1.7736	-3.4501
H	-3.9494	-2.7014	-2.7980
H	-3.9312	-0.9210	-2.7044
Na	-0.0392	1.5610	3.4277

Mo-ts-CNH

Gibbs energy: -1421726.9 kcal mol-1

Atom	X	Y	Z
Mo	0.3412	0.5049	-0.3477
P	2.6768	-0.3267	0.0648
P	-1.9354	1.3862	0.1972
O	0.4915	1.2298	-3.3694
O	0.6686	-3.4121	2.0284
N	0.2690	0.0958	1.9787
H	-0.0660	-0.8706	2.0104
N	-0.4413	-1.8534	-0.5936
C	0.3895	0.8492	-2.2262
C	2.5873	-0.7624	1.8732
H	2.2356	-1.8034	1.9273
H	3.5715	-0.7284	2.3600
C	1.5980	0.1192	2.6185
H	1.5203	-0.2149	3.6678
H	1.9292	1.1673	2.6343
C	-0.6858	0.9667	2.6934
H	-0.2323	1.9678	2.7215
H	-0.7943	0.6322	3.7399
C	-2.0531	1.0215	2.0256
H	-2.6946	1.7421	2.5517
H	-2.5468	0.0433	2.1046
C	4.1485	0.8395	-0.0479
H	5.0369	0.1882	-0.0222
C	4.2481	1.8163	1.1183

H	5.1093	2.4830	0.9699
H	4.3925	1.3164	2.0830
H	3.3552	2.4517	1.1840
C	4.1583	1.6206	-1.3636
H	3.4956	2.4904	-1.2663
H	3.8425	1.0256	-2.2291
H	5.1667	2.0045	-1.5711
C	3.4185	-1.8934	-0.6620
H	2.5544	-2.5708	-0.7012
C	3.8939	-1.6794	-2.0955
H	4.8259	-1.0984	-2.1308
H	3.1462	-1.1646	-2.7142
H	4.1034	-2.6480	-2.5690
C	4.5018	-2.5566	0.1795
H	4.8838	-3.4483	-0.3372
H	4.1323	-2.8846	1.1583
H	5.3628	-1.8942	0.3484
C	-2.2830	3.2278	0.0787
H	-3.3720	3.3273	0.2107
C	-1.6023	4.0536	1.1637
H	-0.5107	3.9530	1.1211
H	-1.9379	3.7878	2.1731
H	-1.8357	5.1179	1.0180
C	-1.9094	3.7709	-1.2990
H	-2.3272	4.7779	-1.4366
H	-2.2628	3.1429	-2.1247
H	-0.8178	3.8560	-1.3753
C	-3.5351	0.6424	-0.4308
H	-3.3742	-0.4239	-0.2354
C	-4.8035	1.0607	0.3006
H	-5.0655	2.1129	0.1208
H	-4.7389	0.9055	1.3851
H	-5.6478	0.4549	-0.0589
C	-3.6873	0.8063	-1.9382
H	-4.4774	0.1370	-2.3064
H	-2.7622	0.5563	-2.4769
H	-3.9806	1.8303	-2.2111
C	0.5218	-3.7615	0.8706
C	-1.7384	-2.2431	-0.3375
C	-2.6567	-2.7001	-1.3221
H	-2.3601	-2.7334	-2.3671
C	-3.9626	-3.0638	-0.9985
H	-4.6307	-3.3811	-1.7994
C	-4.4252	-3.0296	0.3151
H	-5.4435	-3.3223	0.5608
C	-3.5241	-2.6428	1.3132
H	-3.8346	-2.6452	2.3584
C	-2.2277	-2.2689	0.9986
H	-1.5429	-2.0301	1.8099
H	1.3662	-3.7754	0.1506

H	-0.4152	-4.2270	0.5082
C	0.0040	-2.2653	-1.9125
H	-0.5051	-1.7423	-2.7451
H	1.0723	-2.0526	-2.0316
H	-0.1433	-3.3515	-2.0864
C	1.0027	2.2751	-0.2995
O	1.4297	3.4026	-0.4744
Na	1.4798	3.2265	-2.7833

Mo-10

Gibbs energy: -1494325.1 kcal mol-1

Atom	X	Y	Z
C	-0.2469	2.2478	2.1561
H	-0.2615	3.2176	2.6709
H	-1.2589	1.8230	2.2483
C	0.7297	1.2800	2.8055
H	1.7569	1.6752	2.7948
H	0.4429	1.1197	3.8602
C	1.5308	-1.0205	2.7396
H	1.2044	-1.1457	3.7874
H	2.5773	-0.6791	2.7542
C	1.3774	-2.3416	2.0020
H	0.3152	-2.6272	2.0565
H	1.9583	-3.1430	2.4775
C	-1.6369	2.7063	-0.3200
H	-2.1108	1.7318	-0.1083
C	-1.6679	2.9204	-1.8284
H	-2.6966	2.8146	-2.2016
H	-1.0376	2.1999	-2.3662
H	-1.3327	3.9329	-2.0952
C	-2.4237	3.7964	0.3957
H	-1.9787	4.7890	0.2397
H	-2.5036	3.6291	1.4768
H	-3.4474	3.8444	-0.0027
C	0.9745	4.0491	0.1391
H	0.2149	4.8410	0.2430
C	1.6139	4.1412	-1.2432
H	2.0182	5.1485	-1.4134
H	0.9237	3.9081	-2.0620
H	2.4507	3.4308	-1.3028
C	2.0415	4.2462	1.2099
H	2.7740	3.4275	1.1934
H	1.6258	4.3120	2.2217
H	2.5904	5.1797	1.0236
C	0.6778	-3.4505	-0.5677
H	-0.3215	-3.0165	-0.3964
C	0.7364	-4.8172	0.1017

H	0.0110	-5.4972	-0.3671
H	0.4963	-4.7767	1.1706
H	1.7257	-5.2839	-0.0057
C	0.8932	-3.5708	-2.0719
H	1.8312	-4.0971	-2.2992
H	0.9218	-2.5925	-2.5698
H	0.0821	-4.1559	-2.5268
C	3.5159	-2.7935	-0.0179
H	3.4490	-3.8938	-0.0184
C	4.4333	-2.3590	1.1194
H	5.4547	-2.7186	0.9338
H	4.1224	-2.7529	2.0935
H	4.4815	-1.2639	1.1916
C	4.0954	-2.3214	-1.3488
H	4.2488	-1.2335	-1.3085
H	3.4494	-2.5363	-2.2077
H	5.0717	-2.7916	-1.5304
C	2.9515	0.8941	-0.0539
C	1.5827	0.4332	-2.0603
Mo	1.1874	0.2419	-0.1989
N	0.7057	0.0028	2.0811
O	4.0866	1.3177	-0.1608
O	2.0038	0.6108	-3.1820
P	0.0932	2.4108	0.3290
P	1.7754	-2.1395	0.1937
H	-0.2820	-0.3569	2.1830
H	-2.9235	0.4384	2.0095
O	-1.7114	-1.0635	2.7011
N	-3.2600	-1.3426	0.9207
C	-2.8805	-0.6508	2.2534
H	-3.7501	-0.8539	2.9343
C	-4.3439	-0.8658	0.1783
C	-4.6967	0.5024	0.1776
H	-4.1709	1.2056	0.8176
C	-5.7047	0.9846	-0.6488
H	-5.9410	2.0476	-0.6225
C	-6.4137	0.1355	-1.4974
H	-7.2003	0.5203	-2.1421
C	-6.0961	-1.2194	-1.4874
H	-6.6403	-1.9118	-2.1280
C	-5.0907	-1.7163	-0.6630
H	-4.8772	-2.7808	-0.6895
C	-3.1056	-2.7826	1.0195
H	-4.0002	-3.2769	1.4377
H	-2.8866	-3.2362	0.0424
H	-2.2584	-2.9635	1.6899
O	-1.0163	-0.5862	-0.5380
C	-1.6221	-0.7563	-1.8077
H	-1.9299	-1.8011	-1.9682
H	-2.5086	-0.1140	-1.9219

H	-0.8906	-0.4887	-2.5764
H	-1.6888	-0.8327	0.1464
Na	4.0590	1.3777	-2.4684

Mo-ts-10-11

Gibbs energy: -1494320.7 kcal mol-1

Atom	X	Y	Z
C	-0.4865	2.1031	2.1272
H	-0.5965	3.0209	2.7209
H	-1.4977	1.6845	1.9984
C	0.3655	1.0678	2.8488
H	1.3878	1.4360	3.0199
H	-0.0785	0.8425	3.8346
C	1.2028	-1.2264	2.7014
H	0.7926	-1.4139	3.7095
H	2.2455	-0.8955	2.8174
C	1.0988	-2.4951	1.8669
H	0.0340	-2.7711	1.8264
H	1.6317	-3.3336	2.3346
C	-1.3784	2.9489	-0.4973
H	-1.8746	1.9711	-0.6011
C	-1.0767	3.4608	-1.9008
H	-2.0041	3.5133	-2.4880
H	-0.3790	2.8063	-2.4398
H	-0.6537	4.4755	-1.8789
C	-2.2944	3.9110	0.2469
H	-1.8077	4.8797	0.4304
H	-2.6337	3.5195	1.2137
H	-3.1935	4.1159	-0.3529
C	1.1695	4.0004	0.5657
H	0.4479	4.8339	0.5956
C	2.0849	4.1710	-0.6450
H	2.5579	5.1626	-0.6308
H	1.5665	4.0558	-1.6036
H	2.8870	3.4207	-0.5963
C	2.0078	4.0330	1.8384
H	2.6986	3.1795	1.8771
H	1.4018	4.0267	2.7517
H	2.6175	4.9469	1.8611
C	0.6183	-3.4300	-0.8349
H	-0.3765	-2.9560	-0.7948
C	0.5376	-4.8193	-0.2161
H	-0.1233	-5.4622	-0.8156
H	0.1424	-4.8112	0.8064
H	1.5209	-5.3105	-0.1892
C	1.0392	-3.5164	-2.2980
H	1.9777	-4.0775	-2.4131

H	1.1748	-2.5265	-2.7539
H	0.2752	-4.0488	-2.8811
C	3.3868	-2.8670	0.0164
H	3.2931	-3.9620	-0.0741
C	4.2066	-2.5486	1.2617
H	5.2318	-2.9253	1.1401
H	3.8005	-3.0049	2.1716
H	4.2757	-1.4642	1.4221
C	4.1080	-2.3226	-1.2144
H	4.3174	-1.2537	-1.0666
H	3.5329	-2.4282	-2.1412
H	5.0710	-2.8343	-1.3506
C	2.9264	0.7792	0.1924
C	1.6968	0.5280	-1.9675
Mo	1.1459	0.2309	-0.1610
N	0.4406	-0.1561	2.0405
O	4.0826	1.1635	0.2276
O	2.2032	0.7601	-3.0437
P	0.1679	2.4249	0.4093
P	1.6440	-2.1829	0.1100
H	-0.5317	-0.5263	2.0127
H	-3.2277	0.1803	1.9770
O	-2.0190	-1.3540	2.6386
N	-3.1499	-1.3512	0.4664
C	-3.0918	-0.9267	2.0816
H	-4.0679	-1.3647	2.4190
C	-4.3367	-0.8358	-0.1421
C	-4.4184	0.5457	-0.3515
H	-3.5678	1.1710	-0.0897
C	-5.5623	1.1092	-0.9029
H	-5.6040	2.1843	-1.0671
C	-6.6470	0.3039	-1.2483
H	-7.5416	0.7439	-1.6829
C	-6.5717	-1.0687	-1.0337
H	-7.4111	-1.7087	-1.2976
C	-5.4270	-1.6410	-0.4804
H	-5.3915	-2.7158	-0.3254
C	-2.9668	-2.7998	0.3914
H	-3.7424	-3.3267	0.9628
H	-2.9755	-3.1422	-0.6505
H	-2.0013	-3.0297	0.8492
O	-1.0568	-0.4071	-0.5996
C	-1.3833	-0.5037	-1.9493
H	-2.3784	-0.9716	-2.0975
H	-1.4294	0.4838	-2.4521
H	-0.6615	-1.1144	-2.5237
H	-2.1840	-0.8664	0.0373
Na	4.1850	1.4257	-2.0589

Mo-11

Gibbs energy: -1494319.4 kcal mol-1

Atom	X	Y	Z
C	-0.5245	2.0953	2.0708
H	-0.6572	3.0116	2.6623
H	-1.5294	1.6711	1.9130
C	0.3136	1.0660	2.8162
H	1.3257	1.4435	3.0217
H	-0.1594	0.8310	3.7862
C	1.1826	-1.2203	2.6826
H	0.7548	-1.4091	3.6828
H	2.2214	-0.8844	2.8162
C	1.0986	-2.4878	1.8445
H	0.0362	-2.7702	1.7874
H	1.6279	-3.3241	2.3202
C	-1.3495	2.9473	-0.5728
H	-1.8349	1.9667	-0.7037
C	-1.0092	3.4827	-1.9587
H	-1.9186	3.5394	-2.5730
H	-0.2920	2.8409	-2.4872
H	-0.5934	4.4994	-1.9081
C	-2.2937	3.8934	0.1567
H	-1.8174	4.8606	0.3726
H	-2.6629	3.4846	1.1053
H	-3.1734	4.1049	-0.4691
C	1.1621	4.0080	0.5640
H	0.4362	4.8381	0.5812
C	2.1063	4.1903	-0.6228
H	2.5801	5.1812	-0.5867
H	1.6102	4.0863	-1.5945
H	2.9058	3.4381	-0.5633
C	1.9692	4.0352	1.8570
H	2.6612	3.1832	1.9071
H	1.3410	4.0215	2.7551
H	2.5757	4.9506	1.9006
C	0.6517	-3.4153	-0.8619
H	-0.3415	-2.9366	-0.8295
C	0.5617	-4.8066	-0.2484
H	-0.0986	-5.4448	-0.8537
H	0.1617	-4.8017	0.7723
H	1.5425	-5.3024	-0.2187
C	1.0876	-3.4999	-2.3207
H	2.0261	-4.0629	-2.4266
H	1.2298	-2.5095	-2.7729
H	0.3287	-4.0295	-2.9131
C	3.4115	-2.8655	0.0283
H	3.3160	-3.9603	-0.0626
C	4.2154	-2.5480	1.2842

H	5.2434	-2.9205	1.1733
H	3.8006	-3.0089	2.1879
H	4.2785	-1.4639	1.4491
C	4.1497	-2.3238	-1.1932
H	4.3521	-1.2532	-1.0480
H	3.5888	-2.4351	-2.1281
H	5.1163	-2.8331	-1.3128
C	2.9639	0.7946	0.2067
C	1.7670	0.5376	-1.9784
Mo	1.1863	0.2426	-0.1812
N	0.4308	-0.1529	2.0056
O	4.1194	1.1821	0.2598
O	2.2888	0.7673	-3.0482
P	0.1759	2.4261	0.3716
P	1.6713	-2.1722	0.0959
H	-0.5306	-0.5290	1.9324
H	-3.2611	0.1092	2.0494
O	-2.0391	-1.4304	2.6705
N	-3.2039	-1.3921	0.4709
C	-3.1171	-0.9957	2.1626
H	-4.0884	-1.4628	2.4680
C	-4.3973	-0.8537	-0.0998
C	-4.4342	0.5193	-0.3626
H	-3.5400	1.1148	-0.1862
C	-5.5903	1.1051	-0.8632
H	-5.6027	2.1724	-1.0745
C	-6.7256	0.3305	-1.0986
H	-7.6295	0.7882	-1.4937
C	-6.6912	-1.0339	-0.8271
H	-7.5713	-1.6478	-1.0067
C	-5.5351	-1.6297	-0.3256
H	-5.5265	-2.6977	-0.1251
C	-3.0030	-2.8361	0.3556
H	-3.7569	-3.3797	0.9372
H	-3.0369	-3.1554	-0.6929
H	-2.0223	-3.0629	0.7821
O	-0.9800	-0.3897	-0.6108
C	-1.3280	-0.4971	-1.9459
H	-2.3434	-0.9334	-2.0797
H	-1.3490	0.4807	-2.4753
H	-0.6410	-1.1429	-2.5306
H	-2.3208	-0.9107	0.0928
Na	4.2400	1.4565	-2.0220

Mo-ts-11-9

Gibbs energy: -1494319.9 kcal mol-1

Atom	X	Y	Z

C	-0.5970	2.0623	2.0478
H	-0.7424	2.9693	2.6505
H	-1.6008	1.6566	1.8416
C	0.1974	1.0108	2.8097
H	1.2022	1.3752	3.0674
H	-0.3195	0.7601	3.7530
C	1.0558	-1.2827	2.6700
H	0.5797	-1.4882	3.6441
H	2.0893	-0.9571	2.8579
C	1.0035	-2.5307	1.8011
H	-0.0556	-2.8089	1.6885
H	1.5060	-3.3793	2.2838
C	-1.3139	2.9644	-0.6082
H	-1.8087	1.9930	-0.7715
C	-0.9160	3.5183	-1.9714
H	-1.8004	3.5952	-2.6188
H	-0.1854	2.8782	-2.4831
H	-0.4915	4.5292	-1.8872
C	-2.2718	3.9128	0.1005
H	-1.7931	4.8727	0.3420
H	-2.6764	3.4986	1.0320
H	-3.1277	4.1396	-0.5522
C	1.1600	3.9882	0.6409
H	0.4401	4.8237	0.6461
C	2.1489	4.1839	-0.5067
H	2.6291	5.1697	-0.4352
H	1.6885	4.1016	-1.4980
H	2.9391	3.4233	-0.4315
C	1.9187	3.9854	1.9632
H	2.6013	3.1265	2.0236
H	1.2570	3.9615	2.8368
H	2.5309	4.8943	2.0460
C	0.6691	-3.3977	-0.9389
H	-0.3232	-2.9158	-0.9291
C	0.5524	-4.8045	-0.3665
H	-0.0971	-5.4200	-1.0059
H	0.1255	-4.8250	0.6432
H	1.5281	-5.3095	-0.3258
C	1.1574	-3.4454	-2.3827
H	2.0978	-4.0088	-2.4691
H	1.3189	-2.4436	-2.8023
H	0.4191	-3.9559	-3.0163
C	3.3931	-2.8910	0.0847
H	3.2940	-3.9832	-0.0295
C	4.1415	-2.6013	1.3809
H	5.1731	-2.9729	1.3071
H	3.6870	-3.0813	2.2551
H	4.1974	-1.5211	1.5721
C	4.1873	-2.3333	-1.0936
H	4.3852	-1.2654	-0.9237

H	3.6687	-2.4293	-2.0541
H	5.1568	-2.8441	-1.1774
C	2.9686	0.7748	0.3040
C	1.8564	0.5548	-1.9309
Mo	1.2035	0.2383	-0.1630
N	0.3472	-0.1929	1.9811
O	4.1232	1.1550	0.4074
O	2.4194	0.7952	-2.9771
P	0.1731	2.4160	0.3824
P	1.6574	-2.1840	0.0868
H	-0.6058	-0.5566	1.8367
H	-3.2727	0.0547	2.0357
O	-2.1466	-1.5422	2.6744
N	-3.2492	-1.4038	0.3103
C	-3.1821	-1.0485	2.1839
H	-4.1778	-1.5168	2.3711
C	-4.4295	-0.8303	-0.2133
C	-4.4620	0.5591	-0.3935
H	-3.5589	1.1359	-0.1972
C	-5.6199	1.1835	-0.8390
H	-5.6258	2.2622	-0.9832
C	-6.7669	0.4355	-1.1047
H	-7.6726	0.9243	-1.4560
C	-6.7381	-0.9436	-0.9204
H	-7.6254	-1.5389	-1.1265
C	-5.5806	-1.5797	-0.4750
H	-5.5782	-2.6589	-0.3452
C	-3.0588	-2.8398	0.1768
H	-3.8223	-3.3901	0.7409
H	-3.0802	-3.1583	-0.8746
H	-2.0846	-3.0872	0.6102
O	-0.9401	-0.3726	-0.6627
C	-1.2569	-0.4453	-2.0070
H	-2.2872	-0.8326	-2.1738
H	-1.2174	0.5392	-2.5227
H	-0.5849	-1.1138	-2.5849
H	-2.3672	-0.9070	-0.0047
Na	4.3284	1.4662	-1.8649

Mo-14

Gibbs energy: -1494321.5 kcal mol-1

Atom	X	Y	Z
C	-0.6556	2.0203	2.1430
H	-0.7828	2.9210	2.7601
H	-1.6597	1.5905	1.9906
C	0.2217	0.9759	2.8222
H	1.2058	1.4232	3.0775

H	-0.2444	0.7019	3.7940
C	1.2361	-1.1631	2.6630
H	0.8419	-1.3863	3.6774
H	2.2579	-0.7560	2.8158
C	1.2964	-2.4681	1.8777
H	0.2873	-2.9057	1.8682
H	1.9651	-3.2046	2.3435
C	-1.5667	2.8538	-0.4682
H	-2.0893	1.8835	-0.4785
C	-1.3071	3.2494	-1.9169
H	-2.2478	3.2387	-2.4860
H	-0.6050	2.5655	-2.4126
H	-0.8998	4.2681	-1.9886
C	-2.4515	3.8820	0.2245
H	-1.9645	4.8655	0.2846
H	-2.7325	3.5886	1.2436
H	-3.3815	4.0267	-0.3448
C	0.9277	4.0147	0.6119
H	0.1697	4.8136	0.6610
C	1.8200	4.2445	-0.6062
H	2.2357	5.2616	-0.5913
H	1.2997	4.1033	-1.5609
H	2.6637	3.5408	-0.5680
C	1.7737	4.0653	1.8790
H	2.4932	3.2358	1.9076
H	1.1733	4.0253	2.7949
H	2.3496	5.0010	1.9076
C	0.6962	-3.3877	-0.7923
H	-0.3186	-3.0143	-0.5691
C	0.8115	-4.8165	-0.2768
H	0.0752	-5.4622	-0.7774
H	0.6337	-4.8975	0.8024
H	1.8023	-5.2434	-0.4861
C	0.8941	-3.3381	-2.3029
H	1.8430	-3.8096	-2.5953
H	0.8952	-2.3095	-2.6877
H	0.0948	-3.8929	-2.8146
C	3.5001	-2.7182	-0.1015
H	3.4630	-3.8191	-0.1460
C	4.3807	-2.3027	1.0719
H	5.4190	-2.6129	0.8887
H	4.0693	-2.7525	2.0212
H	4.3806	-1.2117	1.1976
C	4.0960	-2.1810	-1.3997
H	4.2213	-1.0921	-1.3196
H	3.4743	-2.3748	-2.2811
H	5.0875	-2.6201	-1.5779
C	2.8666	0.9010	0.2093
C	1.6574	0.5924	-1.9541
Mo	1.1238	0.2659	-0.1365

N	0.3770	-0.2009	1.9806
O	4.0018	1.3419	0.2449
O	2.1577	0.8518	-3.0302
P	0.0042	2.3947	0.4429
P	1.7399	-2.1150	0.1061
H	-0.9771	-1.0019	2.2683
H	-3.1263	-0.1495	2.2482
O	-1.7358	-1.6149	2.6427
N	-3.1680	-1.6324	0.7580
C	-2.9641	-1.2358	2.1809
H	-3.7446	-1.7293	2.7911
C	-4.2184	-0.9925	0.0611
C	-4.3658	0.4058	0.1299
H	-3.7038	0.9957	0.7604
C	-5.3216	1.0625	-0.6335
H	-5.4015	2.1459	-0.5613
C	-6.1640	0.3531	-1.4898
H	-6.9079	0.8713	-2.0901
C	-6.0341	-1.0292	-1.5574
H	-6.6834	-1.6067	-2.2127
C	-5.0814	-1.6986	-0.7906
H	-5.0129	-2.7792	-0.8711
C	-3.0627	-3.0760	0.6060
H	-3.9505	-3.6064	0.9930
H	-2.9213	-3.3501	-0.4463
H	-2.1882	-3.4115	1.1717
O	-1.0816	-0.4387	-0.7469
C	-1.6038	-0.5523	-2.0560
H	-1.7517	-1.6040	-2.3472
H	-2.5659	-0.0247	-2.1551
H	-0.8830	-0.1016	-2.7463
H	-1.6910	-0.8763	-0.1100
Na	4.0598	1.6853	-2.0304

Mo-ts-14-15

Gibbs energy: -1494320.5 kcal mol-1

Atom	X	Y	Z
C	-0.6153	2.0490	2.1307
H	-0.7277	2.9567	2.7403
H	-1.6257	1.6310	1.9881
C	0.2524	1.0001	2.8154
H	1.2442	1.4357	3.0586
H	-0.2107	0.7417	3.7926
C	1.2431	-1.1527	2.6693
H	0.8552	-1.3616	3.6888
H	2.2694	-0.7534	2.8078
C	1.2825	-2.4655	1.8955

H	0.2682	-2.8908	1.8975
H	1.9457	-3.2052	2.3638
C	-1.5304	2.8706	-0.4832
H	-2.0649	1.9067	-0.4834
C	-1.2728	3.2516	-1.9361
H	-2.2164	3.2488	-2.5006
H	-0.5822	2.5548	-2.4299
H	-0.8526	4.2643	-2.0177
C	-2.3992	3.9150	0.2055
H	-1.9001	4.8929	0.2552
H	-2.6789	3.6332	1.2283
H	-3.3302	4.0663	-0.3605
C	0.9842	4.0094	0.5736
H	0.2366	4.8181	0.6188
C	1.8737	4.2169	-0.6504
H	2.3020	5.2289	-0.6468
H	1.3475	4.0734	-1.6016
H	2.7090	3.5034	-0.6095
C	1.8367	4.0609	1.8363
H	2.5460	3.2228	1.8693
H	1.2401	4.0370	2.7553
H	2.4243	4.9895	1.8537
C	0.6529	-3.4029	-0.7619
H	-0.3559	-3.0144	-0.5375
C	0.7529	-4.8276	-0.2323
H	0.0063	-5.4691	-0.7230
H	0.5789	-4.8954	0.8485
H	1.7374	-5.2691	-0.4415
C	0.8438	-3.3713	-2.2739
H	1.7852	-3.8578	-2.5660
H	0.8560	-2.3468	-2.6694
H	0.0349	-3.9210	-2.7759
C	3.4691	-2.7608	-0.0963
H	3.4181	-3.8616	-0.1317
C	4.3626	-2.3471	1.0681
H	5.3957	-2.6719	0.8806
H	4.0518	-2.7853	2.0230
H	4.3772	-1.2552	1.1848
C	4.0633	-2.2416	-1.4025
H	4.2038	-1.1539	-1.3317
H	3.4332	-2.4337	-2.2783
H	5.0476	-2.6953	-1.5839
C	2.8805	0.8678	0.1913
C	1.6596	0.5576	-1.9644
Mo	1.1287	0.2517	-0.1429
N	0.3869	-0.1880	1.9851
O	4.0212	1.2947	0.2189
O	2.1584	0.8029	-3.0444
P	0.0393	2.3998	0.4237
P	1.7177	-2.1347	0.1176

H	-0.9452	-0.9516	2.2653
H	-3.0887	-0.0760	2.2445
O	-1.7219	-1.5615	2.6427
N	-3.1803	-1.5817	0.7786
C	-2.9479	-1.1665	2.1940
H	-3.7323	-1.6339	2.8202
C	-4.2304	-0.9381	0.0858
C	-4.3642	0.4623	0.1413
H	-3.6917	1.0528	0.7600
C	-5.3207	1.1202	-0.6202
H	-5.3900	2.2050	-0.5580
C	-6.1778	0.4105	-1.4615
H	-6.9222	0.9297	-2.0602
C	-6.0619	-0.9737	-1.5155
H	-6.7230	-1.5517	-2.1585
C	-5.1088	-1.6442	-0.7504
H	-5.0521	-2.7263	-0.8197
C	-3.0964	-3.0286	0.6471
H	-3.9874	-3.5408	1.0508
H	-2.9695	-3.3206	-0.4024
H	-2.2208	-3.3672	1.2093
O	-1.0872	-0.4300	-0.7380
C	-1.6145	-0.5469	-2.0449
H	-1.7768	-1.5989	-2.3272
H	-2.5703	-0.0080	-2.1451
H	-0.8903	-0.1107	-2.7411
H	-1.7014	-0.8572	-0.0980
Na	4.0763	1.6194	-2.0596

Mo_16

Gibbs energy: -1494333.9 kcal mol-1

Atom	X	Y	Z
C	-0.3336	2.1137	2.2930
H	-0.4243	3.0380	2.8795
H	-1.3345	1.6526	2.2698
C	0.6339	1.1375	2.9485
H	1.6512	1.5517	2.9942
H	0.3175	0.9293	3.9863
C	1.5666	-1.1345	2.7438
H	1.2976	-1.3244	3.7986
H	2.5952	-0.7463	2.7287
C	1.4348	-2.4206	1.9393
H	0.3960	-2.7705	2.0267
H	2.0804	-3.2115	2.3433
C	-1.5474	2.8279	-0.2229
H	-2.0390	1.8423	-0.1593
C	-1.4452	3.2053	-1.6962

H	-2.4409	3.1779	-2.1613
H	-0.7983	2.5170	-2.2563
H	-1.0574	4.2267	-1.8248
C	-2.3652	3.8577	0.5457
H	-1.8609	4.8339	0.5916
H	-2.5875	3.5473	1.5745
H	-3.3276	4.0279	0.0415
C	1.0395	4.0618	0.5246
H	0.2821	4.8563	0.6239
C	1.7927	4.2477	-0.7905
H	2.2174	5.2594	-0.8514
H	1.1683	4.0871	-1.6771
H	2.6272	3.5328	-0.8295
C	2.0188	4.1690	1.6876
H	2.7583	3.3568	1.6558
H	1.5255	4.1480	2.6663
H	2.5722	5.1163	1.6248
C	0.5419	-3.3572	-0.6386
H	-0.4250	-2.8926	-0.3850
C	0.5878	-4.7601	-0.0479
H	-0.1690	-5.3953	-0.5309
H	0.3794	-4.7755	1.0287
H	1.5615	-5.2443	-0.2119
C	0.6624	-3.4054	-2.1575
H	1.5477	-3.9773	-2.4714
H	0.7253	-2.4044	-2.6045
H	-0.2146	-3.9071	-2.5905
C	3.4330	-2.8101	-0.2211
H	3.3426	-3.9088	-0.2432
C	4.4312	-2.4155	0.8619
H	5.4339	-2.7792	0.5975
H	4.1846	-2.8314	1.8456
H	4.4937	-1.3227	0.9568
C	3.9364	-2.3177	-1.5753
H	4.1136	-1.2339	-1.5244
H	3.2335	-2.4975	-2.3966
H	4.8894	-2.8019	-1.8301
C	2.9806	0.8845	0.0849
C	1.5717	0.5826	-1.9616
Mo	1.1854	0.2855	-0.1188
N	0.6871	-0.1054	2.1665
O	4.1274	1.2960	0.0186
O	1.9724	0.8237	-3.0797
P	0.1252	2.4277	0.5068
P	1.7257	-2.1200	0.1158
H	-0.2601	-0.5026	2.1532
H	-3.5658	-0.8897	2.3818
O	-1.8082	-1.7406	1.6736
N	-3.9667	-2.0547	0.7165
C	-3.1839	-1.8014	1.8994

H	-3.3590	-2.6276	2.6047
C	-4.5646	-1.0409	-0.0198
C	-4.4599	0.3192	0.3384
H	-3.8650	0.6226	1.1964
C	-5.0821	1.3055	-0.4160
H	-4.9723	2.3445	-0.1065
C	-5.8173	0.9912	-1.5580
H	-6.2943	1.7708	-2.1469
C	-5.9168	-0.3475	-1.9272
H	-6.4786	-0.6272	-2.8169
C	-5.3063	-1.3481	-1.1782
H	-5.4069	-2.3800	-1.5031
C	-3.8651	-3.3861	0.1579
H	-4.8477	-3.8699	0.0585
H	-3.3803	-3.3868	-0.8317
H	-3.2499	-3.9991	0.8235
O	-0.9652	-0.3915	-0.2879
C	-1.5967	-0.5595	-1.5080
H	-2.1960	-1.4952	-1.5447
H	-2.3108	0.2593	-1.7432
H	-0.8891	-0.6083	-2.3579
H	-1.5796	-1.1870	0.8350
Na	4.0038	1.5600	-2.2681

N-methylformanilide-cis

Gibbs energy: -276070.3 kcal mol-1

Atom	X	Y	Z
O	0.4457	-2.0720	2.7195
N	2.3705	-1.7906	1.5093
C	1.1280	-2.3114	1.7379
H	0.7972	-2.9983	0.9368
C	3.1084	-2.1337	0.3488
C	2.4727	-2.2355	-0.8932
H	1.4125	-2.0090	-0.9811
C	3.2034	-2.5940	-2.0220
H	2.6975	-2.6707	-2.9819
C	4.5742	-2.8244	-1.9337
H	5.1438	-3.0921	-2.8204
C	5.2108	-2.6951	-0.7016
H	6.2818	-2.8668	-0.6197
C	4.4858	-2.3563	0.4367
H	4.9898	-2.2769	1.3972
C	2.9834	-0.9748	2.5477
H	3.5900	-1.5791	3.2343
H	3.6126	-0.2077	2.0878
H	2.1889	-0.4970	3.1249

N-methylformanilide-trans

Gibbs energy: -276068.1 kcal mol-1

Atom	X	Y	Z
O	0.2236	-1.3361	2.3446
N	2.3404	-1.8169	1.5346
C	0.9718	-1.7626	1.4851
H	0.5918	-2.1675	0.5257
C	3.1005	-1.3542	2.6432
C	2.5938	-1.4069	3.9479
H	1.5941	-1.7861	4.1252
C	3.3728	-0.9655	5.0118
H	2.9650	-1.0145	6.0192
C	4.6585	-0.4713	4.8026
H	5.2597	-0.1279	5.6411
C	5.1648	-0.4280	3.5077
H	6.1671	-0.0478	3.3227
C	4.3954	-0.8662	2.4334
H	4.8102	-0.8155	1.4305
C	3.0474	-2.2619	0.3422
H	3.4782	-1.4232	-0.2187
H	3.8473	-2.9582	0.6143
H	2.3464	-2.7839	-0.3147

Hemiaminal

Gibbs energy: -276797.3 kcal mol-1

Atom	X	Y	Z
O	2.4214	-1.1091	-1.1789
N	1.2476	0.2751	0.2886
C	2.1260	-0.8342	0.1873
H	1.6719	-1.7121	0.6701
C	-0.1252	0.1196	0.0956
C	-0.6934	-1.1262	-0.2278
H	-0.0580	-1.9883	-0.4086
C	-2.0695	-1.2653	-0.3629
H	-2.4759	-2.2430	-0.6157
C	-2.9231	-0.1753	-0.2028
H	-3.9984	-0.2901	-0.3162
C	-2.3661	1.0663	0.0899
H	-3.0078	1.9372	0.2103
C	-0.9906	1.2195	0.2347
H	-0.5924	2.2028	0.4701
C	1.8294	1.6001	0.1887
H	1.5355	2.1074	-0.7415
H	1.5453	2.2365	1.0379

H	2.9191	1.5106	0.1881
H	3.0511	-0.5953	0.7325
H	3.0547	-1.8361	-1.2239

Formaldehyde

Gibbs energy: -71830.5 kcal mol-1

Atom	X	Y	Z
C	-0.6764	-0.6865	0.0000
H	-0.1558	-1.6680	0.0000
H	-1.7866	-0.7269	0.0000
O	-0.0736	0.3578	0.0000

Methanol

Gibbs energy: -72580.3 kcal mol-1

Atom	X	Y	Z
C	1.1283	0.1894	0.0103
H	1.5014	-0.8390	0.0261
H	1.4892	0.6698	-0.9113
H	0.0290	0.1539	-0.0169
O	1.6142	0.8284	1.1723
H	1.2989	1.7384	1.1807

N-methylaniline

Gibbs energy: -204967.6 kcal mol-1

Atom	X	Y	Z
H	1.3756	-1.3179	-0.4958
N	1.8702	-1.6819	0.3064
C	3.1926	-2.0132	0.0837
C	4.0999	-2.2040	1.1384
H	3.7775	-2.0690	2.1682
C	5.4182	-2.5694	0.8726
H	6.1026	-2.7130	1.7072
C	5.8678	-2.7442	-0.4326
H	6.8989	-3.0263	-0.6310
C	4.9686	-2.5486	-1.4837
H	5.2986	-2.6783	-2.5130
C	3.6526	-2.1892	-1.2347
H	2.9569	-2.0447	-2.0615
C	1.3968	-1.2490	1.5976
H	1.9534	-0.3826	1.9923
H	0.3433	-0.9703	1.5131

H	1.4629	-2.0601	2.3347

Mo-ts-6-7NH

Gibbs energy: -1397104.0 kcal mol-1

Atom	X	Y	Z
C	-3.0039	-0.5497	1.9709
H	-4.0800	-0.6055	2.1839
H	-2.5108	-1.3206	2.5810
C	-2.4466	0.8113	2.3598
H	-2.9864	1.6244	1.8526
H	-2.5612	0.9655	3.4480
C	-0.3950	2.1389	2.4605
H	-0.4850	2.2113	3.5592
H	-0.9435	2.9902	2.0315
C	1.0714	2.1712	2.0586
H	1.5894	1.3310	2.5477
H	1.5546	3.0929	2.4107
C	-2.4243	-2.8112	0.2976
H	-1.6783	-2.8861	1.1061
C	-1.8105	-3.4179	-0.9590
H	-1.4107	-4.4193	-0.7457
H	-0.9949	-2.7989	-1.3552
H	-2.5553	-3.5345	-1.7578
C	-3.6680	-3.5767	0.7298
H	-4.4272	-3.5935	-0.0644
H	-4.1317	-3.1590	1.6327
H	-3.4128	-4.6231	0.9484
C	-4.2692	-0.6966	-0.6659
H	-4.9594	-1.4497	-0.2536
C	-4.1244	-0.9132	-2.1671
H	-5.0861	-0.7580	-2.6758
H	-3.7739	-1.9212	-2.4200
H	-3.4000	-0.1999	-2.5862
C	-4.8407	0.6864	-0.3798
H	-4.1609	1.4783	-0.7193
H	-5.0436	0.8461	0.6864
H	-5.7928	0.8152	-0.9131
C	3.0107	1.2070	0.1634
H	2.8545	0.2302	0.6460
C	4.0694	1.9744	0.9445
H	5.0154	1.4129	0.9350
H	3.7967	2.1332	1.9950
H	4.2800	2.9569	0.4991
C	3.4748	0.9435	-1.2632
H	3.7336	1.8795	-1.7797
H	2.7130	0.4159	-1.8515
H	4.3795	0.3193	-1.2567

C	1.4821	3.6372	-0.4762
H	2.5062	3.9614	-0.2296
C	0.4957	4.6268	0.1321
H	0.5849	5.6025	-0.3657
H	0.6646	4.7935	1.2024
H	-0.5398	4.2846	-0.0056
C	1.3138	3.6272	-1.9952
H	0.2479	3.5061	-2.2354
H	1.8831	2.8299	-2.4899
H	1.6370	4.5862	-2.4233
C	-1.6488	1.8448	-1.2254
C	-0.2161	-0.1246	-2.0714
Mo	-0.6570	0.4741	-0.3114
N	-1.0304	0.9064	1.9755
O	-2.1565	2.6249	-2.0075
O	-0.0071	-0.4002	-3.2320
P	-2.6193	-0.9481	0.1830
P	1.2767	1.9059	0.2203
H	-0.5403	0.1036	2.4046
H	0.4486	-0.8843	0.5163
O	0.3204	-1.5171	2.8532
N	2.2462	-1.9426	1.7078
C	0.8548	-1.9458	1.8085
H	0.3942	-2.7394	1.1950
C	3.0033	-2.3170	0.6043
C	2.4160	-2.6920	-0.6127
H	1.3362	-2.6677	-0.7330
C	3.2239	-3.0561	-1.6868
H	2.7504	-3.3384	-2.6253
C	4.6125	-3.0424	-1.5816
H	5.2332	-3.3266	-2.4282
C	5.1957	-2.6508	-0.3757
H	6.2792	-2.6270	-0.2744
C	4.4028	-2.2901	0.7061
H	4.8603	-1.9858	1.6480
H	2.7176	-1.4627	2.4672
Na	-0.9710	1.6625	-3.7622

Mo-12

Gibbs energy: -1396396.2 kcal mol-1

Atom	X	Y	Z
P	-0.1916	2.3922	-0.0846
P	1.6080	-2.0944	-0.0351
O	2.7072	0.9560	2.8199
O	-0.7774	-0.4159	1.7332
N	-0.2566	-0.2572	-1.6263
H	-1.1215	-0.5938	-1.1734

N	-2.4267	-0.9224	0.2317
C	2.1057	0.7196	1.7980
C	-1.2412	1.9921	-1.5807
H	-2.1948	1.5982	-1.1953
H	-1.4804	2.8822	-2.1778
C	-0.5853	0.9209	-2.4399
H	-1.2615	0.6413	-3.2678
H	0.3463	1.2915	-2.8933
C	0.3241	-1.3496	-2.4169
H	1.2548	-0.9808	-2.8736
H	-0.3541	-1.6311	-3.2431
C	0.5905	-2.5598	-1.5340
H	1.0405	-3.3773	-2.1135
H	-0.3698	-2.9336	-1.1465
C	0.6847	3.9961	-0.4934
H	-0.0514	4.8089	-0.3766
C	1.2063	4.0135	-1.9263
H	1.7496	4.9504	-2.1109
H	0.4056	3.9521	-2.6729
H	1.9121	3.1916	-2.1032
C	1.8401	4.2160	0.4804
H	2.5973	3.4294	0.3647
H	1.5267	4.2091	1.5309
H	2.3228	5.1829	0.2817
C	-1.5277	2.9084	1.1307
H	-2.0356	1.9488	1.3221
C	-0.9325	3.4020	2.4445
H	-0.5447	4.4252	2.3464
H	-0.0950	2.7762	2.7865
H	-1.6969	3.4251	3.2334
C	-2.5571	3.8994	0.6020
H	-3.3135	4.1086	1.3721
H	-3.0907	3.5244	-0.2792
H	-2.0994	4.8620	0.3353
C	3.3465	-2.6770	-0.4122
H	3.3516	-3.7737	-0.2987
C	3.7698	-2.3322	-1.8358
H	3.7612	-1.2474	-2.0019
H	3.1372	-2.8048	-2.5970
H	4.7981	-2.6780	-2.0097
C	4.3256	-2.0597	0.5831
H	5.3424	-2.4309	0.3944
H	4.0773	-2.2844	1.6271
H	4.3402	-0.9664	0.4823
C	0.9611	-3.3532	1.1988
H	-0.0812	-3.0158	1.3239
C	0.9530	-4.8010	0.7250
H	1.9710	-5.1809	0.5628
H	0.3908	-4.9403	-0.2057
H	0.4887	-5.4477	1.4833

C	1.6646	-3.2384	2.5467
H	1.0810	-3.7342	3.3346
H	1.8369	-2.1925	2.8394
H	2.6495	-3.7250	2.5250
C	-1.9820	-0.7212	1.4373
H	-2.6827	-0.8371	2.2940
C	-3.7935	-1.0971	0.0215
C	-4.2117	-2.0358	-0.9365
H	-3.4504	-2.6199	-1.4534
C	-5.5603	-2.2221	-1.2129
H	-5.8595	-2.9610	-1.9543
C	-6.5299	-1.4675	-0.5499
H	-7.5853	-1.6105	-0.7708
C	-6.1273	-0.5164	0.3846
H	-6.8711	0.0952	0.8926
C	-4.7760	-0.3226	0.6631
H	-4.4717	0.4540	1.3648
C	2.5228	0.8698	-0.8039
O	3.4378	1.2303	-1.4693
Mo	1.0451	0.2861	0.2774
Na	0.4648	0.0663	3.4973

Mo-12-alt1

Gibbs energy: -1396395.1 kcal mol-1

Atom	X	Y	Z
P	2.5900	-0.2298	0.1784
P	-2.0057	1.3782	0.2547
O	-0.1902	-0.4709	-3.1971
O	0.2138	-1.0534	3.1265
N	0.6111	1.3949	1.8674
H	0.3904	0.5862	2.4760
N	-0.4915	-1.5701	0.9821
C	-0.0677	-0.2431	-2.0142
C	2.8936	0.5081	1.8650
H	2.5995	-0.2660	2.5884
H	3.9536	0.7348	2.0414
C	2.0209	1.7341	2.0950
H	2.1651	2.1038	3.1263
H	2.2906	2.5554	1.4138
C	-0.3013	2.4986	2.1787
H	-0.0459	3.3468	1.5258
H	-0.1589	2.8359	3.2211
C	-1.7449	2.0632	1.9718
H	-2.4413	2.8774	2.2140
H	-1.9674	1.2352	2.6635
C	3.9922	0.4223	-0.8861
H	4.8749	-0.1946	-0.6512

C	4.3295	1.8802	-0.5937
H	5.1174	2.2243	-1.2782
H	4.6968	2.0395	0.4264
H	3.4570	2.5279	-0.7517
C	3.6616	0.2697	-2.3691
H	2.8872	1.0005	-2.6412
H	3.2961	-0.7276	-2.6377
H	4.5487	0.4833	-2.9816
C	3.0690	-2.0185	0.4634
H	2.2080	-2.3991	1.0369
C	3.1436	-2.7891	-0.8502
H	4.0654	-2.5519	-1.4003
H	2.2924	-2.5776	-1.5107
H	3.1526	-3.8700	-0.6567
C	4.3380	-2.2303	1.2797
H	4.5353	-3.3054	1.3936
H	4.2705	-1.8065	2.2880
H	5.2201	-1.7948	0.7887
C	-2.7055	2.8080	-0.7370
H	-3.7738	2.8772	-0.4759
C	-2.0422	4.1404	-0.4089
H	-0.9591	4.0995	-0.5854
H	-2.2119	4.4585	0.6263
H	-2.4478	4.9280	-1.0593
C	-2.5794	2.5302	-2.2335
H	-3.1577	3.2671	-2.8082
H	-2.9206	1.5294	-2.5222
H	-1.5265	2.6317	-2.5306
C	-3.4925	0.2765	0.5318
H	-3.0628	-0.5196	1.1611
C	-4.6525	0.9187	1.2821
H	-5.1179	1.7293	0.7040
H	-4.3575	1.3255	2.2566
H	-5.4376	0.1720	1.4665
C	-3.9699	-0.3602	-0.7684
H	-4.7031	-1.1504	-0.5568
H	-3.1471	-0.8149	-1.3370
H	-4.4715	0.3763	-1.4127
C	-0.3027	-1.8147	2.2796
H	-0.6219	-2.8241	2.6265
C	-1.2157	-2.5765	0.3071
C	-0.7740	-3.0690	-0.9297
H	0.1471	-2.6678	-1.3454
C	-1.4934	-4.0377	-1.6188
H	-1.1242	-4.3935	-2.5793
C	-2.6753	-4.5573	-1.0884
H	-3.2394	-5.3134	-1.6304
C	-3.1207	-4.0910	0.1452
H	-4.0425	-4.4789	0.5764
C	-2.4062	-3.1102	0.8308

H	-2.7873	-2.7332	1.7795
Mo	0.2256	0.4249	-0.2481
C	0.7498	1.9398	-1.2554
O	1.0444	2.7983	-2.0667
Na	0.5442	1.5597	-3.9410

Mo-12-alt2

Gibbs energy: -1396393.8 kcal mol-1

Atom	X	Y	Z
P	-0.1816	2.4226	-0.1961
P	1.5764	-2.0917	-0.0265
O	3.0134	1.0790	2.5316
O	-0.6858	-0.3701	1.5146
N	-0.2224	-0.2366	-1.6800
H	-1.1112	-0.5089	-1.2196
N	-2.4394	-0.8194	0.1079
C	2.1820	0.7666	1.7107
C	-1.1421	2.0426	-1.7581
H	-2.1489	1.7267	-1.4477
H	-1.2658	2.9348	-2.3871
C	-0.4883	0.9173	-2.5485
H	-1.1431	0.6241	-3.3890
H	0.4709	1.2425	-2.9798
C	0.2886	-1.3964	-2.4205
H	1.2285	-1.1004	-2.9101
H	-0.4181	-1.6831	-3.2206
C	0.5045	-2.5731	-1.4796
H	0.8953	-3.4449	-2.0220
H	-0.4676	-2.8680	-1.0541
C	0.6190	4.0642	-0.5971
H	-0.2001	4.7798	-0.7714
C	1.4604	3.9785	-1.8644
H	1.8980	4.9614	-2.0883
H	0.8741	3.6798	-2.7421
H	2.2852	3.2631	-1.7542
C	1.4641	4.5473	0.5739
H	2.2189	3.7923	0.8421
H	0.8708	4.7418	1.4750
H	1.9892	5.4772	0.3150
C	-1.5831	2.8374	0.9790
H	-2.2535	1.9756	0.8239
C	-1.1378	2.8132	2.4365
H	-0.4809	3.6591	2.6804
H	-0.6022	1.8865	2.6738
H	-2.0126	2.8831	3.0986
C	-2.3513	4.1085	0.6432
H	-3.2501	4.1838	1.2710

H	-2.6826	4.1414	-0.4031
H	-1.7509	5.0083	0.8366
C	3.2849	-2.7256	-0.4528
H	3.2714	-3.8168	-0.3002
C	3.6610	-2.4390	-1.9019
H	3.6285	-1.3613	-2.1127
H	3.0110	-2.9510	-2.6211
H	4.6881	-2.7784	-2.0948
C	4.3222	-2.1017	0.4770
H	5.3049	-2.5681	0.3220
H	4.0675	-2.1911	1.5392
H	4.4200	-1.0317	0.2451
C	0.9428	-3.2715	1.2793
H	-0.0953	-2.9223	1.3971
C	0.9257	-4.7409	0.8786
H	1.9412	-5.1478	0.7747
H	0.3916	-4.9216	-0.0620
H	0.4246	-5.3353	1.6554
C	1.6535	-3.0758	2.6131
H	1.1109	-3.6000	3.4113
H	1.7214	-2.0163	2.8921
H	2.6698	-3.4947	2.5902
C	-1.8865	-0.6927	1.2930
H	-2.5042	-0.9128	2.1930
C	-3.8052	-1.0594	0.0107
C	-4.2670	-1.8846	-1.0313
H	-3.5276	-2.3285	-1.6990
C	-5.6219	-2.1376	-1.2029
H	-5.9485	-2.7854	-2.0150
C	-6.5633	-1.5697	-0.3422
H	-7.6248	-1.7653	-0.4776
C	-6.1232	-0.7353	0.6833
H	-6.8459	-0.2689	1.3515
C	-4.7664	-0.4724	0.8559
H	-4.4439	0.2131	1.6392
C	2.6379	0.8693	-0.7413
O	3.7132	1.2471	-1.1725
Mo	1.0913	0.3224	0.2018
Na	4.5333	1.7368	0.9183

Fe-ts-10-11

Gibbs energy: -1332174.4 kcal mol-1

Atom	X	Y	Z
C	-0.6918	1.9711	1.8870
H	-0.9211	2.9030	2.4203
H	-1.6428	1.4367	1.7367
C	0.2653	1.0904	2.6644

H	1.1799	1.6400	2.9255
H	-0.1928	0.7461	3.6066
C	1.6827	-0.8704	2.5400
H	1.3201	-1.1536	3.5412
H	2.5789	-0.2469	2.6639
C	1.9589	-2.1036	1.7063
H	1.0422	-2.7118	1.6857
H	2.7677	-2.7205	2.1172
C	-1.4873	2.6169	-0.8255
H	-1.9405	1.6166	-0.9092
C	-1.1433	3.1027	-2.2292
H	-2.0456	3.0925	-2.8556
H	-0.3899	2.4783	-2.7243
H	-0.7719	4.1368	-2.2153
C	-2.4833	3.5635	-0.1657
H	-2.0439	4.5548	0.0154
H	-2.8668	3.1879	0.7903
H	-3.3474	3.7123	-0.8287
C	0.9515	3.8667	0.2971
H	0.1844	4.6567	0.2419
C	1.9141	4.0174	-0.8773
H	2.3820	5.0108	-0.8490
H	1.4303	3.9055	-1.8533
H	2.7160	3.2689	-0.8159
C	1.7188	4.0271	1.6045
H	2.4660	3.2300	1.7248
H	1.0690	4.0285	2.4873
H	2.2612	4.9822	1.5962
C	1.7246	-3.0325	-1.0217
H	0.6278	-2.9745	-0.9143
C	2.2044	-4.3700	-0.4690
H	1.8189	-5.1893	-1.0917
H	1.8653	-4.5535	0.5567
H	3.3006	-4.4464	-0.4824
C	2.0799	-2.9107	-2.4995
H	3.1519	-3.0874	-2.6646
H	1.8313	-1.9281	-2.9189
H	1.5362	-3.6675	-3.0810
C	4.1367	-1.5139	-0.2061
H	4.4483	-2.5649	-0.3217
C	4.8278	-0.9406	1.0262
H	5.9146	-0.9347	0.8665
H	4.6359	-1.5191	1.9373
H	4.5185	0.0984	1.2050
C	4.5676	-0.7254	-1.4385
H	4.3258	0.3393	-1.3177
H	4.0951	-1.0714	-2.3639
H	5.6554	-0.8045	-1.5701
C	1.5650	0.7258	-1.7467
N	0.6529	-0.0731	1.8476

O	1.8984	1.0089	-2.8344
P	0.0231	2.2482	0.2020
P	2.2759	-1.5517	-0.0303
H	-0.1641	-0.7144	1.8403
H	-2.6409	-0.5320	2.2970
O	-1.2568	-2.0453	2.5712
N	-2.5993	-1.8169	0.5992
C	-2.4230	-1.6291	2.2015
H	-3.3204	-2.1766	2.5853
C	-3.8707	-1.4095	0.0943
C	-4.1590	-0.0457	0.0127
H	-3.3999	0.6806	0.3000
C	-5.4062	0.3710	-0.4438
H	-5.6257	1.4350	-0.5092
C	-6.3679	-0.5671	-0.8177
H	-7.3405	-0.2385	-1.1770
C	-6.0779	-1.9277	-0.7273
H	-6.8239	-2.6653	-1.0153
C	-4.8339	-2.3519	-0.2662
H	-4.6011	-3.4138	-0.1915
O	-0.7030	-0.6197	-0.5673
C	-1.0403	-0.8086	-1.9003
H	-1.9505	-1.4352	-1.9983
H	-1.2601	0.1389	-2.4338
H	-0.2508	-1.3205	-2.4818
H	-1.7114	-1.2552	0.1317
Fe	1.0994	0.3386	-0.1465
H	-2.4493	-2.8158	0.4440
H	2.3575	1.0360	0.3490

Formanilide-cis

Gibbs energy: -251435.1 kcal mol-1

Atom	X	Y	Z
O	2.7670	0.9553	0.0000
N	1.4095	-0.9097	0.0001
C	2.6025	-0.2508	0.0000
H	3.4486	-0.9667	0.0002
C	0.1013	-0.3870	0.0000
C	-0.9546	-1.3060	0.0001
H	-0.7361	-2.3737	0.0001
C	-2.2709	-0.8634	0.0000
H	-3.0793	-1.5913	0.0001
C	-2.5524	0.5019	-0.0000
H	-3.5823	0.8506	-0.0001
C	-1.4994	1.4122	-0.0001
H	-1.7054	2.4805	-0.0001
C	-0.1738	0.9843	-0.0000

H	0.6413	1.6988	-0.0001
H	1.4767	-1.9211	0.0002

Formanilide-trans

Gibbs energy: -251435.0 kcal mol-1

Atom	X	Y	Z
O	3.6120	0.0377	0.0816
N	1.4165	-0.4812	-0.2206
C	2.4332	0.3182	0.2050
H	2.0858	1.2487	0.6925
C	0.0381	-0.2106	-0.1175
C	-0.4548	1.0965	-0.1593
H	0.2208	1.9351	-0.3105
C	-1.8230	1.3217	-0.0354
H	-2.1977	2.3424	-0.0647
C	-2.7102	0.2565	0.0978
H	-3.7786	0.4397	0.1817
C	-2.2166	-1.0467	0.1104
H	-2.8982	-1.8887	0.2080
C	-0.8499	-1.2820	0.0122
H	-0.4595	-2.2982	0.0445
H	1.6977	-1.3904	-0.5760

Aniline

Gibbs energy: --180334.1 kcal mol-1

Atom	X	Y	Z
H	2.0548	-1.5298	0.1503
N	1.7858	-0.6094	-0.1712
C	0.4364	-0.2962	-0.0672
C	0.0114	1.0406	-0.0631
H	0.7552	1.8357	-0.1074
C	-1.3432	1.3488	-0.0086
H	-1.6502	2.3931	-0.0050
C	-2.3041	0.3395	0.0424
H	-3.3624	0.5851	0.0858
C	-1.8839	-0.9897	0.0371
H	-2.6176	-1.7928	0.0769
C	-0.5316	-1.3096	-0.0170
H	-0.2132	-2.3517	-0.0250
H	2.4337	0.1097	0.1216

Mo-ts-6-7-Me

Gibbs energy: -1446373.7 kcal mol-1

Atom	X	Y	Z
C	2.8043	-1.0675	-2.1420
H	3.8522	-1.2536	-2.4138
H	2.1976	-1.8537	-2.6146
C	2.3436	0.2847	-2.6658
H	2.9893	1.0972	-2.3012
H	2.3899	0.2932	-3.7698
C	0.4048	1.7767	-2.8064
H	0.4175	1.7058	-3.9089
H	1.0502	2.6222	-2.5260
C	-1.0202	1.9895	-2.3167
H	-1.6392	1.1420	-2.6520
H	-1.4540	2.8980	-2.7568
C	2.1356	-3.0386	-0.1588
H	1.3219	-3.1298	-0.8971
C	1.5738	-3.4221	1.2060
H	1.0379	-4.3798	1.1455
H	0.8787	-2.6649	1.5922
H	2.3709	-3.5488	1.9511
C	3.2614	-3.9769	-0.5733
H	4.0826	-3.9669	0.1568
H	3.6803	-3.7302	-1.5574
H	2.8939	-5.0113	-0.6251
C	4.2458	-1.0357	0.4081
H	4.8268	-1.8956	0.0382
C	4.1951	-1.0773	1.9302
H	5.1987	-0.9388	2.3560
H	3.7979	-2.0236	2.3164
H	3.5505	-0.2722	2.3125
C	4.9224	0.2432	-0.0688
H	4.3606	1.1328	0.2434
H	5.0354	0.2784	-1.1595
H	5.9299	0.3168	0.3637
C	-2.8642	1.4322	-0.1739
H	-2.8155	0.4073	-0.5730
C	-3.9343	2.2085	-0.9303
H	-4.9085	1.7109	-0.8123
H	-3.7342	2.2833	-2.0067
H	-4.0530	3.2287	-0.5391
C	-3.2146	1.3275	1.3050
H	-3.3684	2.3200	1.7534
H	-2.4375	0.8004	1.8735
H	-4.1532	0.7695	1.4302
C	-1.1161	3.8007	0.0140
H	-2.1253	4.1801	-0.2137
C	-0.0980	4.6099	-0.7806
H	-0.0766	5.6476	-0.4193
H	-0.3254	4.6457	-1.8525

H	0.9142	4.1999	-0.6559
C	-0.8417	3.9705	1.5074
H	0.2249	3.7835	1.6976
H	-1.4381	3.2979	2.1371
H	-1.0527	5.0015	1.8233
C	1.9255	1.8287	0.8012
C	0.3806	0.1257	1.9782
Mo	0.7458	0.4626	0.1347
N	0.9737	0.5620	-2.2075
O	2.5559	2.6457	1.4435
O	0.2246	0.0095	3.1737
P	2.5205	-1.2072	-0.2974
P	-1.0984	1.9830	-0.4491
H	0.3893	-0.2424	-2.4886
H	-0.5475	-0.8555	-0.4461
O	-0.5704	-1.8841	-2.6109
N	-2.5231	-1.8853	-1.3714
C	-1.1289	-2.0564	-1.5074
H	-0.7459	-2.7868	-0.7751
C	-3.1769	-2.1334	-0.1613
C	-2.4611	-2.2905	1.0413
H	-1.3826	-2.1656	1.0589
C	-3.1330	-2.5407	2.2321
H	-2.5506	-2.6477	3.1458
C	-4.5234	-2.6274	2.2722
H	-5.0411	-2.8215	3.2087
C	-5.2375	-2.4456	1.0908
H	-6.3249	-2.4983	1.0955
C	-4.5805	-2.1966	-0.1109
H	-5.1686	-2.0648	-1.0151
C	-3.2761	-1.4406	-2.5252
H	-3.9397	-2.2315	-2.9021
H	-3.8871	-0.5566	-2.2925
H	-2.5635	-1.1920	-3.3146
Na	1.4061	2.0284	3.3711

Mo-ts-CNMe

Gibbs energy: -1446371.4 kcal mol-1

Atom	X	Y	Z
Mo	0.4109	-0.4799	0.3258
P	2.7433	0.4272	-0.0617
P	-1.7809	-1.5579	-0.2516
O	0.5913	-1.1704	3.3565
O	0.0861	2.7599	-2.0378
N	0.3591	-0.1041	-1.9913
H	0.0265	0.8727	-2.0487
N	-0.6092	1.9406	0.5090

C	0.4720	-0.7986	2.2123
C	2.6628	0.8158	-1.8808
H	2.2836	1.8447	-1.9661
H	3.6530	0.7857	-2.3563
C	1.6971	-0.1050	-2.6125
H	1.6214	0.2124	-3.6676
H	2.0617	-1.1428	-2.6098
C	-0.5531	-1.0006	-2.7239
H	-0.0609	-1.9826	-2.7781
H	-0.6783	-0.6473	-3.7623
C	-1.9183	-1.1343	-2.0625
H	-2.5328	-1.8596	-2.6144
H	-2.4492	-0.1733	-2.1047
C	4.2199	-0.7298	0.0991
H	5.1020	-0.0721	0.0490
C	4.3400	-1.7447	-1.0318
H	5.2236	-2.3770	-0.8645
H	4.4637	-1.2733	-2.0137
H	3.4696	-2.4110	-1.0715
C	4.2394	-1.4521	1.4470
H	3.5524	-2.3065	1.4160
H	3.9610	-0.8114	2.2911
H	5.2440	-1.8512	1.6458
C	3.4937	2.0104	0.6315
H	2.6585	2.7224	0.5876
C	3.8825	1.8559	2.0981
H	4.8009	1.2641	2.2141
H	3.0910	1.3788	2.6928
H	4.0805	2.8417	2.5400
C	4.6382	2.6022	-0.1819
H	4.9868	3.5319	0.2894
H	4.3390	2.8524	-1.2063
H	5.5058	1.9305	-0.2370
C	-1.9504	-3.4309	-0.2164
H	-3.0277	-3.6114	-0.3529
C	-1.2181	-4.1491	-1.3441
H	-0.1376	-3.9608	-1.3101
H	-1.5900	-3.8685	-2.3364
H	-1.3603	-5.2347	-1.2429
C	-1.5333	-4.0244	1.1324
H	-2.0321	-4.9893	1.2963
H	-1.7712	-3.3785	1.9868
H	-0.4533	-4.2196	1.1211
C	-3.4577	-1.0371	0.4034
H	-3.4077	0.0532	0.3187
C	-4.6649	-1.5076	-0.3972
H	-4.8155	-2.5945	-0.3353
H	-4.6045	-1.2328	-1.4577
H	-5.5739	-1.0372	0.0043
C	-3.6149	-1.3683	1.8824

H	-4.4926	-0.8451	2.2868
H	-2.7419	-1.0511	2.4699
H	-3.7766	-2.4432	2.0485
C	0.1827	3.2521	-0.8886
C	-1.9940	2.0872	0.4342
C	-2.8317	2.3217	1.5490
H	-2.4118	2.3594	2.5506
C	-4.2103	2.4781	1.4057
H	-4.8164	2.6384	2.2972
C	-4.8158	2.4356	0.1531
H	-5.8908	2.5645	0.0465
C	-4.0009	2.2380	-0.9660
H	-4.4413	2.2184	-1.9631
C	-2.6324	2.0606	-0.8311
H	-2.0135	1.9455	-1.7176
H	1.1404	3.1574	-0.3370
C	-0.0797	2.3587	1.7981
H	-0.3575	1.6776	2.6193
H	1.0152	2.3715	1.7613
H	-0.4146	3.3714	2.0931
C	1.1649	-2.2010	0.3055
O	1.6312	-3.3125	0.4745
C	-0.5596	4.5204	-0.5605
H	-0.5837	4.7451	0.5120
H	-0.0315	5.3415	-1.0655
H	-1.5817	4.4904	-0.9542
Na	1.3446	-3.2686	2.7761

N-methylacetanilide-cis

Gibbs energy: -300715.1 kcal mol-1

Atom	X	Y	Z
O	3.2775	-0.5552	-0.5262
N	1.2206	0.1083	0.1409
C	2.0624	-0.7241	-0.5552
C	-0.2023	0.0090	0.0985
C	-0.8583	-1.0229	0.7697
H	-0.2715	-1.7545	1.3227
C	-2.2480	-1.1038	0.7304
H	-2.7560	-1.9126	1.2510
C	-2.9863	-0.1450	0.0395
H	-4.0719	-0.2068	0.0149
C	-2.3308	0.8962	-0.6157
H	-2.9030	1.6479	-1.1551
C	-0.9412	0.9732	-0.5899
H	-0.4176	1.7762	-1.1066
C	1.8097	1.2486	0.8342
H	2.1162	2.0367	0.1335

H	1.0756	1.6564	1.5337
H	2.6960	0.9279	1.3886
C	1.4366	-1.8405	-1.3487
H	1.1109	-2.6539	-0.6901
H	0.5625	-1.5120	-1.9209
H	2.1936	-2.2347	-2.0303

N-methylacetanilide-trans

Gibbs energy: -300713.9 kcal mol-1

Atom	X	Y	Z
O	0.2236	-1.3361	2.3446
N	2.3404	-1.8169	1.5346
C	0.9718	-1.7626	1.4851
H	0.5918	-2.1675	0.5257
C	3.1005	-1.3542	2.6432
C	2.5938	-1.4069	3.9479
H	1.5941	-1.7861	4.1252
C	3.3728	-0.9655	5.0118
H	2.9650	-1.0145	6.0192
C	4.6585	-0.4713	4.8026
H	5.2597	-0.1279	5.6411
C	5.1648	-0.4280	3.5077
H	6.1671	-0.0478	3.3227
C	4.3954	-0.8662	2.4334
H	4.8102	-0.8155	1.4305
C	3.0474	-2.2619	0.3422
H	3.4782	-1.4232	-0.2187
H	3.8473	-2.9582	0.6143
H	2.3464	-2.7839	-0.3147

Acetaldehyde

Gibbs energy: -96481.4 kcal mol-1

Atom	X	Y	Z
C	-0.0324	0.5273	-0.0010
H	0.7975	0.9588	-0.6076
O	0.1276	-0.5209	0.5858
C	-1.2892	1.3267	0.0138
H	-2.0573	0.8462	0.6259
H	-1.0782	2.3336	0.3968
H	-1.6531	1.4558	-1.0137

Ethanol

Gibbs energy: -97224.1 kcal mol-1

Atom	X	Y	Z
C	-3.8724	-1.2038	0.0224
H	-3.5229	-2.2425	-0.0040
H	-3.5179	-0.6981	-0.8835
H	-4.9686	-1.2088	0.0038
C	-3.3653	-0.5028	1.2548
H	-3.7151	-1.0236	2.1615
H	-2.2629	-0.5124	1.2731
O	-3.8509	0.8320	1.2292
H	-3.5357	1.2965	2.0119

Pre_hydride_transfer

Gibbs energy: -1217505.7 kcal mol-1

Atom	X	Y	Z
C	-2.5540	-1.3971	1.5035
H	-3.4722	-1.2927	2.0982
H	-2.5960	-2.3883	1.0253
C	-1.3244	-1.3345	2.3986
H	-1.2925	-0.3932	2.9664
H	-1.3480	-2.1636	3.1295
C	1.1297	-1.4446	2.3767
H	1.0880	-2.2672	3.1139
H	1.2019	-0.5000	2.9350
C	2.3264	-1.6334	1.4562
H	2.2529	-2.6232	0.9793
H	3.2642	-1.6270	2.0278
C	-3.3945	-1.0365	-1.2310
H	-2.8509	-1.9959	-1.2455
C	-3.1982	-0.3918	-2.5975
H	-3.5953	-1.0464	-3.3860
H	-2.1359	-0.2122	-2.8029
H	-3.7261	0.5676	-2.6811
C	-4.8657	-1.3085	-0.9466
H	-5.4632	-0.3871	-0.9864
H	-5.0317	-1.7749	0.0336
H	-5.2802	-1.9876	-1.7050
C	-3.6702	1.1768	0.7165
H	-4.6543	0.6899	0.8062
C	-3.7656	2.3153	-0.2910
H	-4.4023	3.1236	0.0950
H	-4.1802	1.9987	-1.2548
H	-2.7672	2.7357	-0.4885
C	-3.2721	1.7163	2.0843
H	-2.2733	2.1714	2.0581
H	-3.2687	0.9408	2.8601

H	-3.9844	2.4897	2.4041
C	3.1492	-1.3890	-1.2786
H	2.4216	-2.2111	-1.3848
C	4.5116	-1.9721	-0.9254
H	4.8275	-2.6900	-1.6955
H	4.5179	-2.5027	0.0351
H	5.2863	-1.1940	-0.8849
C	3.2026	-0.6479	-2.6082
H	3.9783	0.1307	-2.6026
H	2.2409	-0.1781	-2.8504
H	3.4555	-1.3418	-3.4219
C	3.6389	0.8611	0.5744
H	4.6178	0.3732	0.4414
C	3.4962	1.2807	2.0326
H	4.2313	2.0619	2.2724
H	3.6640	0.4536	2.7328
H	2.4987	1.6980	2.2267
C	3.5638	2.0928	-0.3232
H	2.6250	2.6309	-0.1290
H	3.5882	1.8543	-1.3930
H	4.3950	2.7786	-0.1079
C	0.0704	1.8554	0.9377
C	0.0280	1.4265	-1.6191
Mo	-0.0424	0.2402	-0.1249
N	-0.1055	-1.4028	1.5838
O	0.1390	2.9865	1.3815
O	0.0875	2.2907	-2.4678
P	-2.4604	-0.1300	0.1230
P	2.3223	-0.3752	0.0674
H	-0.1478	-2.2755	1.0478
H	-0.1527	-1.2925	-1.2348
O	-0.2161	-4.2720	0.4131
C	-0.3043	-4.2906	-0.7963
H	-0.3782	-5.2523	-1.3480
Na	0.0397	3.8589	-0.7562
H	-0.3115	-3.3509	-1.3910

Hydride_transfer

Gibbs energy: -1217503.3 kcal mol-1

Atom	X	Y	Z
C	-2.4635	1.5331	-1.4016
H	-3.3788	1.4610	-2.0053
H	-2.5024	2.5042	-0.8845
C	-1.2295	1.4992	-2.2932
H	-1.2115	0.5924	-2.9151
H	-1.2333	2.3711	-2.9728
C	1.2282	1.5760	-2.2500

H	1.2033	2.4489	-2.9277
H	1.2908	0.6723	-2.8732
C	2.4189	1.6840	-1.3084
H	2.3405	2.6318	-0.7515
H	3.3615	1.7229	-1.8710
C	-3.2937	1.0796	1.3200
H	-2.7188	2.0195	1.3762
C	-3.1175	0.3705	2.6567
H	-3.4866	1.0061	3.4739
H	-2.0622	0.1419	2.8502
H	-3.6839	-0.5694	2.7024
C	-4.7561	1.4090	1.0510
H	-5.3820	0.5058	1.0573
H	-4.9094	1.9174	0.0898
H	-5.1480	2.0709	1.8362
C	-3.6235	-1.0526	-0.7131
H	-4.6055	-0.5542	-0.7469
C	-3.6993	-2.2480	0.2281
H	-4.3855	-3.0100	-0.1672
H	-4.0440	-1.9800	1.2335
H	-2.7068	-2.7109	0.3377
C	-3.2676	-1.5145	-2.1205
H	-2.2745	-1.9818	-2.1468
H	-3.2745	-0.6952	-2.8500
H	-3.9971	-2.2606	-2.4658
C	3.2770	1.1924	1.3853
H	2.5772	2.0229	1.5751
C	4.6507	1.7640	1.0591
H	4.9965	2.4088	1.8793
H	4.6596	2.3693	0.1437
H	5.4025	0.9709	0.9446
C	3.3280	0.3392	2.6460
H	4.0723	-0.4648	2.5573
H	2.3531	-0.1123	2.8698
H	3.6238	0.9515	3.5092
C	3.6727	-0.9078	-0.6544
H	4.6677	-0.4646	-0.4892
C	3.5048	-1.1912	-2.1422
H	4.2127	-1.9696	-2.4602
H	3.6914	-0.3099	-2.7673
H	2.4926	-1.5592	-2.3598
C	3.5657	-2.2130	0.1293
H	2.6233	-2.7149	-0.1331
H	3.5757	-2.0708	1.2165
H	4.3889	-2.8917	-0.1335
C	0.0662	-1.7939	-0.9981
C	0.0296	-1.4948	1.5808
Mo	0.0148	-0.2336	0.1468
N	-0.0133	1.4970	-1.4699
O	0.0980	-2.9008	-1.5022

O	0.0474	-2.4011	2.3861
P	-2.3885	0.2074	-0.0759
P	2.3992	0.3190	-0.0250
H	-0.0470	2.3361	-0.8846
H	-0.0282	1.2500	1.3330
O	-0.3448	4.3190	-0.2526
C	-0.3981	4.2340	0.9556
H	-1.0346	4.9204	1.5539
Na	0.1449	-3.8757	0.5967
H	0.1666	3.4614	1.5167

Post_hydride_transfer

Gibbs energy: -1217519.0 kcal mol-1

Atom	X	Y	Z
Mo	-0.0053	0.1196	-0.1208
P	2.4247	-0.3156	0.1660
P	-2.4334	-0.3181	0.1639
C	3.6376	1.0009	0.7020
C	2.4529	-1.5284	1.5845
C	3.3126	-1.2960	-1.1617
C	-0.0209	1.2176	-1.6898
C	-0.0110	1.8116	0.7052
C	-3.6414	1.0016	0.7028
C	-2.4626	-1.5358	1.5777
C	-3.3253	-1.2911	-1.1665
C	3.2446	1.5963	2.0482
C	3.7520	2.0919	-0.3544
H	4.6132	0.5013	0.8133
C	1.2082	-1.3881	2.4479
H	3.3640	-1.4315	2.1909
H	2.4566	-2.5337	1.1388
C	4.7883	-1.5558	-0.8898
C	3.0885	-0.7283	-2.5581
H	2.7762	-2.2578	-1.1098
O	-0.0371	2.0482	-2.5687
O	-0.0096	2.9761	1.0532
C	-3.2573	1.5832	2.0576
C	-3.7377	2.1024	-0.3455
H	-4.6213	0.5078	0.8011
C	-1.2204	-1.3942	2.4446
H	-3.3755	-1.4446	2.1823
H	-2.4626	-2.5397	1.1284
C	-3.1049	-0.7171	-2.5610
C	-4.8006	-1.5520	-0.8926
H	-2.7888	-2.2533	-1.1208
H	3.2279	0.8482	2.8498
H	3.9697	2.3682	2.3407

H	2.2548	2.0690	2.0052
H	2.7656	2.5375	-0.5540
H	4.4204	2.8932	-0.0106
H	4.1425	1.7208	-1.3090
N	-0.0049	-1.4190	1.6184
H	1.2178	-0.4420	3.0113
H	1.1825	-2.2081	3.1881
H	5.3886	-0.6412	-0.9925
H	5.1832	-2.2806	-1.6149
H	4.9674	-1.9686	0.1115
H	3.6022	0.2316	-2.7037
H	3.4807	-1.4233	-3.3133
H	2.0235	-0.5673	-2.7670
H	-3.2528	0.8287	2.8533
H	-3.9805	2.3572	2.3494
H	-2.2647	2.0507	2.0274
H	-2.7474	2.5462	-0.5272
H	-4.4085	2.9025	-0.0038
H	-4.1160	1.7409	-1.3086
H	-1.2346	-0.4497	3.0103
H	-1.1939	-2.2160	3.1829
H	-3.6206	0.2425	-2.7016
H	-3.4976	-1.4094	-3.3183
H	-2.0407	-0.5535	-2.7716
H	-5.4007	-0.6368	-0.9915
H	-5.1970	-2.2744	-1.6192
H	-4.9779	-1.9677	0.1079
H	-0.0011	-2.3440	1.0804
H	0.0724	-1.7901	-1.1117
Na	0.0269	3.8257	-1.0865
C	0.0192	-2.9647	-1.1506
H	0.8934	-3.1825	-1.8158
H	-0.8900	-3.1183	-1.7856
O	0.0193	-3.5681	0.0283

Proton_transfer

Gibbs energy: -1217519.7 kcal mol-1

Atom	X	Y	Z
Mo	0.0096	0.1074	0.1152
P	-2.4317	-0.3119	-0.1430
P	2.4287	-0.3673	-0.1260
C	-3.6138	0.9617	-0.8297
C	-2.4752	-1.6668	-1.4220
C	-3.3471	-1.1288	1.2746
C	0.0569	1.3248	1.5985
C	0.0365	1.7233	-0.8414
C	3.6035	0.8727	-0.8857

C	2.4448	-1.7836	-1.3353
C	3.3658	-1.1046	1.3224
C	-3.1880	1.4033	-2.2246
C	-3.7262	2.1601	0.1034
H	-4.5961	0.4687	-0.9044
C	-1.2040	-1.6607	-2.2594
H	-3.3690	-1.6020	-2.0576
H	-2.5262	-2.6173	-0.8720
C	-4.8201	-1.4096	1.0091
C	-3.1409	-0.4106	2.6026
H	-2.8181	-2.0942	1.3373
O	0.1048	2.2200	2.4110
O	0.0480	2.8495	-1.2986
C	3.1982	1.2179	-2.3137
C	3.6838	2.1313	-0.0313
H	4.5937	0.3914	-0.9134
C	1.2011	-1.7535	-2.2134
H	3.3602	-1.7987	-1.9437
H	2.4313	-2.7075	-0.7388
C	3.1261	-0.3431	2.6208
C	4.8493	-1.3402	1.0713
H	2.8738	-2.0861	1.4241
H	-3.1728	0.5735	-2.9415
H	-3.8923	2.1531	-2.6107
H	-2.1893	1.8584	-2.2138
H	-2.7349	2.6062	0.2763
H	-4.3706	2.9332	-0.3371
H	-4.1433	1.8987	1.0827
N	-0.0135	-1.6047	-1.4049
H	-1.1925	-0.8028	-2.9541
H	-1.1819	-2.5760	-2.8812
H	-5.4142	-0.4855	0.9930
H	-5.2339	-2.0422	1.8065
H	-4.9845	-1.9349	0.0593
H	-3.6657	0.5537	2.6378
H	-3.5342	-1.0218	3.4265
H	-2.0794	-0.2162	2.8011
H	3.2333	0.3489	-2.9816
H	3.8858	1.9702	-2.7244
H	2.1848	1.6374	-2.3557
H	2.6884	2.5893	0.0679
H	4.3484	2.8717	-0.4973
H	4.0606	1.9368	0.9798
H	1.2547	-0.9270	-2.9428
H	1.1532	-2.6914	-2.7986
H	3.5389	0.6745	2.5897
H	3.6085	-0.8650	3.4588
H	2.0563	-0.2545	2.8468
H	5.4094	-0.3959	1.0298
H	5.2818	-1.9330	1.8889

H	5.0366	-1.8869	0.1379
H	-0.0631	-2.5397	-0.6942
H	-0.1499	-1.7268	1.3364
Na	-0.0039	3.8566	0.7742
C	-0.0635	-2.8763	1.4062
H	-0.8872	-3.1170	2.1165
H	0.8856	-3.0224	1.9725
O	-0.1318	-3.5112	0.2242

Post_proton_transfer

Gibbs energy: -1217520.4 kcal mol-1

Atom	X	Y	Z
C	2.7145	-1.2879	-2.1696
H	3.7542	-1.4513	-2.4875
H	2.1265	-2.1451	-2.5270
C	2.1468	-0.0088	-2.7695
H	2.9116	0.7947	-2.7553
H	1.9372	-0.1969	-3.8429
C	0.3013	1.4643	-2.9107
H	0.1548	1.0910	-3.9457
H	0.9685	2.3446	-3.0111
C	-1.0555	1.8925	-2.3688
H	-1.8030	1.1237	-2.6165
H	-1.3920	2.8319	-2.8310
C	2.3433	-3.1161	0.0205
H	1.5439	-3.3763	-0.6939
C	1.8127	-3.4060	1.4185
H	1.6334	-4.4837	1.5371
H	0.8679	-2.8836	1.6075
H	2.5188	-3.1052	2.2034
C	3.5716	-3.9576	-0.3025
H	4.3843	-3.7772	0.4145
H	3.9618	-3.7675	-1.3106
H	3.3246	-5.0266	-0.2445
C	4.2864	-0.8650	0.2507
H	4.9609	-1.6020	-0.2142
C	4.4126	-0.9501	1.7664
H	5.3955	-0.5799	2.0890
H	4.3116	-1.9761	2.1400
H	3.6479	-0.3349	2.2606
C	4.6687	0.5249	-0.2467
H	3.9727	1.2869	0.1321
H	4.6739	0.5925	-1.3417
H	5.6764	0.7876	0.1032
C	-2.8489	1.7243	-0.1233
H	-3.0557	0.8136	-0.7096
C	-3.7817	2.8293	-0.6017

H	-4.8291	2.5125	-0.5017
H	-3.6206	3.0919	-1.6548
H	-3.6659	3.7444	-0.0047
C	-3.1044	1.3739	1.3380
H	-2.9142	2.2277	2.0056
H	-2.4906	0.5242	1.6611
H	-4.1570	1.0946	1.4832
C	-0.7326	3.8169	-0.1991
H	-1.5502	4.3661	-0.6928
C	0.5922	4.2487	-0.8192
H	0.8163	5.2890	-0.5458
H	0.5780	4.1947	-1.9137
H	1.4220	3.6217	-0.4603
C	-0.7390	4.1453	1.2899
H	0.1116	3.6581	1.7884
H	-1.6734	3.8581	1.7892
H	-0.6141	5.2262	1.4418
C	1.5236	1.3489	1.4250
C	-0.0704	-0.4253	1.6569
Mo	0.7009	0.3232	0.0703
N	0.9315	0.4351	-2.0921
O	1.9306	1.9854	2.3753
O	-0.5286	-0.7627	2.7266
P	2.5561	-1.2845	-0.3171
P	-1.0315	1.9955	-0.5137
H	-0.1353	-0.9775	-2.5483
H	-1.0089	-1.6131	-0.7090
O	-0.7095	-1.7547	-2.7727
C	-1.4461	-2.0647	-1.6252
H	-1.4684	-3.1529	-1.4689
Na	-0.1092	1.4395	3.4483
H	-2.4925	-1.7208	-1.6977

Literature

[1]T. Leischner, A. Spannenberg. K. Junge, M. Beller, *Organometallics*, 2018, 37, 4332-4335.

[2]J. Chen, W. Long, S. Fang, Y. Yang, X. Wan, *Chem. Commun.*, 2017, 53, 13256-13259.

[3]R. Bisht, M. E. Hoque, B. Chattopadhyay, *Angew. Chem. Int. Ed.*, 2018, 57, 15726-15766.

[4]P. V. Ilyin, A. S. Pankova, M. A. Kuznetzov, *Synthesis*, 2012, 44, 1353-1358.

[5]H. Taneda, K. Inamoto, Y. Kondo, *Chem. Commun.*, 2014, 50, 6523-6525.

[6]L. Artús Suàrez, Z. Culakova, D. Balcells, W. H. Bernskoetter, O. Eisenstein, K. I. Goldberg, N. Hazari, M. Tilset, A. Nova, *ACS Catal*, 2018, 8, 8751-8762.

[7]S. Hoops, S. Sahle, R. Gauges, C. Lee, J. Pahle, N. Simus, M. Singhal, L. Xu, M. Pedro, U. Kummer, *Bioinformatics,* 2006, 22, 3067-3074.

[8]E. Brunner, *J. Chem. Eng. Data.* 1985, 30, 269-273.

[9]L. Petzold, *SIAM. J. Sci. and Stat. Comput.* 1983, 4, 136-148.

[10]M. J. Frisch, G. W. Trucks, H. B. Schlegel, G. E. Scuseria, M. A. Robb, J. R. Cheeseman, G. Scalmani, V. Barone, B. Mennucci, G. A. Petersson, H. Nakatsuji, M. Caricato, X. Li, H. P. Hratchian, A. F. Izmavlov, J. Blonio, G. Zheng, J. L. Sonnenberg, M. Hada, M. Ehara, K. Toyota, R. Fukuda, J. Hasegawa, M. Ishida, T. Nakajima, Y. Honda, O. Kitao, H. Nakai, T. Verve, J. A. Montgomery, J. E. Peralta, F. Ogliare, M. Bearpark, J. J. Heyd, E. Brothers, K. N. Kudin, V. N. Staroverov, T. Keith, R. Kobayashi, J. Normand, K. Raghvachari, A. Rendell, J. C. Burant, S. S. Ivengar, J. Tomasi, M. Cossi, N. Rega, J. M. Millam, M. Klene, J. E. Knox, J. B. Cross, V. Bakken, C. Adamo, J. Jaramillo, R. Gomperts, R. E. Stratmann, O. Yazyev, A. J. Austin, R. Cammi, C. Pomelli, J. W. Ochterski, R. L. Martin, K. Morokuma, V. G. Zakrezweski, G. A. Voth, P. Salvador, J. J. Dannenberg, S. Dapprich, A. D. Daniels, O. Fakas, J. B. Foresman, J. V. Ortiz, J. Cioslowski, G. A. Voth, *Gaussian 09*, Revision D.01; Gaussian, Inc.: Wallingford CT, 2013.

[11]Y. Zhao, D. Truhlar, Theor. *Chem. Acc.*, 2008, 120, 215-241.

[12](a) P. Hay, W. Wadt, *J. Chem. Phys.,* 1985, 82, 270-283 (b) W. Wadt, P. Hay, *J. Chem. Phys.*, 1985, 82, 284-298.

[13](a) W. J. Hehre, R. Ditchfield, J. A. Pople, *J. Chem. Phys.*, 1972, 56, 2257-2261 (b) W. Kohn, A. D. Becke, R. G. Parr, *J. Phys. Chem.*, 1996, 1, 12974-12980.

[14]A. McLean, G. Chandler, *J. Chem. Phys.*, 1980, 72, 5639-5648.

[15]A. Marenich, C. Cramer, D. Truhlar, *J. Phys. Chem. B.*, 2009, 113, 6378-6396.

[16]M. Álvarez-Moreno, C. De Graaf, N. López, F. Maseras, J. M. Poblet, C. Bo, *J. Chem. Inf. Model.*, 2015, 55, 95-103.

[17]S. Hoops, R. Gauges, C. Lee, J. Pahle, N, Simus, M. Singhal, L. Xu, P. Mendes, U. Kummez, *Bioinformatics*, 2006, 22, 3067-3074.
